# The N6-methyladenosine METTL3 regulates tumorigenesis and glycolysis by mediating m6A methylation of the tumor suppressor LATS1 in breast cancer

**DOI:** 10.1186/s13046-022-02581-1

**Published:** 2023-01-07

**Authors:** Youqin Xu, Mu Song, Ziyang Hong, Wancheng Chen, Qianbing Zhang, Jianlong Zhou, Chao Yang, Zilong He, Juanjuan Yu, Xiaolin Peng, Qiuhong Zhu, Shaotian Li, Kaiyuan Ji, Minfeng Liu, Qiang Zuo

**Affiliations:** 1 Department of Thyroid and Breast Surgery, The Seventh Affiliated Hospital, Southern Medical University, Foshan, 528200 China; 2 Department of Oncology, Nanfang Hospital, Southern Medical University, Guangzhou, 510515 China; 3 Institute of Oncology, School of Basic Medical Sciences, Southern Medical University, Guangzhou, 510515 China; 4 Department of Radiotherapy, Zhujiang Hospital, Southern Medical University, Guangzhou, 510282 China; 5 Department of Oncology, Guangxi International Zhuang Medicine Hospital, Nanning, 530021 China; 6 Department of Laboratory Medicine, Nanfang Hospital, Southern Medical University, Guangzhou, 510515 China; 7 Department of Radiology, Nanfang Hospital, Southern Medical University, Guangzhou, 510515 China; 8 Guangzhou Women and Children’s Medical Center, Guangzhou Medical University, Guangzhou, 510620 China; 9 Breast Center, Department of General Surgery, Nanfang Hospital, Southern Medical University, Guangzhou, 510515 China

**Keywords:** Breast cancer, METTL3, LATS1, Hippo-YAP/TAZ signaling pathway

## Abstract

**Background:**

Posttranscriptional modification of tumor-associated factors plays a pivotal role in breast cancer progression. However, the underlying mechanism remains unknown. M6A modifications in cancer cells are dynamic and reversible and have been found to impact tumor initiation and progression through various mechanisms. In this study, we explored the regulatory mechanism of breast cancer cell proliferation and metabolism through m6A methylation in the Hippo pathway.

**Methods:**

A combination of MeRIP-seq, RNA-seq and metabolomics-seq was utilized to reveal a map of m6A modifications in breast cancer tissues and cells. We conducted RNA pull-down assays, RIP-qPCR, MeRIP-qPCR, and RNA stability analysis to identify the relationship between m6A proteins and LATS1 in m6A regulation in breast cancer cells. The expression and biological functions of m6A proteins were confirmed in breast cancer cells in vitro and in vivo. Furthermore, we investigated the phosphorylation levels and localization of YAP/TAZ to reveal that the activity of the Hippo pathway was affected by m6A regulation of LATS1 in breast cancer cells.

**Results:**

We demonstrated that m6A regulation plays an important role in proliferation and glycolytic metabolism in breast cancer through the Hippo pathway factor, LATS1. METTL3 was identified as the m6A writer, with YTHDF2 as the reader protein of LATS1 mRNA, which plays a positive role in promoting both tumorigenesis and glycolysis in breast cancer. High levels of m6A modification were induced by METTL3 in LATS1 mRNA. YTHDF2 identified m6A sites in LATS1 mRNA and reduced its stability. Knockout of the protein expression of METTL3 or YTHDF2 increased the expression of LATS1 mRNA and suppressed breast cancer tumorigenesis by activating YAP/TAZ in the Hippo pathway.

**Conclusions:**

In summary, we discovered that the METTL3-LATS1-YTHDF2 pathway plays an important role in the progression of breast cancer by activating YAP/TAZ in the Hippo pathway.

**Supplementary Information:**

The online version contains supplementary material available at 10.1186/s13046-022-02581-1.

## Background

The latest global burden of cancer released by the International Agency for Research on Cancer (IARC) in 2020 declared breast cancer to be the world's leading cancer [1]. Owing to the high heterogeneity of breast cancer, there are considerable differences in the diagnosis, treatment, and prognosis of different breast cancer types. It is widely recognized that the axillary lymph node metastasis rate in patients with luminal B breast cancer is higher than that in patients with luminal A disease, whereas the axillary lymph node metastasis rate in patients with triple-negative breast cancer (TNBC) is lower. Patients with the luminal A type generally have a better survival rate than those with other types [2]. Endocrine therapy is usually the preferred choice of treatment for patients with luminal A and luminal B tumors. Patients with HER-2 overexpression are sensitive to targeted therapy, while patients with TNBC experience rapid clinical progression [3]. The heterogeneity of breast cancer is also internally reflected in differences in the genome, transcriptome, proteome, and metabolome [4].

Nonmutational epigenetic reprogramming is one of the 14 features of cancer listed in the third edition of Hallmarks of Cancer [5]. As the most abundant internal modification of mammalian mRNA, N6-methyladenosine (m6A) modification is involved in multiple aspects of RNA metabolism, including RNA stability, translation, splicing, transport, and localization, which have been discovered to have an impact on tumor initiation and progression through various mechanisms [6]. The m6A protein affects tumor proliferation and metastasis in breast cancer and is closely related to the prognosis of patients [7–9]. However, other potential mechanisms remain unclear. The fate of m6A modification of mRNA varies according to different reader proteins. Therefore, it is necessary to conduct systematic sequencing and bioinformatics analysis of the levels of m6A modification in key pathways to examine the development of breast cancer.

The Hippo pathway plays an important role in organ development and serves as a tumorigenesis suppressor. Following stimulation with extracellular growth inhibition signals, a series of kinase cascades, such as LATS1/LATS2, are activated, resulting in phosphorylation of the effector factor Yes-associated protein (YAP) and transcriptional coactivator with PDZ-binding motif (TAZ). Notably, recent findings have implicated YAP and TAZ in the metabolism of cancer cells [10]. The Hippo pathway controls cell proliferation and organ size by binding to cytoskeletal proteins and remaining in the intracytoplasmic space as well as by reducing nuclear activity. LATS1 has also been reported to be a tumor suppressor [11]. We previously found that HERC4 is an E3 ligase for the tumor suppressor LATS1 and destabilizes LATS1 by promoting its ubiquitination of LATS1 [12].

Whether m6A modification occurs in the Hippo pathway remains unclear. Recent studies have shown that METTL3 is highly expressed in breast cancer [9] and can be regulated by multiple noncoding RNAs such as miR-483-3p [13], let-7 g [7] and pri-miR-221-3p [8]. Moreover, m6A-mediated lincRNAs, such as LINC00958 [14], were found to regulate breast cancer tumorigenesis. Here, we demonstrated that LATS1 mRNA m6A modification mediated by METTL3 and recognized by YTHDF2, plays a positive role in promoting both tumorigenesis and glycolysis in breast cancer.

## Methods

### Breast cancer patient tumor samples

After obtaining adequate informed consent, breast cancer tissue and adjacent normal tissue were obtained from 8 patients who underwent curative resection for breast cancer at Nanfang Hospital of Southern Medical University, between November 2019 and May 2020. All patients satisfied the following inclusion criteria: the surgical margins were confirmed to contain no residual carcinoma tissue; clinicopathological information on age, sex, clinical stage, neoadjuvant therapy, ER, PR, HER2, Ki67, histological subtype and histological grade was available and is listed in Supplementary Table 1. All patient-related studies were approved by the institutional review boards of the Seventh Affiliated Hospital of Southern Medical University and Nanfang Hospital of Southern Medical University.

### Human cancer cell lines and cell culture

Human breast cancer cell lines (SK-BR-3, MCF-7, T47D, BT474, MDA-MB-468, and MDA-MB-231) were purchased from American Type Culture Collection (ATCC, Manassas, VA, USA). MCF-7 cells were cultured in MEM (Gibco cat:11,095) supplemented with 10% fetal bovine serum (FBS, HyClone, Utah, USA), 1% penicillin‒streptomycin (Gibco), 10 μg/ml insulin (CellCook, cat: CM1007) and 1⨯ nonessential amino acids (CellCook, cat: CM10085/L). SK-BR-3, MCF-7, T47D, BT474, MDA-MB-468, and MDA-MB-231 cells were cultured in RPMI 1640 (Gibco, cat:11,875) supplemented with 10% fetal bovine serum (FBS; HyClone, Utah, USA) and 1% penicillin‒streptomycin (Gibco).

### Establishment of transfected cell lines

For CRISPR‒Cas9 cells, 100 μM sgRNA Top/Bottom Oligo solution was configured with ddH2O, and the sgRNA sequences were as follows: sgMETTL3:5’-ATTCTGTGACTATGGAACCA-3’, sgYTHDF1 5’-AGTTTCAAAGCCGACCTCGT-3’, sgYTHDF2 5’-GTCCATTACTAGTAACATCG-3’, and sgYTHDF3: CAACCGAAACTTAAACCCAA-3’. PCR was used to produce linked products that were transformed into DH5α cells. Lentiviral overexpression plasmids for human METTL3 and YTHDF2 were transfected into cells as previously described [15]. Cells were selected using puromycin (2 μg/ml, Gene-Chem) for 3 days for stable transfection. 293 T cells (ATCC Cat# CRL-3216) were used to generate lentiviral particles by cotransfecting the packaging vectors using a transfection reagent (SignaGen, SL100668), following the manufacturer’s instructions.

### Western blot analysis

Lysis buffer was used for total protein analysis of extracted cells. Samples were separated on an 8–15% gel by SDS-PAGE. Nitrocellulose membranes were blocked with blocking buffer and incubated with the appropriate primary antibody. The membranes were washed with blocking buffer three times, probed with the appropriate secondary antibody and developed using SuperSignal West Pico or Dura (Thermo Fisher Scientific).

### Quantitative PCR analysis

Real-time PCR analysis was performed by using the Biosystems Step-One-Plus Real-Time PCR System with FastStart Universal SYBR Green Master Mix (Roche). The primer sets used were as follows.

GAPDH-F: GAACGGGAAGCTCACTGG,

GAPDH-R: GCCTGCTTCACCACCTTCT,

METTL3-F: AGATGGGGTAGAAAGCCTCCT,

METTL3-R: TGGTCAGCATAGGTTACAAGAGT,

LATS1-F: AATTTGGGACGCATCATAAAGCC,

LATS1-R: TCGTCGAGGATCTTGGTAACTC,

YTHDF2-F: TAGCCAACTGCGACACATTC, and

YTHDF2-R: CACGACCTTGACGTTCCTTT.

The PCR conditions were as follows: 10 min at 95 °C and 40 cycles of 15 s at 95 °C and 1 min at 60 °C. The average Ct value for each gene was determined from triplicate reactions and was normalized as previously reported.

### Cell proliferation, apoptosis, migration and invasion assays

For the EdU cell proliferation assay, the cells were trypsinized and seeded onto 6-well plates at a density of 1 × 10^6^ cells per well. After incubation for 24 h at 37 °C, 1 ml medium was added to each well and incubated for 2 h after discarding the old medium. The cells were collected in flow tubes and centrifuged at 1,500 rpm for 5 min. The supernatant was discarded and the cells were resuspended in PBS and centrifuged at 1,500 rpm for 5 min. After discarding the supernatant, the cells were fixed with 4% paraformaldehyde for 15–30 min, neutralized with glycine for 5 min, and resuspended in PBS. The cells were then incubated with 0.5% Triton X-100 penetrant at room temperature for 10 min. One hundred microliters of 1⨯ Apollo staining reaction solution was added to each tube and the cells were resuspended again. After incubation for 10 min at room temperature, the staining reaction solution was discarded by centrifugation at 1,500 rpm for 5 min. Next, 0.5% Triton X-100 penetrant was used to clean the cells 1–3 times at room temperature and the cells were resuspended in PBS. Flow cytometry was performed immediately after staining.

### Human cancer cell xenograft model

All animal experiments were approved by the Institutional Animal Care and Use Committee of Southern Medical University. Breast cancer cells (5 × 10^6^) were implanted into the subcutaneous axilla of the forelimb of 3–4-week-old BALB/c nude mice. Seven days after transplantation, the diameter of the tumors was measured, and the tumors were removed after three weeks.

### Coimmunoprecipitation assay

Immunoprecipitation assays were performed as previously described [16]. Cells were lysed in radioimmunoprecipitation assay buffer containing protease and phosphatase inhibitors. The cells were then centrifuged at 12,000 × g for 10 min at 4 °C and collected. The supernatants were immunoprecipitated with antibodies and magnetic protein A/G beads (Pierce) were used for incubation for 2 h at 4 °C. The above immune complexes were washed with PBS and resuspended in SDS-PAGE buffer, followed by western blotting analysis.

### MeRIP-seq

Total RNA was isolated from cells and tissues using Magzol Reagent (Magen, China), according to the manufacturer’s protocol. The quantity and integrity of the RNA yield were assessed using a K5500 microspectrophotometer (Beijing Kaiao, China) and the Agilent 2200 TapeStation system (Agilent Technologies, USA), respectively. m6A antibody-immunoprecipitated RNA was quality controlled using the Qubit (Thermo Fisher Scientific, USA) and Agilent 2200 TapeStation (Agilent Technologies, USA) systems. Briefly, RNA was fragmented into molecules approximately 200 bp in length. The RNA fragments were then subjected to first-strand and second-strand cDNA synthesis, followed by adaptor ligation and enrichment with a low cycle according to the instructions of the NEBNext® Ultra RNA LibraryPrep Kit for Illumina (NEB, USA). The final library product was assessed using the Agilent 2200 TapeStation and Qubit® system (Life Technologies, USA), and then sequenced on an Illumina platform (Illumina, USA) with a paired-end length of 150 bp at RiboBio Co., Ltd. (Ribobio, China). Adaptor and low-quality bases were trimmed using Trimmomatic tools (version: 0.36), and the clean reads were subjected to rRNA deletion through RNAcentral to obtain effective reads. Genomic alignment was performed using TopHat (version 2.0.13) to obtain uniquely mapped reads. Effective reads from the input sample were used for RNA-seq analysis and the read count value of each transcript was calculated using HTSeq (version 0.6.0). Differentially expressed genes were identified using the DEseq/DESeq2/edgeR/DEGseq R package according to the following criteria: |log2 (fold change)|≥ 1 and *P* value < 0.05.

### Sequence-based RNA adenosine methylation site predictor (SRAMP)

SRAMP is a site prediction tool based on a random forest machine-learning framework that can predict m6A sites based on sequence-derived features. The m6A locus was predicted using the target sequence [17].

### Metabolomic analysis

A high-resolution mass spectrometer (QEXactive, Thermo Fisher Scientific, USA) was used to collect positive ion (POS) and negative ion (NEG) data from 18 cell samples for untargeted metabolomics detection using LC‒MS/MS technology to explore the metabolomic composition and biological function of the samples. Compound Discoverer 3.1.0 (Thermo Fisher Scientific, USA) software was used for data processing, including peak extraction, peak alignment, fill gaps, and compound identification. An in-house metabolome information analysis process was used to carry out metabolite annotation, classification (Kyoto Encyclopedia of Genes and Genomes [KEGG], Human Metabolome Database [HMDB]), and enrichment analysis for the identified substances, and to explain the physical and chemical properties and biological functions of the metabolites. The R software package metaX [18] was used for data preprocessing, statistical analysis (univariate and multivariate analyses), and screening of metabolites with significant differences. Discriminant analysis was performed using partial least squares discriminant analysis (PLS-DA). Differentially expressed metabolites were screened by the VIP values of the first two principal components of the model [19] and Student’s t-test was used to analyze the results of the univariate analysis (fold change).

### Transcriptome sequencing

Total RNA was extracted using TRIzol reagent (Thermo Fisher, 15,596,018) as previously reported [12]. Total RNA quantity and purity were analyzed using a Bioanalyzer 2100 and RNA 6000 Nano LabChip Kit (Agilent, CA, USA, 5067–1511). Additionally, mRNA was purified from total RNA (5 μg) using Dynabeads Oligo (dT) (Thermo Fisher Scientific, CA, USA) with two rounds of purification and fragmented into short fragments using divalent cations at an elevated temperature (Magnesium RNA Fragmentation Module (NEB, cat. e6150, USA) at 94 °C for 5–7 min). Then, the cleaved RNA fragments were reverse-transcribed to create cDNA with SuperScript™ II Reverse Transcriptase (Invitrogen, cat.1896649, USA). Dual-index adapters were used and size selection was performed by using AMPureXP beads. The U-labeled second-stranded DNAs were treated with the heat-labile UDG enzyme (NEB, cat.m0280, USA). PCR was used to amplify to the ligated products under the following conditions: 95 °C for 3 min; 8 cycles of denaturation at 98 °C for 15 s, 60 °C for 15 s, and 72 °C for 30 s; and 72 °C for 5 min. Finally, 2 × 150 bp paired-end sequencing (PE150) was performed on an Illumina Novaseq 6000 (LC-Bio Technology Co., Ltd., Hangzhou, China).

### RNA stability analysis

Cells in the treatment and control groups were collected after 24 h. RNA was extracted using TRIzol reagent, and mRNA expression was determined by qPCR.

### Seahorse assay

On the same day that the cells were seeded, 180 μl of hydration solution was added to the lower part of the XF96 Extracellular Flux Assay Kit. Cells were hydrated overnight in an incubator without CO_2_ at 37 °C. The required drugs and Seahorse XF basic culture medium were prepared simultaneously, and the pH of the medium was adjusted to 7.4. The cells were then placed in a 37 °C water bath for 1 h before use. On the second day, the cells were washed twice with Seahorse XF basic culture medium in a water bath and 175 μl Seahorse XF basic culture medium was added to each well. The cells were then cultured in a CO_2_-free incubator at 37 °C for 1 h. Each drug was diluted to the required concentration, and 25 μl was added to each well of an XF96 extracellular flux assay kit. Thirty minutes later, the lower part of the XF96 Extracellular Flux Assay Kit was removed and replaced with cell plates that had been cultured in a CO_2_-free incubator at 37 °C for 1 h and assessed with a computer.

### RIP-qPCR

Endogenous RNA was retrieved after trapping in the nucleus or cytoplasm with an antibody or epitope marker. The RNA-binding protein was separated from the bound RNA by immunoprecipitation. The cells were crosslinked with 1% formaldehyde and treated with 150 mM NaCl RIPA buffer containing RNase and protease inhibitors. Cells were lysed in 0.5% sodium deoxycholate, 0.1% SDS, 1% NP40, 1 mM EDTA, and 50 mM Tris (pH 8.0) for 30 min and centrifuged for precipitation. The supernatant was incubated four times with METTL3, YTHDF1, or YTHDF2 primary antibody or the corresponding IgG antibody for 4 h, and protein A/G glycosylated beads were added and incubated for 2 h with shaking. After washing three times with RIPA buffer, RNA was extracted after crosslinking. Quantitative RT-PCR was performed to measure RNA.

### RNA pulldown assays

RNA pulldown assays were performed in accordance with the Pierce™ Magnetic RNA‒ Protein Pull-down Kit protocol. RNA was prepared by in vitro transcription and biotin was used. Poly (A) 25 RNA was used as the negative control. Total protein was extracted from each group, and the required protein concentration was greater than 2 mg/ml. RNA pull-down was performed according to a standard protocol, and the expression level was confirmed by western blotting.

### Statistical analysis

Data were analyzed using SPSS 20.0. With a two-tailed independent Student’s t-test, *p* < 0.05 was considered significant. Two patient cohorts were compared using a Kaplan–‒Meier survival plot, and log-rank *p* value were calculated.

## Result

### Aberrant m6A methylation levels of Hippo pathway proteins were found in breast cancer

**Fig. 1:**
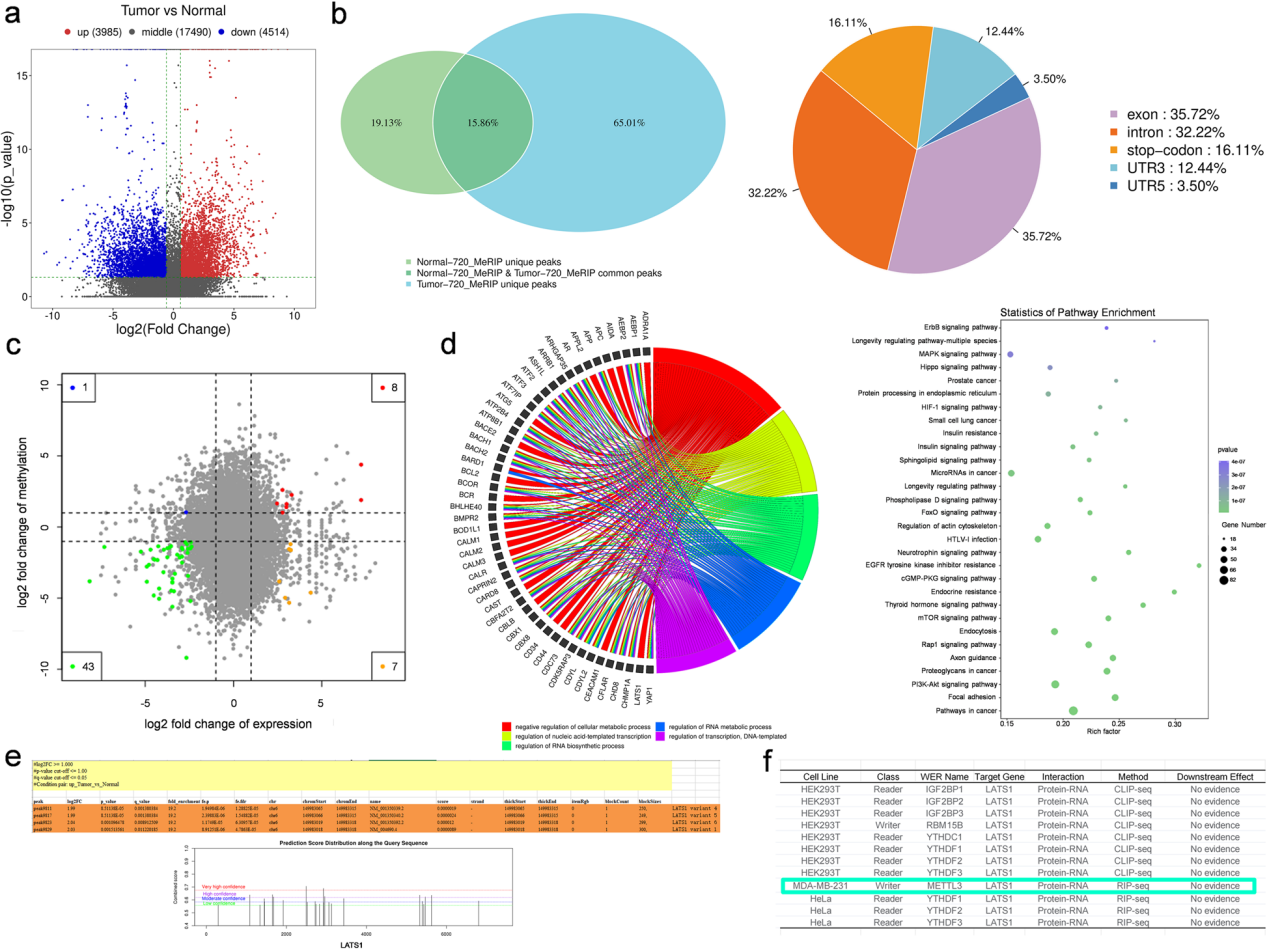
Abnormal levels of m6A modified mRNA are related to multiple pathways in breast cancer. **a** The volcano plot of differential methylation sites of breast cancer tissues compared with adjacent normal tissues, *n* = 2, |log2(fold change)|> 1 and *p* value < 0.05. **b** Venn diagram (left panel) of common (intersection region) and unique peaks (non-intersection region) of breast cancer tissue compared with adjacent normal tissue, |log2(fold change)|> 1 and *p* value < 0.05. The gene region (right panel) annotated with different peaks of breast cancer tissue compared with adjacent normal tissue, |log2(fold change)|> 1 and *p* value < 0.05. **c** Differential m6A and differential gene expression combined analysis diagram. Blue dots represent transcripts with significantly downregulated and upregulated m6A expression, red dots represent transcripts with significantly upregulated expression and upregulated m6A expression, green dots represent transcripts with significantly downregulated and downregulated m6A expression, and yellow dots represent transcripts with significantly upregulated and downregulated m6A expression. **d** Functional enrichment of differentially expressed genes with different m6A methylation sites (left panel). The KEGG pathway enrichment bubble map of differential m6A methylation and differentially expressed genes (right panel). **e** List of LATS1 containing transcripts of differentially methylated sites (upper panel) in breast cancer tissues compared with adjacent normal tissues, *n* = 2, |log2(fold change)|> 1 and *p* value < 0.05. SRAMP prediction results of m6A sites on LATS1 (lower panel). **f** m6A2Target prediction results of m6A proteins related to LATS1

To investigate m6A modification levels in breast cancer tissue samples, MeRIP-seq was conducted for paired breast ductal cancer tissues and adjacent normal breast tissue samples. The corresponding sequencing data have been uploaded to the GEO database (GSE217977, www.ncbi.nlm.nih.gov/geo/). By analyzing the sequencing data, we found that large amounts of differentially methylated or modified mRNAs existed in cancer tissue (Fig. 1a). The differential enrichment peaks of each pair revealed that more than half of the m6A-specific sites were enriched in tumor tissue and appeared near the classic mRNA-modified regions, namely exons, 3’UTRs, and stop codons (Fig. 1b and Supplementary Fig. 1a). Combined analysis of differentially expressed m6A genes and mRNAs helped us discover the influence of m6A modification on mRNA expression, mRNA abundance, and differentially expressed genes. Figure 1c and Supplementary Fig. 1b show the results of the combined analysis of the differentially expressed m6A mRNAs and differentially expressed genes. Functional enrichment analysis of these differentially expressed genes (Fig. 1d, right panel and Supplementary Fig. 1c-d) further revealed that m6A modification plays an important role in regulating multiple cancers and pathways, such as the MAPK, Hippo, and PI3K-Akt signaling pathways. Notably, genes in the Hippo pathway were found to be differentially modified and expressed in breast cancer tissue (Fig. 1d, left panel and Supplementary Fig. 1e). The Hippo pathway is a significant proliferation-regulating pathway involved in cancer development. The results showed that m6A modification of the tumor suppressor LATS1 may be involved in the downregulation of mRNA expression (Supplementary Fig. 1f-g) and negative regulation of cellular metabolic processes (Fig. 1d, left panel). MeRIP-seq results revealed that the m6A modification level of LATS1 mRNA was significantly upregulated at more than two sites (Fig. 1e, upper panel, Supplementary Table 2 and Supplementary Fig. 1h). To determine the mechanism of m6A modification of LATS1 in breast cancer, we used SRAMP to predict the m6A sites in LATS1 mRNA (Fig. 1e, lower panel). m6A2Target (http://m6a2target.canceromics.org/#/), a database for predicting the target genes of m6A writers, erasers, and readers, was used to search for proteins related to m6A modification of LATS1 mRNA. As shown in Fig. 1f, the m6A writer protein mettl3 was predicted to be the binding protein of LATS1 mRNA during m6A modification in MDA-MB-231 breast cancer cells.

### Expression of METTL3 could be an independent factor affecting the prognosis of breast cancer patients and promoting tumorigenesis of breast cancer cells

**Fig. 2:**
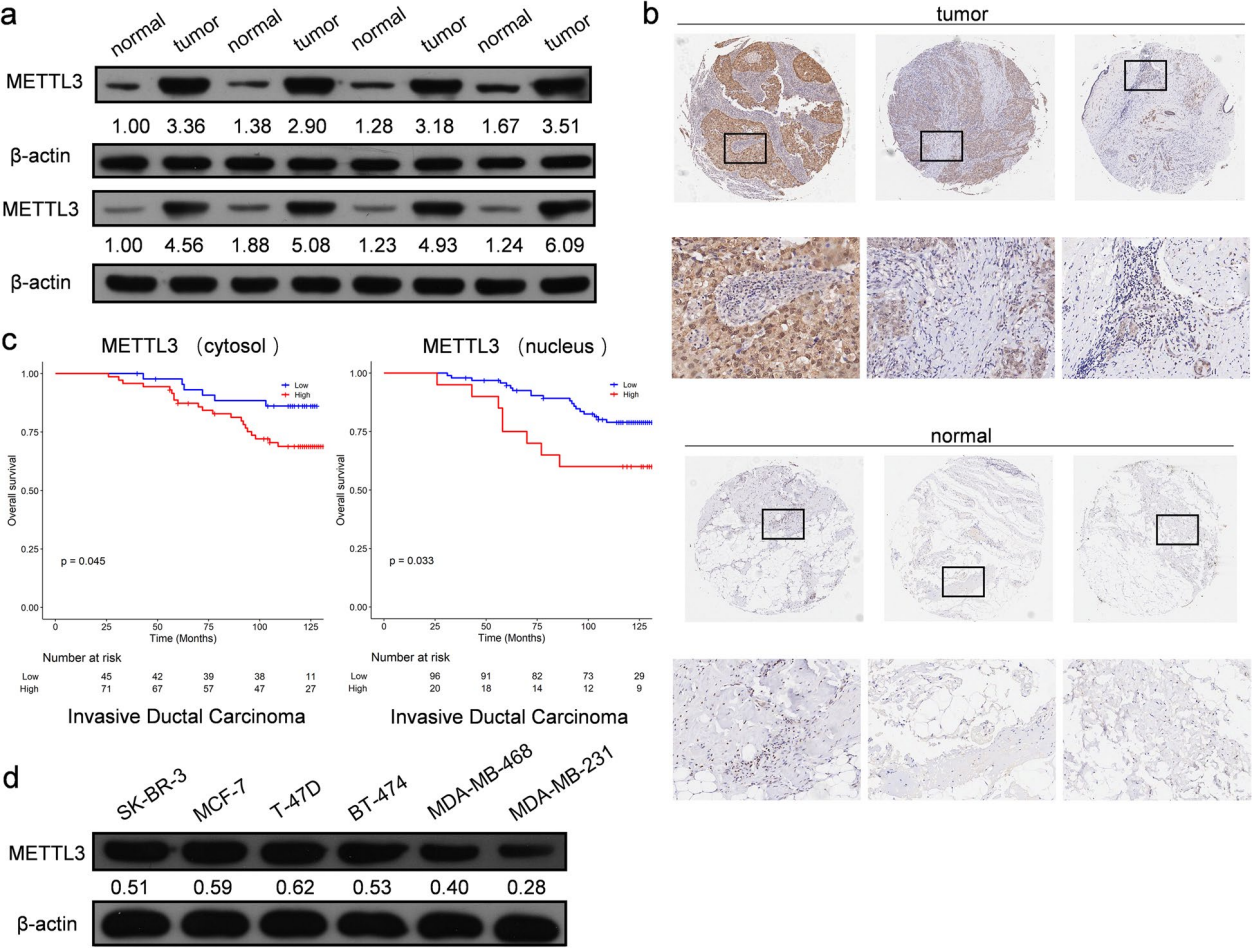
Changes in METTL3 expression were found in breast cancer tissues. **a** The protein level of METTL3 in breast cancer tissues and adjacent normal tissues, *n* = 8. **b** The expression of METTL3 protein in breast cancer tissues and normal tissues by IHC in the TMA (129 breast cancer tissue samples and 3 normal breast tissue samples). Strong staining of mettl3 was detected in breast cancer tissues (upper panel) and weak staining was detected in normal tissues (lower panel). **c** High METTL3 protein expression was significantly associated with poor prognosis of invasive ductal carcinoma, *p* < 0.05. **d** The protein level of METTL3 in 6 different breast cancer cell lines

To investigate the expression of METTL3 in breast cancer, we examined its protein levels in breast cancer tissues and paired adjacent normal tissues. As shown in Fig. 2a, Mettl3 was highly expressed in breast ductal carcinoma tissues. Bioinformatics analysis was applied to the expression data of breast tumor tissues and tumor-adjacent normal tissues in the Gene Expression Omnibus (GEO) database (GSE70951, 195 breast adenocarcinomas and matched adjacent normal breast tissue samples). We found that the expression levels of ER/PR/HER2 were not correlated with those of Mettl3 (Supplementary Fig. 2a). However, the immunohistochemistry (IHC) results conducted in breast cancer tissue microarrays (129 breast cancer tissue samples and three normal breast tissue samples) revealed that Mettl3 was expressed mostly in the cytoplasm and nuclei of cancer tissues, with a few expression in the cell membrane (Fig. 2b). The relationship between Mettl3 and patient clinicopathological features is shown in Supplementary Table 3, and the results of univariate and multivariate analyses of the factors that correlated with the overall survival of cancer patients are shown in Table 1. Kaplan–Meier survival analysis revealed that high METTL3 protein expression was significantly associated with poor prognosis in invasive ductal carcinoma and luminal breast cancer tissues (Fig. 2c and Supplementary Fig. 2b). Moreover, we examined the protein level of METTL3 in six different breast cancer cell types. The results are shown in Fig. 2d. Mettl3 showed the highest expression in T47D cells, followed by MCF-7 cells, and the lowest expression in MDA-MB-231 cells.

**Table 1:** The univariate and multivariate analyses of the factors that correlated with the overall survival of cancer patients

	Cytosol expression								Nucleus expression							
Variables	Univariate analysis				Multivariate analysis				Univariate analysis				Multivariate analysis			
	*p* value	HR	95%CI		*p* value	HR	95%CI		*p* value	HR	95%CI		*p* value	HR	95%CI	
			Lower limit	Upper limit			Lower limit	Upper limit			Lower limit	Upper limit			Lower limit	Upper limit
METTL3expression	0.029	2.422	1.095	5.356	0.04	2.31	1.04	5.13	0.56	1.247	0.593	2.621				
Age	0.074	1.997	0.935	4.265					0.074	1.997	0.935	4.265				
Grade stage	0.049	2.11	1.003	4.436	0.112	1.832	0.868	3.866	0.049	2.11	1.003	4.436	0.003	2.612	1.393	4.897
TNM stage	0.01	2.678	1.266	5.664	0.01	2.672	1.262	5.655	0.01	2.678	1.266	5.664	0.023	2.04	1.104	3.768
T stage	0.621	1.221	0.552	2.7					0.621	1.221	0.552	2.7				
N stage	0.01	2.678	1.266	5.664	0.01	2.672	1.262	5.655	0.01	2.678	1.266	5.664	0.191	2	0.756	4.078

Legends: High METTL3 protein expression in the cytosol was significantly associated with poor prognosis in breast cancer patients

**Fig. 3:**
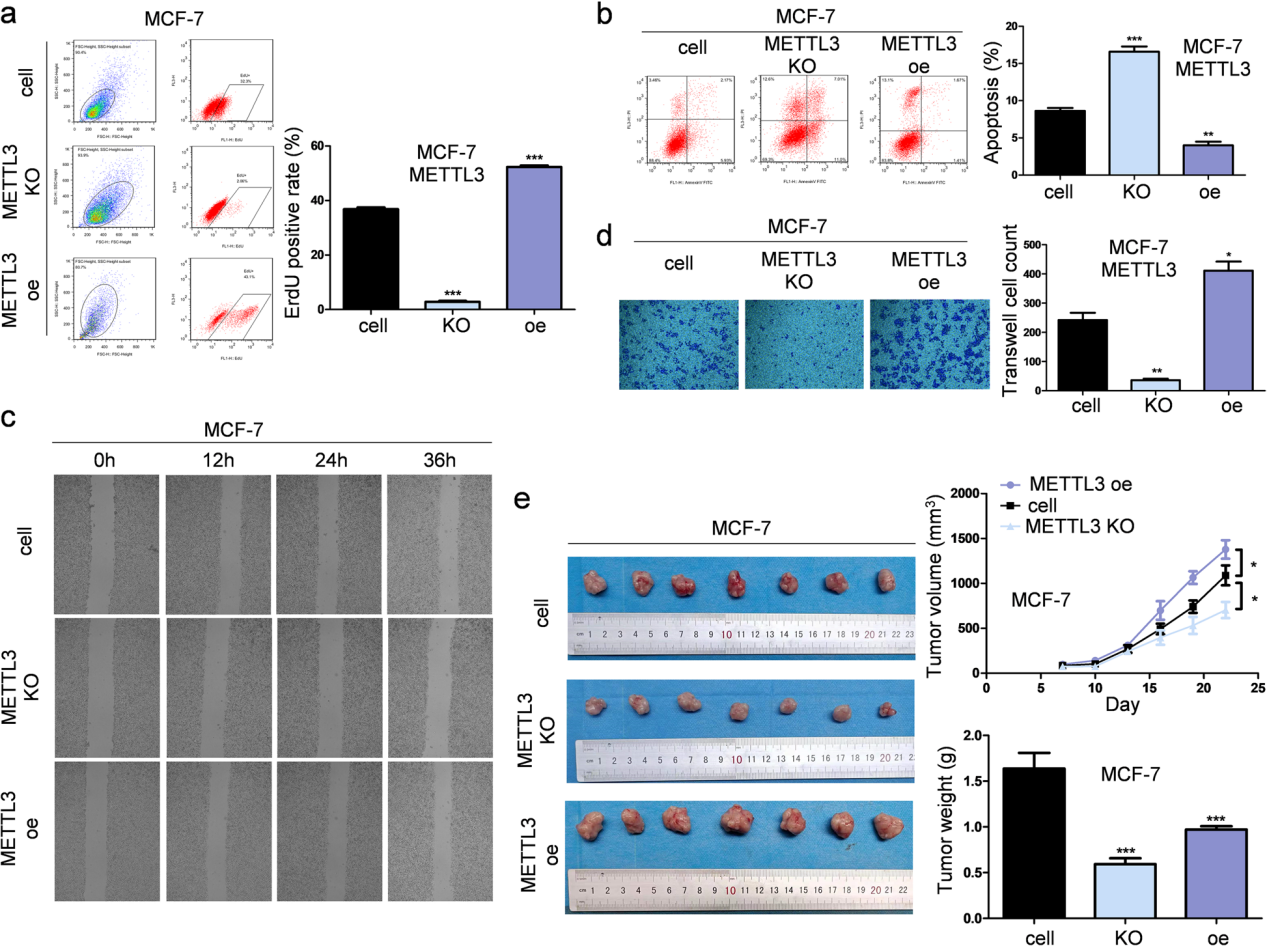
METTL3 promotes tumorigenesis of breast cancer cell MCF-7 both in vitro and in vivo. **a** The proliferation of MCF-7 cells was suppressed when the expression of METTL3 was altered in cells. The cell number was determined with EdU cellular proliferation assay with flow detection, *n* = 3, *** *p* < 0.001. **b** An apoptosis assay was performed after knocking out or overexpressing METTL3 in MCF-7 cells, and the apoptotic cell number was counted with a fluorescein assay, *n* = 3, ** *p* < 0.01, *** *p* < 0.001. **c** Blocking METTL3 inhibited the migration of MCF-7 cells, as detected by wound healing tests at 0 h, 12 h, 24 h and 36 h. **d** The invasion ability of MCF-7 cells, as revealed by the trans-well assay, was significantly suppressed by knocking out METTL3, *n* = 3, * *p* < 0.05, ** *p* < 0.01. **e** The tumor growth of MCF-7 cells in nude mice was delayed by METTL3 knockout, *n* = 7, * *p* < 0.05, *** *p* < 0.001

These data indicate that METTL3 promotes tumorigenesis of invasive ductal carcinoma of the breast. Therefore, we examined the role of METTL3 in breast tumorigenesis by knocking out or overexpressing METTL3 in MCF-7 and T47D cells (Supplementary Fig. 2c-d). Tumor cell proliferation and survival were significantly suppressed after METTL3 deletion (Fig. 3a-b and Supplementary Fig. 3a-b). Trans-well assays showed that METTL3 deletion inhibited the migration and invasion of MCF-7 and T47D cells (Fig. 3c-d and Supplementary Fig. 3c-d). Knockout of METTL3 in MCF-7 and T47D cells inhibited the growth of tumors formed by the corresponding cells in immunodeficient mice, whereas overexpression of METTL3 significantly promoted the growth of transplanted tumors (Fig. 3e and Supplementary Fig. 3e). Based on these data, we confirmed the important role of METTL3 in breast cancer tumorigenesis.

### METTL3 affects breast cancer cell metabolism by regulating m6A modification of LATS1 mRNA

**Fig. 4:**
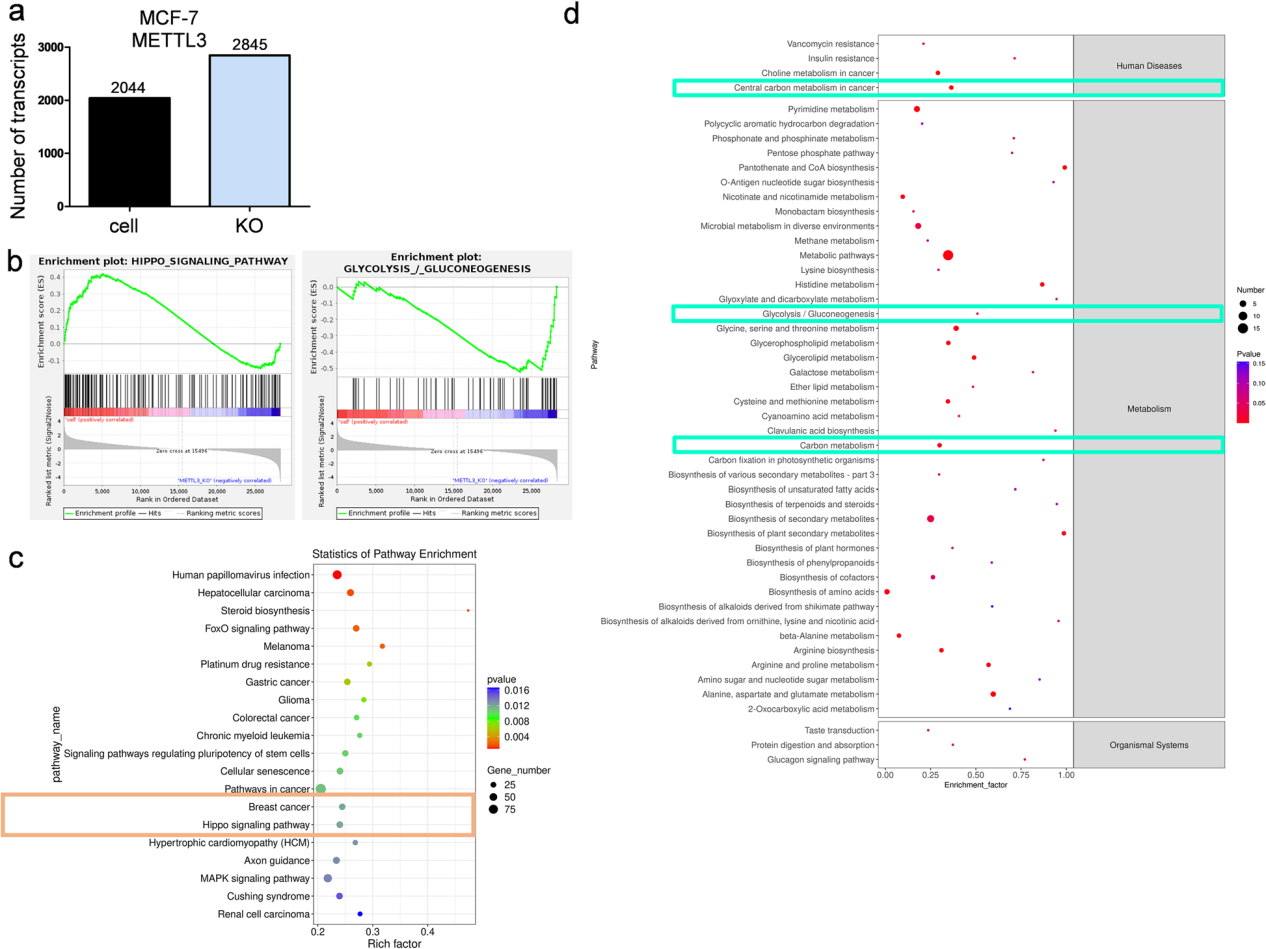
METTL3 affects the Hippo pathway and glycolysis in breast cancer cells. **a** Knocking out METTL3 remarkably affected transcriptome expression in MCF-7 cells. **b** KEGG enrichment analysis indicated that the Hippo pathway was upregulated, whereas glycolysis progression was downregulated by MTEEL3 knockout. **c** RNA-seq results showed that multiple differentially expressed genes involved in different pathways were significantly related to METTL3, *n* = 3. **d** KEGG enrichment of differential metabolites from metabolomic-seq analysis demonstrated that metabolites of glycolysis progression could be largely affected by METTL3, *n* = 6

RNA-seq was used to determine the influence of METTL3 on transcriptome expression in the MCF-7-cells. METTL3 expression markedly increased 2845 transcripts and reduced the expression of 2044 transcripts (Fig. 4a). KEGG analysis revealed that the Hippo pathway and glycolysis were significantly associated with METTL3 expression (Fig. 4b and Supplementary Table 4). Additionally, pathway enrichment analysis of the differentially expressed genes indicated that METTL3 was significantly related to breast cancer and the Hippo pathway (Fig. 4c). Since RNA-seq revealed that METTL3 might be related to glycolysis in breast tumor cells, we conducted metabolomic-seq analysis in METTL3 KO MCF-7 breast cancer cells. Differential metabolite KEGG analysis further demonstrated that the expression of METTL3 is involved in central carbon metabolism in cancer, especially in glycolysis and gluconeogenesis (Fig. 4d). Multiple metabolites involved in central carbon metabolism in cancer and glycolysis/gluconeogenesis were identified (Supplementary Fig. 4a-b and Supplementary Table 5).

**Fig. 5:**
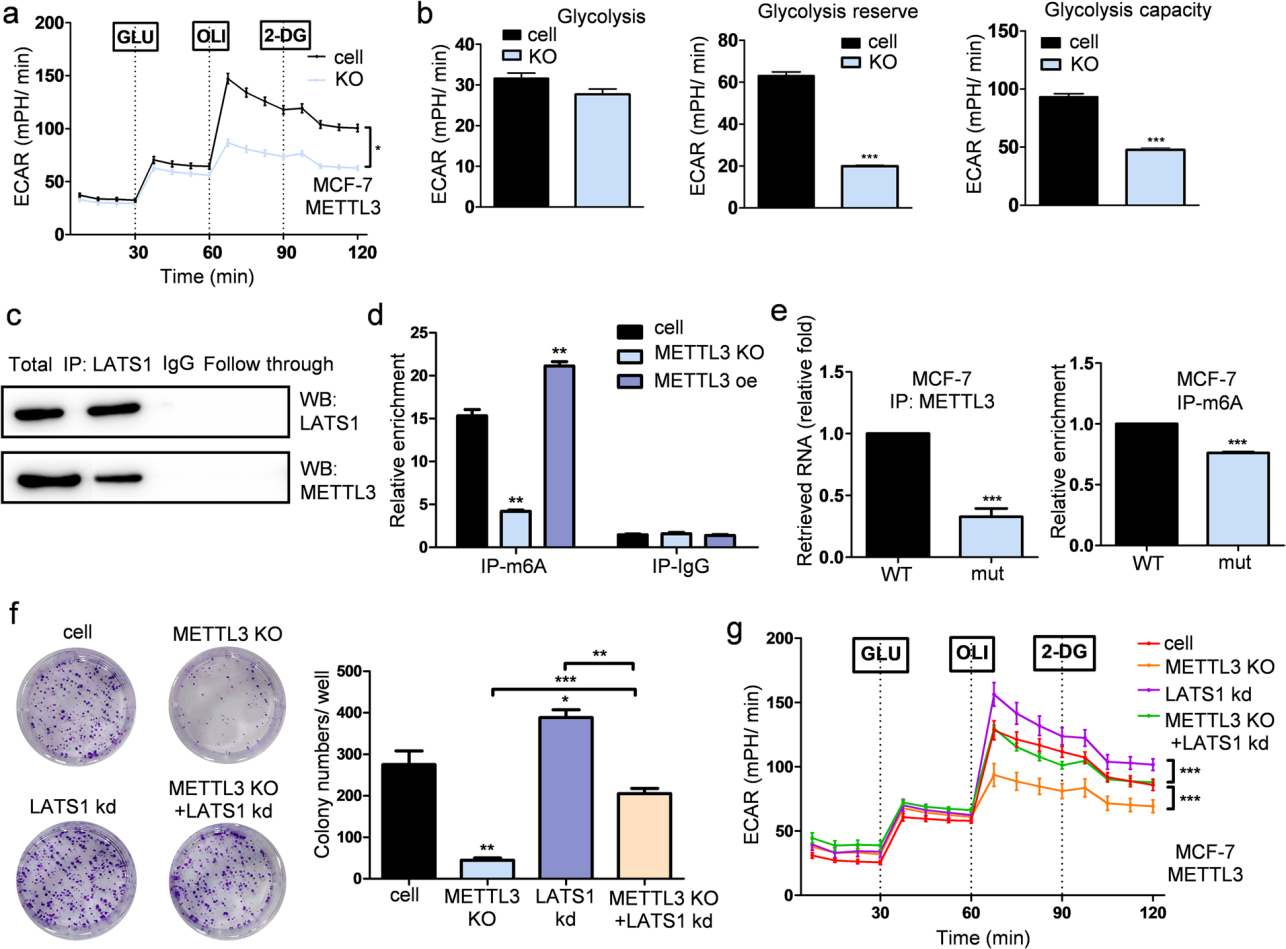
METTL3 promotes glycolysis and the colony-forming ability of breast cancer cells. **a** The ECAR in the METTL3 KO group and control group of MCF-7 cells in response to glucose, oligomycin and 2-DG, * *p* < 0.05. **b** The levels of glycolysis, glycolysis reserve and glycolysis capacity in MCF-7 cells compared with the control cells after deleting the expression of METTL3, *n* = 3, *** *p* < 0.001. **c** RNA-pull down assay showing the interaction between METTL3 and LATS1 mRNA. **d** The m6A level of LATS1 mRNA when the expression of METTL3 was altered in MCF-7 cells, as detected by MeRIP-qPCR, *n* = 3, ** *p* < 0.01. **e** The m6A level and METTL3 binding level of LATS1 mRNA when the m6A methylation sites on LATS1 mRNA were mutated in MCF-7 cells detected by MeRIP-qPCR, *n* = 3, *** *p* < 0.001. (**f**) Rescue of the expression of LATS1 by knocking out the expression of METTL3 and siRNA of LATS1 remarkably affected the proliferation of MCF-7 cells, *n* = 3, ** *p* < 0.01, *** *p* < 0.001. **g** Rescue of the expression of LATS1 by METTL3 significantly restored glycolysis in MCF-7 cells, *** *p* < 0.001

To understand the mechanism by which METTL3 promotes glycolysis in breast cancer, we evaluated the effect of METTL3 on glycolysis in MCF-7 and T47D cells. Notably, we found that deletion of METTL3 decreased glycolytic activity in breast cancer cell lines (Fig. 5a-b and Supplementary Fig. 5a-b). Since it is well known that the Hippo pathway is involved in cancer development, we predicted that METTL3 could mediate m6A modification of LATS1 and further affect the Hippo pathway. Using an RNA pull-down assay with western blotting, we confirmed the interaction between LATS1 mRNA and METTL3 (Fig. 5c). In addition, the protein levels of METTL3 were inversely correlated with the mRNA levels of LATS1 in breast cancer cells (Supplementary Fig. 5c). MeRIP-qPCR results indicated that the presence of METTL3 directly increased the m6A level of LATS1 mRNA (Fig. 5d and Supplementary Fig. 5d). To identify the region of LATS1 mRNA that interacted with METTL3, we examined the interaction between METTL3 and LATS1 mRNA. The mutation sites were based on the results of MeRIP-seq and SRAMP prediction. The results of MeRIP-qPCR further confirmed that the regulation of METTL3 mediates m6A methylation of LATS1 mRNA (Fig. 5e and Supplementary Fig. 5e). To investigate the impact of m6A regulation on proliferation and glycolysis in breast cancer cells, a colony formation assay was performed, which revealed that the proliferation of breast cancer cells could be rescued by the inhibition of LATS1 after METTL3 knockout (Fig. 5f and Supplementary Fig. 5f). Moreover, the seahorse assay revealed that glycolysis was upregulated when LATS1 expression was suppressed after METTL3 expression was deleted in breast cancer cells (Fig. 5g and Supplementary Fig. 5g).

### M6A regulation of LATS1 mRNA was identified by ythdf2 in breast cancer cells

**Fig. 6:**
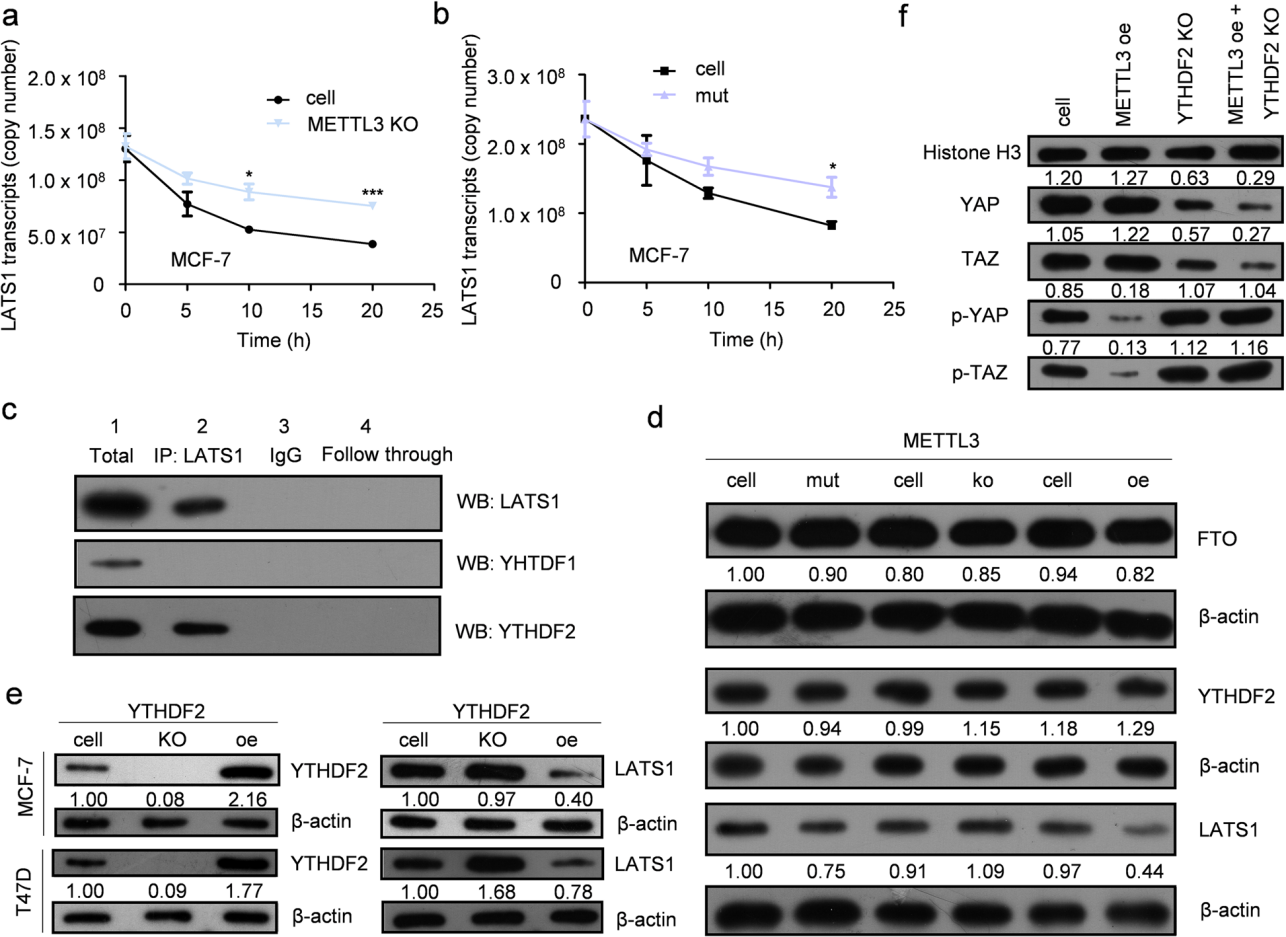
M6A regulation of LATS1 mRNA was identified by ythdf2 in breast cancer cells. **a** Deletion of METTL3 improved the stability of LATS1 mRNA, *n* = 3, * *p* < 0.05, *** *p* < 0.001. **b** Conducting the m6A methylation sites mutation on LATS1 mRNA helped enhance the stability of LATS1 mRNA, *n* = 3, * *p* < 0.05. **c** RNA-pull down assay showed the interaction between YTHDF2 and LATS1 mRNA. **d** Altering the expression of METTL3 and mutating the LATS1 mRNA binding site of METTL3 affected only the protein level of LATS1. **e** YTHDF2 negatively regulated the protein level of LATS1 in breast cancer cells. **f** Rescue of the expression of LATS1 by altering the protein level of METTL3 together with YTHDF2 affected the phosphorylation level of YAP/TAZ in the nucleus

LATS1 acts as a tumor suppressor in various human cancers [20]. Our previous study showed that an E3 ligase of LATS1 could destabilize the protein level of LATS1 by inducing its ubiquitination [12]. Since a high m6A modification level of LATS1 mRNA was found by MeRIP-seq, we aimed to determine which m6A readers directly recognize m6A modification sites and regulate the expression of LATS1 in breast cancer cells. A stability assay of LATS1 mRNA showed that METTL3 deletion promoted the stability of mRNA (Fig. 6a), which was also enhanced by mutating the m6A sites in LATS1 mRNA (Fig. 6b). Using an RNA pull-down assay and western blotting, we found that YTHDF2 was an m6A reader of LATS1 mRNA (Fig. 6c). It has been reported that YTHDF2 promoted the degradation of its target gene mRNA by recognizing m6A modifications [21]. We found that the expression of METTL3 had no influence on the expression of the m6A eraser FTO or the m6A reader YTHDF2 (Fig. 6d). However, the protein level of LATS1 was inversely correlated with the expression of YTHDF2 in breast cancer cells (Fig. 6e).

**Fig. 7:**
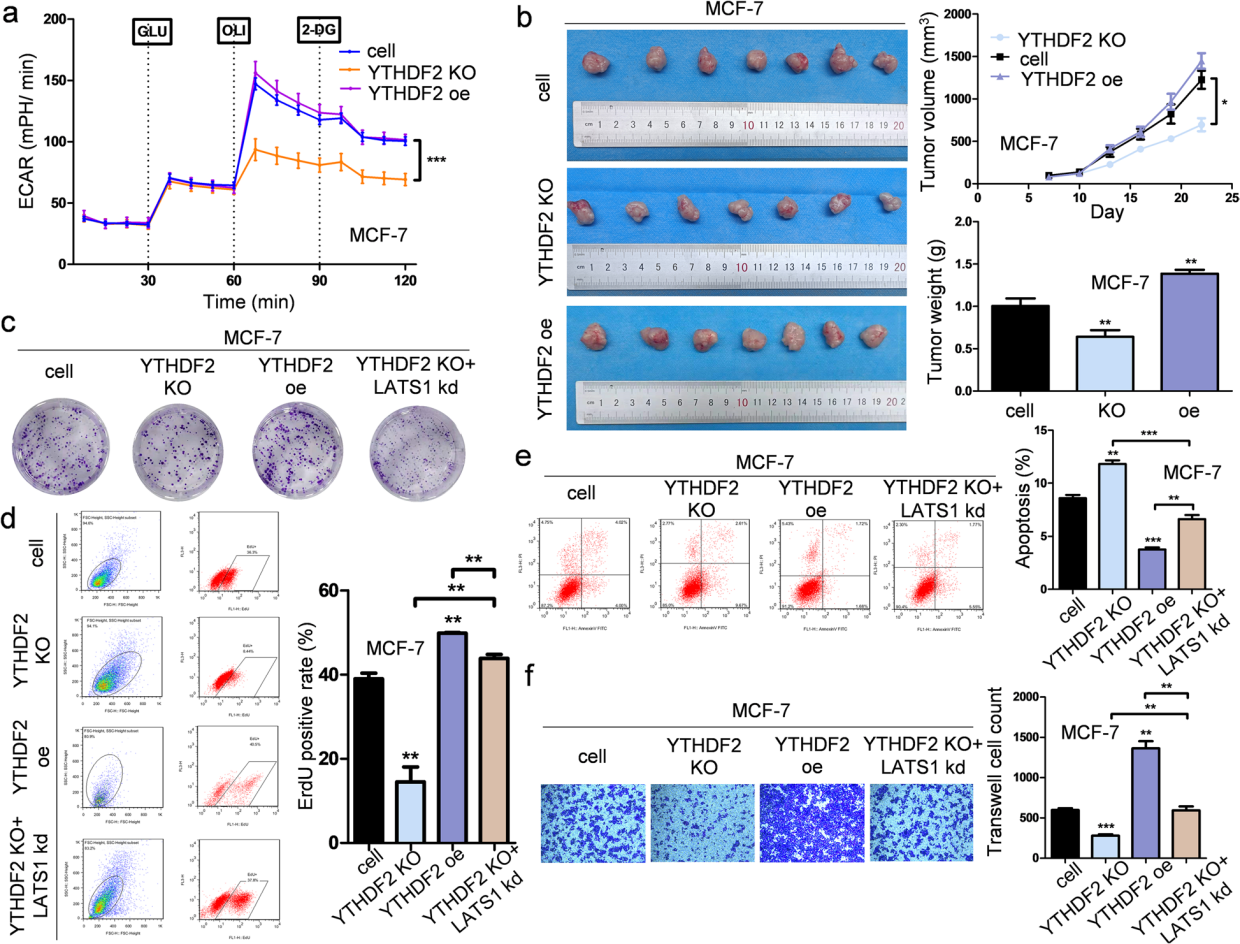
Rescuing the protein level of LATS1 by altering the expression of YTHDF2 in breast cancer cells could help suppress tumorigenesis. **a** YTHDF2 deletion remarkably suppressed glycolysis progression in MCF-7 cells, *** *p* < 0.001. **b** Knocking out the expression of YTHDF2 significantly inhibited tumor growth in vivo, *n* = 7, * *p* < 0.05, ** *p* < 0.01. **c** The colony formation ability of MCF-7 cells was suppressed by knocking out the expression of YTHDF2 and reversed by inhibiting the expression of LATS1 at the same time. **d **YTHDF2 overexpression led to the rapid proliferation of MCF-7 cells and such stimulation could be rescued by LATS1 knockdown, *n* = 3, ** *p* < 0.01. **e** YTHDF2 deletion raised the apoptosis level of MCF-7 cells, while LATS1 knockdown rescued this stimulation, *n* = 3, ** *p* < 0.01, *** *p* < 0.001. **f** The suppressed invasion ability of MCF-7 cells affected by YTHDF2 knockout could be rescued by LATS1 knockdown, *n* = 3, ** *p* < 0.01, *** *p* < 0.001

To determine the impact of YTHDF2 in breast cancer cells and understand its role in the m6A methylation of LATS1 mRNA in tumorigenesis and glycolysis, we altered the expression of YTHDF2 in breast cancer cells (Fig. 6e). We found that m6A methylation of LATS1 downregulated LATS1 expression in breast cancer cells (Figs. 5d, 6e and Supplementary Fig. 5c-d) and activated YAP/TAZ by inhibiting its phosphorylation (Fig. 6f). High levels of YAP/TAZ were found in the nucleus of breast cancer cells after inducing the expression of METTL3, while deleting the expression of YTHDF2 corrected such deviated expression in the nuclears (Fig. 6f). The seahorse assay showed that YTHDF2 had a positive effect on glycolysis in breast cancer cells (Fig. 7a and Supplementary Fig. 6a). The expression of YTHDF2 in breast cancer cells promoted tumorigenesis both in vitro and in vivo (Fig. 7b-c and Supplementary Fig. 6b-c). Since YTHDF2 destabilized LATS1 mRNA by reading the m6A methylation in LATS1, we predicted that deleting the expression of YTHDF2 could promote the tumor-suppressive effects of LATS1. Consistent with this hypothesis, tumor proliferation, survival, and invasion were inhibited by YTHDF2 depletion, and this tumor suppressive effect was rescued by inhibiting the expression of LATS1 (Fig. 7c-f and Supplementary Fig. 6c-f).

## Discussion

The Hippo signaling pathway plays an important role in the development and progression of breast cancer. It mainly controls organ size by regulating cell proliferation and apoptosis. Once the Hippo signaling pathway is inhibited, cells overcome contact inhibition and enter a state of uncontrolled proliferation. As the core effector molecule of the Hippo signaling pathway, LATS1 is key to tumor therapy research, and the regulatory element targeting LATS1 has also become a hot topic in tumor research. Among multiple transcription factors, RNA methylation is a new and important research direction in epigenetics. The m6A methylation modification of mRNA occurs via the participation of methyltransferase by dynamic control, and protein identification is determined by methylation, which affects protein localization, translation, degradation, and expression, ultimately contributing to the development of tumors. Currently, studies on m6A methylation in tumors have suggested that m6A modification determines the fate of RNA, but its mechanism has not been clarified. Therefore, we focused on the mechanism and impact of m6A modification on LATS1 mRNA.

We demonstrated that Mettl3-mediated m6A methylation of LATS1 mRNA is recognized by YTHDF2, which reduces LATS1 expression and inactivates the Hippo pathway in breast cancer. We found that the deletion of METTL3 significantly suppressed proliferation, migration, and invasion in MCF-7 and T47D breast cancer cells, as well as tumor progression in vivo. Combined analysis of the multiple sequencing results revealed that METTL3 participates in the inhibition of LATS1 and affects tumorigenesis and metabolism in breast cancer cells. Considering the important role of LATS1 in tumor suppression, we investigated the exact mechanism of METTL3-mediated m6A modification of LATS1. We found that METTL3 mediates the methylation modification of m6A sites of LATS1 mRNA. Such m6A sites of LATS1 mRNA can be recognized by YTHDF2, which promotes the degradation of LATS1 mRNA and eventually tumorigenesis and glycolysis in breast cancer cells. Our findings suggest that METTL3 exerts oncogenic activity by inhibiting the expression of LATS1 through m6A modification. Therefore, our discovery of m6A modification of LATS1 mRNA poses a new regulatory mechanism for the expression of LATS1.

## Conclusion

We discovered a negative regulatory mechanism of LATS1 mRNA, in which METTL3 mediated m6A modification of certain sites in LATS1 mRNA, and this modification was recognized by YTHDF2. Thus, the expression of LATS1 and the phosphorylation of proteins in the Hippo pathway in breast cancer were inhibited, ultimately leading to glycolysis and tumor development. 

## Supplementary Information

**Additional file 1:** **Supplementary Figure 1. **m6A modification levels in the Hippo pathway and metabolism in breast cancer. (a) Venn diagram (left panel) of common (the intersection area) and unique peaks (non-intersection region) of another pair of breast cancer tissue compared with adjacent normal tissue, |log2(fold change)|>1 and q value<0.05. The gene region (right panel) annotated by difference Peaks of breast cancer tissue compared with adjacent normal tissue, |log2(fold change)|>1 and q value<0.05. (b) Differential m6A and differential expression combined analysis diagram of another pair of breast cancer tissue compared with adjacent normal tissue. (c) GO analysis results of differential methylated genes. Differential methylated genes were obtained by TCGA clinical data of breast cancer and compared with MeRIP- seq data. (d) Enrichment analysis result of biological progress (BP) of differential methylated genes. (e) Location map of LATS1 in differential methylated KEGG pathway of Hippo. (f) Volcano map of differentially expressed genes based on transcriptome sequencing of two pairs of breast cancer tissue compared with adjacent normal tissue. (g) Differentially expressed genes of Hippo pathway in transcriptome sequencing of breast cancer tissues compared with adjacent normal tissues, *n*=2. (h) M6A peaks on LATS1 mRNA originated from MeRIP-seq. M6A peaks were visualized by IGV. The red peaks show the results of MeRIP and the blue peaks represent the input. **Supplementary Figure 2**. Expression of m6A proteins in breast cancer tissue and cells. (a) The correlation between METTL3 and the expression level of ER/ PR/ HER2 in breast adenocarcinomas (GSE70951, 195 breast adenocarcinomas and matched adjacent normal breast tissue samples). (b) The correlation between METTL3 protein expression and the prognosis of Luminal A (upper panel) and Luminal B (lower panel) breast adenocarcinomas. (c) Alter the mRNA expression level of METTL3 in MCF-7 and T47D cells, ** *p*<0.01. (d) Alter the protein level of METTL3 in MCF-7 and T47D cells. **Supplementary Figure 3. **METTL3 promotes tumorigenesis of T47D cells. (a) The proliferation of T47D was determined with EdU cellular proliferation assay with flow detection after altering the expression of METTL3, *n*=3, * *p* <0.05, ** *p* <0.01. (b) The apoptotic cell number was counted with fluorescein assay when knocking out or overexpressing METTL3 in T47D, *n*=3, *** *p* <0.001. (c) Migration ability of T47D was suppressed when knocking out the expression of METTL3 detected at 0h, 12h, 24h and 36h. (d) Cell invasion ability was inhibited after blocking the expression of METTL3 in cells detected by trans-well assay, *n*=3, ** *p* <0.01. (e) The tumorigenesis of T47D in nude mice was significantly suppressed when knocking out the expression of METTL3, *n*=7, * *p* <0.05, *** *p* <0.001. **Supplementary Figure 4. **Large amounts of differently expressed metabolites were involved in the deletion of METTL3 in MCF-7. (a) Heat map of differentially expressed metabolites when knocking out the expression of METTL3 in MCF-7, *n*=6. (b) KEGG enrichment results of differential metabolites affected by METTL3 deletion in MCF-7. **Supplementary Figure 5. **METTL3 promotes glycolysis and proliferation of T47D cells. (a) The ECAR change between METTL3 KO group and control group of T47D cells in response to glucose, oligomycin and 2-DG, *n*=4, ** *p* <0.01. (b) The level of glycolysis, glycolysis reserve and glycolysis capacity in T47D cells with METTL3 knock-out and control group, *n*=4, * *p* <0.05, *** *p* < 0.001. (c) The protein level (left panel) and mRNA level (right panel) of LATS1 after knocking out and overexpressing METTL3 in MCF-7 and T47D cells, *n*=3, * *p* <0.05, ** *p* < 0.01. (d) MeRIP-qPCR shown the m6A level of LATS1 mRNA after altered the expression of METTL3 in T47D, *n*=3, ** *p* <0.01. (e) MeRIP-qPCR revealed the m6A level and METTL3 binding level of LATS1 mRNA when muted the m6A methylation sites on LATS1 mRNA in T47D, *n*=3, ** *p* <0.01, *** *p* < 0.001. (f) Rescued the expression of LATS1 with knocking out the expression of METTL3 and siRNA of LATS1 remarkably affected the proliferation of T47D cells. (g) The glycolysis was regained by rescuing the expression of LATS1 by METTL3 in T47D cells, *n*=4, *** *p* < 0.001. **Supplementary Figure 6. **YTHDF2 helped promote the tumorigenesis of T47D breast cancer cells. (a) Knocking out YTHDF2 remarkably suppress the glycolysis progress in T47D cells, *n*=4, *** *p* <0.001. (b) Deleting the expression of YTHDF2 significantly inhibited the tumor growth in vivo, *n*=7, ** *p* <0.01, *** *p* <0.001. (c)The colony formation ability of T47D cells was suppressed by the deletion of YTHDF2 and reversed by downregulated the expression of LATS1 at the same time. (d) YTHDF2 knock out led to the slower proliferation of T47D cells and such inhibition could be rescued by LATS1 knockdown, *n*=3, ** *p* <0.01. (e) YTHDF2 deletion increased the apoptosis rate of T47D cells, *n*=3, ** *p* <0.01, *** *p* <0.001. (f) Trans-well assay revealed the invasion ability of T47D could be controlled by missing expression of YTHDF2, but also could be rescued by LATS1 siRNA, *n*=3, * *p* <0.05, ** *p* <0.01.

**Additional file 2:** **Supplementary Table 1.** Data of the included patients. Clinicopathological information on age, sex, clinical stage, neoadjuvant therapy, adjuvant therapy, ER, PR, HER2, Ki67, histological subtype and histological grade of each included patient. **Supplementary Table 2.** Differential m6A sites in two groups. Specific sites of 1850 up regulated and 1807 down regulated genes of breast cancer cells, *p*-values <0.05 were indicated. **Supplementary Table 3.** The relationship between METTL3 and patient clinicopathological features. The age of patients was significantly related to the expression of METTL3, *p*-values <0.05 were indicated. **Supplementary Table 4**. KEGG analysis of MCF-7 between METTL3 KO group and control group. KEGG pathways of differentially expressed genes that were altered significantly after METTL3 knockout, *p*-values <0.05 were indicated. **Supplementary Table 5.** Metabolites involved in METTL3 knockout in MCF-7. KEGG pathways of different metabolites that were altered significantly after knocking out METTL3 expression, *p*-values <0.05 were indicated.

## Data Availability

The datasets generated and analyzed during the current study are available from the corresponding author upon reasonable request.

## Abbreviations

IARC: International Agency for Research on Cancer
TNBC: Triple-negative breast cancer
m6A: N6-methyladenosine
LATS1/LATS2: Large tumor suppressor homolog 1/ large tumor suppressor homolog 2
YAP: Yes-associated protein
TAZ: Tafazzin
METTL3: Methyltransferase-like protein 3
YTHDF1: YTH domain-containing family protein 1
YTHDF2: YTH domain-containing family protein 2
YTHDF3: YTH domain-containing family protein 3
POS: Positive ion
NEG: Negative ion
RIP: RNA binding protein immunoprecipitation
MeRIP- qPCR: M6A-seq-IP-qPCR
KEGG: Kyoto Encyclopedia of Genes and Genomes
HMDB: Human Metabolome Database
PLS-DA: Partial least squares-discriminant analysis
IHC: Immunohistochemistry
GEO: Gene Expression Omnibus
TMA: Tissue microarrays
