# Supplementary figures and images for: The N6-methyladenosine METTL3 regulates tumorigenesis and glycolysis by mediating m6A methylation of the tumor suppressor LATS1 in breast cancer
Source: J Exp Clin Cancer Res. 2023 Jan 7;42:10. doi: 10.1186/s13046-022-02581-1 (PMC9824909; doi:10.1186/s13046-022-02581-1)

a

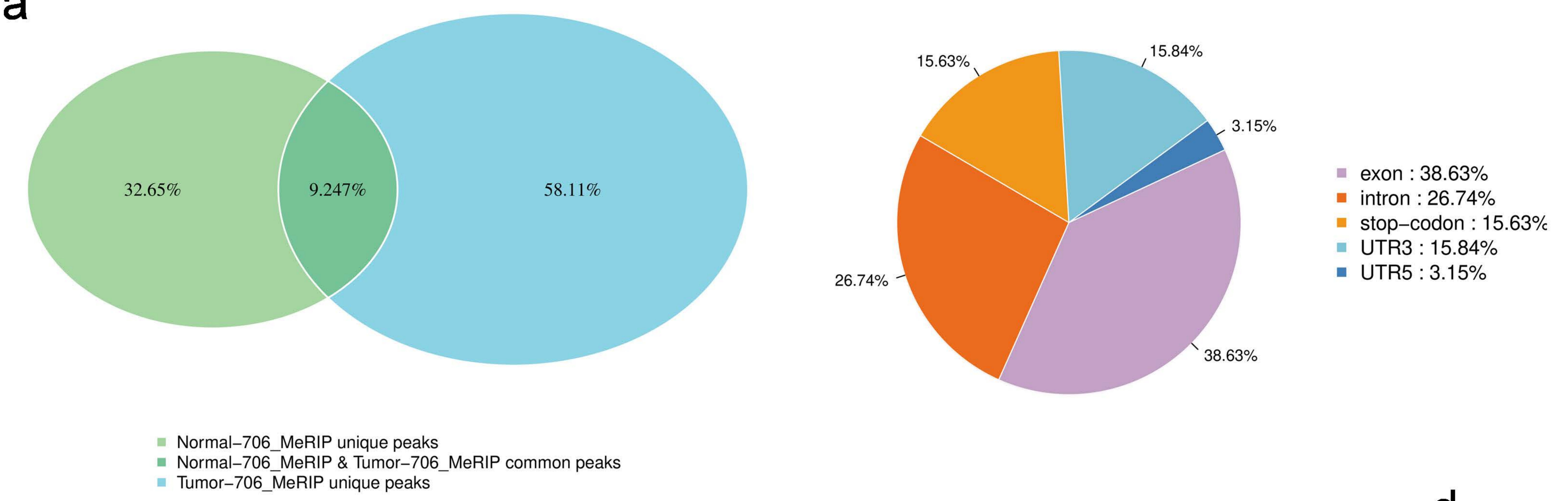

c

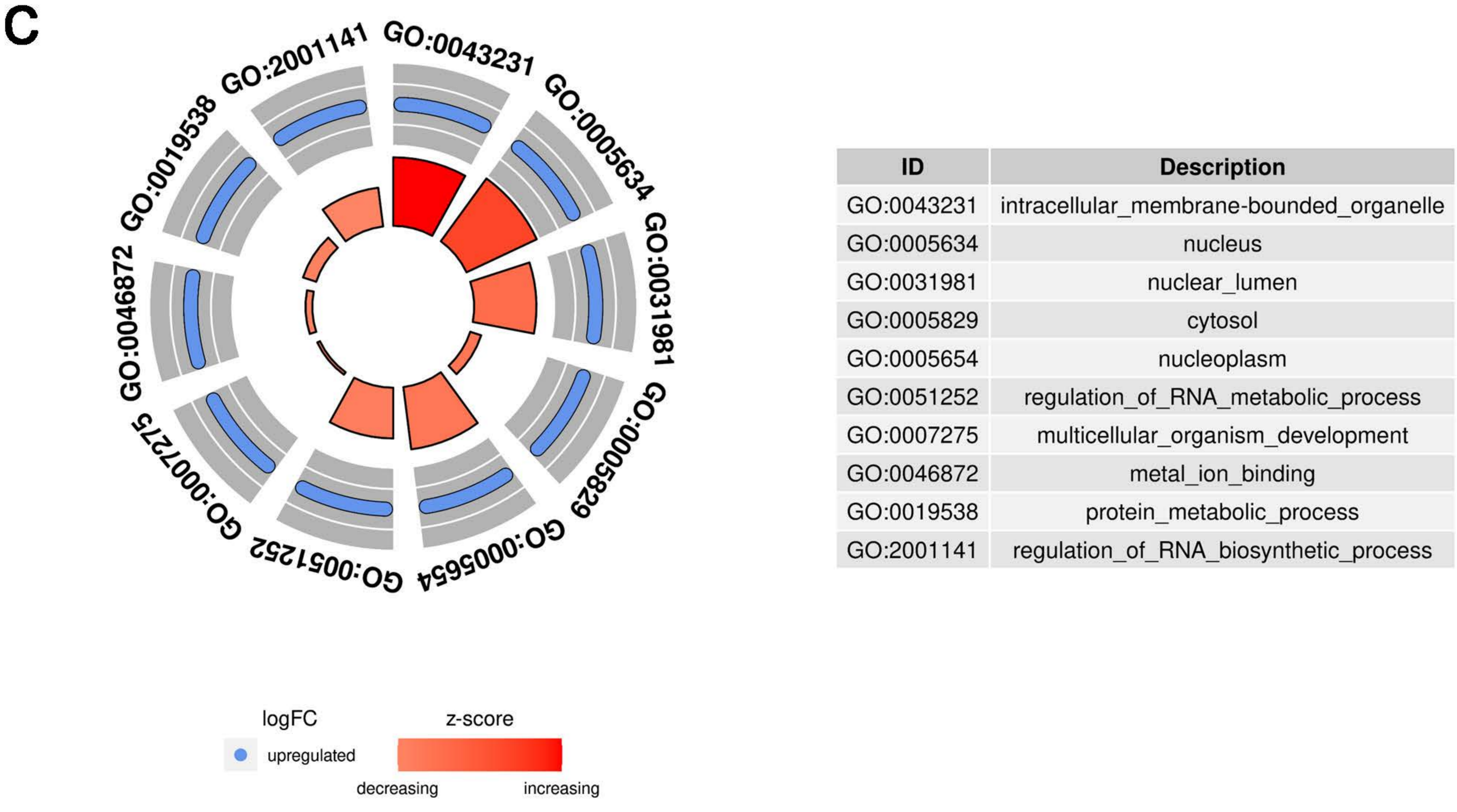

e

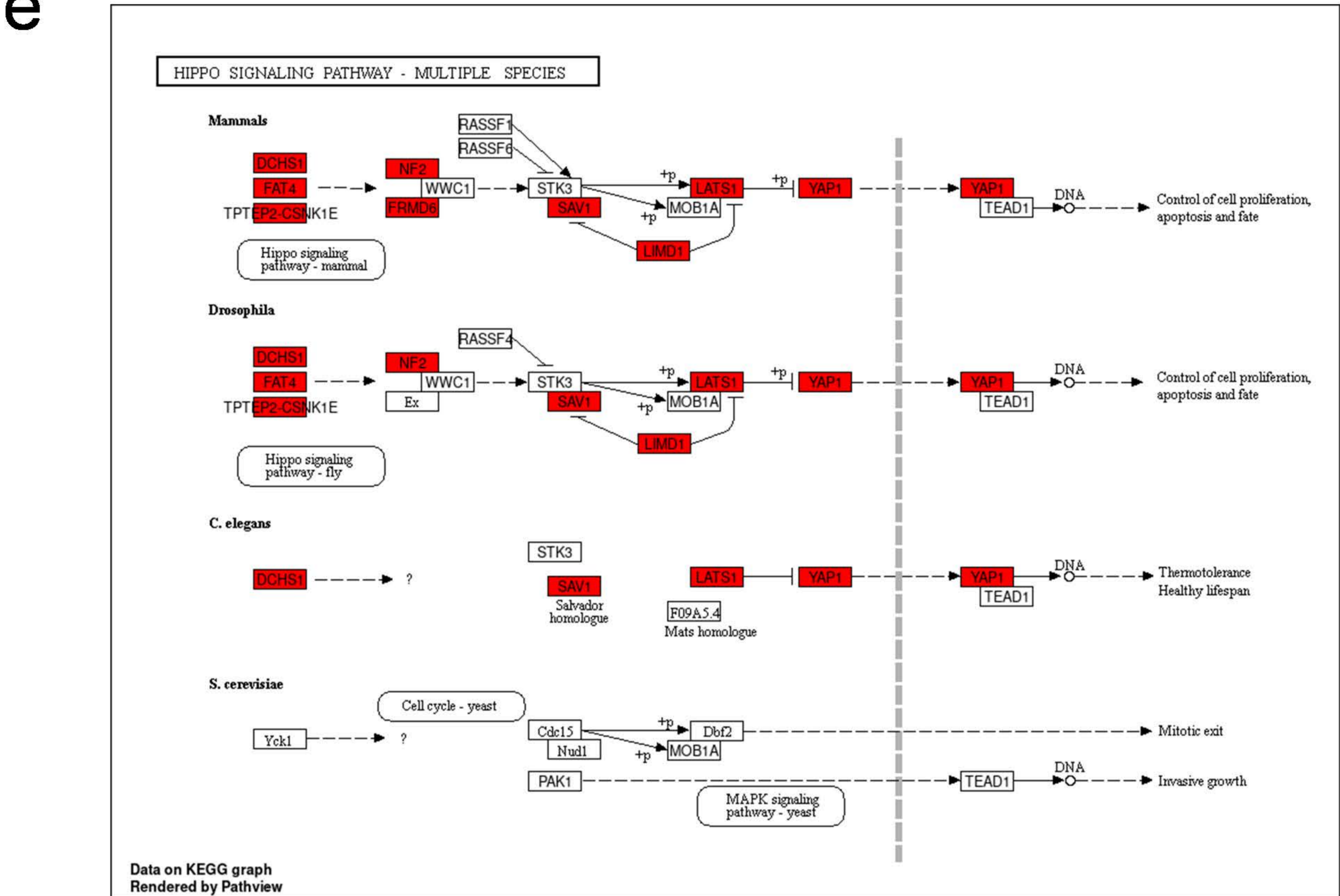

h

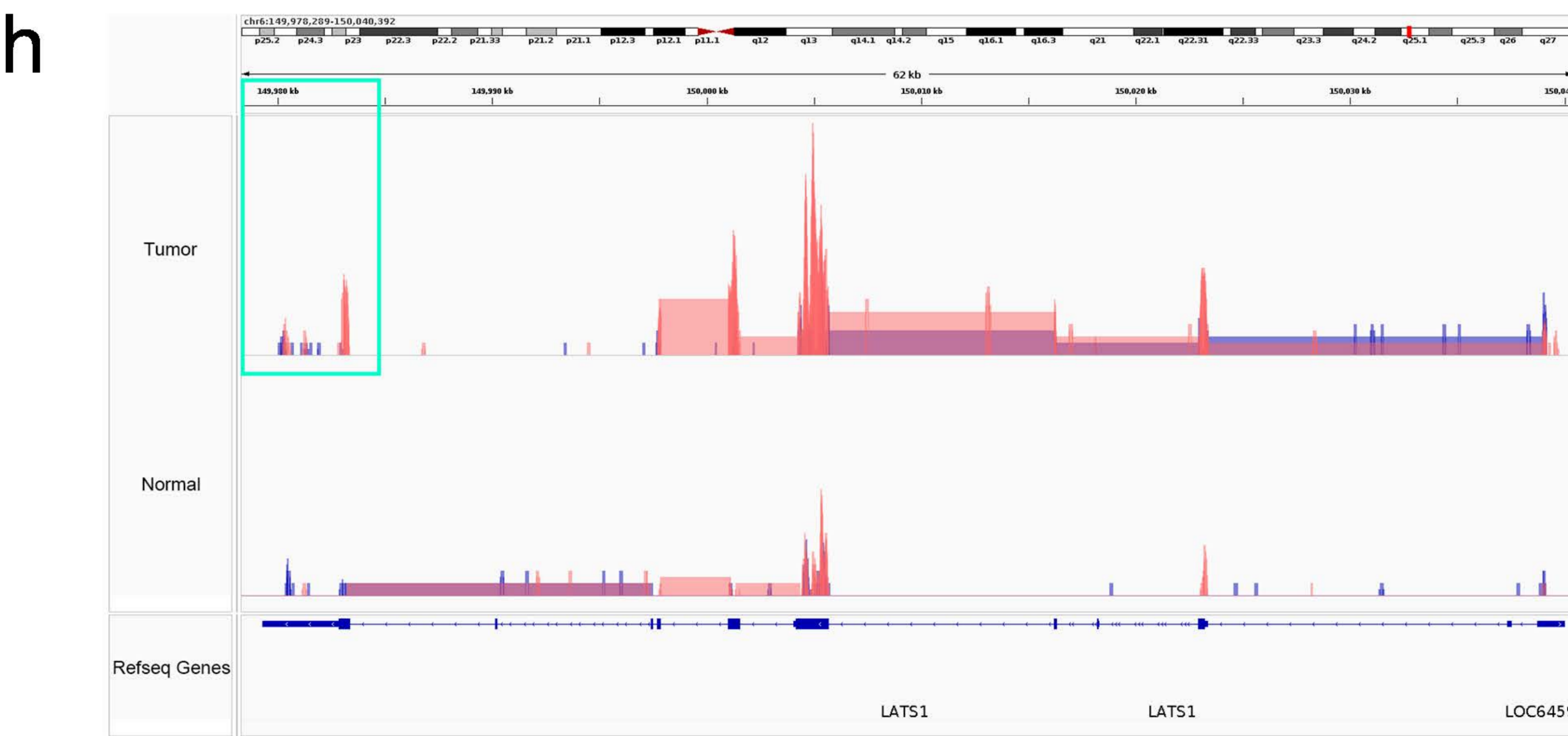

b

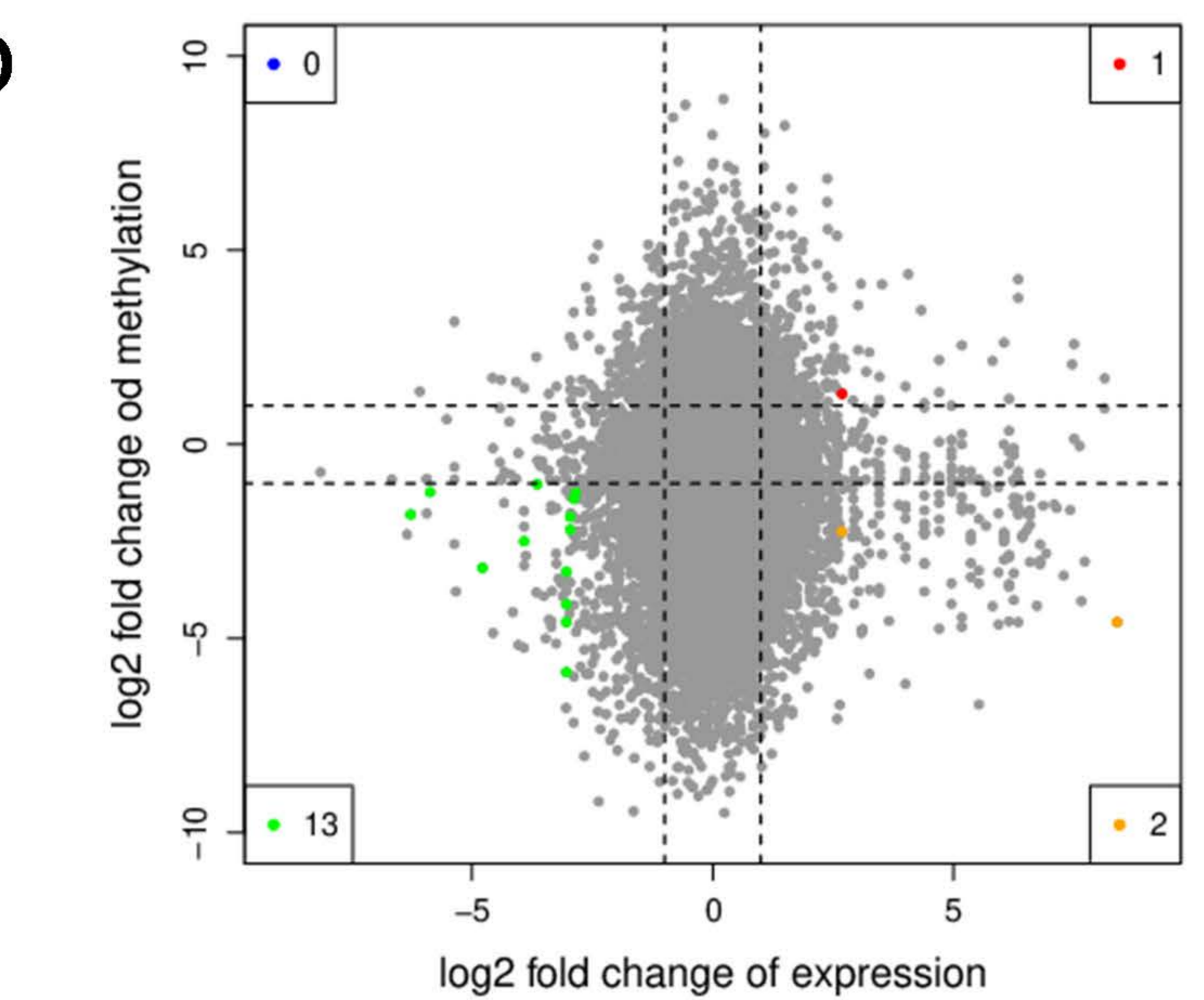

d

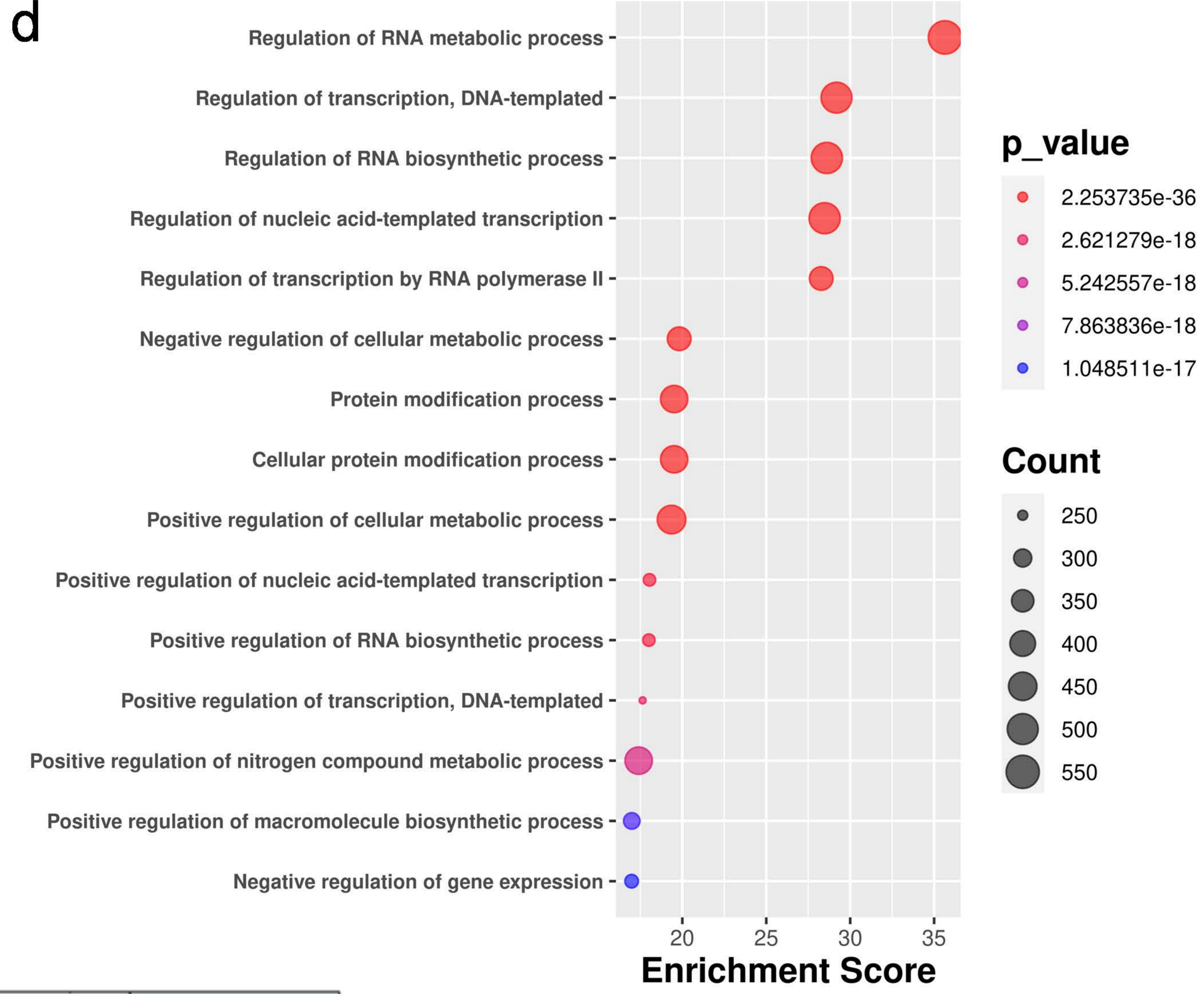

f

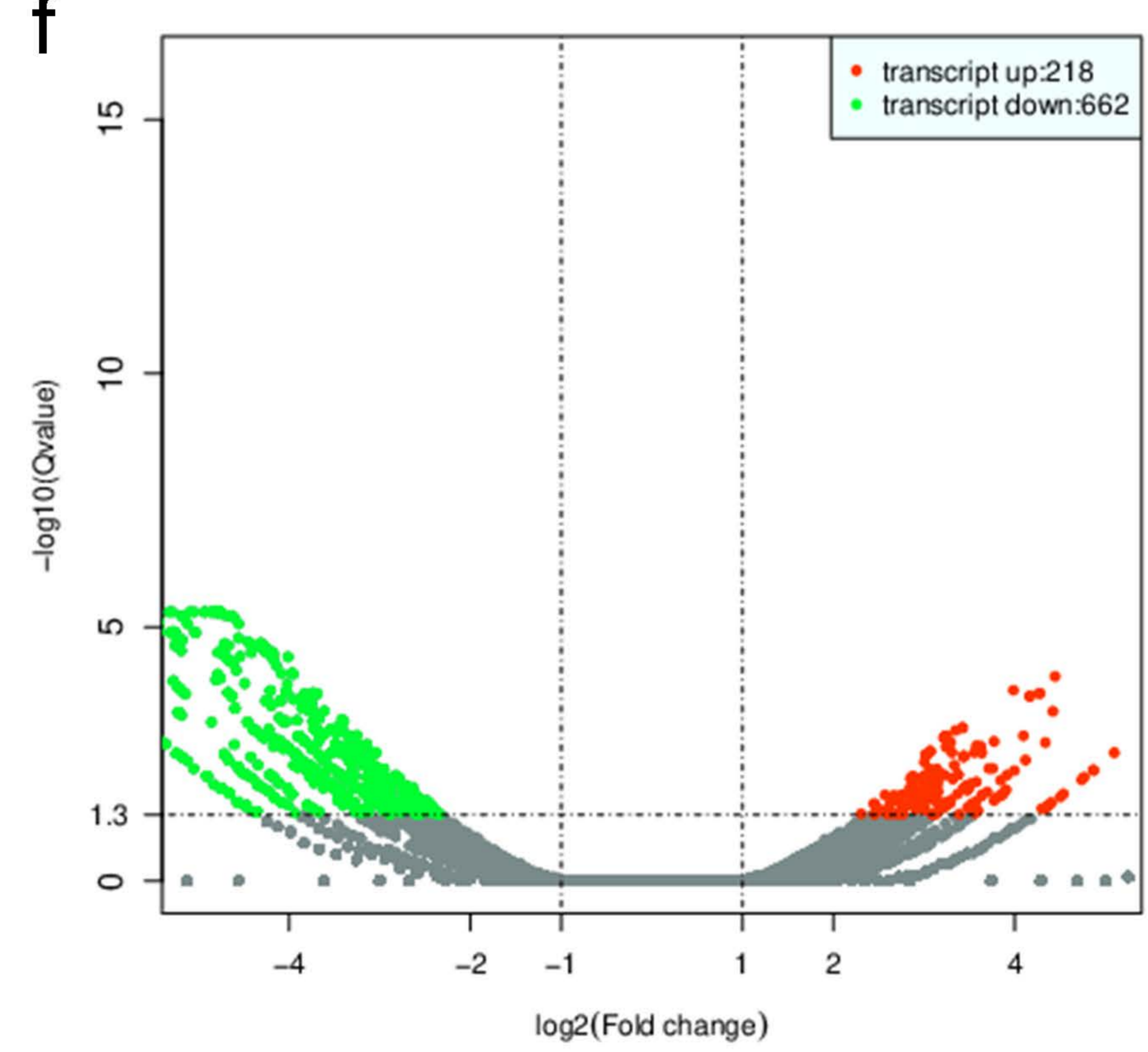

g

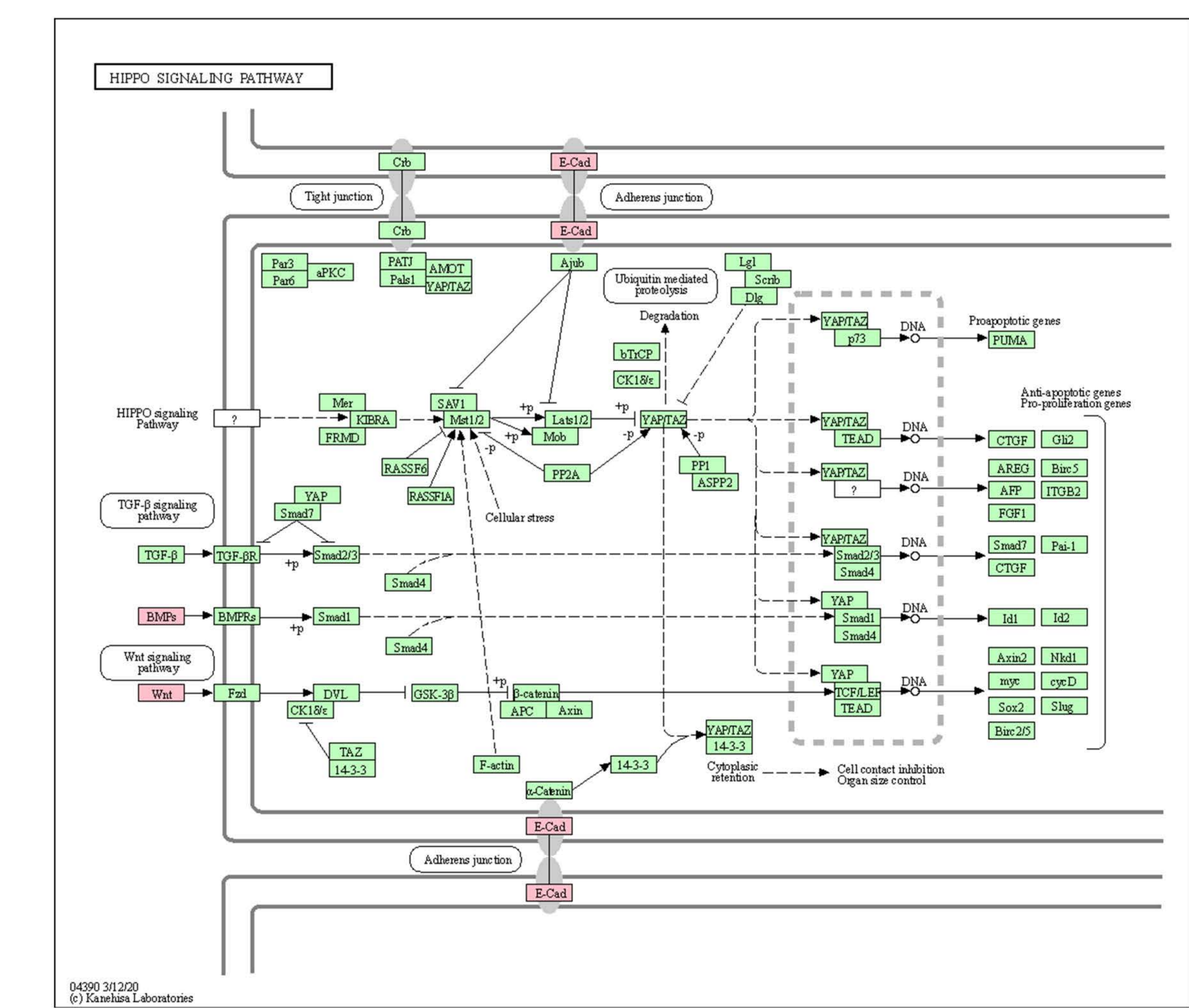

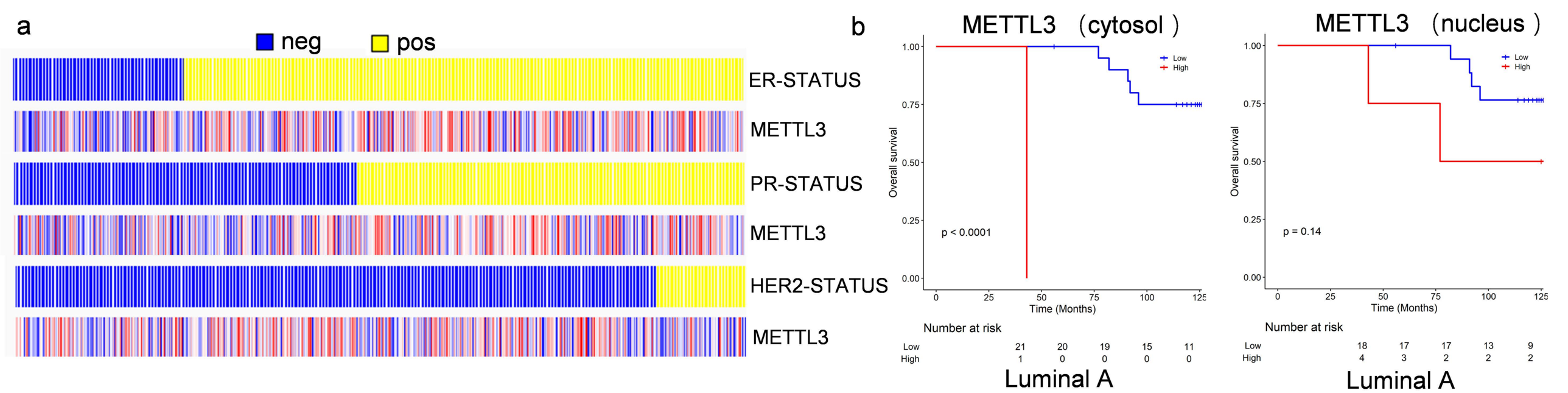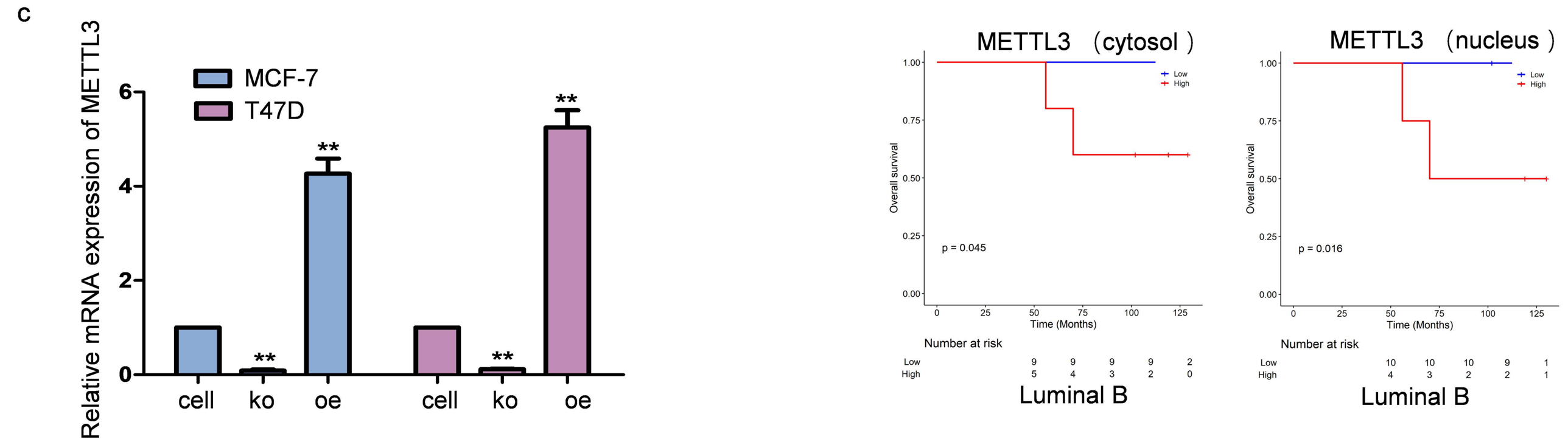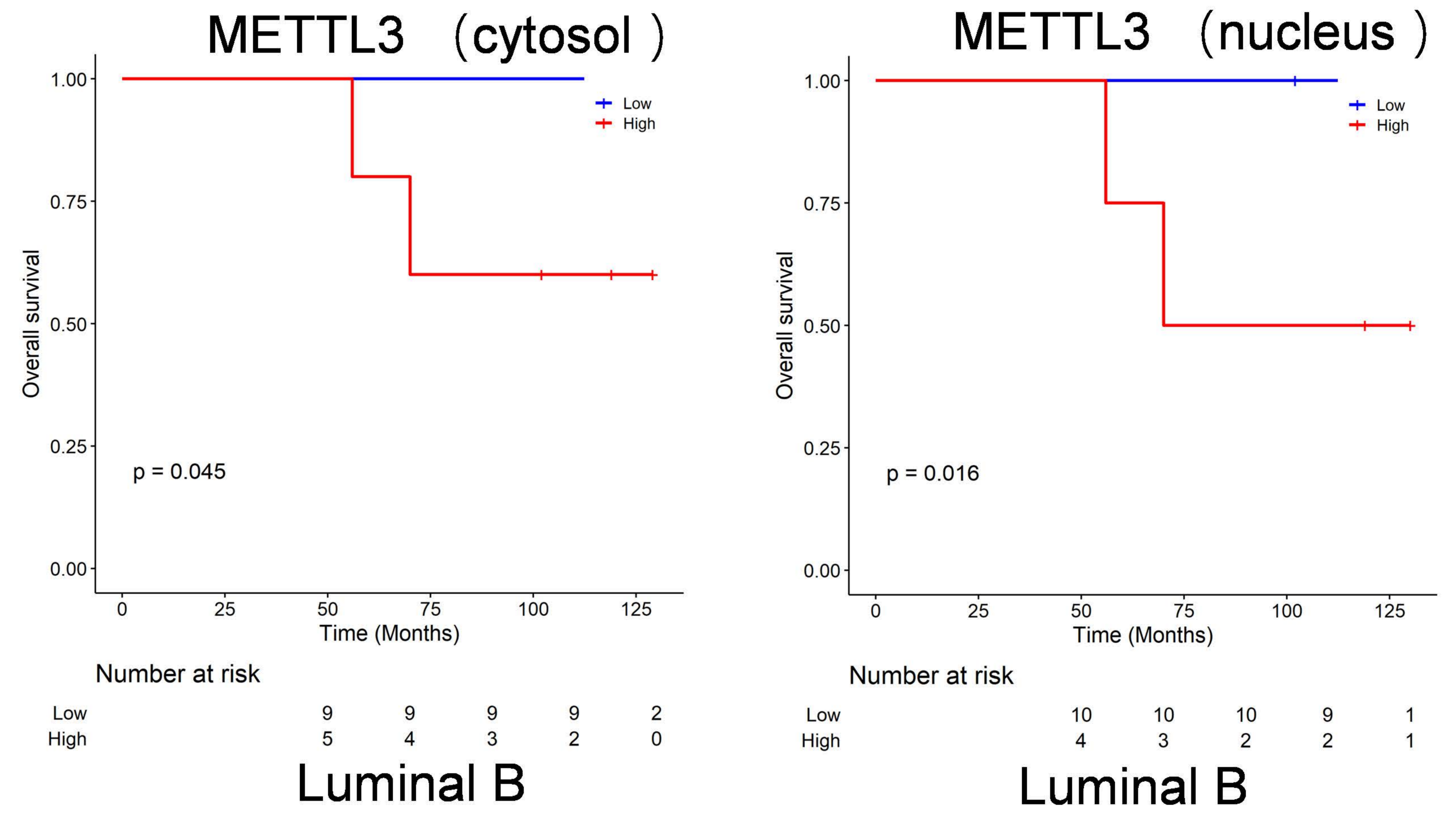

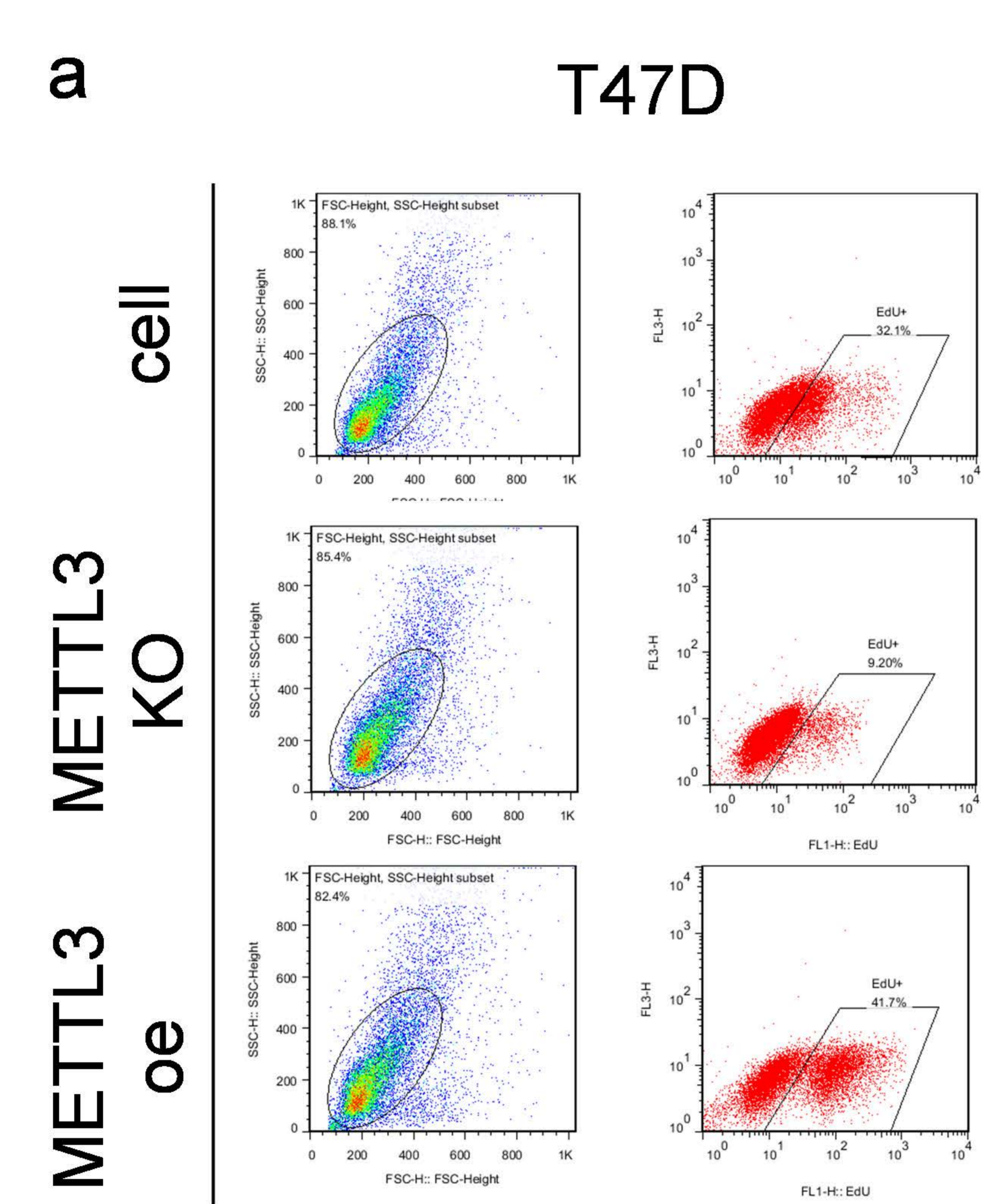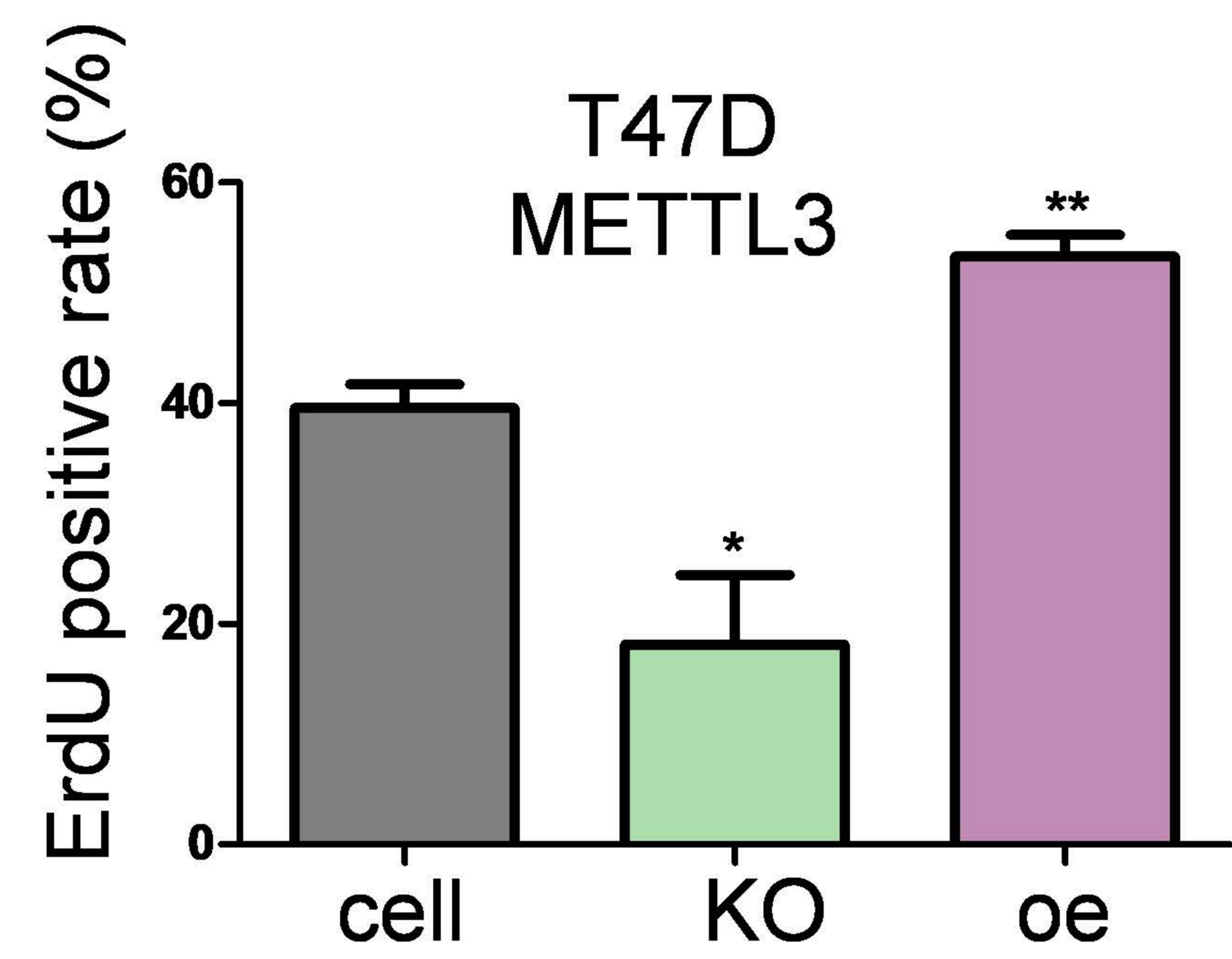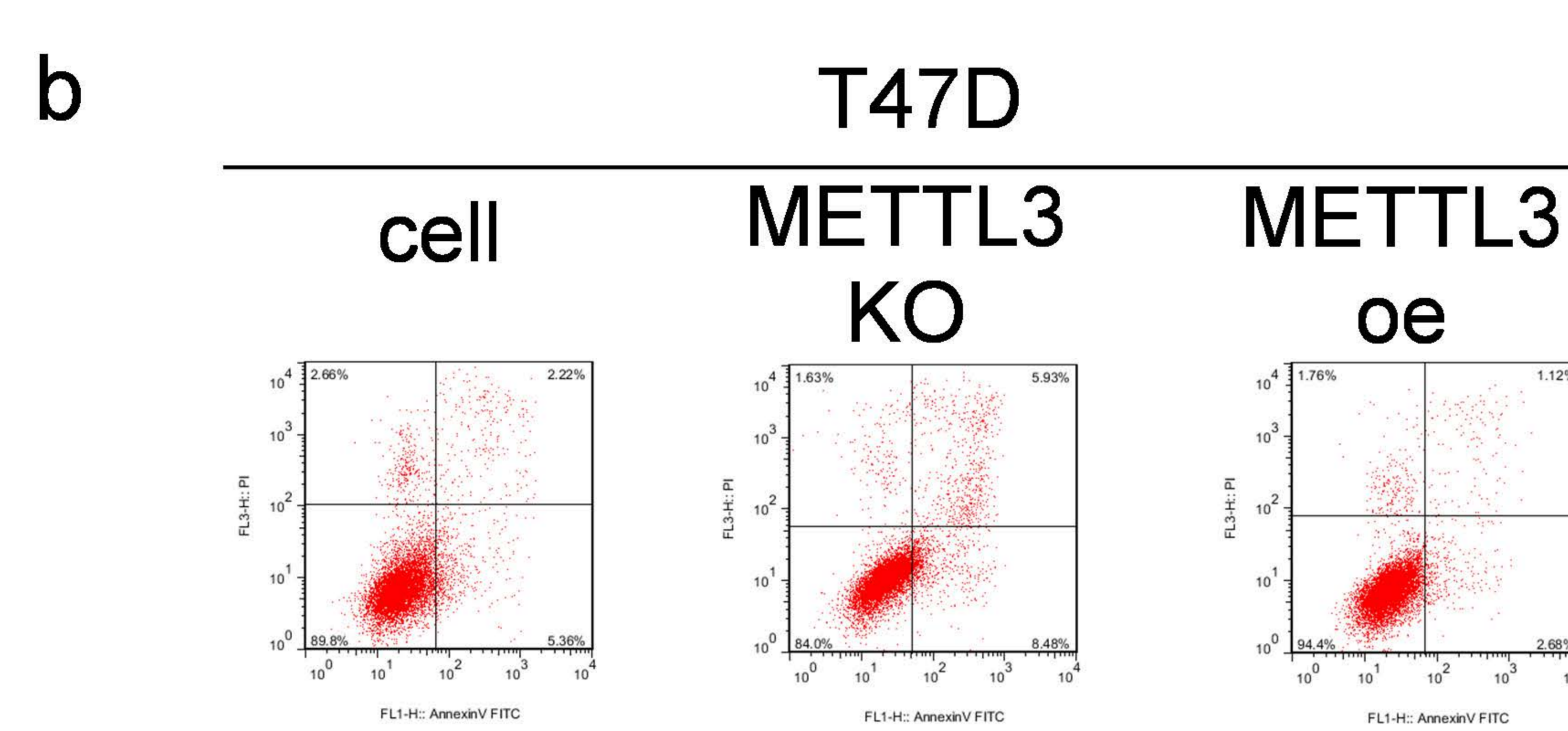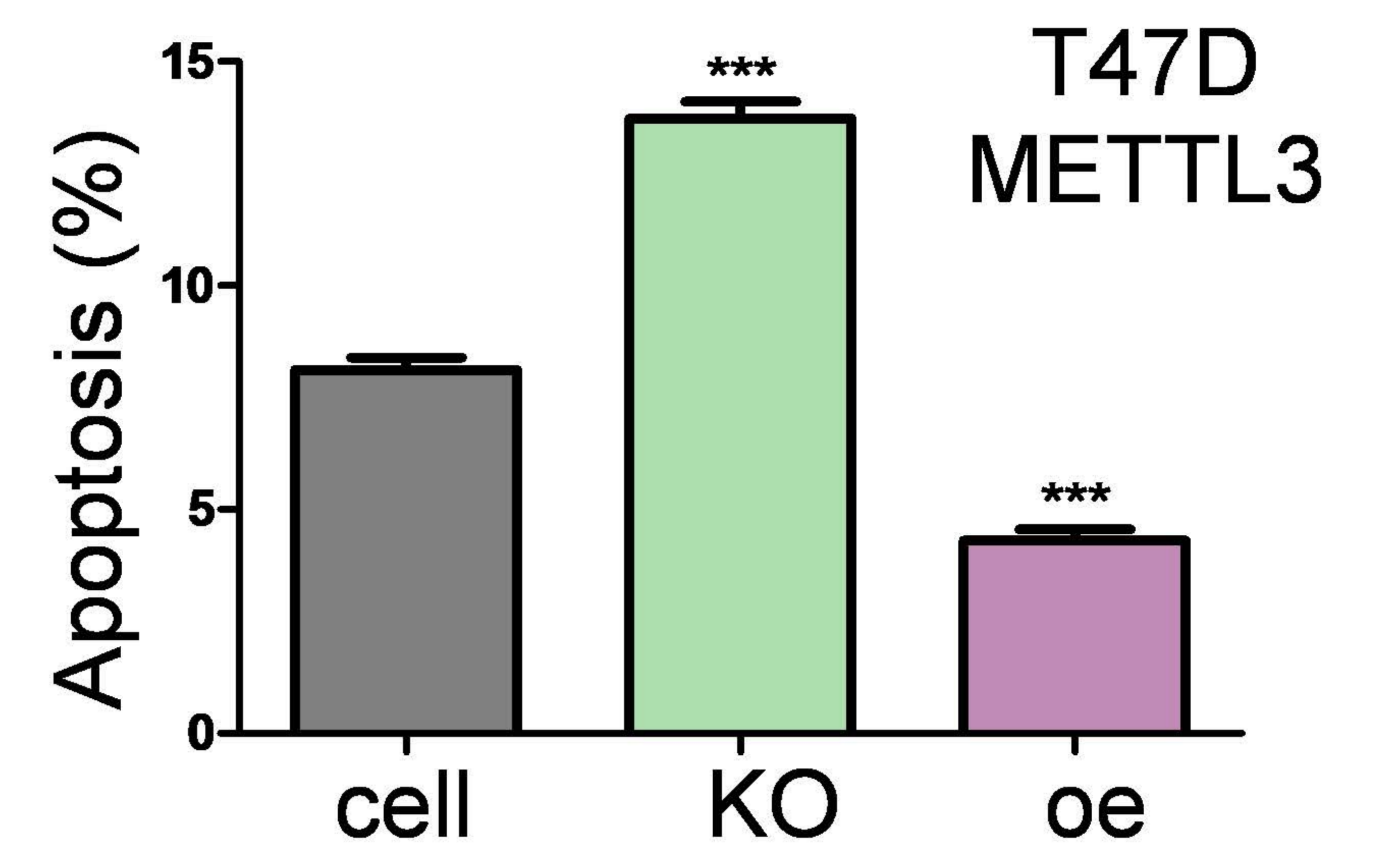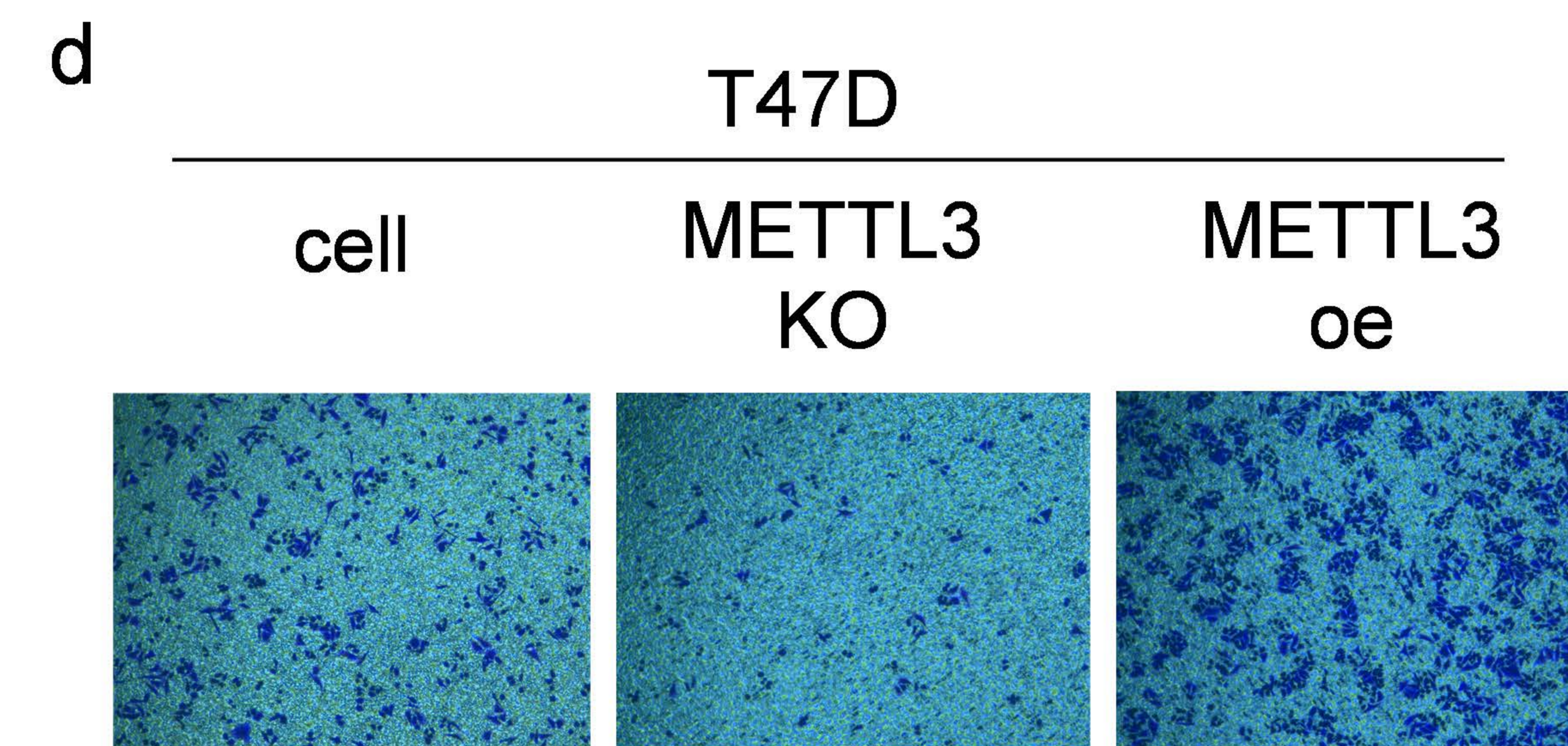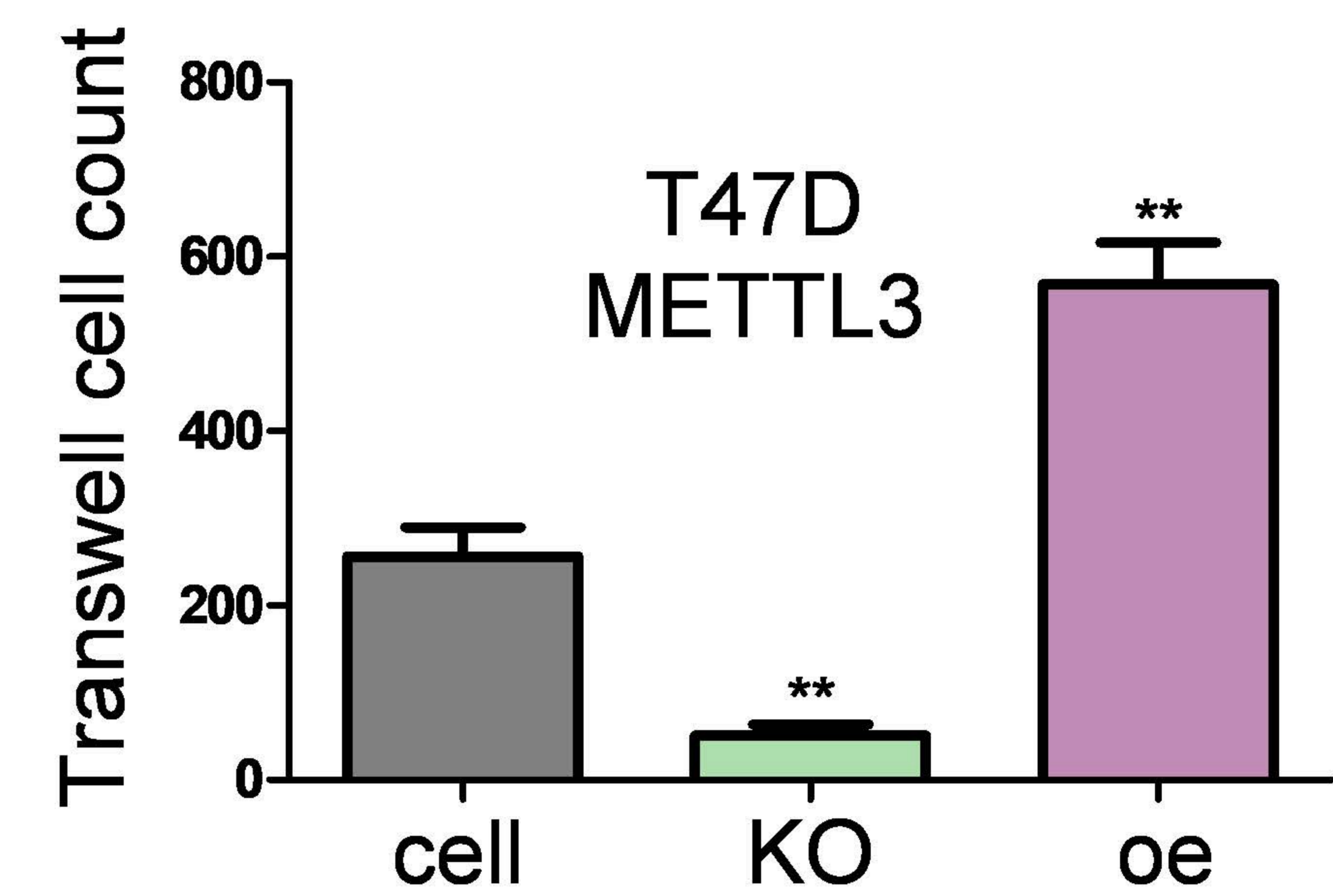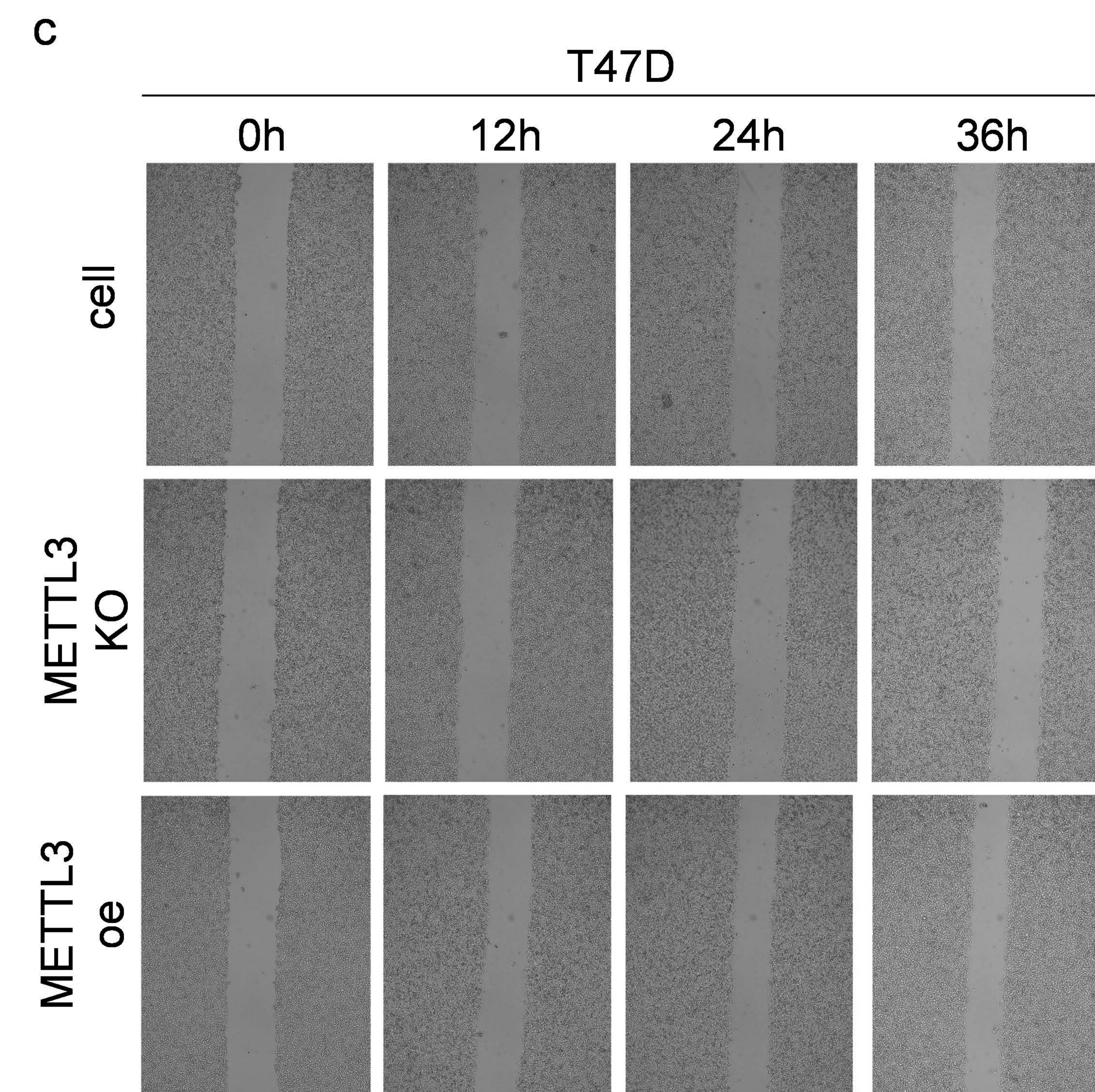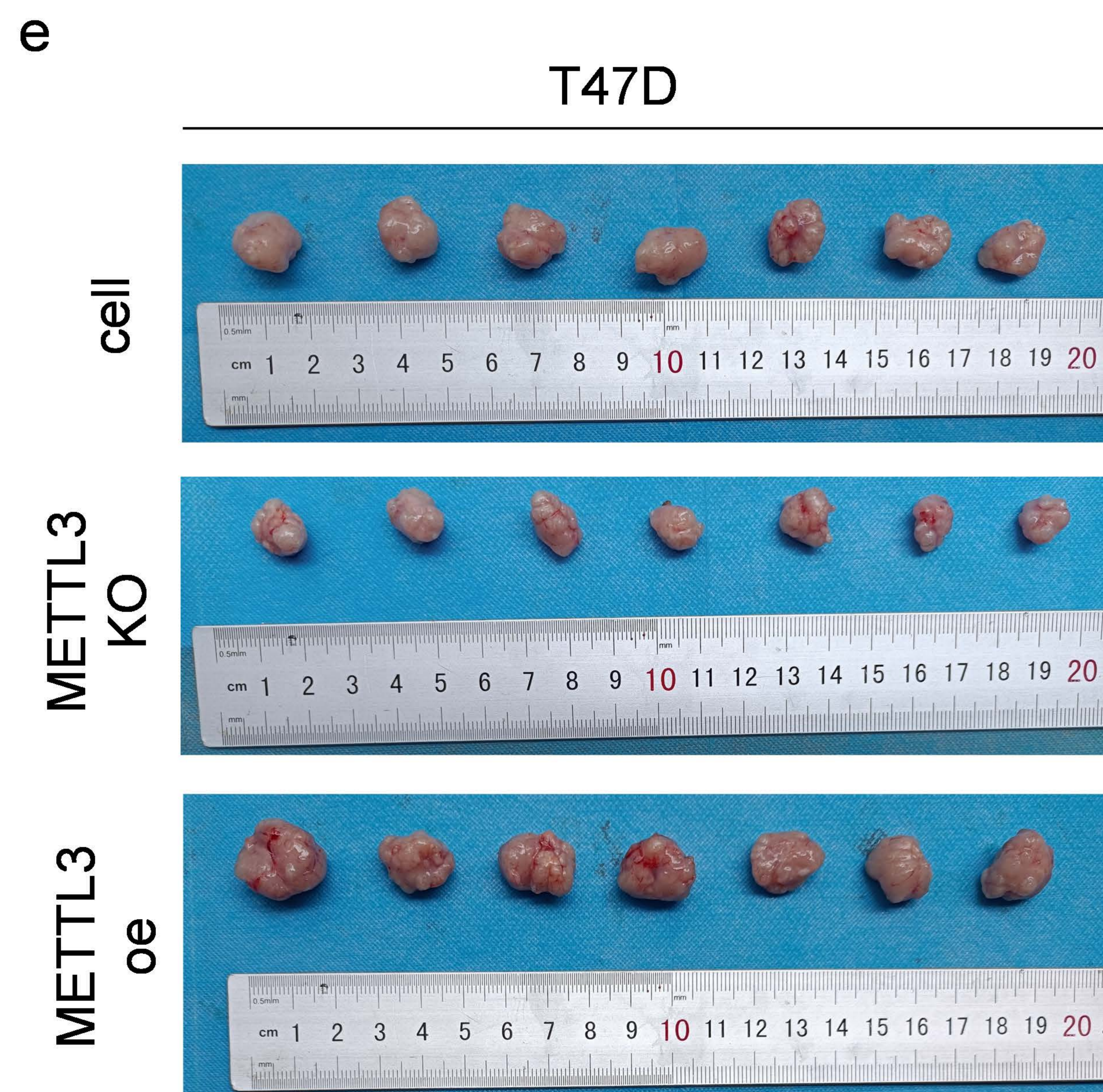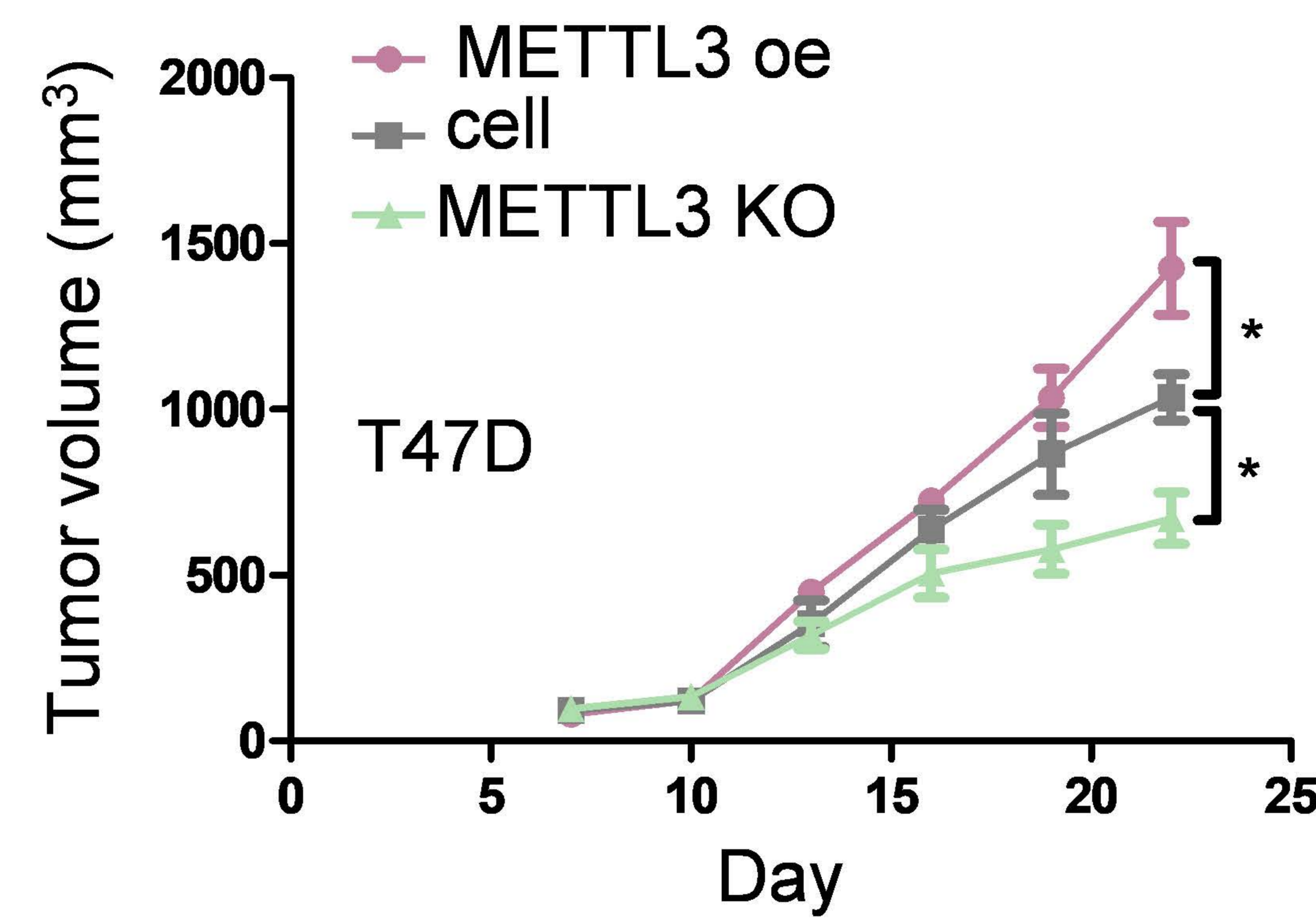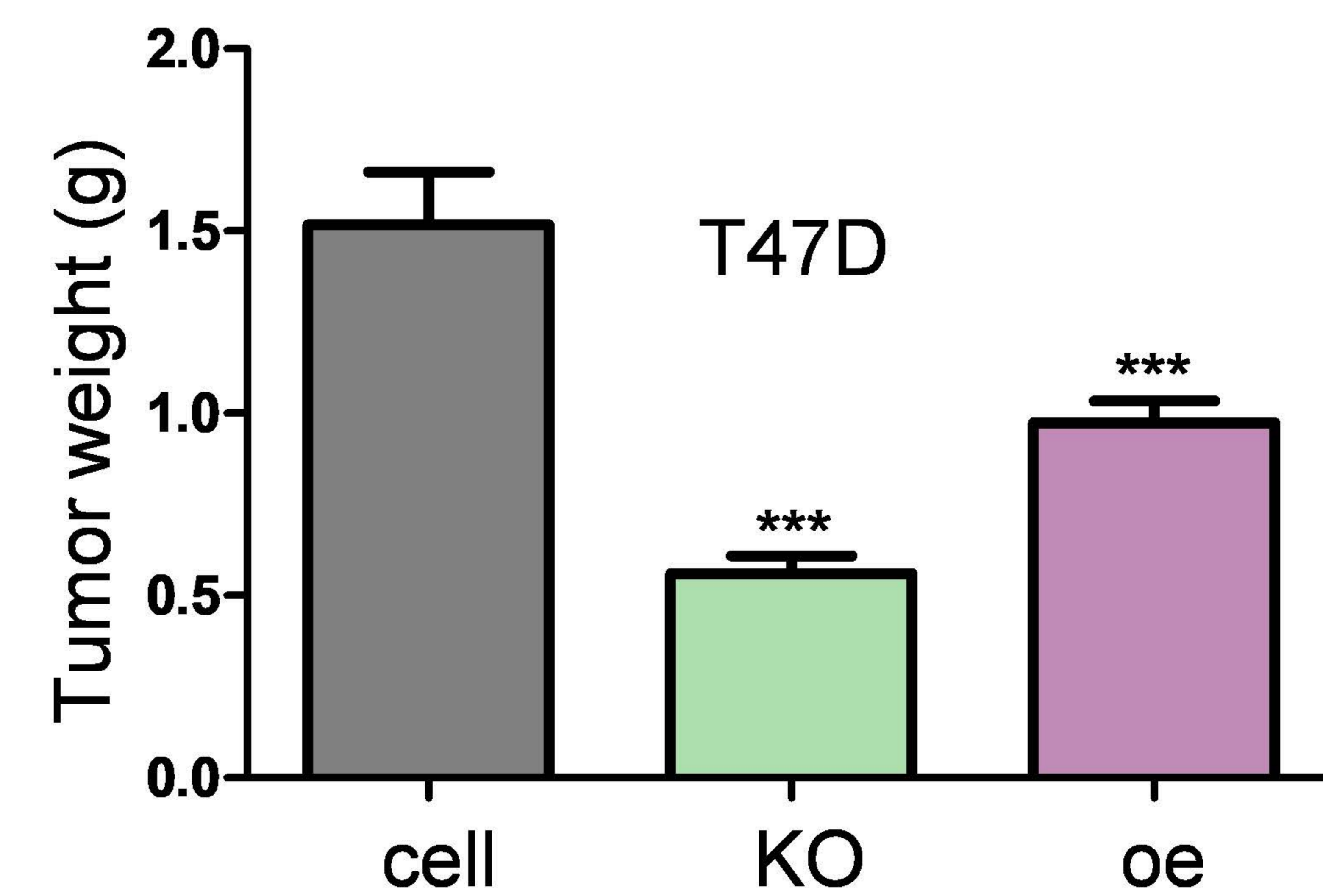

a

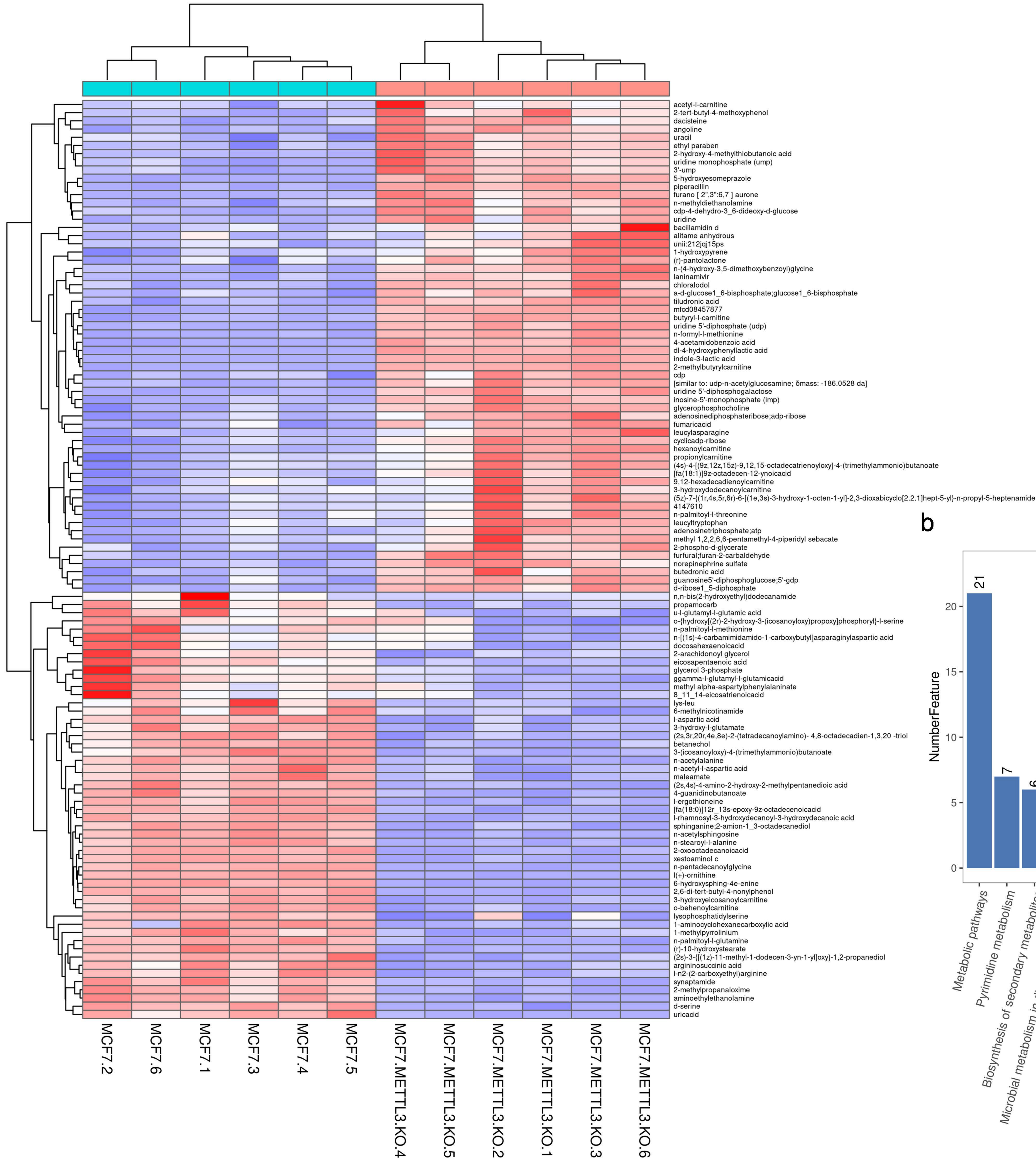

b

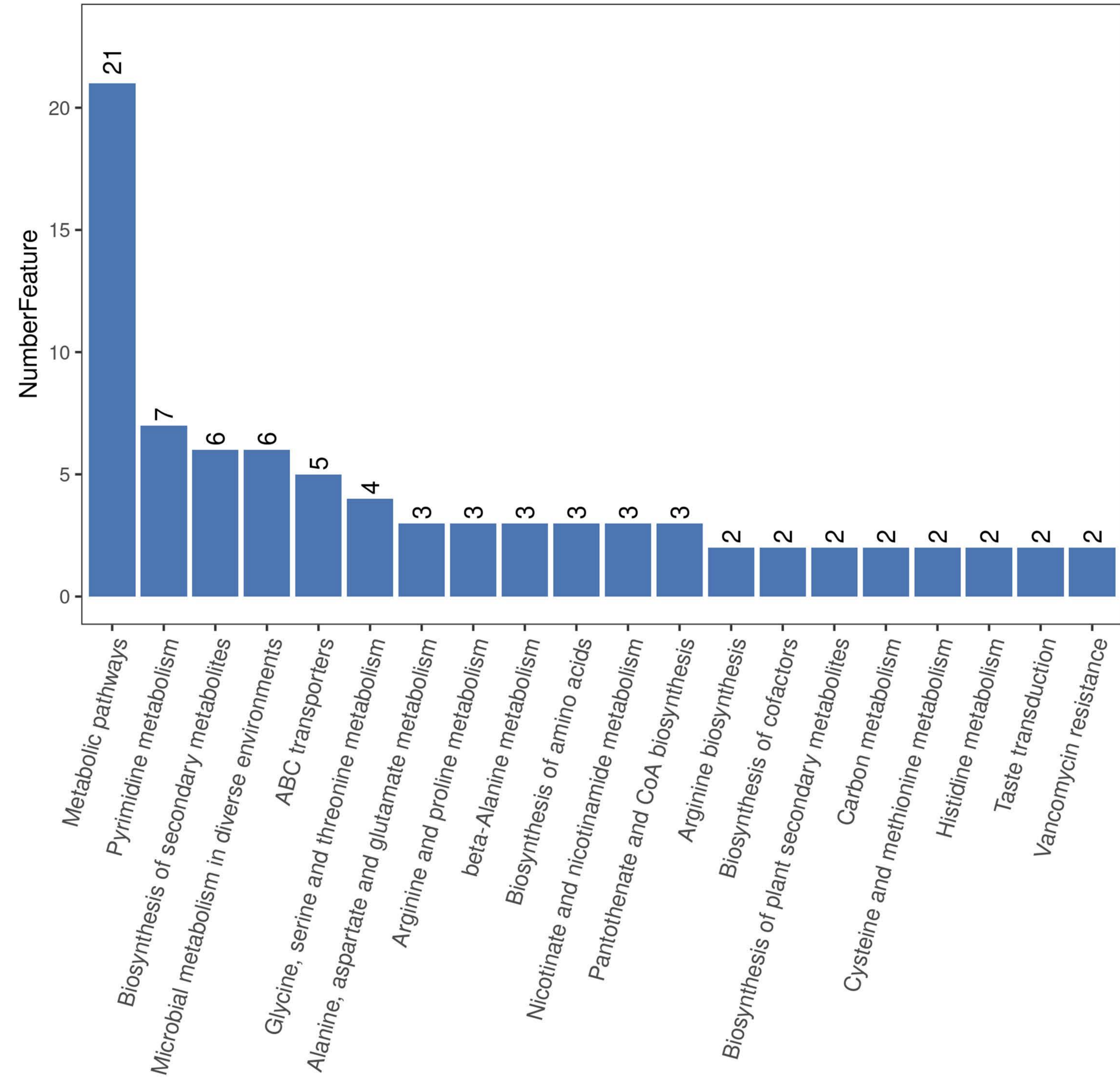

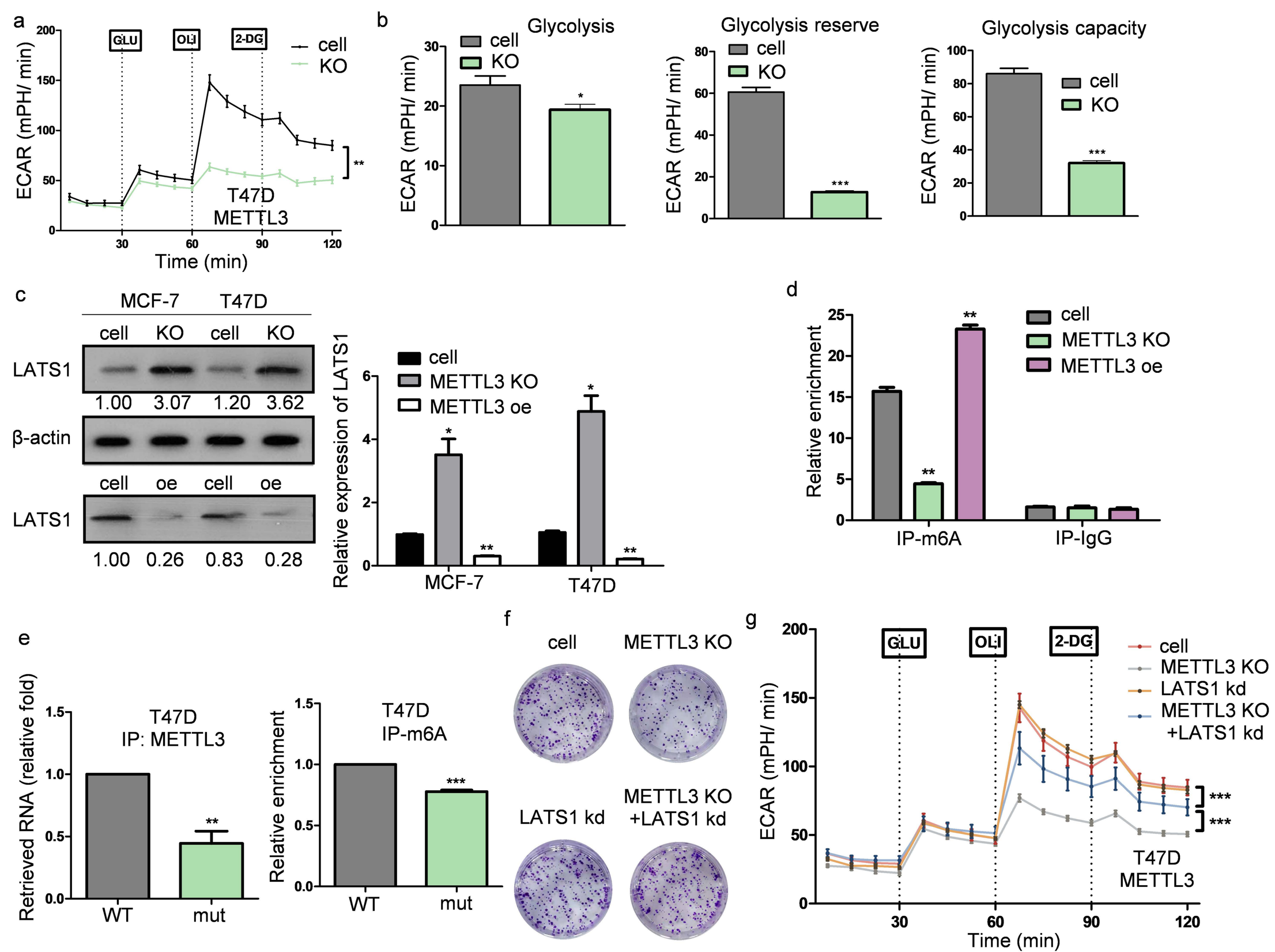

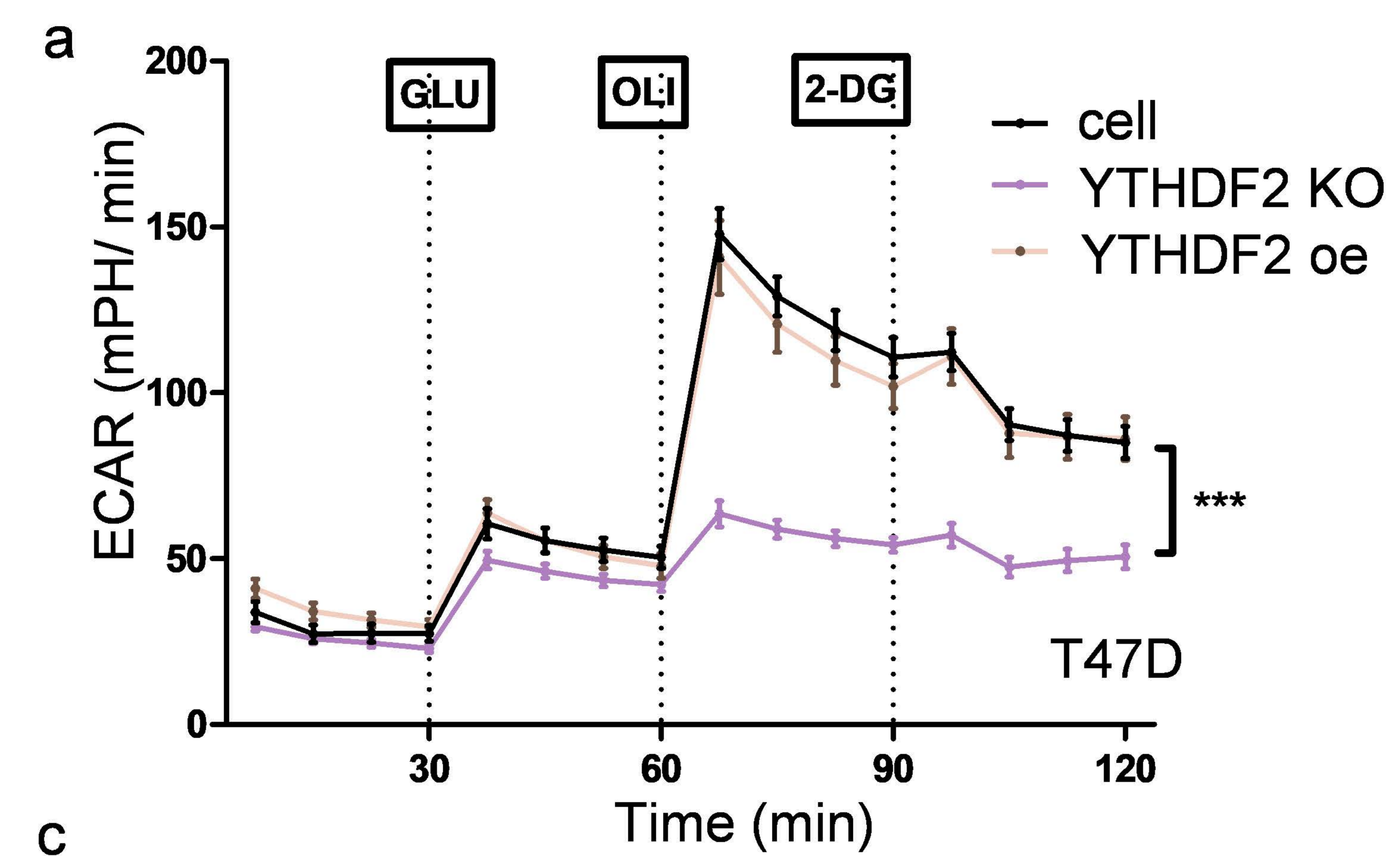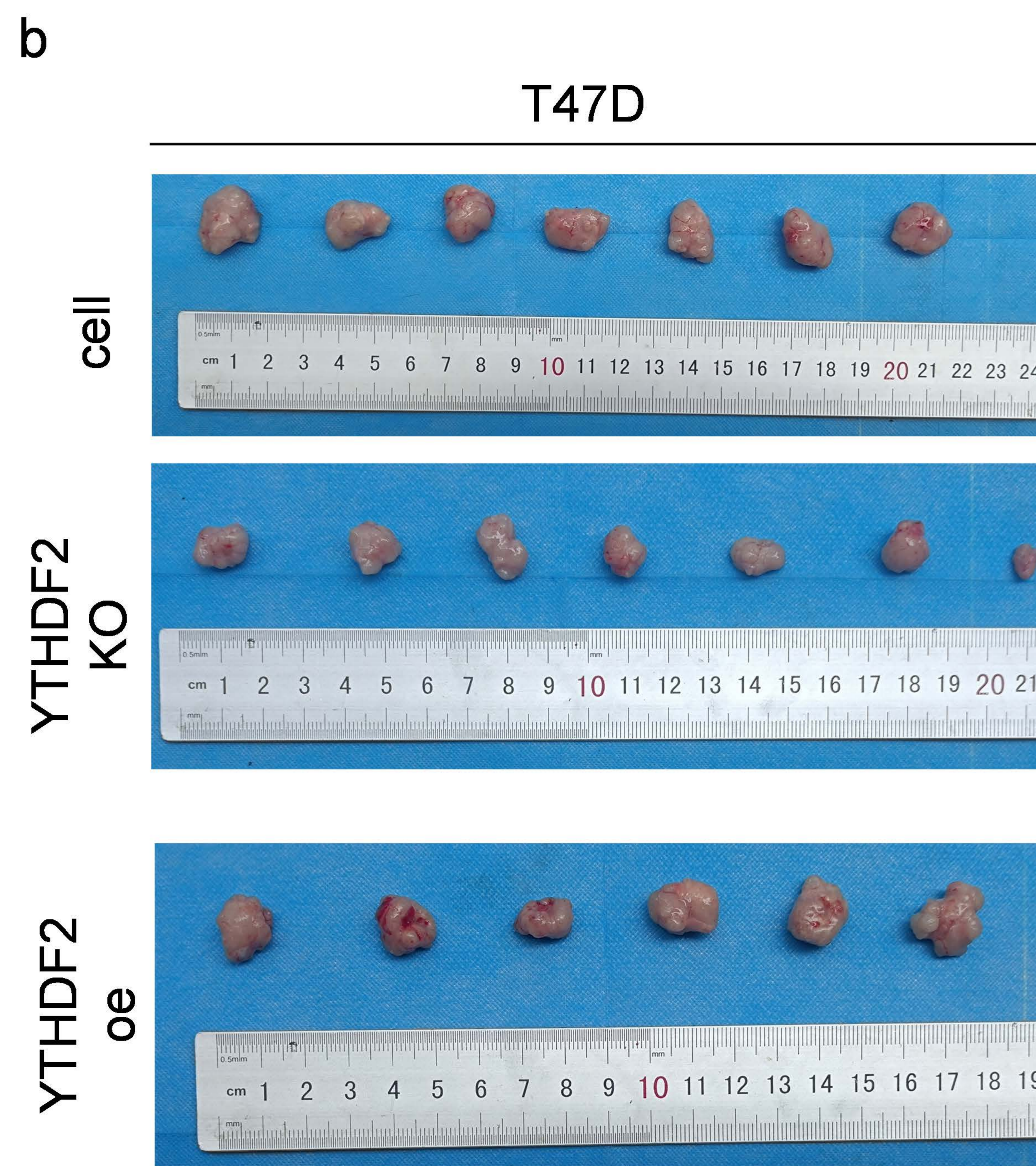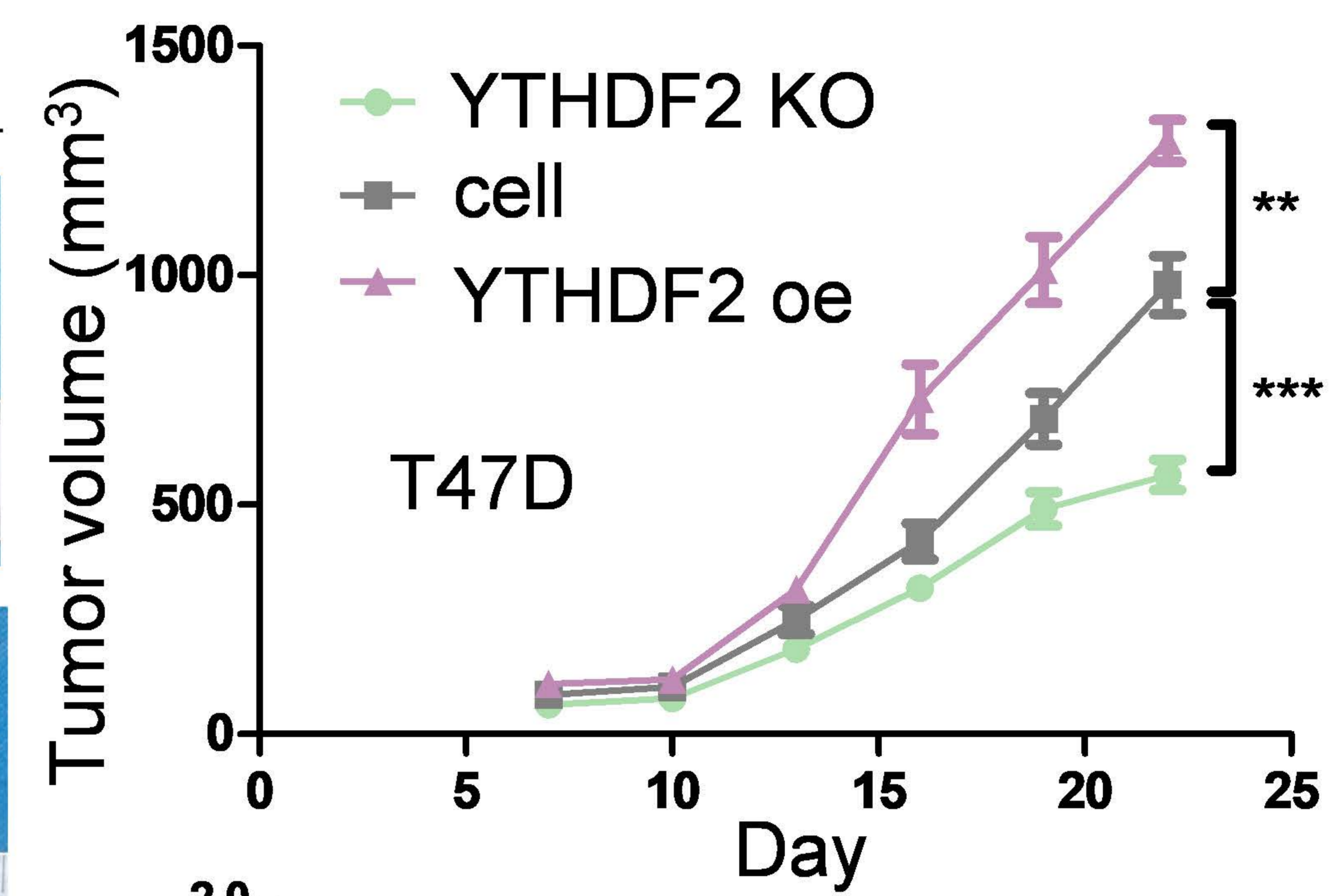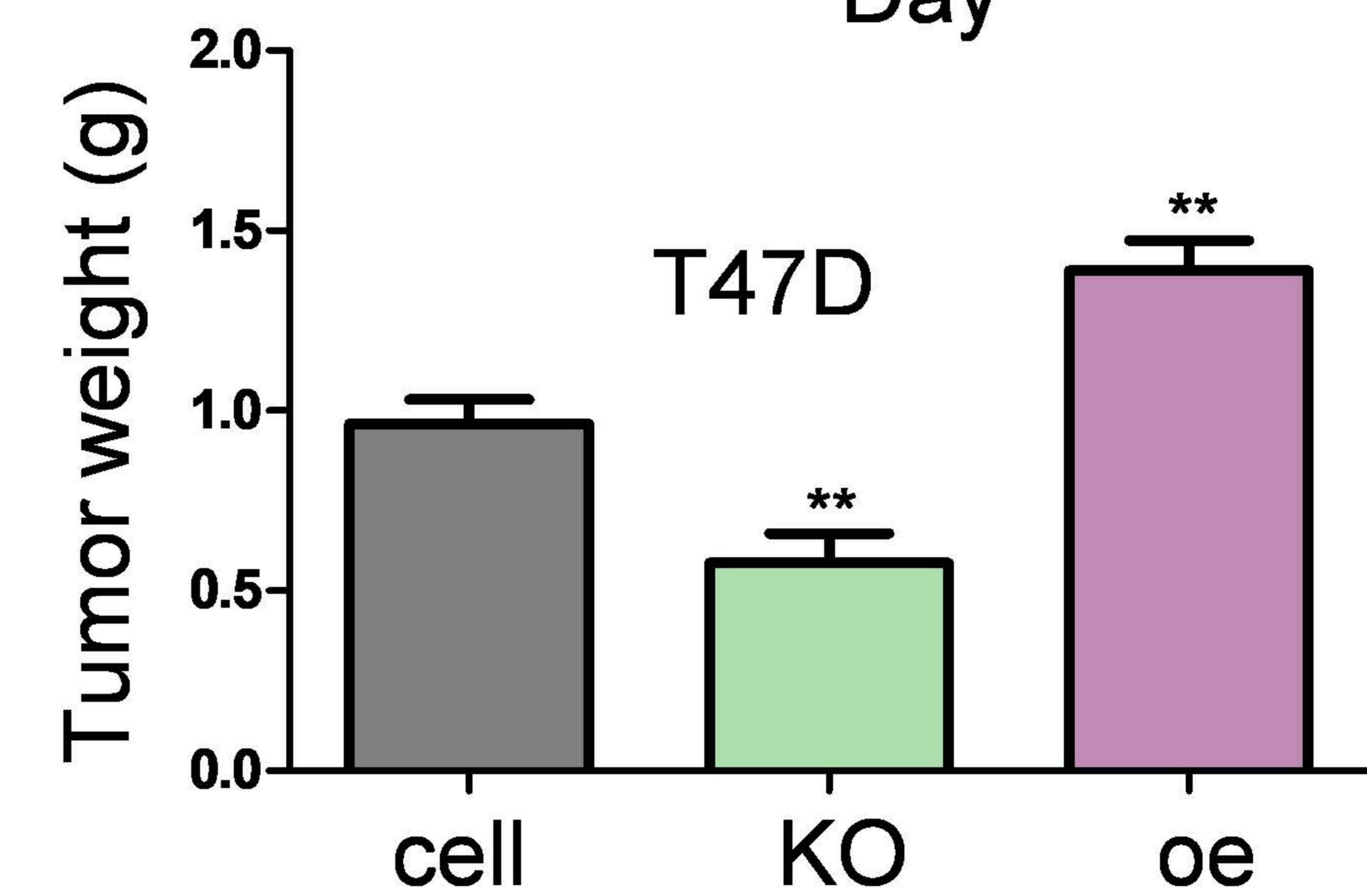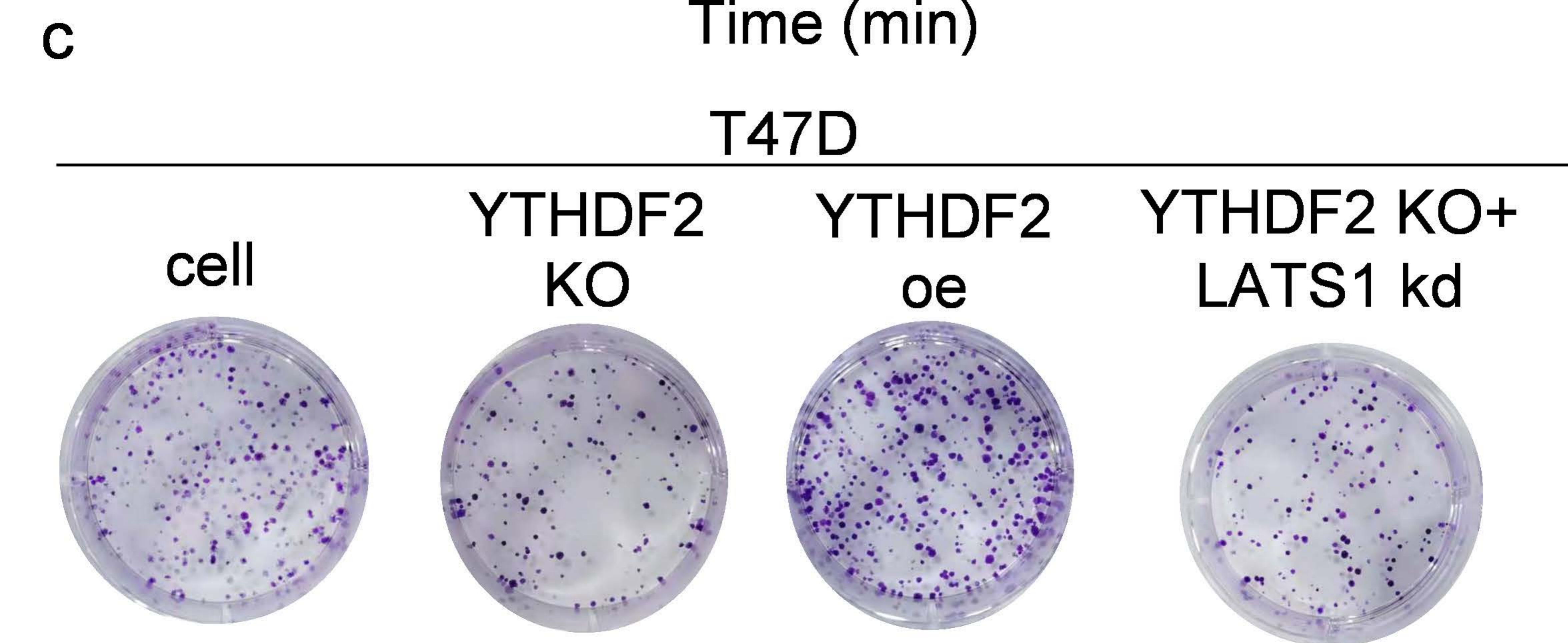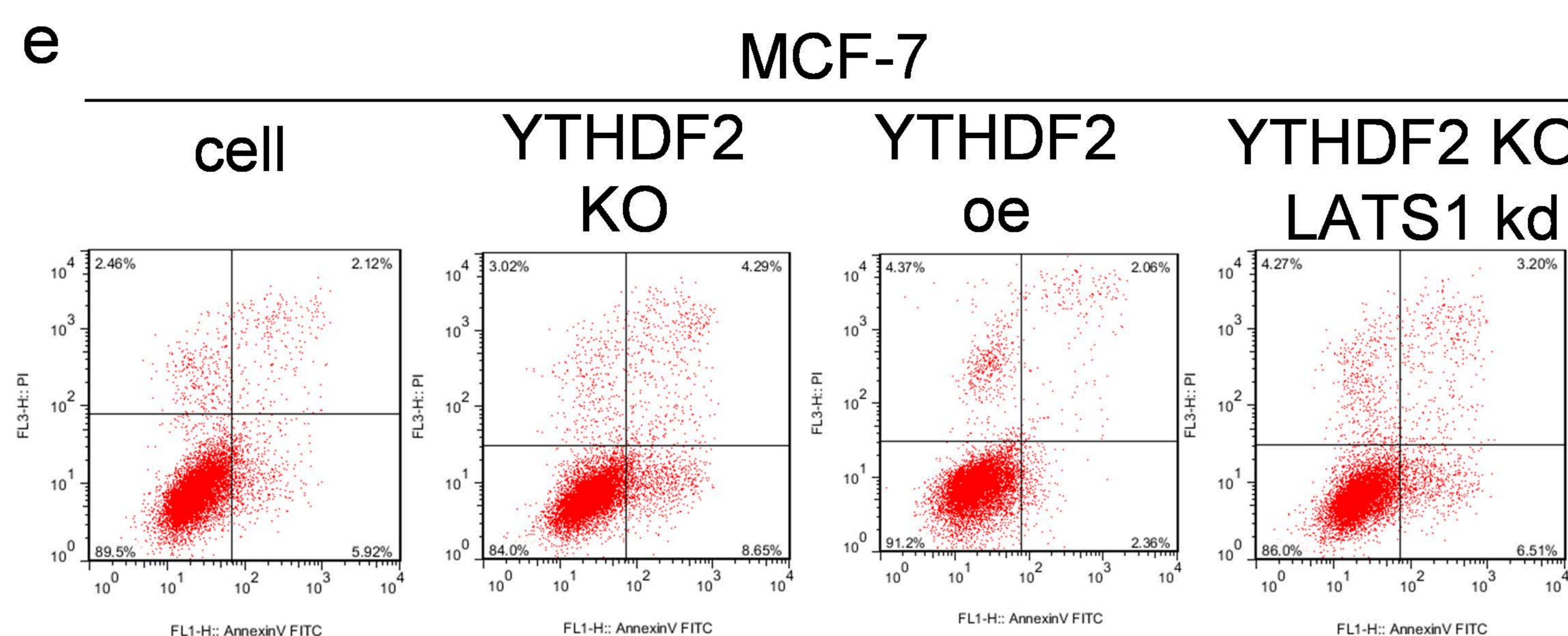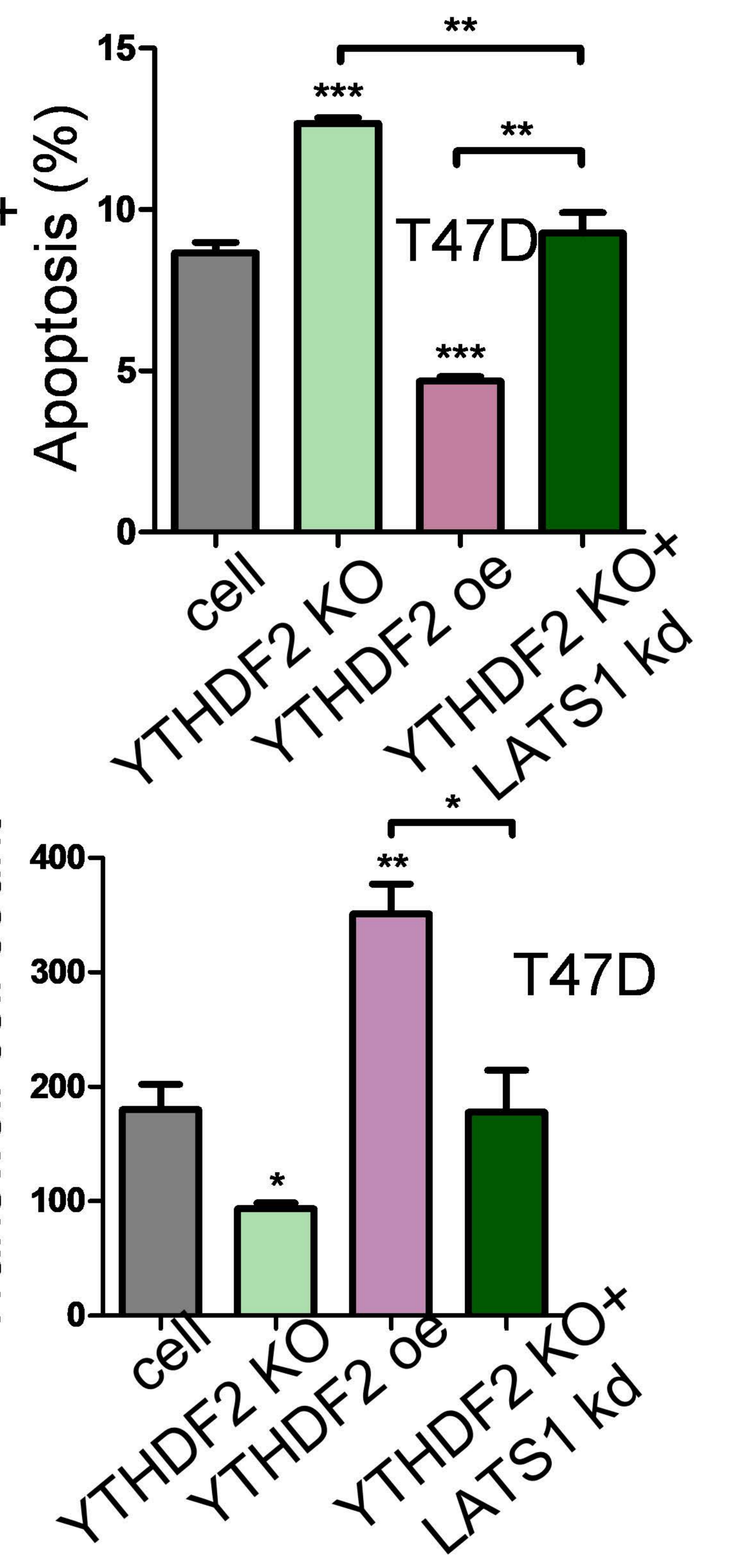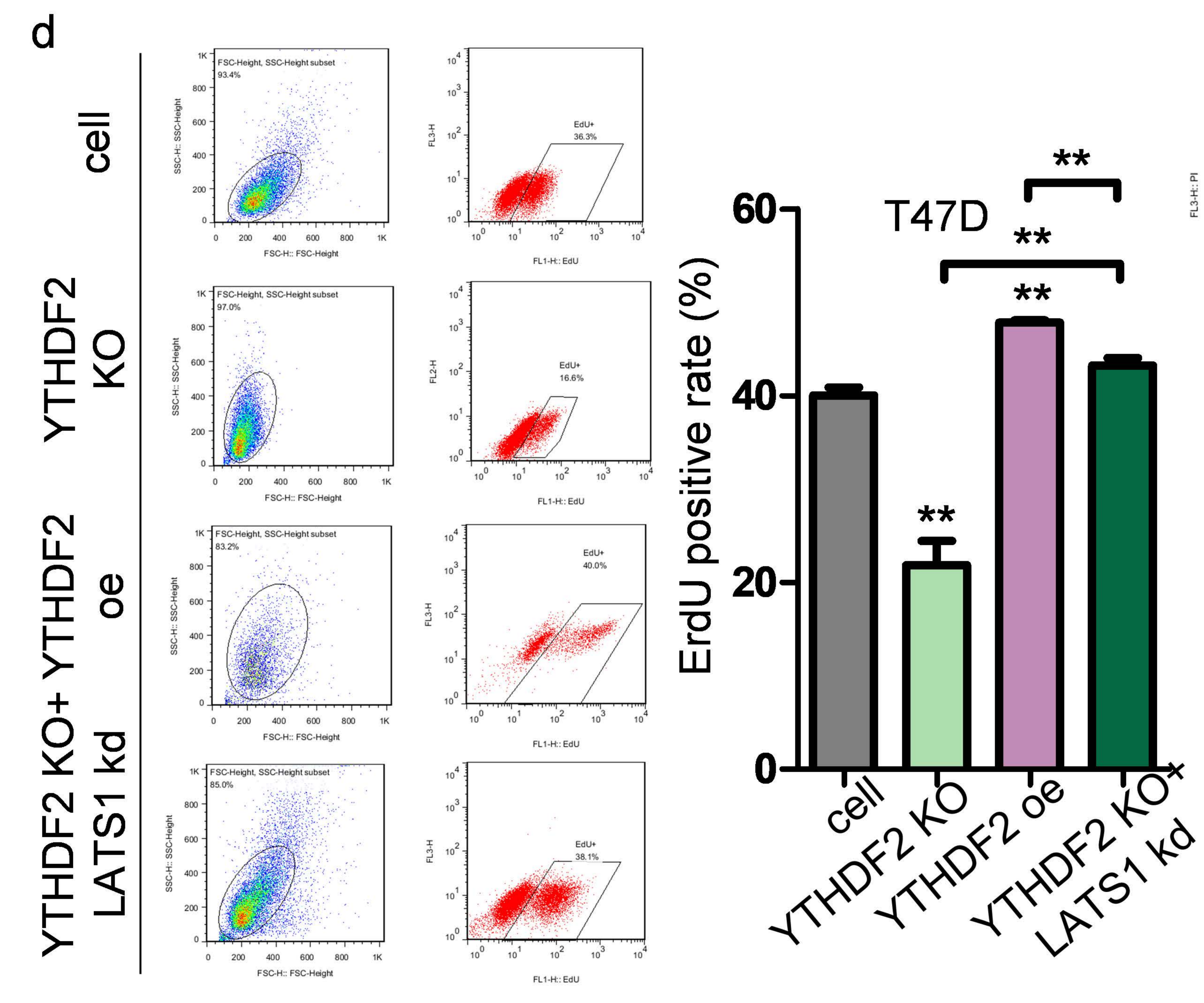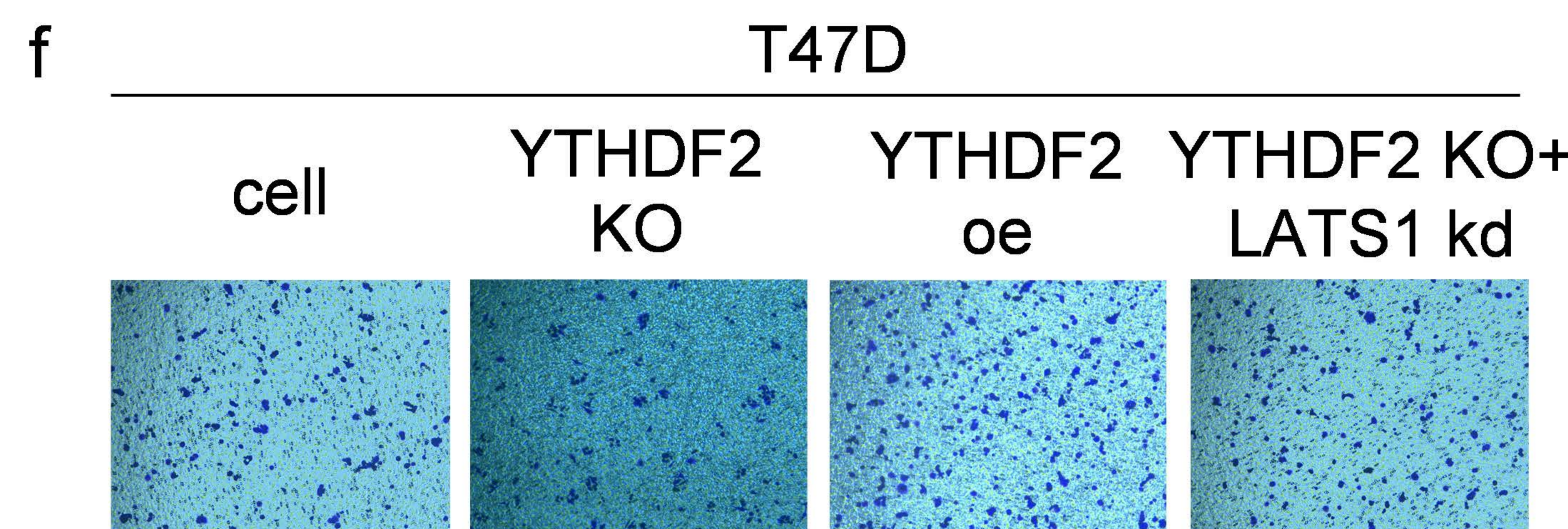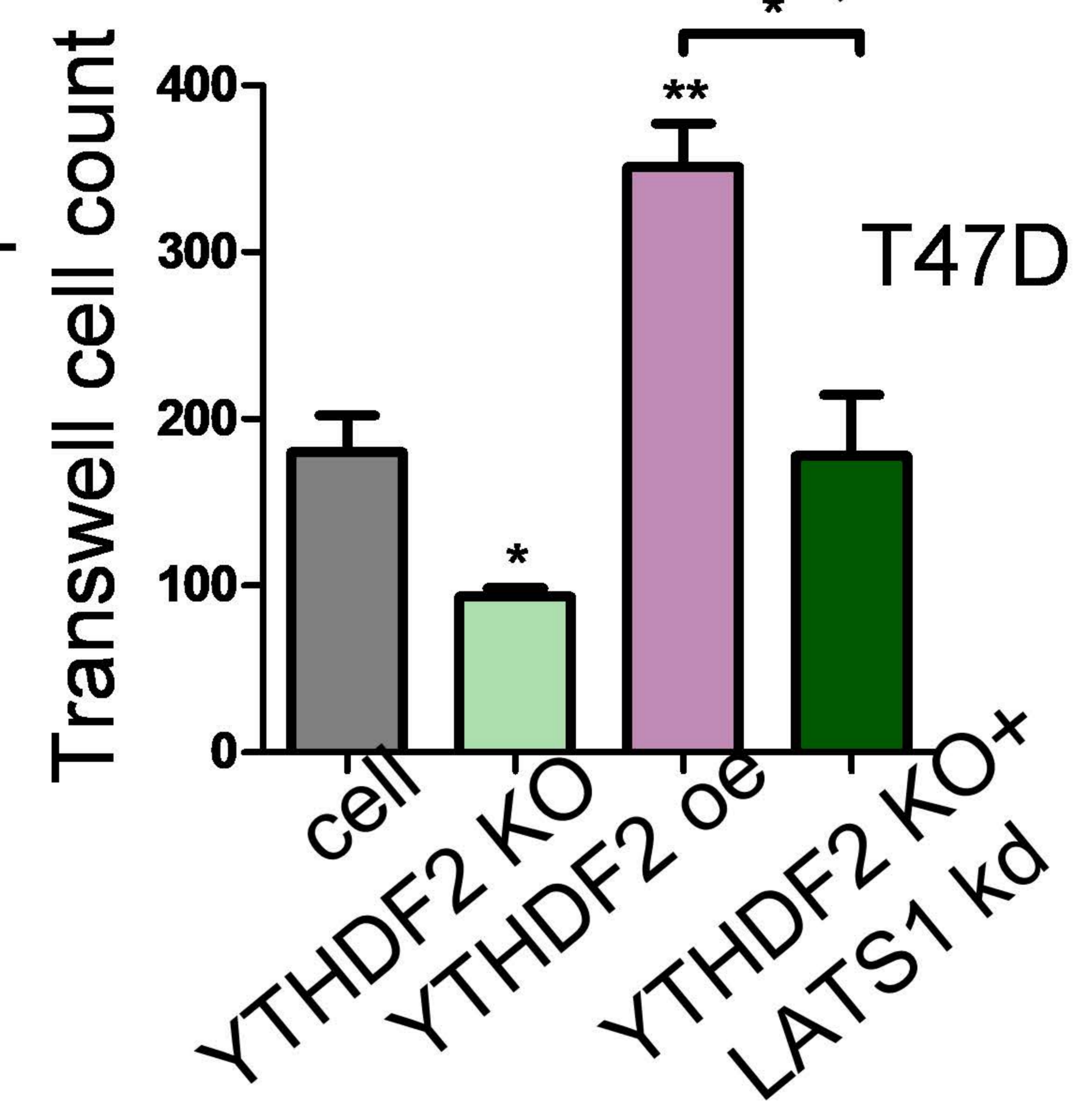

Supplement: Supplementary file 1 — Additional file 1: Supplementary Figure 1. m6A modification levels in the Hippo pathway and metabolism in breast cancer. (a) Venn diagram (left panel) of common (the intersection area) and unique peaks (non-intersection region) of another pair of breast cancer tissue compared with adjacent normal tissue, |log2(fold change)|>1 and q value<0.05. The gene region (right panel) annotated by difference Peaks of breast cancer tissue compared with adjacent normal tissue, |log2(fold change)|>1 and q value<0.05. (b) Differential m6A and differential expression combined analysis diagram of another pair of breast cancer tissue compared with adjacent normal tissue. (c) GO analysis results of differential methylated genes. Differential methylated genes were obtained by TCGA clinical data of breast cancer and compared with MeRIP- seq data. (d) Enrichment analysis result of biological progress (BP) of differential methylated genes. (e) Location map of LATS1 in differential methylated KEGG pathway of Hippo. (f) Volcano map of differentially expressed genes based on transcriptome sequencing of two pairs of breast cancer tissue compared with adjacent normal tissue. (g) Differentially expressed genes of Hippo pathway in transcriptome sequencing of breast cancer tissues compared with adjacent normal tissues, n=2. (h) M6A peaks on LATS1 mRNA originated from MeRIP-seq. M6A peaks were visualized by IGV. The red peaks show the results of MeRIP and the blue peaks represent the input. Supplementary Figure 2. Expression of m6A proteins in breast cancer tissue and cells. (a) The correlation between METTL3 and the expression level of ER/ PR/ HER2 in breast adenocarcinomas (GSE70951, 195 breast adenocarcinomas and matched adjacent normal breast tissue samples). (b) The correlation between METTL3 protein expression and the prognosis of Luminal A (upper panel) and Luminal B (lower panel) breast adenocarcinomas. (c) Alter the mRNA expression level of METTL3 in MCF-7 and T47D cells, ** p<0.01. (d) Al [file 13046_2022_2581_MOESM1_ESM.pdf]
