# Supplementary material for: The N6-methyladenosine METTL3 regulates tumorigenesis and glycolysis by mediating m6A methylation of the tumor suppressor LATS1 in breast cancer
Source: J Exp Clin Cancer Res. 2023 Jan 7;42:10. doi: 10.1186/s13046-022-02581-1 (PMC9824909; doi:10.1186/s13046-022-02581-1)
Supplement: Supplementary file 2 — Additional file 2: Supplementary Table 1. Data of the included patients. Clinicopathological information on age, sex, clinical stage, neoadjuvant therapy, adjuvant therapy, ER, PR, HER2, Ki67, histological subtype and histological grade of each included patient. Supplementary Table 2. Differential m6A sites in two groups. Specific sites of 1850 up regulated and 1807 down regulated genes of breast cancer cells, p-values <0.05 were indicated. Supplementary Table 3. The relationship between METTL3 and patient clinicopathological features. The age of patients was significantly related to the expression of METTL3, p-values <0.05 were indicated. Supplementary Table 4. KEGG analysis of MCF-7 between METTL3 KO group and control group. KEGG pathways of differentially expressed genes that were altered significantly after METTL3 knockout, p-values <0.05 were indicated. Supplementary Table 5. Metabolites involved in METTL3 knockout in MCF-7. KEGG pathways of different metabolites that were altered significantly after knocking out METTL3 expression, p-values <0.05 were indicated. [file 13046_2022_2581_MOESM2_ESM.pdf]

Supplementary Table 1. Data of the included patients.

| | | | | | Medication history | | Receptor status | | | | | | |
|---|---|---|---|---|---|---|---|---|---|---|---|---|---|
| Patient code | Specimen source | Age | Sex | Clinical stage | Neoadjuvant therapy | Adjuvant therapy | ER | PR | HER2 | Ki67 | Histological subtype | Histological grade | Note (ID) |
| BC1 | breast | 36 | female | T2N1M0 | / | Cyclophosphamide + Epirubicin (Farmasin 135mg, Andosan 0.9g) | - | - | +++ | + | invasive ductal carcinoma | III | ZA266524 |
| BC2 | breast | 41 | female | T2N1M1 | / | Nored endocrine therapy | - | - | +++ | + | invasive ductal carcinoma | II | 821378 |
| BC3 | breast | 61 | female | T2N0M0 | / | Cyclophosphamide + Epirubicin (Farmasin 135mg, Andosan 0.98g) | + | + | + | + | invasive ductal carcinoma | II | 996724 |
| BC4 | breast | 80 | female | T2N0M0 | / | endocrinotherapy | - | - | + | + | uctal carcinoma, left mam | II | ZA4125700 |
| BC5 | breast | 82 | female | T2N0M0 | / | / | ++ | - | - | + | invasive ductal carcinoma | II | ZA4381792 |
| BC6 | breast | 68 | female | T1N0M0 | / | yclophosphamide + Paclitaxel liposome (Paexin 1.2g, Liprosol 280mg | ++ | - | - | + | invasive ductal carcinoma | II | ZA0608472 |
| BC7 | breast | 81 | female | T3N1M0 | / | / | + | - | - | + | invasive ductal carcinoma | III | 34446371 |
| BC8 | breast | 60 | female | T2N1M0 | / | Lipak 280mg+ Andosen 0.95g+ Herceptin 0.39g | - | - | +++ | + | invasive ductal carcinoma | II | 1176367 |

Legend: Clinicopathological information on age, sex, clinical stage, neoadjuvant therapy, adjuvant therapy, ER, PR, HER2, Ki67, histological subtype and histological grade of each included patient..

Supplementary Table 2. Differential m6A sites in the two groups.

| peak | log2FC | p_value | q_value | fold_enrchment | fe.p | fe.fdr | chr | chromStart | chromEnd | name | score | strand | thickStart | thickEnd | blockSizes |
|---|---|---|---|---|---|---|---|---|---|---|---|---|---|---|---|
| peak7 | 5.36 | 0.004365158 | 0.023988329 | 4.15 | 0.000186209 | 0.000758578 | chr1 | 788836 | 788937 | NR_047524.1 | 0.00019 | + | 788836 | 788937 | 101, |
| peak10 | 5.42 | 0.000645654 | 0.006025596 | 4.23 | 1.54882E-06 | 1.04713E-05 | chr1 | 788835 | 788986 | NR_047525.1 | 0.0000015 | + | 788835 | 788986 | 151, |
| peak71 | 1.25 | 0.00011749 | 0.001737801 | 8.43 | 1.58489E-05 | 8.12831E-05 | chr1 | 10364319 | 10364519 | NM_001365953.1 | 0.000016 | + | 10364319 | 10364519 | 200, |
| peak107 | 1.13 | 0.004073803 | 0.022387211 | 18.3 | 1.25893E-11 | 4.0738E-10 | chr1 | 14108058 | 14142997 | NM_012231.4 | 1.3E-11 | + | 14108058 | 14142997 | 1268,76, |
| peak114 | 2.62 | 0.000269153 | 0.003162278 | 54.8 | 0.000169824 | 0.000691831 | chr1 | 15894588 | 15894738 | NM_001287811.1 | 0.00017 | + | 15894588 | 15894738 | 150, |
| peak115 | 2.64 | 0.000251189 | 0.003019952 | 54.8 | 0.000162181 | 0.000676083 | chr1 | 15894587 | 15894738 | NM_015291.4 | 0.00016 | + | 15894587 | 15894738 | 151, |
| peak121 | 1.28 | 1.41254E-07 | 9.33254E-06 | 47.4 | 3.38844E-10 | 7.76247E-09 | chr1 | 15986766 | 15987814 | NM_032341.5 | 3.4E-10 | + | 15986766 | 15987814 | 1048, |
| peak138 | 2.16 | 5.12861E-07 | 2.81838E-05 | 11.3 | 2.75423E-05 | 0.000134896 | chr1 | 16265901 | 16266502 | NM_015001.3 | 0.000028 | + | 16265901 | 16266502 | 601, |
| peak436 | 2.9 | 7.24436E-06 | 0.000229087 | 4.54 | 7.58578E-07 | 5.62341E-06 | chr1 | 26448414 | 26448614 | NM_001243532.1 | 0.00000076 | + | 26448414 | 26448614 | 200, |
| peak437 | 2.84 | 5.88844E-05 | 0.001071519 | 4.17 | 7.58578E-07 | 3.0903E-05 | chr1 | 26448712 | 26449110 | NM_001243532.1 | 0.00000076 | + | 26448712 | 26449110 | 398, |
| peak438 | 1.29 | 5.49541E-05 | 0.001023293 | 4.46 | 2.29087E-08 | 2.75423E-07 | chr1 | 26448385 | 26448633 | NM_001243533.2 | 0.000000023 | + | 26448385 | 26448633 | 248, |
| peak439 | 3.44 | 0.000758578 | 0.00676083 | 4.71 | 2.29087E-08 | 1.62181E-06 | chr1 | 26448731 | 26449078 | NM_001243533.2 | 0.000000023 | + | 26448731 | 26449078 | 347, |
| peak441 | 3.47 | 0.000645654 | 0.006025596 | 4.71 | 3.54813E-08 | 2.34423E-06 | chr1 | 26448726 | 26449073 | NM_152835.5 | 0.000000035 | + | 26448726 | 26449073 | 347, |
| peak449 | 2.46 | 0.002290868 | 0.014791084 | 3.2 | 2.51189E-05 | 0.000123027 | chr1 | 26900667 | 26901167 | NM_002953.4 | 0.000025 | + | 26900667 | 26901167 | 500, |
| peak452 | 2.89 | 0.00025704 | 0.003019952 | 3.1 | 0.000134896 | 0.000562341 | chr1 | 26900674 | 26901120 | NM_001006665.2 | 0.00013 | + | 26900674 | 26901120 | 446, |
| peak467 | 3.44 | 0.000776247 | 0.00691831 | 4.78 | 0.000165959 | 0.000691831 | chr1 | 28817521 | 28823078 | NM_001048183.3 | 0.00017 | + | 28817521 | 28823078 | 3,138,77,33, |
| peak471 | 6.14 | 4.7863E-05 | 0.000933254 | 4.67 | 5.62341E-05 | 0.00025704 | chr1 | 28818175 | 28819553 | NM_001350158.2 | 0.000056 | + | 28818175 | 28819553 | 124,27, |
| peak475 | 6.14 | 4.7863E-05 | 0.000933254 | 4.67 | 6.16595E-05 | 0.000275423 | chr1 | 28818175 | 28819552 | NM_001350159.2 | 0.000062 | + | 28818175 | 28819552 | 124,26, |
| peak479 | 3.44 | 0.000776247 | 0.00691831 | 4.78 | 0.000147911 | 0.000616595 | chr1 | 28817521 | 28823078 | NM_001350160.2 | 0.00015 | + | 28817521 | 28823078 | 3,138,77,33, |
| peak483 | 6.16 | 2.45471E-05 | 0.00057544 | 4.67 | 8.51138E-05 | 0.000371535 | chr1 | 28818173 | 28819550 | NM_001350161.2 | 0.000085 | + | 28818173 | 28819550 | 126,24, |
| peak487 | 3.79 | 0.001071519 | 0.008709636 | 5.38 | 9.77237E-05 | 0.00042658 | chr1 | 28818164 | 28823083 | NM_023923.4 | 0.000098 | + | 28818164 | 28823083 | 135,77,38, |
| peak491 | 2.9 | 0.001698244 | 0.012022644 | 2.58 | 4.36516E-05 | 0.000204174 | chr1 | 28864432 | 28864531 | NR_030726.2 | 0.000044 | + | 28864432 | 28864531 | 99, |
| peak493 | 2.99 | 0.000346737 | 0.003801894 | 2.92 | 4.0738E-05 | 0.000190546 | chr1 | 28864436 | 28864584 | NM_001048195.3 | 0.000041 | + | 28864436 | 28864584 | 148, |
| peak495 | 3.03 | 0.002951209 | 0.017782794 | 2.92 | 0.000134896 | 0.000562341 | chr1 | 28864437 | 28864536 | NM_001269.5 | 0.00013 | + | 28864437 | 28864536 | 99, |
| peak514 | 4.76 | 0.003388442 | 0.019498446 | 8.5 | 0.00020893 | 0.000851138 | chr1 | 31821771 | 31836974 | NM_001282566.1 | 0.00021 | + | 31821771 | 31836974 | 50,96, |
| peak587 | 3.43 | 0.000331131 | 0.003715352 | 14.2 | 0.00020893 | 0.000851138 | chr1 | 39853592 | 39853843 | NM_012090.5 | 0.00021 | + | 39853592 | 39853843 | 251, |
| peak595 | 1.9 | 0.000489779 | 0.004897788 | 4.36 | 0.004786301 | 0.016218101 | chr1 | 39876932 | 39877082 | NM_015038.2 | 0.0048 | + | 39876932 | 39877082 | 150, |
| peak598 | 3.62 | 3.54813E-05 | 0.000758578 | 9.29 | 0.00060256 | 0.002238721 | chr1 | 40705495 | 40705646 | NM_012421.4 | 0.0006 | + | 40705495 | 40705646 | 151, |
| peak612 | 4.05 | 0.001949845 | 0.013182567 | 3.31 | 0.000794328 | 0.002951209 | chr1 | 42925577 | 42925823 | NM_001287507.1 | 0.00079 | + | 42925577 | 42925823 | 246, |
| peak614 | 4.05 | 0.000933254 | 0.007943282 | 3.31 | 4.16869E-05 | 0.000194984 | chr1 | 42925576 | 42925822 | NM_001287508.1 | 0.000042 | + | 42925576 | 42925822 | 246, |
| peak617 | 4.28 | 0.000263027 | 0.003090295 | 3.31 | 2.95121E-05 | 0.000141254 | chr1 | 42925575 | 42925822 | NM_024664.4 | 0.00003 | + | 42925575 | 42925822 | 247, |
| peak619 | 2.46 | 0.002398833 | 0.015488166 | 10.5 | 0.000954993 | 0.003467369 | chr1 | 43916121 | 43916422 | NM_001365999.1 | 0.00095 | + | 43916121 | 43916422 | 301, |
| peak655 | 2.18 | 4.57088E-05 | 0.000891251 | 9.29 | 2.69153E-06 | 1.69824E-05 | chr1 | 55207431 | 55207781 | NR_037639.2 | 0.0000027 | + | 55207431 | 55207781 | 350, |
| peak657 | 2.32 | 0.000218776 | 0.002754229 | 9.29 | 1.31826E-09 | 2.45471E-08 | chr1 | 55207432 | 55207732 | NR_037641.2 | 1.3E-09 | + | 55207432 | 55207732 | 300, |
| peak658 | 2.14 | 0.000102329 | 0.001584893 | 9.71 | 0.000354813 | 0.001380384 | chr1 | 55207430 | 55207781 | NM_001291333.1 | 0.00035 | + | 55207430 | 55207781 | 351, |
| peak659 | 1.85 | 0.00074131 | 0.00676083 | 11.1 | 0.001122018 | 0.004073803 | chr1 | 55207446 | 55207741 | NM_004623.5 | 0.0011 | + | 55207446 | 55207741 | 295, |
| peak682 | 2.99 | 0.002187762 | 0.014454398 | 4.82 | 2.88403E-05 | 0.000141254 | chr1 | 67423790 | 67424570 | NM_001146111.1 | 0.000029 | + | 67423790 | 67424570 | 113,37, |
| peak684 | 4.12 | 0.000194984 | 0.002511886 | 5.4 | 1.94984E-05 | 9.77237E-05 | chr1 | 67423785 | 67424664 | NM_001077702.3 | 0.000019 | + | 67423785 | 67424664 | 118,131, |
| peak689 | 4.63 | 0.000120226 | 0.001737801 | 5.3 | 6.0256E-06 | 3.46737E-05 | chr1 | 67423827 | 67424608 | NM_001146112.2 | 0.000006 | + | 67423827 | 67424608 | 76,75, |
| peak692 | 3.35 | 5.12861E-05 | 0.000977237 | 5.38 | 4.67735E-06 | 2.75423E-05 | chr1 | 67423848 | 67424579 | NM_001350530.2 | 0.0000047 | + | 67423848 | 67424579 | 55,46, |
| peak696 | 4.63 | 0.000120226 | 0.001737801 | 5.3 | 6.45654E-06 | 3.71535E-05 | chr1 | 67423824 | 67424605 | NM_001077701.3 | 0.0000065 | + | 67423824 | 67424605 | 79,72, |
| peak715 | 1.5 | 7.24436E-07 | 3.71535E-05 | 14.4 | 0.000109648 | 0.000467735 | chr1 | 77528792 | 77529142 | NM_001320273.2 | 0.00011 | + | 77528792 | 77529142 | 350, |
| peak835 | 4.6 | 1.07152E-05 | 0.00030903 | 5.83 | 0.00144544 | 0.022387211 | chr1 | 93602347 | 93602546 | NM_007358.4 | 0.0014 | + | 93602347 | 93602546 | 199, |
| peak866 | 5.75 | 0.000660693 | 0.00616595 | 2.12 | 6.0256E-06 | 3.46737E-05 | chr1 | 112999164 | 112999364 | NM_018704.3 | 0.000006 | + | 112999164 | 112999364 | 200, |
| peak869 | 2.97 | 5.7544E-05 | 0.001047129 | 25.2 | 0.001949845 | 0.00676083 | chr1 | 114184745 | 114184895 | NM_001142782.2 | 0.0019 | + | 114184745 | 114184895 | 150, |
| peak889 | 2.13 | 0.000194984 | 0.002511886 | 4.29 | 0.000141254 | 0.002754229 | chr1 | 116234169 | 116234419 | NM_001172411.2 | 0.00014 | + | 116234169 | 116234419 | 250, |
| peak893 | 2.13 | 0.000371535 | 0.004073803 | 5.14 | 4.57088E-05 | 0.001023293 | chr1 | 116234164 | 116234414 | NM_138959.3 | 0.000046 | + | 116234164 | 116234414 | 250, |
| peak898 | 2.19 | 0.000239883 | 0.002884032 | 4 | 5.49541E-05 | 0.001202264 | chr1 | 116234156 | 116234407 | NM_001172412.1 | 0.000055 | + | 116234156 | 116234407 | 251, |
| peak900 | 2.21 | 0.001047129 | 0.008709636 | 4.17 | 3.01995E-05 | 0.000724436 | chr1 | 117617634 | 117617835 | NM_003594.4 | 0.00003 | + | 117617634 | 117617835 | 201, |
| peak928 | 1.22 | 0.002570396 | 0.016218101 | 15.5 | 0.001659587 | 0.005888437 | chr1 | 154321589 | 154321739 | NM_001370597.1 | 0.0017 | + | 154321589 | 154321739 | 150, |
| peak968 | 2.29 | 0.001258925 | 0.009772372 | 23.2 | 5.01187E-13 | 2.51189E-11 | chr1 | 161882118 | 161928681 | NM_007348.4 | 5E-13 | + | 161882118 | 161928681 | 52,446, |
| peak1000 | 1.63 | 0.004677351 | 0.024547089 | 4.51 | 7.94328E-05 | 0.000346737 | chr1 | 167757126 | 167757326 | NM_024569.5 | 0.000079 | + | 167757126 | 167757326 | 200, |
| peak1008 | 1.32 | 8.12831E-06 | 0.000245471 | 13.9 | 4.67735E-08 | 5.12861E-07 | chr1 | 170705194 | 170705591 | NM_022716.4 | 0.000000047 | + | 170705194 | 170705591 | 397, |
| peak1032 | 2.46 | 0.000218776 | 0.002754229 | 80.8 | 2.0893E-07 | 1.8197E-06 | chr1 | 173855002 | 173855403 | NM_001346115.2 | 0.00000021 | + | 173855002 | 173855403 | 401, |
| peak1033 | 2.54 | 0.00017378 | 0.002290868 | 88.3 | 1.23027E-07 | 1.1749E-06 | chr1 | 173855000 | 173855400 | NM_001369846.1 | 0.00000012 | + | 173855000 | 173855400 | 400, |

| | | | | | | | | | | | | | | | |
|---|---|---|---|---|---|---|---|---|---|---|---|---|---|---|---|
| peak1034 | 2.54 | 0.000177828 | 0.002344229 | 88.5 | 2.95121E-07 | 2.45471E-06 | chr1 | 173855001 | 173855401 | NM_001122770.3 | 0.0000003 | + | 173855001 | 173855401 | 400, |
| peak1035 | 2.3 | 4.7863E-05 | 0.000933254 | 83.7 | 1.8197E-07 | 1.62181E-06 | chr1 | 173854962 | 173855462 | NM_032522.5 | 0.00000018 | + | 173854962 | 173855462 | 500, |
| peak1081 | 1.85 | 0.003630781 | 0.020417379 | 20.9 | 8.31764E-09 | 1.1749E-07 | chr1 | 180080269 | 180080569 | NM_014810.5 | 8.3E-09 | + | 180080269 | 180080569 | 300, |
| peak1170 | 3.88 | 3.63078E-05 | 0.000758578 | 2.6 | 3.63078E-05 | 0.00017378 | chr1 | 201847038 | 201847138 | NM_018085.5 | 0.000036 | + | 201847038 | 201847138 | 100, |
| peak1171 | 3.32 | 6.91831E-06 | 0.000218776 | 5.8 | 3.63078E-05 | 0.000851138 | chr1 | 201847436 | 201847637 | NM_018085.5 | 0.000036 | + | 201847436 | 201847637 | 201, |
| peak1178 | 3.61 | 7.76247E-10 | 7.24436E-08 | 7.1 | 2.51189E-05 | 0.000123027 | chr1 | 203708724 | 203709173 | NM_001001396.2 | 0.000025 | + | 203708724 | 203709173 | 449, |
| peak1235 | 1.18 | 0.000676083 | 0.006309573 | 31.2 | 1.23027E-06 | 8.51138E-06 | chr1 | 204518437 | 204518787 | NM_001278519.1 | 0.0000012 | + | 204518437 | 204518787 | 350, |
| peak1240 | 2.74 | 0.003715352 | 0.020892961 | 2.5 | 0.000204174 | 0.003890451 | chr1 | 210024624 | 210024725 | NM_014388.7 | 0.0002 | + | 210024624 | 210024725 | 101, |
| peak1300 | 1.29 | 0.002187762 | 0.014454398 | 8 | 0.00030903 | 0.001230269 | chr1 | 222817694 | 222819010 | NM_001300867.1 | 0.00031 | + | 222817694 | 222819010 | 234,115, |
| peak1305 | 1.37 | 0.001148154 | 0.009120108 | 6.75 | 0.000851138 | 0.003090295 | chr1 | 222817694 | 222819012 | NM_001324065.1 | 0.00085 | + | 222817694 | 222819012 | 234,117, |
| peak1329 | 3.54 | 0.00011749 | 0.001737801 | 4.5 | 5.24807E-05 | 0.001174898 | chr1 | 227842139 | 227842289 | NM_001367911.1 | 0.000052 | + | 227842139 | 227842289 | 150, |
| peak1352 | 2.47 | 1.07152E-05 | 0.000301995 | 5.25 | 0.001047129 | 0.003801894 | chr1 | 228547852 | 228548801 | NM_052843.4 | 0.001 | + | 228547852 | 228548801 | 949, |
| peak1363 | 4.19 | 8.12831E-05 | 0.001348963 | 7.44 | 4.0738E-09 | 6.30957E-08 | chr1 | 229772467 | 229772567 | NM_014777.4 | 4.1E-09 | + | 229772467 | 229772567 | 100, |
| peak1365 | 3.64 | 0.000138038 | 0.001949845 | 11.4 | 2.95121E-06 | 1.86209E-05 | chr1 | 229773756 | 229779289 | NM_014777.4 | 0.000003 | + | 229773756 | 229779289 | 238,10, |
| peak1374 | 2.34 | 0.001047129 | 0.00851138 | 7.3 | 0.007079458 | 0.023442288 | chr1 | 231829921 | 231830021 | NM_001164554.2 | 0.0071 | + | 231829921 | 231830021 | 100, |
| peak1376 | 1.92 | 0.002398833 | 0.015488166 | 6.67 | 0.00128825 | 0.004570882 | chr1 | 231829918 | 231830067 | NM_001164550.2 | 0.0013 | + | 231829918 | 231830067 | 149, |
| peak1377 | 1.92 | 0.002398833 | 0.015488166 | 6.67 | 0.001348963 | 0.004897788 | chr1 | 231829918 | 231830067 | NM_001164552.2 | 0.0013 | + | 231829918 | 231830067 | 149, |
| peak1404 | 1.25 | 0.004786301 | 0.025118864 | 9.6 | 8.12831E-07 | 5.88844E-06 | chr1 | 246754909 | 246784796 | NM_001139459.1 | 0.00000081 | + | 246754909 | 246784796 | 334,66, |
| peak1424 | 2.43 | 0.001122018 | 0.008912509 | 23.7 | 0.008709636 | 0.028840315 | chr1 | 1365734 | 1365884 | NR_125996.1 | 0.0087 | - | 1365734 | 1365884 | 150, |
| peak1502 | 3.51 | 7.4131E-06 | 0.000234423 | 2.5 | 0.000234423 | 0.000933254 | chr1 | 19234908 | 19235109 | NM_001136265.2 | 0.00023 | - | 19234908 | 19235109 | 201, |
| peak1525 | 2.51 | 0.001348963 | 0.01023293 | 22.4 | 6.30957E-05 | 0.000281838 | chr1 | 21231463 | 21268481 | NM_001198802.2 | 0.000063 | - | 21231463 | 21268481 | 1,498, |
| peak1531 | 3.35 | 0.002884032 | 0.017782794 | 1.92 | 0.003801894 | 0.012882496 | chr1 | 21545583 | 21545683 | NM_001113349.2 | 0.0038 | - | 21545583 | 21545683 | 100, |
| peak1536 | 1.03 | 0.00057544 | 0.005495409 | 14.5 | 3.54813E-05 | 0.000165959 | chr1 | 23414302 | 23414801 | NM_001142546.1 | 0.000035 | - | 23414302 | 23414801 | 499, |
| peak1540 | 1.17 | 5.88844E-05 | 0.001071519 | 12.5 | 2.95121E-05 | 0.000141254 | chr1 | 23414312 | 23414811 | NM_033631.4 | 0.00003 | - | 23414312 | 23414811 | 499, |
| peak1574 | 1.22 | 0.001096478 | 0.008912509 | 3.62 | 2.69153E-08 | 3.23594E-07 | chr1 | 27212584 | 27216377 | NM_018066.4 | 0.000000027 | - | 27212584 | 27216377 | 40,157,201, |
| peak1595 | 1.9 | 0.003630781 | 0.020417379 | 2.01 | 1.69824E-06 | 1.14815E-05 | chr1 | 33478861 | 33478961 | NR_134976.2 | 0.0000017 | - | 33478861 | 33478961 | 100, |
| peak1598 | 2.19 | 0.004786301 | 0.025118864 | 2.62 | 2.13796E-06 | 1.41254E-05 | chr1 | 33478858 | 33478958 | NM_001319140.1 | 0.0000021 | - | 33478858 | 33478958 | 100, |
| peak1612 | 1.45 | 0.000489779 | 0.004897788 | 3.64 | 0.000891251 | 0.003311311 | chr1 | 36602652 | 36602798 | NM_001270895.1 | 0.00089 | - | 36602652 | 36602798 | 146, |
| peak1625 | 3.41 | 0.004570882 | 0.024547089 | 7 | 3.71535E-05 | 0.000851138 | chr1 | 40091293 | 40091791 | NM_014571.4 | 0.000037 | - | 40091293 | 40091791 | 498, |
| peak1654 | 2.45 | 0.002089296 | 0.014125375 | 3.08 | 0.001047129 | 0.003801894 | chr1 | 52520804 | 52520953 | NR_046406.1 | 0.001 | - | 52520804 | 52520953 | 149, |
| peak1673 | 1.5 | 0.004265795 | 0.023442288 | 2.57 | 3.98107E-13 | 2.51189E-11 | chr1 | 67878772 | 67878923 | NM_001018067.2 | 4E-13 | - | 67878772 | 67878923 | 151, |
| peak1675 | 1.55 | 0.001318257 | 0.01 | 2.69 | 3.98107E-13 | 2.51189E-11 | chr1 | 67878758 | 67878908 | NM_001018068.2 | 4E-13 | - | 67878758 | 67878908 | 150, |
| peak1679 | 1.6 | 0.001023293 | 0.008317638 | 2.69 | 1E-13 | 6.30957E-12 | chr1 | 67878762 | 67878912 | NM_015640.4 | 1E-13 | - | 67878762 | 67878912 | 150, |
| peak1686 | 1.46 | 0.003090295 | 0.018620871 | 8.86 | 1.69824E-07 | 1.54882E-06 | chr1 | 78097576 | 78098074 | NM_015534.6 | 0.00000017 | - | 78097576 | 78098074 | 498, |
| peak1702 | 3.72 | 0.000831764 | 0.00724436 | 17.2 | 0.000107152 | 0.000467735 | chr1 | 86816955 | 86817305 | NM_001366783.1 | 0.00011 | - | 86816955 | 86817305 | 350, |
| peak1706 | 3.88 | 0.001479108 | 0.010964782 | 16.4 | 7.94328E-05 | 0.000346737 | chr1 | 86816949 | 86817299 | NM_020729.3 | 0.000079 | - | 86816949 | 86817299 | 350, |
| peak1766 | 2.51 | 0.000660693 | 0.00616595 | 14.5 | 1.20226E-07 | 1.12202E-06 | chr1 | 113459967 | 113460366 | NM_001166496.1 | 0.00000012 | - | 113459967 | 113460366 | 399, |
| peak1768 | 2.57 | 0.001698244 | 0.012302688 | 11.5 | 1.31826E-07 | 1.23027E-06 | chr1 | 113459905 | 113460202 | NM_003051.3 | 0.00000013 | - | 113459905 | 113460202 | 297, |
| peak1784 | 3.8 | 0.002187762 | 0.014454398 | 4.44 | 0.000112202 | 0.00047863 | chr1 | 117142692 | 117142942 | NM_001007237.3 | 0.00011 | - | 117142692 | 117142942 | 250, |
| peak1939 | 2.92 | 0.000239883 | 0.002884032 | 3.19 | 7.07946E-06 | 3.98107E-05 | chr1 | 160187194 | 160187344 | NM_015726.4 | 0.0000071 | - | 160187194 | 160187344 | 150, |
| peak1968 | 2.28 | 0.00011749 | 0.001737801 | 3.5 | 0.000512861 | 0.001949845 | chr1 | 161019011 | 161019299 | NM_001287602.2 | 0.00051 | - | 161019011 | 161019299 | 113,86, |
| peak1974 | 3.46 | 0.000933254 | 0.007943282 | 3 | 0.000190546 | 0.000776247 | chr1 | 161021157 | 161021505 | NM_181720.3 | 0.00019 | - | 161021157 | 161021505 | 348, |
| peak2051 | 3.24 | 4.7863E-07 | 2.63027E-05 | 7.78 | 4.36516E-06 | 2.63027E-05 | chr1 | 179076981 | 179077580 | NM_001168236.2 | 0.0000044 | - | 179076981 | 179077580 | 599, |
| peak2095 | 1.07 | 0.003162278 | 0.018620871 | 23 | 1E-10 | 2.5704E-09 | chr1 | 179834536 | 179834886 | NM_022347.5 | 1E-10 | - | 179834536 | 179834886 | 350, |
| peak2102 | 4.22 | 0.001258925 | 0.009772372 | 2.48 | 1.51356E-06 | 1.02329E-05 | chr1 | 186303520 | 186304255 | NM_003292.3 | 0.0000015 | - | 186303520 | 186304255 | 145,56, |
| peak2129 | 1.03 | 0.000524807 | 0.005128614 | 5.5 | 1.04713E-05 | 5.62341E-05 | chr1 | 197168576 | 197168926 | NR_135153.1 | 0.00001 | - | 197168576 | 197168926 | 350, |
| peak2138 | 1.33 | 0.003715352 | 0.020892961 | 32.7 | 6.30957E-12 | 2.29087E-10 | chr1 | 204379451 | 204380746 | NM_032833.4 | 6.3E-12 | - | 204379451 | 204380746 | 1295, |
| peak2140 | 5.47 | 1.07152E-05 | 0.00030903 | 4.4 | 0.003235937 | 0.046773514 | chr1 | 204586842 | 204586991 | NM_201630.2 | 0.0032 | - | 204586842 | 204586991 | 149, |
| peak2178 | 1.34 | 0.00011749 | 0.001737801 | 6.5 | 4.0738E-05 | 0.000190546 | chr1 | 212115238 | 212115439 | NM_001199811.1 | 0.000041 | - | 212115238 | 212115439 | 201, |
| peak2179 | 1.2 | 0.000346737 | 0.003801894 | 6.17 | 2.18776E-07 | 1.90546E-06 | chr1 | 212115232 | 212115432 | NM_001199812.1 | 0.00000022 | - | 212115232 | 212115432 | 200, |
| peak2180 | 1.2 | 0.000301995 | 0.003388442 | 6.17 | 8.91251E-05 | 0.000389045 | chr1 | 212115230 | 212115430 | NR_037667.1 | 0.000089 | - | 212115230 | 212115430 | 200, |
| peak2181 | 1.23 | 0.000281838 | 0.003311311 | 6.7 | 5.01187E-05 | 0.000229087 | chr1 | 212115237 | 212115437 | NM_015434.4 | 0.00005 | - | 212115237 | 212115437 | 200, |
| peak2182 | 3.18 | 0.000114815 | 0.001698244 | 16.5 | 0.000562341 | 0.002137962 | chr1 | 212911743 | 212911944 | NM_001042549.2 | 0.00056 | - | 212911743 | 212911944 | 201, |
| peak2183 | 3.14 | 0.000338844 | 0.003801894 | 16.5 | 8.51138E-05 | 0.000371535 | chr1 | 212911785 | 212911936 | NM_001297736.2 | 0.000085 | - | 212911785 | 212911936 | 151, |
| peak2184 | 3.38 | 0.000389045 | 0.004168694 | 16.5 | 0.000363078 | 0.001412538 | chr1 | 212911790 | 212911941 | NM_001297737.2 | 0.00036 | - | 212911790 | 212911941 | 151, |
| peak2185 | 3.21 | 0.000223872 | 0.002754229 | 16.9 | 2.0893E-10 | 5.12861E-09 | chr1 | 212911760 | 212911961 | NM_015471.4 | 2.1E-10 | - | 212911760 | 212911961 | 201, |
| peak2188 | 4.48 | 0.000186209 | 0.002454709 | 57.5 | 3.80189E-08 | 4.2658E-07 | chr1 | 217604503 | 217622635 | NM_018040.5 | 0.000000038 | - | 217604503 | 217622635 | 204,45, |

| | | | | | | | | | | | | | | | |
|---|---|---|---|---|---|---|---|---|---|---|---|---|---|---|---|
| peak2192 | 1.68 | 0.00144544 | 0.010715193 | 8.24 | 1E-13 | 7.94328E-12 | chr1 | 222843089 | 222849500 | NM_022831.4 | 1E-13 | - | 222843089 | 222849500 | 242,118,123,63, |
| peak2266 | 2.28 | 8.12831E-06 | 0.000251189 | 8 | 0.000645654 | 0.002398833 | chr1 | 235377241 | 235383690 | NR_135074.2 | 0.00065 | - | 235377241 | 235383690 | 100,176,74, |
| peak2270 | 3.64 | 4.67735E-06 | 0.000162181 | 17.5 | 0.001148154 | 0.004168694 | chr1 | 235973337 | 235973637 | NM_001301365.1 | 0.0011 | - | 235973337 | 235973637 | 300, |
| peak2273 | 2 | 0.004570882 | 0.024547089 | 3.67 | 0.000616595 | 0.01023293 | chr1 | 235972482 | 235972682 | NM_000081.4 | 0.00062 | - | 235972482 | 235972682 | 200, |
| peak2274 | 3.61 | 2.23872E-05 | 0.000537032 | 12.3 | 0.000616595 | 0.002290868 | chr1 | 235973329 | 235973580 | NM_000081.4 | 0.00062 | - | 235973329 | 235973580 | 251, |
| peak2290 | 1.85 | 0.000104713 | 0.001584893 | 17.7 | 0.003467369 | 0.011748976 | chr1 | 247014384 | 247014534 | NM_015446.5 | 0.0035 | - | 247014384 | 247014534 | 150, |
| peak2294 | 1.31 | 0.003235937 | 0.019054607 | 5.8 | 0.000141254 | 0.002754229 | chr1 | 247263902 | 247264149 | NM_001142572.1 | 0.00014 | - | 247263902 | 247264149 | 247, |
| peak2296 | 1.31 | 0.003235937 | 0.019054607 | 5.8 | 2.51189E-05 | 0.000616595 | chr1 | 247263906 | 247264155 | NM_024804.2 | 0.000025 | - | 247263906 | 247264155 | 249, |
| peak2402 | 1.08 | 0.002818383 | 0.017378008 | 2.44 | 4.46684E-05 | 0.00020893 | chr2 | 54853331 | 54856125 | NM_003128.3 | 0.000045 | + | 54853331 | 54856125 | 40,154,56, |
| peak2420 | 3.66 | 6.91831E-05 | 0.001202264 | 5.29 | 3.98107E-11 | 1.23027E-09 | chr2 | 58366953 | 58386510 | NM_001288838.1 | 4E-11 | + | 58366953 | 58386510 | 14,159,49,27, |
| peak2439 | 2.29 | 0.003235937 | 0.019054607 | 4.5 | 6.0256E-06 | 0.000177828 | chr2 | 64779261 | 64779510 | NM_203437.3 | 0.000006 | + | 64779261 | 64779510 | 249, |
| peak2441 | 1.04 | 0.000169824 | 0.002238721 | 3.77 | 6.0256E-06 | 3.46737E-05 | chr2 | 64780402 | 64794804 | NM_203437.3 | 0.000006 | + | 64780402 | 64794804 | 141,109, |
| peak2444 | 2.86 | 0.000645654 | 0.006025596 | 1.86 | 1.34896E-06 | 5.12861E-05 | chr2 | 64778638 | 64778738 | NM_001002243.3 | 0.0000013 | + | 64778638 | 64778738 | 100, |
| peak2446 | 2.61 | 0.001348963 | 0.01023293 | 5.5 | 1.34896E-06 | 5.12861E-05 | chr2 | 64779232 | 64779481 | NM_001002243.3 | 0.0000013 | + | 64779232 | 64779481 | 249, |
| peak2448 | 5.5 | 1.51356E-05 | 0.000398107 | 6.25 | 1.34896E-06 | 5.12861E-05 | chr2 | 64779876 | 64780026 | NM_001002243.3 | 0.0000013 | + | 64779876 | 64780026 | 150, |
| peak2449 | 1.45 | 2.75423E-05 | 0.000630957 | 3.38 | 1.34896E-06 | 9.33254E-06 | chr2 | 64780372 | 64794822 | NM_001002243.3 | 0.0000013 | + | 64780372 | 64794822 | 171,127, |
| peak2453 | 2.53 | 0.001230269 | 0.009549926 | 5.5 | 0.000323594 | 0.005754399 | chr2 | 64779233 | 64779482 | NM_017657.5 | 0.00032 | + | 64779233 | 64779482 | 249, |
| peak2455 | 5.52 | 1.7378E-05 | 0.000446684 | 6.25 | 0.000323594 | 0.005754399 | chr2 | 64779878 | 64780027 | NM_017657.5 | 0.00032 | + | 64779878 | 64780027 | 149, |
| peak2469 | 1.47 | 0.003801894 | 0.021379621 | 19.1 | 3.23594E-07 | 2.69153E-06 | chr2 | 68401875 | 68402075 | NM_020143.4 | 0.00000032 | + | 68401875 | 68402075 | 200, |
| peak2485 | 1.61 | 0.000457088 | 0.004677351 | 5.8 | 0.000891251 | 0.003311311 | chr2 | 71360152 | 71360694 | NM_005791.3 | 0.00089 | + | 71360152 | 71360694 | 542, |
| peak2504 | 2.87 | 0.00030903 | 0.003548134 | 13.4 | 0.01 | 0.032359366 | chr2 | 73799672 | 73800022 | NM_015120.4 | 0.01 | + | 73799672 | 73800022 | 350, |
| peak2505 | 1.49 | 0.001023293 | 0.008317638 | 16.2 | 0.01 | 0.032359366 | chr2 | 73800121 | 73800371 | NM_015120.4 | 0.01 | + | 73800121 | 73800371 | 250, |
| peak2526 | 1.92 | 0.000107152 | 0.00162181 | 8.11 | 3.98107E-05 | 0.000186209 | chr2 | 74783581 | 74784030 | NM_001318868.1 | 0.00004 | + | 74783581 | 74784030 | 449, |
| peak2527 | 1.94 | 9.12011E-05 | 0.00144544 | 9.55 | 0.000123027 | 0.000524807 | chr2 | 74783584 | 74784032 | NM_001318869.1 | 0.00012 | + | 74783584 | 74784032 | 448, |
| peak2536 | 2.23 | 8.31764E-06 | 0.000251189 | 6.75 | 2.63027E-08 | 1.8197E-06 | chr2 | 88874229 | 88874829 | NR_110236.1 | 0.000000026 | + | 88874229 | 88874829 | 600, |
| peak2537 | 2.09 | 0.000338844 | 0.003801894 | 31.9 | 2.63027E-10 | 6.30957E-09 | chr2 | 96936984 | 96937283 | NM_004804.3 | 2.6E-10 | + | 96936984 | 96937283 | 299, |
| peak2541 | 1.78 | 0.000275423 | 0.003235937 | 32 | 7.76247E-08 | 7.76247E-07 | chr2 | 97911101 | 97911302 | NM_001354587.1 | 0.000000078 | + | 97911101 | 97911302 | 201, |
| peak2637 | 3.61 | 0.001412538 | 0.010471285 | 12.4 | 2.5704E-09 | 4.2658E-08 | chr2 | 132278102 | 132278302 | NR_026922.1 | 2.6E-09 | + | 132278102 | 132278302 | 200, |
| peak2641 | 1.67 | 2.18776E-05 | 0.000524807 | 20.3 | 1.99526E-10 | 4.89779E-09 | chr2 | 135206304 | 135206705 | NM_002410.5 | 2E-10 | + | 135206304 | 135206705 | 401, |
| peak2673 | 3.63 | 0.001949845 | 0.013489629 | 5.8 | 1.38038E-06 | 9.54993E-06 | chr2 | 149542341 | 149543944 | NM_015630.4 | 0.0000014 | + | 149542341 | 149543944 | 229,71, |
| peak2676 | 2.05 | 0.000891251 | 0.007762471 | 12.3 | 0.005754399 | 0.019054607 | chr2 | 152321747 | 152321947 | NM_001177665.2 | 0.0058 | + | 152321747 | 152321947 | 200, |
| peak2677 | 1.99 | 0.002137962 | 0.014125375 | 16.5 | 0.005754399 | 0.019054607 | chr2 | 152322146 | 152322546 | NM_001177665.2 | 0.0058 | + | 152322146 | 152322546 | 400, |
| peak2679 | 1.57 | 0.001737801 | 0.012302688 | 6.3 | 0.002041738 | 0.00724436 | chr2 | 153473656 | 153475509 | NM_052905.4 | 0.002 | + | 153473656 | 153475509 | 50,150, |
| peak2699 | 3.78 | 0.000524807 | 0.005248075 | 2.5 | 8.51138E-09 | 1.20226E-07 | chr2 | 162091894 | 162091995 | NM_004180.3 | 8.5E-09 | + | 162091894 | 162091995 | 101, |
| peak2762 | 3.72 | 2.75423E-05 | 0.000630957 | 34.2 | 1.25893E-07 | 1.1749E-06 | chr2 | 196545296 | 196545396 | NM_020342.3 | 0.00000013 | + | 196545296 | 196545396 | 100, |
| peak3014 | 5.51 | 0.002691535 | 0.016982437 | 5.43 | 8.70964E-07 | 6.45654E-06 | chr2 | 207631998 | 207632099 | NM_014929.3 | 0.00000087 | + | 207631998 | 207632099 | 101, |
| peak3017 | 5.4 | 4.57088E-06 | 0.000158489 | 4.42 | 1.69824E-06 | 1.14815E-05 | chr2 | 207631921 | 207632121 | NM_001136193.2 | 0.0000017 | + | 207631921 | 207632121 | 200, |
| peak3069 | 1.16 | 4.16869E-05 | 0.000851138 | 4.62 | 1.69824E-08 | 2.13796E-07 | chr2 | 233659501 | 233660869 | NM_001103146.2 | 0.000000017 | + | 233659501 | 233660869 | 153,98, |
| peak3074 | 1.18 | 1.23027E-05 | 0.000338844 | 6.2 | 4.0738E-07 | 3.23594E-06 | chr2 | 233659465 | 233671201 | NM_001103147.2 | 0.00000041 | + | 233659465 | 233671201 | 189,160,1, |
| peak3080 | 1.37 | 5.88844E-06 | 0.000190546 | 5.6 | 2.18776E-07 | 1.90546E-06 | chr2 | 233659480 | 233671214 | NM_001103148.2 | 0.00000022 | + | 233659480 | 233671214 | 174,160,14, |
| peak3085 | 1.26 | 6.91831E-06 | 0.000218776 | 3.56 | 7.94328E-09 | 1.12202E-07 | chr2 | 233659459 | 233660926 | NM_015575.4 | 7.9E-09 | + | 233659459 | 233660926 | 195,155, |
| peak3114 | 1.39 | 0.000346737 | 0.003801894 | 6.72 | 0.001949845 | 0.00676083 | chr2 | 237478516 | 237489474 | NM_020311.3 | 0.0019 | + | 237478516 | 237489474 | 51,392, |
| peak3118 | 1.67 | 0.003890451 | 0.021877616 | 6.66 | 5.37032E-06 | 3.0903E-05 | chr2 | 238668778 | 238688141 | NM_001137550.2 | 0.0000054 | + | 238668778 | 238688141 | 70,50,118,80,105,77, |
| peak3121 | 2.12 | 3.63078E-05 | 0.000758578 | 14.5 | 8.91251E-06 | 4.89779E-05 | chr2 | 238662107 | 238671541 | NM_001137552.2 | 0.0000089 | + | 238662107 | 238671541 | 15,93,142,296, |
| peak3210 | 5.83 | 0.00047863 | 0.004897788 | 8.37 | 0.00162181 | 0.005754399 | chr2 | 24261710 | 24261910 | NM_025203.3 | 0.0016 | - | 24261710 | 24261910 | 200, |
| peak3249 | 1.03 | 0.000457088 | 0.004677351 | 15.8 | 4.57088E-08 | 5.01187E-07 | chr2 | 25965370 | 25966520 | NM_018263.6 | 0.000000046 | - | 25965370 | 25966520 | 1150, |
| peak3271 | 3 | 0.00074131 | 0.00676083 | 10.1 | 0.000831764 | 0.003019952 | chr2 | 37873632 | 37873732 | NM_006449.5 | 0.00083 | - | 37873632 | 37873732 | 100, |
| peak3276 | 3.6 | 0.003630781 | 0.020892961 | 11.2 | 0.00057544 | 0.002187762 | chr2 | 37873652 | 37873753 | NM_001270438.2 | 0.00058 | - | 37873652 | 37873753 | 101, |
| peak3301 | 2.61 | 4.46684E-06 | 0.000154882 | 11 | 0.000141254 | 0.000588844 | chr2 | 40656046 | 40656446 | NM_001252624.2 | 0.00014 | - | 40656046 | 40656446 | 400, |
| peak3305 | 3.02 | 1.41254E-06 | 6.16595E-05 | 9 | 2.23872E-06 | 1.44544E-05 | chr2 | 40656070 | 40656421 | NM_001351485.2 | 0.0000022 | - | 40656070 | 40656421 | 351, |
| peak3312 | 3.02 | 1.41254E-06 | 6.16595E-05 | 9 | 5.62341E-05 | 0.00025704 | chr2 | 40656070 | 40656421 | NM_001351489.2 | 0.000056 | - | 40656070 | 40656421 | 351, |
| peak3315 | 2.89 | 4.0738E-06 | 0.000144544 | 11.3 | 0.000436516 | 0.001659587 | chr2 | 40656065 | 40656466 | NM_001351492.2 | 0.00044 | - | 40656065 | 40656466 | 401, |
| peak3321 | 2.46 | 1.62181E-05 | 0.00042658 | 9 | 0.00001 | 5.37032E-05 | chr2 | 40656029 | 40656429 | NM_001351483.2 | 0.00001 | - | 40656029 | 40656429 | 400, |
| peak3333 | 3.16 | 1.65959E-06 | 7.07946E-05 | 9 | 1.1749E-06 | 8.31764E-06 | chr2 | 40656030 | 40656430 | NM_001351490.2 | 0.0000012 | - | 40656030 | 40656430 | 400, |
| peak3368 | 1.01 | 4.2658E-07 | 2.39883E-05 | 5.52 | 3.01995E-06 | 1.90546E-05 | chr2 | 61415146 | 61416045 | NM_014709.4 | 0.000003 | - | 61415146 | 61416045 | 698,1, |
| peak3383 | 2.71 | 5.88844E-05 | 0.001071519 | 6 | 8.91251E-06 | 4.89779E-05 | chr2 | 69692774 | 69692975 | NM_001371575.1 | 0.0000089 | - | 69692774 | 69692975 | 201, |
| peak3389 | 3.26 | 8.31764E-05 | 0.001348963 | 5.89 | 5.62341E-06 | 3.23594E-05 | chr2 | 69692812 | 69692963 | NM_014911.4 | 0.0000056 | - | 69692812 | 69692963 | 151, |

| | | | | | | | | | | | | | | | |
|---|---|---|---|---|---|---|---|---|---|---|---|---|---|---|---|
| peak3401 | 4.17 | 5.24807E-05 | 0.001 | 1.71 | 0.000144544 | 0.000616595 | chr2 | 73315773 | 73316101 | NM_015470.3 | 0.00014 | - | 73315773 | 73316101 | 104,95, |
| peak3420 | 2.98 | 0.001819701 | 0.012882496 | 19.7 | 1.99526E-08 | 2.45471E-07 | chr2 | 85771941 | 85772141 | NM_000821.7 | 0.00000002 | - | 85771941 | 85772141 | 200, |
| peak3421 | 3.01 | 0.001148154 | 0.009120108 | 22 | 2.63027E-08 | 3.0903E-07 | chr2 | 85771942 | 85772142 | NM_001142269.4 | 0.000000026 | - | 85771942 | 85772142 | 200, |
| peak3516 | 4.63 | 0.003715352 | 0.020892961 | 7.14 | 4.46684E-05 | 0.00020893 | chr2 | 99342715 | 99347595 | NM_012214.3 | 0.000045 | - | 99342715 | 99347595 | 315,85, |
| peak3546 | 2.67 | 0.00074131 | 0.00676083 | 3.73 | 9.77237E-10 | 1.86209E-08 | chr2 | 106710553 | 106715206 | NM_001253876.1 | 9.8E-10 | - | 106710553 | 106715206 | 60,104,35, |
| peak3547 | 2.76 | 0.000346737 | 0.003890451 | 3.83 | 5.7544E-05 | 0.00025704 | chr2 | 106710552 | 106715205 | NM_025076.4 | 0.000058 | - | 106710552 | 106715205 | 61,104,34, |
| peak3566 | 3.65 | 0.0001 | 0.001548817 | 2 | 0.000138038 | 0.000588844 | chr2 | 110843023 | 110843122 | NM_005434.5 | 0.00014 | - | 110843023 | 110843122 | 99, |
| peak3568 | 3.75 | 7.4131E-05 | 0.001258925 | 2.2 | 0.000158489 | 0.000660693 | chr2 | 110843015 | 110843114 | NM_001371559.1 | 0.00016 | - | 110843015 | 110843114 | 99, |
| peak3570 | 3.4 | 5.37032E-05 | 0.001 | 9.25 | 0.0001 | 0.000436516 | chr2 | 111419185 | 111419384 | NM_001278616.1 | 0.0001 | - | 111419185 | 111419384 | 199, |
| peak3575 | 2.05 | 0.003630781 | 0.020892961 | 7.2 | 0.000416869 | 0.001584893 | chr2 | 112101780 | 112252538 | NR_136164.1 | 0.00042 | - | 112101780 | 112252538 | 34,89,77, |
| peak3578 | 4.6 | 3.98107E-05 | 0.000812831 | 7.62 | 0.002884032 | 0.01 | chr2 | 118677444 | 118677595 | NM_019044.5 | 0.0029 | - | 118677444 | 118677595 | 151, |
| peak3594 | 2.26 | 5.37032E-05 | 0.001023293 | 4.11 | 0.000316228 | 0.001230269 | chr2 | 128065268 | 128066199 | NM_006609.5 | 0.00032 | - | 128065268 | 128066199 | 112,39, |
| peak3598 | 2.07 | 0.004265795 | 0.022908677 | 3.35 | 0.000354813 | 0.001380384 | chr2 | 128065205 | 128066235 | NM_001371911.1 | 0.00035 | - | 128065205 | 128066235 | 175,75, |
| peak3608 | 2.59 | 0.004365158 | 0.023988329 | 7.56 | 5.49541E-05 | 0.000251189 | chr2 | 128628922 | 128631739 | NM_001199140.1 | 0.000055 | - | 128628922 | 128631739 | 11,338, |
| peak3628 | 2.66 | 0.000436516 | 0.004466836 | 20 | 1.04713E-05 | 5.62341E-05 | chr2 | 136873159 | 136873259 | NM_001008540.2 | 0.00001 | - | 136873159 | 136873259 | 100, |
| peak3629 | 2.72 | 0.000489779 | 0.004897788 | 21.9 | 1.38038E-05 | 7.24436E-05 | chr2 | 136873155 | 136873255 | NM_001348056.2 | 0.000014 | - | 136873155 | 136873255 | 100, |
| peak3631 | 1.95 | 0.001230269 | 0.009549926 | 42.8 | 6.0256E-08 | 6.30957E-07 | chr2 | 145156432 | 145156783 | NM_001171653.2 | 0.00000006 | - | 145156432 | 145156783 | 351, |
| peak3657 | 2.76 | 0.000269153 | 0.003162278 | 2.5 | 1.90546E-07 | 1.69824E-06 | chr2 | 152663367 | 152668901 | NM_001037174.2 | 0.00000019 | - | 152663367 | 152668901 | 119,31, |
| peak3658 | 2.81 | 0.000151356 | 0.002089296 | 2.64 | 2.0893E-07 | 1.8197E-06 | chr2 | 152663365 | 152668899 | NM_177985.3 | 0.00000021 | - | 152663365 | 152668899 | 121,29, |
| peak3741 | 4.25 | 0.004897788 | 0.025703958 | 2.81 | 3.98107E-08 | 4.36516E-07 | chr2 | 165561530 | 165578599 | NM_001365672.2 | 0.00000004 | - | 165561530 | 165578599 | 85,15, |
| peak3765 | 4.26 | 0.000933254 | 0.007943282 | 2.32 | 4.16869E-08 | 4.67735E-07 | chr2 | 165561535 | 165578604 | NM_001278460.2 | 0.000000042 | - | 165561535 | 165578604 | 80,20, |
| peak3772 | 4.27 | 0.000831764 | 0.00724436 | 2.11 | 3.80189E-08 | 4.2658E-07 | chr2 | 165561535 | 165578605 | NM_001365670.2 | 0.000000038 | - | 165561535 | 165578605 | 80,21, |
| peak3824 | 2.37 | 0.002238721 | 0.014454398 | 56.7 | 1.7378E-06 | 1.14815E-05 | chr2 | 190428581 | 190430154 | NM_014585.5 | 0.0000017 | - | 190428581 | 190430154 | 370,75, |
| peak3864 | 6.82 | 0.001862087 | 0.012882496 | 2.57 | 6.91831E-09 | 5.88844E-07 | chr2 | 211019149 | 211019249 | NM_001307976.2 | 6.9E-09 | - | 211019149 | 211019249 | 100, |
| peak3866 | 6.82 | 0.001548817 | 0.011220185 | 2.55 | 1.07152E-08 | 8.51138E-07 | chr2 | 211019147 | 211019247 | NM_152519.4 | 0.000000011 | - | 211019147 | 211019247 | 100, |
| peak3877 | 1.08 | 0.000812831 | 0.007079458 | 22.3 | 1.99526E-11 | 6.76083E-10 | chr2 | 213872061 | 213872712 | NM_001371274.1 | 2E-11 | - | 213872061 | 213872712 | 651, |
| peak3895 | 2.49 | 0.000107152 | 0.00162181 | 12 | 1.58489E-07 | 1.44544E-06 | chr2 | 219502891 | 219503192 | NM_001366290.3 | 0.00000016 | - | 219502891 | 219503192 | 301, |
| peak3900 | 2.3 | 0.002818383 | 0.017378008 | 10.3 | 0.000316228 | 0.001230269 | chr2 | 219502909 | 219503209 | NM_001105537.4 | 0.00032 | - | 219502909 | 219503209 | 300, |
| peak3907 | 4.08 | 3.89045E-06 | 0.000138038 | 7.95 | 9.77237E-05 | 0.000416869 | chr2 | 219509276 | 219509677 | NM_001366287.2 | 0.000098 | - | 219509276 | 219509677 | 401, |
| peak3909 | 2.26 | 0.003162278 | 0.018620871 | 10.4 | 0.000120226 | 0.000512861 | chr2 | 219502907 | 219503207 | NM_001366288.2 | 0.00012 | - | 219502907 | 219503207 | 300, |
| peak3913 | 2.46 | 0.000162181 | 0.002187762 | 12.3 | 0.000128825 | 0.000537032 | chr2 | 219502902 | 219503201 | NM_001366289.2 | 0.00013 | - | 219502902 | 219503201 | 299, |
| peak3959 | 1.21 | 0.000776247 | 0.00691831 | 3.86 | 5.12861E-07 | 3.98107E-06 | chr2 | 230632237 | 230633352 | NM_001348315.2 | 0.00000051 | - | 230632237 | 230633352 | 229,21, |
| peak3964 | 1.43 | 0.00047863 | 0.004786301 | 4.03 | 7.76247E-07 | 5.7544E-06 | chr2 | 230632141 | 230633356 | NM_001348316.2 | 0.00000078 | - | 230632141 | 230633356 | 325,25, |
| peak3968 | 1.45 | 0.00074131 | 0.00676083 | 4.75 | 2.18776E-07 | 1.90546E-06 | chr2 | 230632151 | 230633367 | NM_001348318.2 | 0.00000022 | - | 230632151 | 230633367 | 315,36, |
| peak3972 | 1.49 | 0.000446684 | 0.004677351 | 3.89 | 5.49541E-07 | 4.2658E-06 | chr2 | 230632143 | 230633358 | NM_001348320.2 | 0.00000055 | - | 230632143 | 230633358 | 323,27, |
| peak3977 | 1.41 | 0.000645654 | 0.006025596 | 4.66 | 5.49541E-07 | 4.2658E-06 | chr2 | 230632150 | 230633366 | NM_001348324.2 | 0.00000055 | - | 230632150 | 230633366 | 316,35, |
| peak3982 | 1.45 | 0.000162181 | 0.002187762 | 3.88 | 5.01187E-07 | 3.89045E-06 | chr2 | 230632138 | 230633353 | NM_001348325.2 | 0.0000005 | - | 230632138 | 230633353 | 328,22, |
| peak3997 | 1.22 | 0.000467735 | 0.004786301 | 3.94 | 8.31764E-07 | 6.0256E-06 | chr2 | 230632235 | 230633350 | NM_001348329.2 | 0.00000083 | - | 230632235 | 230633350 | 231,19, |
| peak4002 | 1.36 | 0.001148154 | 0.009120108 | 4.64 | 4.0738E-07 | 3.31131E-06 | chr2 | 230632149 | 230633365 | NM_001348330.2 | 0.00000041 | - | 230632149 | 230633365 | 317,34, |
| peak4007 | 1.44 | 0.000446684 | 0.004570882 | 4.03 | 4.67735E-07 | 3.63078E-06 | chr2 | 230632141 | 230633355 | NM_001284214.2 | 0.00000047 | - | 230632141 | 230633355 | 325,24, |
| peak4012 | 1.5 | 0.000398107 | 0.004265795 | 3.89 | 4.57088E-07 | 3.54813E-06 | chr2 | 230632142 | 230633357 | NM_001284215.2 | 0.00000046 | - | 230632142 | 230633357 | 324,26, |
| peak4016 | 1.44 | 0.000162181 | 0.002187762 | 3.86 | 2.63027E-07 | 2.23872E-06 | chr2 | 230632138 | 230633352 | NM_001284216.2 | 0.00000026 | - | 230632138 | 230633352 | 328,21, |
| peak4020 | 1.19 | 0.00057544 | 0.005495409 | 3.86 | 2.51189E-07 | 2.13796E-06 | chr2 | 230632236 | 230633351 | NM_001348317.1 | 0.00000025 | - | 230632236 | 230633351 | 230,20, |
| peak4024 | 1.47 | 0.000331131 | 0.003715352 | 4.24 | 4.7863E-07 | 3.80189E-06 | chr2 | 230632146 | 230633362 | NM_001348319.1 | 0.00000048 | - | 230632146 | 230633362 | 320,31, |
| peak4035 | 1.48 | 0.000467735 | 0.004677351 | 4.03 | 6.91831E-07 | 5.24807E-06 | chr2 | 230632140 | 230633355 | NM_001348322.1 | 0.00000069 | - | 230632140 | 230633355 | 326,24, |
| peak4040 | 1.57 | 0.000218776 | 0.002754229 | 3.95 | 8.91251E-07 | 6.45654E-06 | chr2 | 230632152 | 230633368 | NM_001348323.1 | 0.00000089 | - | 230632152 | 230633368 | 314,37, |
| peak4045 | 1.54 | 8.12831E-05 | 0.001318257 | 4.12 | 3.38844E-05 | 0.000158489 | chr2 | 230632235 | 230633349 | NM_001348328.1 | 0.000034 | - | 230632235 | 230633349 | 231,18, |
| peak4050 | 1.47 | 0.000346737 | 0.003801894 | 3.89 | 6.76083E-07 | 5.12861E-06 | chr2 | 230632144 | 230633359 | NM_001348331.1 | 0.00000068 | - | 230632144 | 230633359 | 322,28, |
| peak4054 | 1.45 | 0.00074131 | 0.00676083 | 4.75 | 4.16869E-07 | 3.31131E-06 | chr2 | 230632151 | 230633367 | NM_001348332.1 | 0.00000042 | - | 230632151 | 230633367 | 315,36, |
| peak4059 | 1.42 | 0.000446684 | 0.004570882 | 4.03 | 7.4131E-07 | 5.49541E-06 | chr2 | 230632142 | 230633357 | NM_001348333.1 | 0.00000074 | - | 230632142 | 230633357 | 324,26, |
| peak4064 | 1.48 | 0.000446684 | 0.004677351 | 3.89 | 6.16595E-07 | 4.67735E-06 | chr2 | 230632143 | 230633358 | NM_004238.3 | 0.00000062 | - | 230632143 | 230633358 | 323,27, |
| peak4078 | 1.04 | 0.00057544 | 0.005495409 | 7.6 | 2.5704E-05 | 0.000125893 | chr2 | 231033782 | 231035365 | NM_080424.2 | 0.000026 | - | 231033782 | 231035365 | 171,29, |
| peak4098 | 2.47 | 5.88844E-06 | 0.000194984 | 4.99 | 3.63078E-05 | 0.000169824 | chr2 | 238275713 | 238275913 | NM_057167.3 | 0.000036 | - | 238275713 | 238275913 | 200, |
| peak4109 | 2.46 | 3.0903E-06 | 0.000114815 | 4.36 | 2.18776E-07 | 1.90546E-06 | chr2 | 238275715 | 238275915 | NM_057166.5 | 0.00000022 | - | 238275715 | 238275915 | 200, |
| peak4112 | 3.54 | 0.001071519 | 0.008709636 | 7.43 | 0.000147911 | 0.000616595 | chr2 | 239155026 | 239157764 | NM_022817.3 | 0.00015 | - | 239155026 | 239157764 | 139,62, |
| peak4128 | 5.55 | 7.58578E-05 | 0.001258925 | 4 | 7.4131E-10 | 1.51356E-08 | chr2 | 242168626 | 242168727 | NM_001243900.2 | 7.4E-10 | - | 242168626 | 242168727 | 101, |
| peak4139 | 5.05 | 3.46737E-05 | 0.00074131 | 3.9 | 3.89045E-06 | 2.34423E-05 | chr2 | 242433771 | 242433920 | NM_001271977.2 | 0.0000039 | - | 242433771 | 242433920 | 149, |

| | | | | | | | | | | | | | | | |
|---|---|---|---|---|---|---|---|---|---|---|---|---|---|---|---|
| peak4141 | 4.95 | 6.16595E-05 | 0.001096478 | 4 | 3.31131E-06 | 2.04174E-05 | chr2 | 242433772 | 242433922 | NM_001271980.2 | 0.0000033 | - | 242433772 | 242433922 | 150, |
| peak4181 | 2.07 | 4.0738E-05 | 0.000831764 | 16 | 6.16595E-05 | 0.000275423 | chr3 | 9512158 | 9512608 | NM_001292043.2 | 0.000062 | + | 9512158 | 9512608 | 450, |
| peak4185 | 2.28 | 3.63078E-06 | 0.000131826 | 16.4 | 0.00018197 | 0.00074131 | chr3 | 9512163 | 9512562 | NM_001349451.2 | 0.00018 | + | 9512163 | 9512562 | 399, |
| peak4188 | 2.31 | 0.00042658 | 0.004466836 | 15.9 | 6.0256E-05 | 0.000269153 | chr3 | 9512219 | 9512569 | NM_001080517.3 | 0.00006 | + | 9512219 | 9512569 | 350, |
| peak4206 | 3.3 | 0.000512861 | 0.005011872 | 2.22 | 9.33254E-07 | 3.80189E-05 | chr3 | 12544891 | 12545040 | NM_001145393.2 | 0.00000093 | + | 12544891 | 12545040 | 149, |
| peak4208 | 2.88 | 0.001202264 | 0.009549926 | 2.14 | 7.58578E-05 | 0.00162181 | chr3 | 12544885 | 12545082 | NM_001321278.2 | 0.000076 | + | 12544885 | 12545082 | 197, |
| peak4209 | 3.19 | 0.001122018 | 0.009120108 | 2.89 | 7.76247E-05 | 0.00162181 | chr3 | 12544912 | 12545012 | NM_001145392.1 | 0.000078 | + | 12544912 | 12545012 | 100, |
| peak4211 | 3.63 | 0.001318257 | 0.01 | 2 | 0.00042658 | 0.007413102 | chr3 | 12544929 | 12545079 | NM_001321279.1 | 0.00043 | + | 12544929 | 12545079 | 150, |
| peak4233 | 3.56 | 0.000194984 | 0.002511886 | 10.2 | 1.38038E-06 | 9.54993E-06 | chr3 | 15301246 | 15301496 | NR_046084.1 | 0.0000014 | + | 15301246 | 15301496 | 250, |
| peak4321 | 6.22 | 7.07946E-06 | 0.000223872 | 10.7 | 3.0903E-06 | 1.90546E-05 | chr3 | 42251384 | 42251583 | NM_001265608.2 | 0.0000031 | + | 42251384 | 42251583 | 199, |
| peak4323 | 6.52 | 7.58578E-05 | 0.00128825 | 10.7 | 8.51138E-06 | 4.67735E-05 | chr3 | 42251427 | 42251578 | NM_001265609.1 | 0.0000085 | + | 42251427 | 42251578 | 151, |
| peak4325 | 7.19 | 5.88844E-05 | 0.001071519 | 7.6 | 3.38844E-05 | 0.000162181 | chr3 | 42251416 | 42251566 | NM_014965.5 | 0.000034 | + | 42251416 | 42251566 | 150, |
| peak4335 | 2.44 | 0.000549541 | 0.005370318 | 7.32 | 4.7863E-05 | 0.000223872 | chr3 | 42699829 | 42700029 | NM_145166.4 | 0.000048 | + | 42699829 | 42700029 | 200, |
| peak4373 | 2.91 | 0.004466836 | 0.023988329 | 2 | 0.002754229 | 0.040738028 | chr3 | 48889067 | 48889217 | NR_109996.1 | 0.0028 | + | 48889067 | 48889217 | 150, |
| peak4571 | 4.34 | 2.51189E-05 | 0.000588844 | 4.67 | 0.000131826 | 0.002630268 | chr3 | 107519976 | 107520126 | NM_001142568.3 | 0.00013 | + | 107519976 | 107520126 | 150, |
| peak4572 | 1.81 | 0.000151356 | 0.002089296 | 11.9 | 3.38844E-08 | 3.89045E-07 | chr3 | 107493658 | 107508639 | NM_001276286.2 | 0.000000034 | + | 107493658 | 107508639 | 46,149,6, |
| peak4575 | 3.56 | 0.004466836 | 0.023988329 | 5.17 | 0.000104713 | 0.000457088 | chr3 | 107520019 | 107524357 | NM_020235.7 | 0.0001 | + | 107520019 | 107524357 | 109,141, |
| peak4625 | 1.49 | 0.001995262 | 0.013489629 | 8 | 0.00017378 | 0.003388442 | chr3 | 113804642 | 113804940 | NM_001256835.2 | 0.00017 | + | 113804642 | 113804940 | 298, |
| peak4627 | 1.23 | 0.002187762 | 0.014454398 | 8.5 | 0.000151356 | 0.002951209 | chr3 | 113804648 | 113804946 | NM_001256837.2 | 0.00015 | + | 113804648 | 113804946 | 298, |
| peak4636 | 1.58 | 8.31764E-05 | 0.001380384 | 3.25 | 0.005128614 | 0.017378008 | chr3 | 122283383 | 122284778 | NM_138287.3 | 0.0051 | + | 122283383 | 122284778 | 77,73, |
| peak4637 | 1.42 | 0.000316228 | 0.003548134 | 74.6 | 3.63078E-05 | 0.00017378 | chr3 | 122288140 | 122289316 | NM_138287.3 | 0.000036 | + | 122288140 | 122289316 | 731,15, |
| peak4646 | 1.96 | 0.003388442 | 0.019498446 | 7.14 | 2.18776E-06 | 1.44544E-05 | chr3 | 122420203 | 122420404 | NM_017554.3 | 0.0000022 | + | 122420203 | 122420404 | 201, |
| peak4647 | 1.51 | 6.60693E-05 | 0.001148154 | 17 | 4.46684E-06 | 2.69153E-05 | chr3 | 122436968 | 122437800 | NM_017554.3 | 0.0000045 | + | 122436968 | 122437800 | 156,595, |
| peak4653 | 1.27 | 0.002290868 | 0.015135612 | 9.2 | 6.45654E-06 | 3.63078E-05 | chr3 | 124456481 | 124456978 | NM_000373.4 | 0.0000065 | + | 124456481 | 124456978 | 497, |
| peak4661 | 1.92 | 0.000912011 | 0.007762471 | 5.91 | 0.002398833 | 0.008317638 | chr3 | 127788543 | 127788792 | NM_013336.4 | 0.0024 | + | 127788543 | 127788792 | 249, |
| peak4698 | 2.28 | 8.31764E-05 | 0.001380384 | 10.9 | 1.7378E-07 | 1.58489E-06 | chr3 | 140692835 | 140695440 | NM_001104647.3 | 0.00000017 | + | 140692835 | 140695440 | 12,339, |
| peak4700 | 1.13 | 0.004466836 | 0.023988329 | 16.5 | 6.45654E-09 | 9.33254E-08 | chr3 | 141011154 | 141011352 | NM_001037172.3 | 6.5E-09 | + | 141011154 | 141011352 | 198, |
| peak4703 | 1.36 | 5.01187E-07 | 2.75423E-05 | 21.9 | 1.99526E-11 | 6.30957E-10 | chr3 | 141162361 | 141163109 | NM_001080412.3 | 2E-11 | + | 141162361 | 141163109 | 748, |
| peak4708 | 3.1 | 5.24807E-05 | 0.001 | 6.3 | 3.16228E-06 | 1.99526E-05 | chr3 | 141166393 | 141166643 | NM_001080412.3 | 0.0000032 | + | 141166393 | 141166643 | 250, |
| peak4715 | 3.08 | 4.67735E-07 | 2.63027E-05 | 4.58 | 3.54813E-08 | 3.98107E-07 | chr3 | 141166384 | 141166685 | NM_001350099.2 | 0.000000035 | + | 141166384 | 141166685 | 301, |
| peak4722 | 3.08 | 4.67735E-07 | 2.63027E-05 | 4.58 | 4.16869E-08 | 4.67735E-07 | chr3 | 141166384 | 141166685 | NM_001350100.2 | 0.000000042 | + | 141166384 | 141166685 | 301, |
| peak4782 | 6.89 | 0.001949845 | 0.013489629 | 4 | 6.91831E-09 | 1.28825E-07 | chr3 | 158320618 | 158322923 | NM_001195433.2 | 6.9E-09 | + | 158320618 | 158322923 | 110,38, |
| peak4847 | 5.47 | 1.41254E-06 | 6.30957E-05 | 4.08 | 0.012302688 | 0.039810717 | chr3 | 183528425 | 183528575 | NM_001351370.1 | 0.012 | + | 183528425 | 183528575 | 150, |
| peak4868 | 1.45 | 0.000616595 | 0.005888437 | 3.35 | 1.25893E-12 | 6.30957E-11 | chr3 | 184042796 | 184045124 | NM_198242.3 | 1.3E-12 | + | 184042796 | 184045124 | 106,105,160,101,103,103,121, |
| peak4953 | 1.14 | 0.004265795 | 0.023442288 | 19.8 | 5.01187E-11 | 1.47911E-09 | chr3 | 12625394 | 12625642 | NM_001354689.3 | 5E-11 | - | 12625394 | 12625642 | 248, |
| peak4955 | 1.26 | 0.003467369 | 0.019952623 | 17.8 | 1.38038E-08 | 1.77828E-07 | chr3 | 12625446 | 12625646 | NM_002880.3 | 0.000000014 | - | 12625446 | 12625646 | 200, |
| peak4956 | 1.6 | 0.003388442 | 0.019498446 | 22.6 | 2.75423E-05 | 0.000134896 | chr3 | 12625793 | 12625943 | NM_002880.3 | 0.000028 | - | 12625793 | 12625943 | 150, |
| peak4957 | 1.22 | 0.000165959 | 0.002187762 | 15.4 | 0.000263027 | 0.001047129 | chr3 | 12625403 | 12625651 | NM_001354690.2 | 0.00026 | - | 12625403 | 12625651 | 248, |
| peak4958 | 1.62 | 0.001412538 | 0.010471285 | 21.8 | 0.0001 | 0.000436516 | chr3 | 12625799 | 12625948 | NM_001354690.2 | 0.0001 | - | 12625799 | 12625948 | 149, |
| peak4960 | 1.21 | 0.000186209 | 0.002398833 | 15.4 | 1.20226E-09 | 2.23872E-08 | chr3 | 12625403 | 12625652 | NM_001354692.2 | 1.2E-09 | - | 12625403 | 12625652 | 249, |
| peak4961 | 1.8 | 0.001023293 | 0.008317638 | 20 | 2.51189E-10 | 6.0256E-09 | chr3 | 12625799 | 12625949 | NM_001354692.2 | 2.5E-10 | - | 12625799 | 12625949 | 150, |
| peak4966 | 1.21 | 0.000186209 | 0.002398833 | 15.4 | 2.81838E-09 | 4.67735E-08 | chr3 | 12625403 | 12625652 | NM_001354695.2 | 2.8E-09 | - | 12625403 | 12625652 | 249, |
| peak4967 | 1.8 | 0.001023293 | 0.008317638 | 20 | 2.45471E-09 | 4.16869E-08 | chr3 | 12625799 | 12625949 | NM_001354695.2 | 2.5E-09 | - | 12625799 | 12625949 | 150, |
| peak4970 | 1.24 | 0.000239883 | 0.002884032 | 15.4 | 5.01187E-11 | 1.34896E-09 | chr3 | 12625405 | 12625656 | NR_148941.2 | 5E-11 | - | 12625405 | 12625656 | 251, |
| peak4971 | 1.61 | 0.003235937 | 0.019054607 | 16 | 4.0738E-10 | 8.91251E-09 | chr3 | 12625804 | 12625955 | NR_148941.2 | 4.1E-10 | - | 12625804 | 12625955 | 151, |
| peak4982 | 3.7 | 8.91251E-06 | 0.000263027 | 13 | 3.89045E-07 | 3.16228E-06 | chr3 | 14154401 | 14154596 | NM_144636.3 | 0.00000039 | - | 14154401 | 14154596 | 195, |
| peak5000 | 3.7 | 0.002187762 | 0.014454398 | 6.67 | 0.000977237 | 0.003630781 | chr3 | 15131954 | 15137488 | NM_001302378.2 | 0.00098 | - | 15131954 | 15137488 | 92,9, |
| peak5001 | 1.36 | 0.000346737 | 0.003890451 | 37.2 | 5.12861E-07 | 3.98107E-06 | chr3 | 15115167 | 15117138 | NM_022340.4 | 0.00000051 | - | 15115167 | 15117138 | 1270,26, |
| peak5003 | 3.19 | 0.00128825 | 0.01 | 6 | 5.12861E-07 | 3.98107E-06 | chr3 | 15131960 | 15137494 | NM_022340.4 | 0.00000051 | - | 15131960 | 15137494 | 86,15, |
| peak5020 | 1.41 | 0.001659587 | 0.011748976 | 3.23 | 3.98107E-06 | 2.39883E-05 | chr3 | 17202335 | 17202684 | NM_001134381.1 | 0.000004 | - | 17202335 | 17202684 | 349, |
| peak5023 | 1.37 | 0.003890451 | 0.021877616 | 3.75 | 9.12011E-07 | 6.60693E-06 | chr3 | 17202331 | 17202680 | NM_014744.2 | 0.00000091 | - | 17202331 | 17202680 | 349, |
| peak5025 | 1.25 | 0.00047863 | 0.004897788 | 4.09 | 9.33254E-06 | 5.12861E-05 | chr3 | 17202302 | 17202653 | NM_001349073.1 | 0.0000093 | - | 17202302 | 17202653 | 351, |
| peak5027 | 1.37 | 0.003090295 | 0.018620871 | 3.42 | 3.23594E-06 | 1.99526E-05 | chr3 | 17202332 | 17202681 | NM_001349074.1 | 0.0000032 | - | 17202332 | 17202681 | 349, |
| peak5028 | 1.23 | 0.001949845 | 0.013489629 | 3.2 | 8.91251E-06 | 4.89779E-05 | chr3 | 17202349 | 17202649 | NM_001349075.1 | 0.0000089 | - | 17202349 | 17202649 | 300, |
| peak5030 | 1.34 | 0.001258925 | 0.009772372 | 3.13 | 1.28825E-05 | 6.76083E-05 | chr3 | 17202349 | 17202650 | NM_001349076.1 | 0.000013 | - | 17202349 | 17202650 | 301, |
| peak5036 | 1.43 | 0.004168694 | 0.022908677 | 3.71 | 1.12202E-06 | 7.94328E-06 | chr3 | 17202327 | 17202675 | NM_001349079.1 | 0.0000011 | - | 17202327 | 17202675 | 348, |
| peak5038 | 1.02 | 0.003630781 | 0.020417379 | 3.32 | 7.07946E-06 | 3.98107E-05 | chr3 | 17202343 | 17202643 | NM_001349080.1 | 0.0000071 | - | 17202343 | 17202643 | 300, |

| | | | | | | | | | | | | | | | |
|---|---|---|---|---|---|---|---|---|---|---|---|---|---|---|---|
| peak5040 | 1.48 | 0.003801894 | 0.021379621 | 3.71 | 1.44544E-07 | 1.31826E-06 | chr3 | 17202325 | 17202674 | NM_001349081.1 | 0.00000014 | - | 17202325 | 17202674 | 349, |
| peak5042 | 1.41 | 0.001659587 | 0.011748976 | 3.23 | 3.98107E-06 | 2.39883E-05 | chr3 | 17202335 | 17202684 | NM_001349082.1 | 0.000004 | - | 17202335 | 17202684 | 349, |
| peak5044 | 1.34 | 0.001258925 | 0.009772372 | 3.13 | 1.25893E-05 | 6.60693E-05 | chr3 | 17202349 | 17202650 | NM_001349083.1 | 0.000013 | - | 17202349 | 17202650 | 301, |
| peak5046 | 1.41 | 0.003311311 | 0.019498446 | 3.75 | 6.60693E-06 | 3.71535E-05 | chr3 | 17202331 | 17202680 | NM_001349084.1 | 0.0000066 | - | 17202331 | 17202680 | 349, |
| peak5054 | 1.02 | 0.003715352 | 0.020892961 | 3.32 | 0.000147911 | 0.000616595 | chr3 | 17202343 | 17202643 | NM_001349088.1 | 0.00015 | - | 17202343 | 17202643 | 300, |
| peak5058 | 1.45 | 0.001348963 | 0.01023293 | 3.35 | 9.54993E-06 | 5.12861E-05 | chr3 | 17202337 | 17202686 | NM_001349090.1 | 0.0000095 | - | 17202337 | 17202686 | 349, |
| peak5060 | 1.38 | 0.003715352 | 0.020892961 | 3.75 | 8.12831E-06 | 4.46684E-05 | chr3 | 17202331 | 17202680 | NM_001349091.1 | 0.0000081 | - | 17202331 | 17202680 | 349, |
| peak5064 | 3.21 | 0.002884032 | 0.017782794 | 23.8 | 9.33254E-06 | 5.01187E-05 | chr3 | 18435957 | 18436307 | NM_001195470.3 | 0.0000093 | - | 18435957 | 18436307 | 350, |
| peak5068 | 3.42 | 0.000186209 | 0.002398833 | 22 | 1.65959E-06 | 1.12202E-05 | chr3 | 18435956 | 18436306 | NM_002971.6 | 0.0000017 | - | 18435956 | 18436306 | 350, |
| peak5072 | 3.23 | 0.000676083 | 0.006309573 | 23.3 | 5.88844E-06 | 3.38844E-05 | chr3 | 18435958 | 18436308 | NM_001131010.4 | 0.0000059 | - | 18435958 | 18436308 | 350, |
| peak5075 | 2.77 | 0.00042658 | 0.004466836 | 24.2 | 1.47911E-08 | 1.90546E-07 | chr3 | 18435955 | 18436304 | NM_001322871.2 | 0.000000015 | - | 18435955 | 18436304 | 349, |
| peak5081 | 3.16 | 0.00074131 | 0.00676083 | 15.5 | 4.57088E-07 | 3.63078E-06 | chr3 | 18435965 | 18436316 | NM_001322876.2 | 0.00000046 | - | 18435965 | 18436316 | 351, |
| peak5084 | 2.88 | 0.000165959 | 0.002187762 | 26.7 | 2.0893E-07 | 1.8197E-06 | chr3 | 18436000 | 18436300 | NM_001322872.2 | 0.00000021 | - | 18436000 | 18436300 | 300, |
| peak5087 | 3.21 | 0.000660693 | 0.00616595 | 16.4 | 1.54882E-05 | 7.94328E-05 | chr3 | 18435961 | 18436311 | NM_001322874.2 | 0.000015 | - | 18435961 | 18436311 | 350, |
| peak5090 | 3.22 | 0.00057544 | 0.005623413 | 23.3 | 3.98107E-06 | 2.39883E-05 | chr3 | 18435958 | 18436307 | NM_001322875.1 | 0.000004 | - | 18435958 | 18436307 | 349, |
| peak5145 | 2 | 0.000707946 | 0.006456542 | 4.4 | 0.001380384 | 0.004897788 | chr3 | 36897023 | 36897372 | NM_001329998.2 | 0.0014 | - | 36897023 | 36897372 | 349, |
| peak5147 | 1.96 | 0.000562341 | 0.005495409 | 4.54 | 0.000251189 | 0.001 | chr3 | 36897033 | 36897333 | NM_014831.3 | 0.00025 | - | 36897033 | 36897333 | 300, |
| peak5150 | 4.02 | 8.12831E-06 | 0.000245471 | 5.52 | 1.99526E-06 | 1.31826E-05 | chr3 | 37136368 | 37144489 | NM_006309.4 | 0.000002 | - | 37136368 | 37144489 | 31,45,75, |
| peak5154 | 4.03 | 8.91251E-06 | 0.000263027 | 5.52 | 0.000151356 | 0.000630957 | chr3 | 37136370 | 37154432 | NM_001282691.1 | 0.00015 | - | 37136370 | 37154432 | 29,45,48,27, |
| peak5160 | 4.13 | 2.95121E-05 | 0.000660693 | 7.42 | 0.001862087 | 0.006606934 | chr3 | 37136392 | 37149591 | NM_001348298.1 | 0.0019 | - | 37136392 | 37149591 | 7,45,69,28, |
| peak5165 | 3.27 | 0.001122018 | 0.008912509 | 5.84 | 0.000389045 | 0.001513561 | chr3 | 37136396 | 37163182 | NM_001348300.1 | 0.00039 | - | 37136396 | 37163182 | 3,45,45,57, |
| peak5168 | 4.23 | 0.000120226 | 0.001737801 | 6.22 | 0.000158489 | 0.000645654 | chr3 | 37136388 | 37163175 | NM_001348303.1 | 0.00016 | - | 37136388 | 37163175 | 11,45,45,50, |
| peak5170 | 2.26 | 3.80189E-05 | 0.000794328 | 6.25 | 9.33254E-05 | 0.00040738 | chr3 | 37136394 | 37190498 | NM_001348304.1 | 0.000093 | - | 37136394 | 37190498 | 5,45,87,114, |
| peak5172 | 2.27 | 4.16869E-05 | 0.000851138 | 6.45 | 0.000301995 | 0.001202264 | chr3 | 37136394 | 37190496 | NM_001348306.1 | 0.0003 | - | 37136394 | 37190496 | 5,45,87,112, |
| peak5174 | 2.29 | 5.49541E-05 | 0.001023293 | 6.9 | 4.57088E-05 | 0.000213796 | chr3 | 37136395 | 37190496 | NM_001348310.1 | 0.000046 | - | 37136395 | 37190496 | 4,45,87,112, |
| peak5175 | 1.73 | 0.000812831 | 0.007079458 | 4.46 | 2.45471E-06 | 1.58489E-05 | chr3 | 37125272 | 37190504 | NM_017724.2 | 0.0000025 | - | 37125272 | 37190504 | 25,117,45,87,120, |
| peak5207 | 2.62 | 3.0903E-06 | 0.000114815 | 14.5 | 0.004265795 | 0.014454398 | chr3 | 47162485 | 47162785 | NM_014159.6 | 0.0043 | - | 47162485 | 47162785 | 300, |
| peak5213 | 2.99 | 5.37032E-07 | 2.88403E-05 | 13.8 | 5.24807E-05 | 0.000239883 | chr3 | 47162518 | 47162768 | NM_001349370.2 | 0.000052 | - | 47162518 | 47162768 | 250, |
| peak5278 | 1.35 | 0.003019952 | 0.018197009 | 19.5 | 3.71535E-07 | 3.01995E-06 | chr3 | 49764507 | 49764757 | NM_153273.4 | 0.00000037 | - | 49764507 | 49764757 | 250, |
| peak5284 | 2.13 | 0.003388442 | 0.019498446 | 7 | 0.002344229 | 0.008128305 | chr3 | 49939978 | 49940276 | NM_001318913.1 | 0.0023 | - | 49939978 | 49940276 | 298, |
| peak5285 | 2.22 | 0.001513561 | 0.010964782 | 5.29 | 0.000501187 | 0.00851138 | chr3 | 49939967 | 49940266 | NR_134919.2 | 0.0005 | - | 49939967 | 49940266 | 299, |
| peak5294 | 3.42 | 0.000537032 | 0.005248075 | 7.4 | 2.63027E-06 | 1.65959E-05 | chr3 | 52651290 | 52651491 | NM_001350074.2 | 0.0000026 | - | 52651290 | 52651491 | 201, |
| peak5297 | 3.42 | 0.000537032 | 0.005248075 | 7.4 | 2.39883E-06 | 1.54882E-05 | chr3 | 52651290 | 52651491 | NM_001350077.2 | 0.0000024 | - | 52651290 | 52651491 | 201, |
| peak5300 | 2.95 | 0.000933254 | 0.007943282 | 6.67 | 2.39883E-06 | 1.54882E-05 | chr3 | 52651329 | 52651480 | NM_001350075.2 | 0.0000024 | - | 52651329 | 52651480 | 151, |
| peak5303 | 3.22 | 0.000933254 | 0.007943282 | 8 | 1.54882E-06 | 1.07152E-05 | chr3 | 52651292 | 52651492 | NM_001350076.2 | 0.0000015 | - | 52651292 | 52651492 | 200, |
| peak5306 | 3.22 | 0.000933254 | 0.007943282 | 8 | 1.47911E-06 | 0.00001 | chr3 | 52651291 | 52651491 | NM_001350078.2 | 0.0000015 | - | 52651291 | 52651491 | 200, |
| peak5312 | 3.6 | 0.002187762 | 0.014454398 | 8.17 | 1.58489E-05 | 8.12831E-05 | chr3 | 52651296 | 52651497 | NM_001366070.2 | 0.000016 | - | 52651296 | 52651497 | 201, |
| peak5315 | 2.99 | 0.001202264 | 0.009549926 | 8.17 | 6.91831E-07 | 5.24807E-06 | chr3 | 52651309 | 52651509 | NM_001366071.2 | 0.00000069 | - | 52651309 | 52651509 | 200, |
| peak5318 | 3 | 0.002398833 | 0.015488166 | 9.17 | 5.01187E-07 | 3.89045E-06 | chr3 | 52651313 | 52651513 | NM_001366072.2 | 0.0000005 | - | 52651313 | 52651513 | 200, |
| peak5324 | 3.12 | 0.001318257 | 0.01 | 7.67 | 7.4131E-07 | 5.49541E-06 | chr3 | 52651286 | 52651486 | NM_001366074.2 | 0.00000074 | - | 52651286 | 52651486 | 200, |
| peak5327 | 3.51 | 0.004365158 | 0.023442288 | 8.17 | 3.16228E-06 | 1.94984E-05 | chr3 | 52651303 | 52651504 | NM_001366075.2 | 0.0000032 | - | 52651303 | 52651504 | 201, |
| peak5330 | 3.62 | 0.00020893 | 0.002630268 | 8.17 | 2.29087E-05 | 0.000114815 | chr3 | 52651293 | 52651493 | NM_001366076.2 | 0.000023 | - | 52651293 | 52651493 | 200, |
| peak5333 | 2.98 | 0.00128825 | 0.009772372 | 8.5 | 1.58489E-06 | 1.07152E-05 | chr3 | 52651328 | 52651479 | NM_018313.5 | 0.0000016 | - | 52651328 | 52651479 | 151, |
| peak5336 | 3.13 | 0.001202264 | 0.009332543 | 9.33 | 2.75423E-07 | 2.29087E-06 | chr3 | 52651323 | 52651473 | NM_181042.5 | 0.00000028 | - | 52651323 | 52651473 | 150, |
| peak5341 | 5.34 | 0.000446684 | 0.004677351 | 7.15 | 1.7378E-07 | 1.54882E-06 | chr3 | 55504127 | 55504228 | NM_001256105.1 | 0.00000017 | - | 55504127 | 55504228 | 101, |
| peak5343 | 5.35 | 0.000389045 | 0.004168694 | 7.15 | 4.57088E-07 | 3.63078E-06 | chr3 | 55504125 | 55504226 | NM_003392.4 | 0.00000046 | - | 55504125 | 55504226 | 101, |
| peak5529 | 4.26 | 0.000676083 | 0.006309573 | 1.47 | 6.30957E-05 | 0.001348963 | chr3 | 99569847 | 99594948 | NM_001282793.1 | 0.000063 | - | 99569847 | 99594948 | 67,33, |
| peak5530 | 1.39 | 0.001862087 | 0.012882496 | 16.7 | 2.0893E-05 | 0.000104713 | chr3 | 99567268 | 99567666 | NM_001282794.1 | 0.000021 | - | 99567268 | 99567666 | 398, |
| peak5534 | 1.75 | 4.16869E-05 | 0.000851138 | 16.7 | 6.60693E-07 | 4.89779E-06 | chr3 | 99567218 | 99567716 | NM_182909.3 | 0.00000066 | - | 99567218 | 99567716 | 498, |
| peak5546 | 1.28 | 5.37032E-07 | 2.88403E-05 | 50.6 | 1.20226E-06 | 8.51138E-06 | chr3 | 99567014 | 99567711 | NM_001042459.2 | 0.0000012 | - | 99567014 | 99567711 | 697, |
| peak5549 | 2.93 | 0.001096478 | 0.008912509 | 8.4 | 7.24436E-06 | 4.0738E-05 | chr3 | 100084274 | 100084474 | NM_014820.5 | 0.0000072 | - | 100084274 | 100084474 | 200, |
| peak5553 | 1.39 | 0.004570882 | 0.024547089 | 14.7 | 5.37032E-07 | 4.16869E-06 | chr3 | 101383911 | 101384209 | NM_014415.4 | 0.00000054 | - | 101383911 | 101384209 | 298, |
| peak5643 | 1.85 | 7.4131E-05 | 0.001258925 | 18.5 | 6.76083E-08 | 6.91831E-07 | chr3 | 114057111 | 114057511 | NM_001164342.2 | 0.000000068 | - | 114057111 | 114057511 | 400, |
| peak5650 | 1.77 | 0.000120226 | 0.001778279 | 19 | 7.76247E-08 | 7.76247E-07 | chr3 | 114057053 | 114057503 | NM_001164347.2 | 0.000000078 | - | 114057053 | 114057503 | 450, |
| peak5656 | 1.72 | 0.0001 | 0.001548817 | 18.2 | 1.65959E-07 | 1.51356E-06 | chr3 | 114057127 | 114057478 | NM_001164346.2 | 0.00000017 | - | 114057127 | 114057478 | 351, |
| peak5661 | 1.61 | 0.000436516 | 0.004570882 | 18.7 | 2.63027E-07 | 2.23872E-06 | chr3 | 114057122 | 114057472 | NM_001164343.2 | 0.00000026 | - | 114057122 | 114057472 | 350, |
| peak5666 | 1.65 | 4.2658E-05 | 0.000851138 | 18.7 | 7.24436E-08 | 7.4131E-07 | chr3 | 114057117 | 114057467 | NM_001164344.3 | 0.000000072 | - | 114057117 | 114057467 | 350, |

| | | | | | | | | | | | | | | | |
|---|---|---|---|---|---|---|---|---|---|---|---|---|---|---|---|
| peak5671 | 1.73 | 4.0738E-05 | 0.000831764 | 18.7 | 2.18776E-07 | 1.90546E-06 | chr3 | 114057120 | 114057471 | NM_001164345.3 | 0.00000022 | - | 114057120 | 114057471 | 351, |
| peak5676 | 1.82 | 0.000389045 | 0.004168694 | 14.4 | 1.51356E-05 | 7.94328E-05 | chr3 | 114057042 | 114057493 | NR_121662.2 | 0.000015 | - | 114057042 | 114057493 | 451, |
| peak5679 | 1.52 | 0.000263027 | 0.003090295 | 18.5 | 7.07946E-08 | 7.24436E-07 | chr3 | 114057058 | 114057509 | NM_001348804.2 | 0.000000071 | - | 114057058 | 114057509 | 451, |
| peak5685 | 1.91 | 4.89779E-05 | 0.000933254 | 15.4 | 1.31826E-06 | 9.12011E-06 | chr3 | 114057049 | 114057499 | NM_001348805.2 | 0.0000013 | - | 114057049 | 114057499 | 450, |
| peak5692 | 1.85 | 4.0738E-05 | 0.000831764 | 15.4 | 1.44544E-06 | 0.00001 | chr3 | 114057049 | 114057499 | NM_001348800.2 | 0.0000014 | - | 114057049 | 114057499 | 450, |
| peak5698 | 1.72 | 0.0001 | 0.001548817 | 18.2 | 1.41254E-07 | 1.31826E-06 | chr3 | 114057128 | 114057478 | NM_001348801.2 | 0.00000014 | - | 114057128 | 114057478 | 350, |
| peak5703 | 1.78 | 0.00018197 | 0.002398833 | 14.3 | 1.07152E-06 | 7.58578E-06 | chr3 | 114057035 | 114057486 | NM_001348802.2 | 0.0000011 | - | 114057035 | 114057486 | 451, |
| peak5710 | 1.75 | 8.31764E-05 | 0.001380384 | 18.2 | 1.54882E-07 | 1.41254E-06 | chr3 | 114057125 | 114057476 | NM_001348803.2 | 0.00000015 | - | 114057125 | 114057476 | 351, |
| peak5715 | 1.78 | 0.000158489 | 0.002187762 | 17 | 2.51189E-06 | 1.58489E-05 | chr3 | 114057040 | 114057491 | NM_015642.6 | 0.0000025 | - | 114057040 | 114057491 | 451, |
| peak5739 | 2.14 | 0.000933254 | 0.007943282 | 3.26 | 0.004570882 | 0.015488166 | chr3 | 121445807 | 121448054 | NM_001256488.1 | 0.0046 | - | 121445807 | 121448054 | 81,20, |
| peak5755 | 2.37 | 0.000151356 | 0.002089296 | 2.63 | 0.006456542 | 0.021379621 | chr3 | 121445831 | 121448078 | NM_001256487.2 | 0.0065 | - | 121445831 | 121448078 | 57,44, |
| peak5800 | 1.51 | 0.000218776 | 0.002754229 | 3.54 | 2.95121E-07 | 2.45471E-06 | chr3 | 122255877 | 122264302 | NM_001146104.2 | 0.0000003 | - | 122255877 | 122264302 | 43,381,24, |
| peak5801 | 4.95 | 0.00040738 | 0.004365158 | 5.31 | 0.000380189 | 0.001479108 | chr3 | 122274302 | 122274700 | NM_001146104.2 | 0.00038 | - | 122274302 | 122274700 | 398, |
| peak5806 | 1.5 | 0.000794328 | 0.00691831 | 3.42 | 2.63027E-09 | 4.36516E-08 | chr3 | 122255914 | 122264292 | NM_001146106.2 | 2.6E-09 | - | 122255914 | 122264292 | 6,381,14, |
| peak5807 | 4.99 | 0.000281838 | 0.003235937 | 6.8 | 4.67735E-06 | 2.81838E-05 | chr3 | 122274295 | 122274696 | NM_001146106.2 | 0.0000047 | - | 122274295 | 122274696 | 401, |
| peak5828 | 2.43 | 0.00060256 | 0.005754399 | 6.61 | 3.16228E-07 | 2.5704E-06 | chr3 | 123419090 | 123419340 | NM_053027.4 | 0.00000032 | - | 123419090 | 123419340 | 250, |
| peak5833 | 2.33 | 0.001318257 | 0.01 | 6.85 | 3.54813E-08 | 3.98107E-07 | chr3 | 123419084 | 123419334 | NM_053028.4 | 0.000000035 | - | 123419084 | 123419334 | 250, |
| peak5836 | 1.21 | 5.49541E-05 | 0.001023293 | 16.3 | 4.57088E-08 | 5.01187E-07 | chr3 | 123633858 | 123650309 | NM_001308317.2 | 0.000000046 | - | 123633858 | 123650309 | 707,133,155, |
| peak5839 | 1.04 | 0.000831764 | 0.00724436 | 17.7 | 3.0903E-07 | 2.5704E-06 | chr3 | 123633860 | 123650312 | NM_001366335.1 | 0.00000031 | - | 123633860 | 123650312 | 705,133,158, |
| peak5842 | 1.24 | 0.000114815 | 0.001698244 | 20 | 4.36516E-08 | 4.7863E-07 | chr3 | 123633851 | 123650297 | NM_001366336.1 | 0.000000044 | - | 123633851 | 123650297 | 714,133,143, |
| peak5850 | 1.49 | 0.000954993 | 0.008128305 | 16.4 | 6.0256E-08 | 6.30957E-07 | chr3 | 123633858 | 123650291 | NM_001366338.1 | 0.00000006 | - | 123633858 | 123650291 | 278,133,137, |
| peak5853 | 1.21 | 0.000263027 | 0.003090295 | 20 | 1.7378E-08 | 2.18776E-07 | chr3 | 123633853 | 123650301 | NM_001366339.1 | 0.000000017 | - | 123633853 | 123650301 | 712,133,147, |
| peak5855 | 1.19 | 0.000141254 | 0.001995262 | 20 | 1.14815E-07 | 1.09648E-06 | chr3 | 123633851 | 123650297 | NM_022757.5 | 0.00000011 | - | 123633851 | 123650297 | 714,133,143, |
| peak5872 | 2.41 | 0.000933254 | 0.007943282 | 39.2 | 7.58578E-07 | 5.62341E-06 | chr3 | 124952456 | 124952757 | NM_001348433.2 | 0.00000076 | - | 124952456 | 124952757 | 301, |
| peak5876 | 2.38 | 0.000204174 | 0.002630268 | 32.6 | 6.30957E-11 | 1.69824E-09 | chr3 | 124952453 | 124952754 | NM_001348424.1 | 6.3E-11 | - | 124952453 | 124952754 | 301, |
| peak5883 | 2.63 | 0.00074131 | 0.006606934 | 32.5 | 2.5704E-06 | 1.65959E-05 | chr3 | 124951532 | 124952031 | NM_001348425.2 | 0.0000026 | - | 124951532 | 124952031 | 499, |
| peak5896 | 2.41 | 0.000831764 | 0.00724436 | 33.2 | 6.91831E-10 | 1.41254E-08 | chr3 | 124952451 | 124952752 | NM_001348428.2 | 6.9E-10 | - | 124952451 | 124952752 | 301, |
| peak5899 | 2.68 | 0.000977237 | 0.008128305 | 32.7 | 4.16869E-06 | 2.51189E-05 | chr3 | 124951529 | 124952027 | NM_001348429.2 | 0.0000042 | - | 124951529 | 124952027 | 498, |
| peak5906 | 2.76 | 0.000354813 | 0.003890451 | 32.5 | 3.16228E-06 | 1.94984E-05 | chr3 | 124951533 | 124952032 | NM_001348431.2 | 0.0000032 | - | 124951533 | 124952032 | 499, |
| peak5944 | 1.55 | 6.16595E-05 | 0.001096478 | 16.6 | 1.8197E-06 | 1.20226E-05 | chr3 | 129389367 | 129407376 | NM_001349272.1 | 0.0000018 | - | 129389367 | 129407376 | 740,59, |
| peak5947 | 1.5 | 1.94984E-05 | 0.000489779 | 15.6 | 4.57088E-09 | 6.91831E-08 | chr3 | 129389367 | 129407127 | NM_015008.5 | 4.6E-09 | - | 129389367 | 129407127 | 740,108, |
| peak5950 | 1.61 | 2.75423E-05 | 0.000630957 | 12.9 | 1.07152E-08 | 1.44544E-07 | chr3 | 129389360 | 129442906 | NM_001349274.2 | 0.000000011 | - | 129389360 | 129442906 | 747,100, |
| peak5953 | 1.69 | 3.63078E-05 | 0.000758578 | 16.9 | 2.29087E-10 | 5.49541E-09 | chr3 | 129389352 | 129513256 | NM_001349273.2 | 2.3E-10 | - | 129389352 | 129513256 | 755,90, |
| peak5956 | 1.57 | 0.00018197 | 0.002344229 | 16.7 | 7.76247E-09 | 1.09648E-07 | chr3 | 129389370 | 129546707 | NM_001128224.2 | 7.8E-09 | - | 129389370 | 129546707 | 737,62, |
| peak5959 | 1.62 | 0.000275423 | 0.003162278 | 17.4 | 1.25893E-11 | 4.16869E-10 | chr3 | 129389399 | 129546732 | NM_001017395.5 | 1.3E-11 | - | 129389399 | 129546732 | 708,87, |
| peak5965 | 2.34 | 0.002290868 | 0.015135612 | 13.4 | 1.99526E-10 | 4.89779E-09 | chr3 | 129547090 | 129547240 | NM_001349263.2 | 2E-10 | - | 129547090 | 129547240 | 150, |
| peak5968 | 1.69 | 0.000120226 | 0.001737801 | 17.6 | 3.98107E-11 | 1.23027E-09 | chr3 | 129389401 | 129546734 | NM_001349264.2 | 4E-11 | - | 129389401 | 129546734 | 706,89, |
| peak5972 | 1.56 | 3.46737E-05 | 0.00074131 | 15.9 | 2.51189E-11 | 7.58578E-10 | chr3 | 129389381 | 129546719 | NM_001349265.2 | 2.5E-11 | - | 129389381 | 129546719 | 726,74, |
| peak5973 | 1.97 | 0.004466836 | 0.023988329 | 20 | 2.51189E-11 | 7.58578E-10 | chr3 | 129547068 | 129547219 | NM_001349265.2 | 2.5E-11 | - | 129547068 | 129547219 | 151, |
| peak5976 | 1.59 | 1.94984E-05 | 0.000489779 | 11.3 | 7.94328E-11 | 2.0893E-09 | chr3 | 129389356 | 129546740 | NM_001349266.2 | 7.9E-11 | - | 129389356 | 129546740 | 751,95, |
| peak5977 | 2.27 | 0.003388442 | 0.019952623 | 13 | 7.94328E-11 | 2.0893E-09 | chr3 | 129547087 | 129547237 | NM_001349266.2 | 7.9E-11 | - | 129547087 | 129547237 | 150, |
| peak5980 | 1.63 | 0.000123027 | 0.001778279 | 17.2 | 7.94328E-12 | 3.23594E-10 | chr3 | 129389397 | 129546729 | NM_001349268.2 | 7.9E-12 | - | 129389397 | 129546729 | 710,84, |
| peak5984 | 1.57 | 0.00018197 | 0.002398833 | 16.7 | 7.24436E-09 | 1.04713E-07 | chr3 | 129389370 | 129546707 | NM_001349269.2 | 7.2E-09 | - | 129389370 | 129546707 | 737,62, |
| peak5987 | 1.68 | 3.54813E-05 | 0.000758578 | 12.9 | 1.09648E-07 | 1.07152E-06 | chr3 | 129389361 | 129546745 | NM_001349270.2 | 0.00000011 | - | 129389361 | 129546745 | 746,100, |
| peak5990 | 1.55 | 1.31826E-05 | 0.000354813 | 14.9 | 1.44544E-09 | 2.63027E-08 | chr3 | 129389379 | 129599223 | NM_001349271.2 | 1.4E-09 | - | 129389379 | 129599223 | 728,72, |
| peak5996 | 1.61 | 0.000107152 | 0.00162181 | 17.6 | 2.63027E-09 | 4.36516E-08 | chr3 | 129389401 | 129599187 | NM_001349276.2 | 2.6E-09 | - | 129389401 | 129599187 | 706,53,36, |
| peak5999 | 1.67 | 0.001202264 | 0.009549926 | 13.9 | 3.01995E-08 | 3.46737E-07 | chr3 | 134076382 | 134077408 | NM_001278683.1 | 0.00000003 | - | 134076382 | 134077408 | 223,27, |
| peak6002 | 1.83 | 0.000416869 | 0.004365158 | 10.1 | 1.69824E-08 | 2.13796E-07 | chr3 | 134076373 | 134077398 | NM_016201.4 | 0.000000017 | - | 134076373 | 134077398 | 232,17, |
| peak6005 | 1.76 | 0.001148154 | 0.009120108 | 10.9 | 0.000134896 | 0.00057544 | chr3 | 134076366 | 134077390 | NM_001363943.2 | 0.00013 | - | 134076366 | 134077390 | 239,9, |
| peak6016 | 2.28 | 0.004570882 | 0.023988329 | 8.45 | 7.76247E-05 | 0.000338844 | chr3 | 138374022 | 138374321 | NM_006219.3 | 0.000078 | - | 138374022 | 138374321 | 299, |
| peak6084 | 3.84 | 0.000123027 | 0.001778279 | 4.33 | 7.94328E-05 | 0.001659587 | chr3 | 170825935 | 170828636 | NM_001161560.3 | 0.000079 | - | 170825935 | 170828636 | 16,134, |
| peak6086 | 3.92 | 1.8197E-05 | 0.000457088 | 3.88 | 8.12831E-05 | 0.001698244 | chr3 | 170825917 | 170828619 | NM_001161563.3 | 0.000081 | - | 170825917 | 170828619 | 34,117, |
| peak6089 | 3.87 | 6.16595E-06 | 0.000199526 | 4 | 6.91831E-05 | 0.001479108 | chr3 | 170825933 | 170828634 | NM_001161566.3 | 0.000069 | - | 170825933 | 170828634 | 18,132, |
| peak6108 | 4.61 | 0.000446684 | 0.004570882 | 4.14 | 0.00128825 | 0.020417379 | chr3 | 184801137 | 184801237 | NM_001025266.3 | 0.0013 | - | 184801137 | 184801237 | 100, |
| peak6121 | 1.81 | 0.000316228 | 0.003548134 | 28 | 1.54882E-06 | 1.04713E-05 | chr3 | 194789695 | 194790631 | NM_001308069.1 | 0.0000015 | - | 194789695 | 194790631 | 936, |
| peak6122 | 1.75 | 0.00020893 | 0.002630268 | 29 | 4.67735E-07 | 3.63078E-06 | chr3 | 194789703 | 194790641 | NM_152531.5 | 0.00000047 | - | 194789703 | 194790641 | 938, |
| peak6188 | 2.13 | 0.000512861 | 0.005011872 | 23 | 2.81838E-05 | 0.000138038 | chr4 | 1980437 | 1981184 | NM_001042424.3 | 0.000028 | + | 1980437 | 1981184 | 747, |

| | | | | | | | | | | | | | | | |
|---|---|---|---|---|---|---|---|---|---|---|---|---|---|---|---|
| peak6195 | 2.03 | 0.000691831 | 0.006309573 | 23 | 3.31131E-05 | 0.000158489 | chr4 | 1980436 | 1981185 | NM_133330.2 | 0.000033 | + | 1980436 | 1981185 | 749, |
| peak6199 | 2.24 | 0.001148154 | 0.009120108 | 23.8 | 8.91251E-05 | 0.000389045 | chr4 | 1980449 | 1981097 | NM_133331.2 | 0.000089 | + | 1980449 | 1981097 | 648, |
| peak6203 | 2.07 | 0.00040738 | 0.004265795 | 29.6 | 6.91831E-05 | 0.00030903 | chr4 | 1980440 | 1981090 | NM_133335.3 | 0.000069 | + | 1980440 | 1981090 | 650, |
| peak6233 | 1.11 | 0.001202264 | 0.009549926 | 1.97 | 0.000114815 | 0.000489779 | chr4 | 2930151 | 2930351 | NM_001354754.2 | 0.00011 | + | 2930151 | 2930351 | 200, |
| peak6265 | 1.01 | 0.000489779 | 0.004897788 | 2.84 | 0.013489629 | 0.043651583 | chr4 | 2927817 | 2930025 | NM_001354755.1 | 0.013 | + | 2927817 | 2930025 | 22,128, |
| peak6295 | 2.99 | 3.31131E-06 | 0.000123027 | 4.18 | 0.000234423 | 0.000933254 | chr4 | 6865269 | 6865519 | NM_014743.3 | 0.00023 | + | 6865269 | 6865519 | 250, |
| peak6303 | 4.37 | 0.003801894 | 0.021379621 | 3.7 | 0.000134896 | 0.000562341 | chr4 | 6925622 | 6925773 | NM_020773.3 | 0.00013 | + | 6925622 | 6925773 | 151, |
| peak6336 | 3.25 | 0.002818383 | 0.017378008 | 4.7 | 5.88844E-05 | 0.000263027 | chr4 | 27024317 | 27024467 | NM_001169117.2 | 0.000059 | + | 27024317 | 27024467 | 150, |
| peak6340 | 3.4 | 0.004677351 | 0.024547089 | 4.09 | 2.95121E-05 | 0.000144544 | chr4 | 27024327 | 27024477 | NM_020860.4 | 0.00003 | + | 27024327 | 27024477 | 150, |
| peak6342 | 4.35 | 9.12011E-05 | 0.001479108 | 20.2 | 0.008709636 | 0.028183829 | chr4 | 30722488 | 30722788 | NM_001173523.2 | 0.0087 | + | 30722488 | 30722788 | 300, |
| peak6343 | 1.72 | 1.47911E-08 | 1.1749E-06 | 43.7 | 0.001348963 | 0.004786301 | chr4 | 30724233 | 30725978 | NM_001173523.2 | 0.0013 | + | 30724233 | 30725978 | 1745, |
| peak6345 | 4.43 | 7.58578E-05 | 0.00128825 | 17.6 | 0.006309573 | 0.021379621 | chr4 | 30722490 | 30722791 | NM_032457.4 | 0.0063 | + | 30722490 | 30722791 | 301, |
| peak6348 | 2.08 | 0.002344229 | 0.015135612 | 24.7 | 0.008709636 | 0.028840315 | chr4 | 30725839 | 30726040 | NM_032457.4 | 0.0087 | + | 30725839 | 30726040 | 201, |
| peak6374 | 3.16 | 0.003311311 | 0.019498446 | 15.2 | 8.70964E-06 | 4.7863E-05 | chr4 | 40121781 | 40121932 | NM_018177.6 | 0.0000087 | + | 40121781 | 40121932 | 151, |
| peak6383 | 1.5 | 0.002691535 | 0.016982437 | 5.86 | 1.09648E-07 | 1.04713E-06 | chr4 | 40752720 | 40752919 | NM_001330648.3 | 0.00000011 | + | 40752720 | 40752919 | 199, |
| peak6385 | 1.37 | 0.002691535 | 0.016595869 | 5.43 | 0.001230269 | 0.004466836 | chr4 | 40752722 | 40752873 | NM_024677.6 | 0.0012 | + | 40752722 | 40752873 | 151, |
| peak6405 | 1.99 | 0.001995262 | 0.013803843 | 2.67 | 0.000190546 | 0.000776247 | chr4 | 57344811 | 57350914 | NM_006947.4 | 0.00019 | + | 57344811 | 57350914 | 4,132,13, |
| peak6409 | 2.53 | 2.29087E-06 | 9.12011E-05 | 21.6 | 6.60693E-08 | 6.76083E-07 | chr4 | 57776928 | 57777277 | NM_005612.5 | 0.000000066 | + | 57776928 | 57777277 | 349, |
| peak6413 | 2.59 | 0.000467735 | 0.004677351 | 23.2 | 2.88403E-09 | 4.7863E-08 | chr4 | 57776948 | 57777298 | NM_001363453.2 | 2.9E-09 | + | 57776948 | 57777298 | 350, |
| peak6490 | 1.41 | 0.003981072 | 0.021877616 | 6.14 | 0.001148154 | 0.004168694 | chr4 | 106640377 | 106640576 | NM_001370181.1 | 0.0011 | + | 106640377 | 106640576 | 199, |
| peak6506 | 3.39 | 0.000660693 | 0.00616595 | 40.4 | 1.58489E-11 | 5.49541E-10 | chr4 | 110384117 | 110384367 | NM_006323.5 | 1.6E-11 | + | 110384117 | 110384367 | 250, |
| peak6693 | 2.31 | 0.002754229 | 0.016982437 | 18.8 | 2.51189E-06 | 1.62181E-05 | chr4 | 120213775 | 120214275 | NM_019050.2 | 0.0000025 | + | 120213775 | 120214275 | 500, |
| peak6741 | 3.14 | 0.000346737 | 0.003801894 | 6.29 | 3.54813E-05 | 0.000165959 | chr4 | 128811111 | 128811212 | NM_001190799.2 | 0.000035 | + | 128811111 | 128811212 | 101, |
| peak6743 | 3.14 | 0.000346737 | 0.003801894 | 6.29 | 1.86209E-05 | 9.33254E-05 | chr4 | 128811113 | 128811214 | NM_014264.5 | 0.000019 | + | 128811113 | 128811214 | 101, |
| peak6745 | 3.14 | 0.000346737 | 0.003801894 | 6.29 | 1.44544E-05 | 7.58578E-05 | chr4 | 128811110 | 128811211 | NM_001190801.1 | 0.000014 | + | 128811110 | 128811211 | 101, |
| peak6768 | 5.02 | 0.000436516 | 0.004570882 | 36 | 3.01995E-06 | 1.86209E-05 | chr4 | 144359341 | 144359641 | NM_002039.4 | 0.000003 | + | 144359341 | 144359641 | 300, |
| peak6773 | 5.02 | 0.000398107 | 0.004265795 | 41.1 | 4.0738E-06 | 2.45471E-05 | chr4 | 144359340 | 144359639 | NM_207123.3 | 0.0000041 | + | 144359340 | 144359639 | 299, |
| peak6801 | 2.54 | 2.0893E-05 | 0.000512861 | 6.71 | 3.23594E-06 | 1.99526E-05 | chr4 | 152571190 | 152571391 | NM_001109977.3 | 0.0000032 | + | 152571190 | 152571391 | 201, |
| peak6802 | 2.68 | 5.01187E-06 | 0.000169824 | 5.27 | 0.000301995 | 0.001202264 | chr4 | 152571097 | 152571397 | NM_001348694.2 | 0.0003 | + | 152571097 | 152571397 | 300, |
| peak6812 | 1.61 | 0.002041738 | 0.013803843 | 13 | 6.45654E-09 | 9.33254E-08 | chr4 | 154524995 | 154525344 | NM_015196.3 | 6.5E-09 | + | 154524995 | 154525344 | 349, |
| peak6813 | 1.6 | 0.001174898 | 0.009332543 | 12 | 1.31826E-08 | 1.7378E-07 | chr4 | 154524602 | 154525348 | NM_001131007.2 | 0.000000013 | + | 154524602 | 154525348 | 2,395, |
| peak6879 | 4.87 | 8.91251E-06 | 0.000263027 | 2.64 | 3.71535E-07 | 3.01995E-06 | chr4 | 184161374 | 184166678 | NM_024949.6 | 0.00000037 | + | 184161374 | 184166678 | 41,110, |
| peak6883 | 3.39 | 0.003630781 | 0.020417379 | 2.27 | 5.49541E-05 | 0.001230269 | chr4 | 184367822 | 184367923 | NM_017632.4 | 0.000055 | + | 184367822 | 184367923 | 101, |
| peak6886 | 5.23 | 0.002238721 | 0.014791084 | 2 | 9.54993E-07 | 3.80189E-05 | chr4 | 184426308 | 184426408 | NM_001564.4 | 0.00000095 | + | 184426308 | 184426408 | 100, |
| peak6905 | 2.25 | 0.00144544 | 0.010715193 | 6.4 | 3.54813E-06 | 2.13796E-05 | chr4 | 5823289 | 5827232 | NM_001313.5 | 0.0000035 | - | 5823289 | 5827232 | 289,12, |
| peak6907 | 2.13 | 0.003715352 | 0.020892961 | 5.4 | 8.91251E-05 | 0.000389045 | chr4 | 5823283 | 5827273 | NM_001288661.2 | 0.000089 | - | 5823283 | 5827273 | 295,53, |
| peak6909 | 2.13 | 0.003715352 | 0.020892961 | 5.4 | 7.24436E-05 | 0.000323594 | chr4 | 5823283 | 5827273 | NM_001014809.3 | 0.000072 | - | 5823283 | 5827273 | 295,53, |
| peak6913 | 3.47 | 0.00074131 | 0.006606934 | 5.29 | 0.001071519 | 0.003890451 | chr4 | 7764080 | 7764231 | NM_001371091.1 | 0.0011 | - | 7764080 | 7764231 | 151, |
| peak6916 | 1.73 | 0.001230269 | 0.009549926 | 2.94 | 0.001023293 | 0.003715352 | chr4 | 7802374 | 7817788 | NM_001371091.1 | 0.001 | - | 7802374 | 7817788 | 6,150,44, |
| peak6920 | 2.57 | 0.000234423 | 0.002818383 | 13.4 | 0.003467369 | 0.011748976 | chr4 | 8368602 | 8368702 | NM_003501.3 | 0.0035 | - | 8368602 | 8368702 | 100, |
| peak6923 | 2.46 | 0.001949845 | 0.013182567 | 8 | 6.0256E-08 | 6.30957E-07 | chr4 | 10446022 | 10446222 | NM_053042.3 | 0.00000006 | - | 10446022 | 10446222 | 200, |
| peak6937 | 1.71 | 0.001122018 | 0.009120108 | 19.8 | 1.20226E-06 | 8.31764E-06 | chr4 | 13615984 | 13616984 | NM_148894.3 | 0.0000012 | - | 13615984 | 13616984 | 450,49, |
| peak6949 | 1.03 | 0.00025704 | 0.003019952 | 5.25 | 4.67735E-06 | 0.000147911 | chr4 | 17886030 | 17887692 | NM_001365658.1 | 0.0000047 | - | 17886030 | 17887692 | 345,2, |
| peak6953 | 5.08 | 0.001819701 | 0.012589254 | 3.75 | 0.003235937 | 0.047863009 | chr4 | 36069665 | 36069765 | NM_015230.4 | 0.0032 | - | 36069665 | 36069765 | 100, |
| peak6954 | 2.69 | 0.001318257 | 0.01 | 5.4 | 0.002454709 | 0.036307805 | chr4 | 36230508 | 36230708 | NM_015230.4 | 0.0025 | - | 36230508 | 36230708 | 200, |
| peak7038 | 1.09 | 0.000331131 | 0.003715352 | 5.38 | 4.2658E-07 | 3.38844E-06 | chr4 | 73956611 | 73956960 | NM_001286771.3 | 0.00000043 | - | 73956611 | 73956960 | 349, |
| peak7049 | 1.18 | 1.62181E-05 | 0.00042658 | 4.55 | 7.58578E-07 | 5.62341E-06 | chr4 | 73956626 | 73956976 | NM_032217.5 | 0.00000076 | - | 73956626 | 73956976 | 350, |
| peak7075 | 4.56 | 6.60693E-05 | 0.001148154 | 3.91 | 4.16869E-07 | 3.31131E-06 | chr4 | 80827827 | 80827928 | NM_001286781.2 | 0.00000042 | - | 80827827 | 80827928 | 101, |
| peak7079 | 4.72 | 4.46684E-05 | 0.000891251 | 3.2 | 6.45654E-07 | 4.89779E-06 | chr4 | 80827828 | 80827929 | NM_058172.6 | 0.00000065 | - | 80827828 | 80827929 | 101, |
| peak7083 | 4.26 | 7.07946E-05 | 0.001230269 | 2.76 | 3.0903E-07 | 2.5704E-06 | chr4 | 80827831 | 80827932 | NM_001286780.2 | 0.00000031 | - | 80827831 | 80827932 | 101, |
| peak7105 | 1.09 | 0.003715352 | 0.020892961 | 45.5 | 5.62341E-09 | 8.51138E-08 | chr4 | 88415342 | 88416170 | NM_001291976.1 | 5.6E-09 | - | 88415342 | 88416170 | 408,38, |
| peak7109 | 1.13 | 0.001412538 | 0.010471285 | 45.8 | 1.34896E-07 | 1.25893E-06 | chr4 | 88415335 | 88416162 | NM_004684.5 | 0.00000013 | - | 88415335 | 88416162 | 415,30, |
| peak7141 | 1.92 | 0.001778279 | 0.012589254 | 11.6 | 5.7544E-09 | 8.51138E-08 | chr4 | 90170021 | 90170571 | NM_198281.3 | 5.8E-09 | - | 90170021 | 90170571 | 550, |
| peak7147 | 5.25 | 0.00047863 | 0.004897788 | 1.7 | 3.71535E-05 | 0.00017378 | chr4 | 100822227 | 100824950 | NM_001031723.4 | 0.000037 | - | 100822227 | 100824950 | 82,68, |
| peak7148 | 1.45 | 5.24807E-05 | 0.000977237 | 2.22 | 9.12011E-08 | 8.91251E-07 | chr4 | 101947104 | 101953508 | NM_001130691.2 | 0.000000091 | - | 101947104 | 101953508 | 114,85, |
| peak7149 | 1.48 | 0.00025704 | 0.003090295 | 2.3 | 0.000144544 | 0.00060256 | chr4 | 101947066 | 101961639 | NM_001130692.2 | 0.00014 | - | 101947066 | 101961639 | 152,98,1, |
| peak7165 | 1.34 | 0.003801894 | 0.021379621 | 9.75 | 3.01995E-05 | 0.000707946 | chr4 | 113199160 | 113199310 | NM_052864.3 | 0.00003 | - | 113199160 | 113199310 | 150, |

| | | | | | | | | | | | | | | | |
|---|---|---|---|---|---|---|---|---|---|---|---|---|---|---|---|
| peak7177 | 3.03 | 0.000144544 | 0.002041738 | 2 | 1.99526E-05 | 0.0001 | chr4 | 125631253 | 125631504 | NM_020337.3 | 0.00002 | - | 125631253 | 125631504 | 251, |
| peak7178 | 2.03 | 0.001348963 | 0.01023293 | 4.57 | 1.99526E-05 | 0.0001 | chr4 | 125631653 | 125631804 | NM_020337.3 | 0.00002 | - | 125631653 | 125631804 | 151, |
| peak7260 | 2.12 | 0.002630268 | 0.016595869 | 48.6 | 1.28825E-06 | 8.91251E-06 | chr4 | 186112038 | 186112237 | NM_001292033.2 | 0.0000013 | - | 186112038 | 186112237 | 199, |
| peak7285 | 2.04 | 0.000263027 | 0.003090295 | 1.58 | 0.011220185 | 0.036307805 | chr5 | 14368846 | 14368997 | NM_007118.4 | 0.011 | + | 14368846 | 14368997 | 151, |
| peak7288 | 2.03 | 0.001023293 | 0.00851138 | 1.71 | 0.012302688 | 0.039810717 | chr5 | 14368852 | 14369002 | NR_134469.2 | 0.012 | + | 14368852 | 14369002 | 150, |
| peak7361 | 1.63 | 0.002041738 | 0.013803843 | 5 | 2.51189E-06 | 1.62181E-05 | chr5 | 54461935 | 54462134 | NM_001306198.2 | 0.0000025 | + | 54461935 | 54462134 | 199, |
| peak7363 | 1.38 | 0.004786301 | 0.025118864 | 5 | 8.31764E-06 | 4.57088E-05 | chr5 | 54461892 | 54462090 | NM_001306201.2 | 0.0000083 | + | 54461892 | 54462090 | 198, |
| peak7365 | 1.89 | 0.000562341 | 0.005370318 | 5 | 9.12011E-08 | 8.91251E-07 | chr5 | 54461926 | 54462127 | NR_131336.2 | 0.000000091 | + | 54461926 | 54462127 | 201, |
| peak7372 | 1.67 | 0.001949845 | 0.013182567 | 5 | 1.07152E-07 | 1.02329E-06 | chr5 | 54461934 | 54462133 | NR_131340.2 | 0.00000011 | + | 54461934 | 54462133 | 199, |
| peak7404 | 3.28 | 0.000288403 | 0.003311311 | 4.64 | 3.63078E-05 | 0.000169824 | chr5 | 64867785 | 64867986 | NM_001278926.1 | 0.000036 | + | 64867785 | 64867986 | 201, |
| peak7405 | 3.42 | 0.004786301 | 0.025118864 | 8.78 | 8.12831E-06 | 4.46684E-05 | chr5 | 64867812 | 64867960 | NM_001278929.1 | 0.0000081 | + | 64867812 | 64867960 | 148, |
| peak7407 | 3.11 | 0.000323594 | 0.003630781 | 5 | 6.0256E-06 | 3.46737E-05 | chr5 | 64867798 | 64867948 | NM_001278927.2 | 0.000006 | + | 64867798 | 64867948 | 150, |
| peak7410 | 3.39 | 0.000213796 | 0.002691535 | 19.4 | 9.77237E-06 | 5.37032E-05 | chr5 | 65349471 | 65349720 | NM_001006600.2 | 0.0000098 | + | 65349471 | 65349720 | 249, |
| peak7414 | 1.38 | 0.001905461 | 0.013182567 | 8.82 | 1.14815E-06 | 8.12831E-06 | chr5 | 65349954 | 65350355 | NM_001253697.2 | 0.0000011 | + | 65349954 | 65350355 | 401, |
| peak7416 | 2.13 | 0.001995262 | 0.013803843 | 5.55 | 1.14815E-06 | 8.12831E-06 | chr5 | 65370953 | 65374377 | NM_001253697.2 | 0.0000011 | + | 65370953 | 65374377 | 105,93,75,127, |
| peak7418 | 1.6 | 0.003388442 | 0.019498446 | 9.91 | 5.62341E-07 | 4.2658E-06 | chr5 | 65349955 | 65350356 | NM_001253698.2 | 0.00000056 | + | 65349955 | 65350356 | 401, |
| peak7423 | 2.78 | 0.00017378 | 0.002290868 | 6.15 | 1.34896E-06 | 9.33254E-06 | chr5 | 65370918 | 65372768 | NM_001253699.2 | 0.0000013 | + | 65370918 | 65372768 | 140,93,66, |
| peak7425 | 1.29 | 0.002041738 | 0.013803843 | 8.82 | 4.46684E-07 | 3.54813E-06 | chr5 | 65349954 | 65350353 | NM_001253701.2 | 0.00000045 | + | 65349954 | 65350353 | 399, |
| peak7542 | 3.25 | 0.001 | 0.008317638 | 4 | 3.16228E-05 | 0.00074131 | chr5 | 79734393 | 79734493 | NM_001105251.4 | 0.000032 | + | 79734393 | 79734493 | 100, |
| peak7553 | 3.01 | 0.001819701 | 0.012882496 | 4 | 5.88844E-05 | 0.00128825 | chr5 | 79734393 | 79734494 | NR_146172.2 | 0.000059 | + | 79734393 | 79734494 | 101, |
| peak7560 | 3.25 | 0.001 | 0.008317638 | 4 | 6.60693E-05 | 0.001412538 | chr5 | 79734392 | 79734493 | NR_146174.2 | 0.000066 | + | 79734392 | 79734493 | 101, |
| peak7651 | 1.09 | 5.88844E-05 | 0.001071519 | 9.71 | 4.89779E-06 | 2.88403E-05 | chr5 | 96215637 | 96219518 | NM_022350.5 | 0.0000049 | + | 96215637 | 96219518 | 327,23, |
| peak7653 | 1.59 | 9.77237E-05 | 0.001548817 | 37.6 | 2.18776E-07 | 1.86209E-06 | chr5 | 96315145 | 96315496 | NM_005575.3 | 0.00000022 | + | 96315145 | 96315496 | 351, |
| peak7654 | 3.85 | 1.94984E-05 | 0.000489779 | 6 | 0.00042658 | 0.00162181 | chr5 | 96364528 | 96364729 | NM_005575.3 | 0.00043 | + | 96364528 | 96364729 | 201, |
| peak7655 | 1.74 | 7.76247E-05 | 0.00128825 | 33.8 | 1.09648E-07 | 1.04713E-06 | chr5 | 96315146 | 96315497 | NM_175920.4 | 0.00000011 | + | 96315146 | 96315497 | 351, |
| peak7656 | 3.85 | 1.94984E-05 | 0.000489779 | 6 | 0.000371535 | 0.00144544 | chr5 | 96364529 | 96364729 | NM_175920.4 | 0.00037 | + | 96364529 | 96364729 | 200, |
| peak7657 | 1.76 | 0.000645654 | 0.006025596 | 48.2 | 0.008128305 | 0.02630268 | chr5 | 98129161 | 98129262 | NM_001366510.1 | 0.0081 | + | 98129161 | 98129262 | 101, |
| peak7658 | 1.65 | 0.000676083 | 0.006309573 | 43.4 | 0.011220185 | 0.036307805 | chr5 | 98129158 | 98129259 | NM_001366509.1 | 0.011 | + | 98129158 | 98129259 | 101, |
| peak7659 | 1.68 | 0.001380384 | 0.010471285 | 45 | 0.014454398 | 0.046773514 | chr5 | 98129157 | 98129258 | NM_001366511.1 | 0.014 | + | 98129157 | 98129258 | 101, |
| peak7678 | 3.79 | 1.8197E-06 | 7.58578E-05 | 14.3 | 0.000380189 | 0.001479108 | chr5 | 112179444 | 112179844 | NM_001127511.3 | 0.00038 | + | 112179444 | 112179844 | 400, |
| peak7680 | 1.65 | 0.003019952 | 0.018197009 | 10.4 | 0.001348963 | 0.004786301 | chr5 | 112174387 | 112174588 | NM_001354895.2 | 0.0013 | + | 112174387 | 112174588 | 201, |
| peak7703 | 3.64 | 6.91831E-07 | 3.63078E-05 | 14.6 | 2.39883E-05 | 0.00011749 | chr5 | 112179487 | 112179838 | NM_001354902.2 | 0.000024 | + | 112179487 | 112179838 | 351, |
| peak7711 | 3.77 | 7.4131E-07 | 3.71535E-05 | 14.3 | 0.000239883 | 0.000954993 | chr5 | 112179446 | 112179845 | NM_000038.6 | 0.00024 | + | 112179446 | 112179845 | 399, |
| peak7715 | 3.26 | 0.001 | 0.008317638 | 6.9 | 0.003467369 | 0.012022644 | chr5 | 112176554 | 112176655 | NM_001127510.3 | 0.0035 | + | 112176554 | 112176655 | 101, |
| peak7726 | 3.77 | 7.4131E-07 | 3.71535E-05 | 14.3 | 5.01187E-06 | 2.88403E-05 | chr5 | 112179445 | 112179844 | NM_001354896.2 | 0.000005 | + | 112179445 | 112179844 | 399, |
| peak7729 | 3.27 | 0.000912011 | 0.007762471 | 6.9 | 0.004897788 | 0.016218101 | chr5 | 112176556 | 112176657 | NM_001354898.2 | 0.0049 | + | 112176556 | 112176657 | 101, |
| peak7732 | 3.69 | 1.65959E-06 | 7.07946E-05 | 14.3 | 5.12861E-06 | 2.95121E-05 | chr5 | 112179445 | 112179844 | NM_001354898.2 | 0.0000051 | + | 112179445 | 112179844 | 399, |
| peak7735 | 3.06 | 0.003467369 | 0.019952623 | 8.11 | 7.76247E-06 | 4.2658E-05 | chr5 | 112176551 | 112176751 | NM_001354899.2 | 0.0000078 | + | 112176551 | 112176751 | 200, |
| peak7746 | 3.77 | 7.4131E-07 | 3.71535E-05 | 14.3 | 0.000524807 | 0.001995262 | chr5 | 112179446 | 112179845 | NM_001354900.2 | 0.00052 | + | 112179446 | 112179845 | 399, |
| peak7755 | 3.74 | 7.4131E-07 | 3.71535E-05 | 14.3 | 0.000588844 | 0.002187762 | chr5 | 112179447 | 112179846 | NM_001354901.2 | 0.00059 | + | 112179447 | 112179846 | 399, |
| peak7760 | 3.74 | 7.4131E-07 | 3.71535E-05 | 14.3 | 6.45654E-06 | 3.71535E-05 | chr5 | 112179447 | 112179846 | NM_001354903.2 | 0.0000065 | + | 112179447 | 112179846 | 399, |
| peak7768 | 3.77 | 7.4131E-07 | 3.71535E-05 | 14.3 | 0.000281838 | 0.001122018 | chr5 | 112179446 | 112179845 | NM_001354904.2 | 0.00028 | + | 112179446 | 112179845 | 399, |
| peak7770 | 1.65 | 0.003019952 | 0.018197009 | 10.4 | 9.54993E-05 | 0.00040738 | chr5 | 112174387 | 112174588 | NM_001354905.2 | 0.000095 | + | 112174387 | 112174588 | 201, |
| peak8015 | 1.96 | 0.002089296 | 0.013803843 | 16.9 | 6.60693E-05 | 0.000295121 | chr5 | 139876222 | 139876673 | NM_017747.3 | 0.000066 | + | 139876222 | 139876673 | 451, |
| peak8019 | 2.43 | 0.001318257 | 0.01 | 14.1 | 0.000109648 | 0.000467735 | chr5 | 139946501 | 139946602 | NM_080670.3 | 0.00011 | + | 139946501 | 139946602 | 101, |
| peak8058 | 2.95 | 0.003019952 | 0.018197009 | 5.57 | 0.000301995 | 0.001174898 | chr5 | 149340519 | 149357317 | NM_000112.4 | 0.0003 | + | 149340519 | 149357317 | 23,127, |
| peak8063 | 1.49 | 0.003548134 | 0.020417379 | 1.9 | 0.005128614 | 0.017378008 | chr5 | 149776401 | 149778013 | NM_001135243.1 | 0.0051 | + | 149776401 | 149778013 | 4,97, |
| peak8073 | 1.66 | 3.80189E-05 | 0.000794328 | 90.4 | 1.99526E-11 | 6.16595E-10 | chr5 | 153432573 | 153433372 | NM_001242336.2 | 2E-11 | + | 153432573 | 153433372 | 799, |
| peak8075 | 1.77 | 0.000223872 | 0.002754229 | 86.6 | 5.01187E-11 | 1.51356E-09 | chr5 | 153432571 | 153433370 | NM_005927.5 | 5E-11 | + | 153432571 | 153433370 | 799, |
| peak8087 | 2.42 | 0.000776247 | 0.00691831 | 1.22 | 6.76083E-05 | 0.000301995 | chr5 | 154194625 | 154194726 | NM_001367713.1 | 0.000068 | + | 154194625 | 154194726 | 101, |
| peak8100 | 2.33 | 0.001202264 | 0.009549926 | 1.22 | 6.76083E-05 | 0.000301995 | chr5 | 154194626 | 154194726 | NM_001367715.1 | 0.000068 | + | 154194626 | 154194726 | 100, |
| peak8107 | 2.11 | 0.001995262 | 0.013489629 | 1.36 | 0.000114815 | 0.000489779 | chr5 | 154194625 | 154194725 | NM_001367716.1 | 0.00011 | + | 154194625 | 154194725 | 100, |
| peak8114 | 2.03 | 0.003019952 | 0.018197009 | 1.36 | 3.31131E-05 | 0.000158489 | chr5 | 154194624 | 154194725 | NM_001367717.1 | 0.000033 | + | 154194624 | 154194725 | 101, |
| peak8123 | 2.36 | 0.001047129 | 0.00851138 | 1.5 | 1.1749E-05 | 6.16595E-05 | chr5 | 154194621 | 154194722 | NM_033551.3 | 0.000012 | + | 154194621 | 154194722 | 101, |
| peak8137 | 2.36 | 0.001380384 | 0.01023293 | 2.42 | 2.88403E-05 | 0.000138038 | chr5 | 162939094 | 162940603 | NM_182796.2 | 0.000029 | + | 162939094 | 162940603 | 108,43, |
| peak8174 | 1.63 | 0.003981072 | 0.021877616 | 6.5 | 6.30957E-06 | 3.54813E-05 | chr5 | 176468833 | 176477718 | NM_001363713.1 | 0.0000063 | + | 176468833 | 176477718 | 38,145,15, |
| peak8178 | 3.19 | 7.76247E-10 | 7.24436E-08 | 44.2 | 6.0256E-05 | 0.000275423 | chr5 | 176636647 | 176637248 | NM_172349.2 | 0.00006 | + | 176636647 | 176637248 | 601, |

| | | | | | | | | | | | | | | | | |
|---|---|---|---|---|---|---|---|---|---|---|---|---|---|---|---|---|
| peak8184 | 1.07 | 0.000288403 | 0.003311311 | 71.3 | 2.0893E-09 | 3.63078E-08 | chr5 | 176720865 | 176722565 | NM_172349.2 | 2.1E-09 | + | 176720865 | 176722565 | 1700, |
| peak8186 | 1.02 | 0.002754229 | 0.016982437 | 30 | 5.24807E-06 | 3.0903E-05 | chr5 | 176562365 | 176562916 | NM_022455.4 | 0.0000052 | + | 176562365 | 176562916 | 551, |
| peak8193 | 1.09 | 0.000562341 | 0.005495409 | 76.3 | 7.58578E-09 | 1.07152E-07 | chr5 | 176720872 | 176722570 | NM_022455.4 | 7.6E-09 | + | 176720872 | 176722570 | 1698, |
| peak8201 | 1.08 | 0.000512861 | 0.005128614 | 76.2 | 4.0738E-09 | 6.30957E-08 | chr5 | 176720869 | 176722569 | NM_001365684.1 | 4.1E-09 | + | 176720869 | 176722569 | 1700, |
| peak8234 | 2.43 | 0.000131826 | 0.001862087 | 11.1 | 3.63078E-10 | 8.12831E-09 | chr5 | 476476 | 477498 | NM_004174.4 | 3.6E-10 | - | 476476 | 477498 | 17,130,52, |
| peak8252 | 1.24 | 8.91251E-06 | 0.000263027 | 30.7 | 9.12011E-09 | 1.28825E-07 | chr5 | 14710826 | 14711374 | NM_054027.6 | 9.1E-09 | - | 14710826 | 14711374 | 548, |
| peak8263 | 1.1 | 0.001513561 | 0.011220185 | 3.5 | 0.001348963 | 0.004786301 | chr5 | 31515113 | 31515314 | NM_013235.5 | 0.0013 | - | 31515113 | 31515314 | 201, |
| peak8266 | 1.07 | 0.000223872 | 0.002754229 | 9.14 | 8.12831E-08 | 8.12831E-07 | chr5 | 33527224 | 33527375 | NM_001324512.2 | 0.000000081 | - | 33527224 | 33527375 | 151, |
| peak8272 | 1.65 | 6.45654E-05 | 0.001148154 | 130 | 1.65959E-09 | 3.01995E-08 | chr5 | 35065067 | 35065466 | NM_001204314.2 | 1.7E-09 | - | 35065067 | 35065466 | 399, |
| peak8274 | 1.17 | 1.31826E-06 | 6.0256E-05 | 117 | 3.01995E-09 | 4.89779E-08 | chr5 | 35065088 | 35066136 | NM_000949.7 | 0.000000003 | - | 35065088 | 35066136 | 1048, |
| peak8275 | 1.56 | 0.001862087 | 0.012882496 | 23.8 | 2.75423E-06 | 1.7378E-05 | chr5 | 35065238 | 35066053 | NM_001204315.1 | 0.0000028 | - | 35065238 | 35066053 | 240,3, |
| peak8293 | 2.49 | 0.001659587 | 0.012022644 | 6.71 | 1.90546E-09 | 3.38844E-08 | chr5 | 43298715 | 43298915 | NM_001324220.2 | 1.9E-09 | - | 43298715 | 43298915 | 200, |
| peak8295 | 1.96 | 0.002630268 | 0.016595869 | 2.17 | 0.000316228 | 0.001258925 | chr5 | 43534980 | 43535643 | NM_183323.2 | 0.00032 | - | 43534980 | 43535643 | 92,8, |
| peak8299 | 6.55 | 6.45654E-05 | 0.001148154 | 15.7 | 3.63078E-05 | 0.00017378 | chr5 | 54527227 | 54527327 | NR_125348.1 | 0.000036 | - | 54527227 | 54527327 | 100, |
| peak8300 | 6.33 | 0.000489779 | 0.004897788 | 17.9 | 4.46684E-05 | 0.000204174 | chr5 | 54527222 | 54527320 | NM_021147.5 | 0.000045 | - | 54527222 | 54527320 | 98, |
| peak8301 | 6.59 | 7.07946E-05 | 0.001230269 | 15.7 | 2.5704E-05 | 0.000125893 | chr5 | 54527228 | 54527329 | NR_125346.1 | 0.000026 | - | 54527228 | 54527329 | 101, |
| peak8302 | 6.45 | 0.000251189 | 0.003019952 | 20.1 | 1.8197E-05 | 9.33254E-05 | chr5 | 54527225 | 54527324 | NR_125347.1 | 0.000018 | - | 54527225 | 54527324 | 99, |
| peak8336 | 5.92 | 6.91831E-10 | 6.45654E-08 | 10.7 | 1.44544E-07 | 1.34896E-06 | chr5 | 58270260 | 58270710 | NM_001197223.2 | 0.00000014 | - | 58270260 | 58270710 | 450, |
| peak8341 | 5.39 | 1.94984E-08 | 1.54882E-06 | 10.7 | 1.69824E-07 | 1.54882E-06 | chr5 | 58270261 | 58270712 | NM_001364603.1 | 0.00000017 | - | 58270261 | 58270712 | 451, |
| peak8418 | 1.84 | 0.000489779 | 0.004897788 | 9 | 8.91251E-06 | 4.7863E-05 | chr5 | 114604594 | 114607160 | NM_152549.2 | 0.0000089 | - | 114604594 | 114607160 | 103,96,251, |
| peak8419 | 1.84 | 0.000489779 | 0.004897788 | 9 | 3.46737E-05 | 0.000165959 | chr5 | 114604595 | 114607159 | NM_001040440.3 | 0.000035 | - | 114604595 | 114607159 | 102,96,250, |
| peak8450 | 3.42 | 2.5704E-07 | 1.54882E-05 | 32.5 | 0.000131826 | 0.000562341 | chr5 | 130766653 | 130766903 | NM_001164386.2 | 0.00013 | - | 130766653 | 130766903 | 250, |
| peak8454 | 3.09 | 4.67735E-05 | 0.000912011 | 32 | 5.12861E-06 | 3.01995E-05 | chr5 | 130764823 | 130766916 | NM_016340.6 | 0.0000051 | - | 130764823 | 130766916 | 86,365, |
| peak8459 | 2.44 | 2.04174E-06 | 8.31764E-05 | 15.3 | 1.31826E-05 | 6.91831E-05 | chr5 | 131008241 | 131008540 | NM_133372.3 | 0.000013 | - | 131008241 | 131008540 | 299, |
| peak8501 | 2.96 | 0.001584893 | 0.011481536 | 5.18 | 3.38844E-08 | 3.89045E-07 | chr5 | 137843224 | 137843573 | NM_001282185.1 | 0.000000034 | - | 137843224 | 137843573 | 349, |
| peak8507 | 2.86 | 6.0256E-05 | 0.001096478 | 5.18 | 1.09648E-07 | 1.04713E-06 | chr5 | 137843226 | 137843525 | NM_001256302.2 | 0.00000011 | - | 137843226 | 137843525 | 299, |
| peak8511 | 3.3 | 6.60693E-06 | 0.00020893 | 4.94 | 0.003090295 | 0.010471285 | chr5 | 137843315 | 137843514 | NM_001291975.2 | 0.0031 | - | 137843315 | 137843514 | 199, |
| peak8513 | 3.31 | 5.62341E-06 | 0.000186209 | 5.26 | 0.002187762 | 0.007585776 | chr5 | 137843316 | 137843515 | NM_001364160.2 | 0.0022 | - | 137843316 | 137843515 | 199, |
| peak8515 | 2.86 | 6.0256E-05 | 0.001096478 | 5.18 | 4.2658E-08 | 4.67735E-07 | chr5 | 137843226 | 137843525 | NM_004730.4 | 0.000000043 | - | 137843226 | 137843525 | 299, |
| peak8520 | 2.98 | 0.00047863 | 0.004786301 | 4.39 | 2.75423E-07 | 2.34423E-06 | chr5 | 137843279 | 137843529 | NR_157090.2 | 0.00000028 | - | 137843279 | 137843529 | 250, |
| peak8541 | 2.2 | 0.000691831 | 0.006309573 | 18.5 | 3.63078E-10 | 8.12831E-09 | chr5 | 141244432 | 141245668 | NM_001278615.1 | 3.6E-10 | - | 141244432 | 141245668 | 560,32, |
| peak8550 | 2.92 | 0.000954993 | 0.008128305 | 22.5 | 0.004365158 | 0.014791084 | chr5 | 141334898 | 141335098 | NM_016580.3 | 0.0044 | - | 141334898 | 141335098 | 200, |
| peak8628 | 2.96 | 0.004265795 | 0.023442288 | 4.14 | 2.88403E-05 | 0.000138038 | chr5 | 158630639 | 158634557 | NM_001199382.1 | 0.000029 | - | 158630639 | 158634557 | 25,75, |
| peak8630 | 2.96 | 0.001202264 | 0.009332543 | 2.85 | 0.000354813 | 0.001380384 | chr5 | 158630636 | 158634834 | NM_144726.2 | 0.00035 | - | 158630636 | 158634834 | 28,72, |
| peak8632 | 3.38 | 0.001698244 | 0.012022644 | 4.75 | 0.003715352 | 0.012589254 | chr5 | 158630653 | 158634982 | NM_001199381.2 | 0.0037 | - | 158630653 | 158634982 | 11,90, |
| peak8638 | 1.95 | 0.000489779 | 0.004897788 | 3.12 | 4.2658E-05 | 0.000199526 | chr5 | 167993138 | 167995667 | NM_024594.4 | 0.000043 | - | 167993138 | 167995667 | 133,17, |
| peak8657 | 1.12 | 0.003311311 | 0.019498446 | 18.3 | 1.65959E-06 | 1.12202E-05 | chr5 | 172195687 | 172195786 | NM_004417.4 | 0.0000017 | - | 172195687 | 172195786 | 99, |
| peak8679 | 2.19 | 1.41254E-05 | 0.000380189 | 1.75 | 0.005888437 | 0.019498446 | chr5 | 179315242 | 179318408 | NM_015043.4 | 0.0059 | - | 179315242 | 179318408 | 70,30, |
| peak8710 | 1.89 | 0.000338844 | 0.003715352 | 54.3 | 4.7863E-06 | 2.81838E-05 | chr5 | 180277819 | 180278413 | NM_001172638.1 | 0.0000048 | - | 180277819 | 180278413 | 594, |
| peak8718 | 2.81 | 0.000158489 | 0.002187762 | 25.5 | 2.29087E-05 | 0.000112202 | chr6 | 3114072 | 3114173 | NM_001317061.3 | 0.000023 | + | 3114072 | 3114173 | 101, |
| peak8721 | 2.82 | 0.000229087 | 0.002818383 | 24.9 | 6.76083E-05 | 0.000301995 | chr6 | 3114078 | 3114178 | NM_001354931.2 | 0.000068 | + | 3114078 | 3114178 | 100, |
| peak8724 | 2.82 | 0.000229087 | 0.002818383 | 24.9 | 7.24436E-05 | 0.000323594 | chr6 | 3114077 | 3114177 | NM_001354932.2 | 0.000072 | + | 3114077 | 3114177 | 100, |
| peak8727 | 2.89 | 7.07946E-05 | 0.001230269 | 24.2 | 7.94328E-06 | 4.36516E-05 | chr6 | 3114032 | 3114181 | NM_003804.6 | 0.0000079 | + | 3114032 | 3114181 | 149, |
| peak8730 | 2.93 | 7.4131E-05 | 0.001258925 | 23.5 | 6.91831E-06 | 3.89045E-05 | chr6 | 3114030 | 3114180 | NM_001354934.2 | 0.0000069 | + | 3114030 | 3114180 | 150, |
| peak8733 | 2.78 | 0.000158489 | 0.002137962 | 24.5 | 6.60693E-05 | 0.000295121 | chr6 | 3114073 | 3114174 | NM_001354930.2 | 0.000066 | + | 3114073 | 3114174 | 101, |
| peak8736 | 2.53 | 0.0001 | 0.001548817 | 24 | 1.54882E-05 | 7.94328E-05 | chr6 | 3114035 | 3114184 | NM_001354933.2 | 0.000015 | + | 3114035 | 3114184 | 149, |
| peak8778 | 2.7 | 0.000707946 | 0.006606934 | 11.7 | 0.005370318 | 0.017782794 | chr6 | 15520337 | 15520438 | NM_004973.4 | 0.0054 | + | 15520337 | 15520438 | 101, |
| peak8782 | 2.06 | 9.12011E-05 | 0.001479108 | 7.55 | 0.001258925 | 0.004570882 | chr6 | 15520323 | 15520474 | NM_001267040.1 | 0.0013 | + | 15520323 | 15520474 | 151, |
| peak8787 | 4.09 | 0.000141254 | 0.001995262 | 8.37 | 3.23594E-07 | 2.69153E-06 | chr6 | 20483071 | 20488348 | NM_001243076.2 | 0.00000032 | + | 20483071 | 20488348 | 80,115,5, |
| peak8788 | 1.59 | 0.000263027 | 0.003090295 | 8.5 | 3.23594E-07 | 2.69153E-06 | chr6 | 20488446 | 20491012 | NM_001243076.2 | 0.00000032 | + | 20488446 | 20491012 | 33,614, |
| peak8867 | 1.69 | 0.004168694 | 0.022908677 | 6.42 | 0.00047863 | 0.001819701 | chr6 | 36454727 | 36455077 | NR_104480.2 | 0.00048 | + | 36454727 | 36455077 | 350, |
| peak8879 | 4.6 | 0.003090295 | 0.018197009 | 7 | 2.63027E-06 | 1.65959E-05 | chr6 | 37336831 | 37336931 | NM_003958.4 | 0.0000026 | + | 37336831 | 37336931 | 100, |
| peak8881 | 4.94 | 0.000177828 | 0.002344229 | 7 | 9.77237E-07 | 7.07946E-06 | chr6 | 37336830 | 37336980 | NM_183078.3 | 0.00000098 | + | 37336830 | 37336980 | 150, |
| peak8888 | 2.62 | 0.00057544 | 0.005623413 | 8.88 | 1.8197E-07 | 1.62181E-06 | chr6 | 41066508 | 41066758 | NM_021705.4 | 0.00000018 | + | 41066508 | 41066758 | 250, |
| peak8945 | 2.37 | 0.001258925 | 0.009772372 | 7 | 8.70964E-05 | 0.000380189 | chr6 | 57012508 | 57012808 | NM_015555.2 | 0.000087 | + | 57012508 | 57012808 | 300, |
| peak8949 | 2.33 | 0.001698244 | 0.012022644 | 7.06 | 0.000138038 | 0.000588844 | chr6 | 57012513 | 57012813 | NM_001031623.3 | 0.00014 | + | 57012513 | 57012813 | 300, |
| peak8953 | 2.71 | 0.001148154 | 0.009120108 | 2.82 | 6.91831E-08 | 7.07946E-07 | chr6 | 64412428 | 64413475 | NM_001290259.2 | 0.000000069 | + | 64412428 | 64413475 | 101,50, |

| | | | | | | | | | | | | | | | | |
|---|---|---|---|---|---|---|---|---|---|---|---|---|---|---|---|---|
| peak8964 | 2.66 | 0.003388442 | 0.019498446 | 2.47 | 1.14815E-08 | 1.54882E-07 | chr6 | 64412438 | 64413485 | NM_001370349.2 | 0.000000011 | + | 64412438 | 64413485 | 91,60, | |
| peak9007 | 3.14 | 4.57088E-05 | 0.000891251 | 3.33 | 4.7863E-06 | 2.81838E-05 | chr6 | 87968219 | 87968370 | NM_001351444.2 | 0.0000048 | + | 87968219 | 87968370 | 151, | |
| peak9020 | 3.77 | 1.47911E-05 | 0.000398107 | 2.48 | 4.16869E-06 | 2.51189E-05 | chr6 | 87968216 | 87968367 | NM_015021.3 | 0.0000042 | + | 87968216 | 87968367 | 151, | |
| peak9030 | 2.28 | 0.000524807 | 0.005248075 | 12 | 1.69824E-05 | 8.70964E-05 | chr6 | 96034521 | 96044624 | NM_024641.4 | 0.000017 | + | 96034521 | 96044624 | 338,12, | |
| peak9084 | 2.33 | 0.002511886 | 0.015848932 | 29.8 | 5.7544E-07 | 4.36516E-06 | chr6 | 126210649 | 126210999 | NM_001199619.2 | 0.00000058 | + | 126210649 | 126210999 | 350, | |
| peak9090 | 2.4 | 0.001023293 | 0.008317638 | 33.8 | 1.86209E-05 | 9.33254E-05 | chr6 | 126210656 | 126211006 | NM_001122842.3 | 0.000019 | + | 126210656 | 126211006 | 350, | |
| peak9131 | 4.17 | 0.004677351 | 0.024547089 | 1.43 | 2.34423E-07 | 2.04174E-06 | chr6 | 142539730 | 142539831 | NM_016485.5 | 0.00000023 | + | 142539730 | 142539831 | 101, | |
| peak9146 | 1.11 | 5.88844E-05 | 0.001071519 | 6.37 | 1.20226E-07 | 1.14815E-06 | chr6 | 144033209 | 144086446 | NM_001100166.2 | 0.00000012 | + | 144033209 | 144086446 | 111,81,159,49, | |
| peak9161 | 1.33 | 1.1749E-06 | 5.49541E-05 | 4.91 | 2.95121E-07 | 2.45471E-06 | chr6 | 144033207 | 144086444 | NM_001100165.2 | 0.0000003 | + | 144033207 | 144086444 | 113,81,159,47, | |
| peak9293 | 2.11 | 0.000416869 | 0.004365158 | 22.2 | 2.5704E-10 | 6.16595E-09 | chr6 | 158733994 | 158734244 | NM_001007466.3 | 2.6E-10 | + | 158733994 | 158734244 | 250, | |
| peak9296 | 2.68 | 0.000213796 | 0.002691535 | 2.17 | 3.89045E-07 | 3.16228E-06 | chr6 | 158910669 | 158914610 | NM_001007466.3 | 0.00000039 | + | 158910669 | 158914610 | 95,6, | |
| peak9300 | 2.14 | 0.000346737 | 0.003890451 | 22.2 | 1.09648E-10 | 2.95121E-09 | chr6 | 158733995 | 158734245 | NM_020245.5 | 1.1E-10 | + | 158733995 | 158734245 | 250, | |
| peak9390 | 2.46 | 0.000151356 | 0.002137962 | 3.4 | 0.008709636 | 0.028183829 | chr6 | 3271556 | 3271707 | NR_104448.1 | 0.0087 | - | 3271556 | 3271707 | 151, | |
| peak9393 | 2.55 | 0.001071519 | 0.008709636 | 3.86 | 0.014125375 | 0.045708819 | chr6 | 3271553 | 3271654 | NM_015482.1 | 0.014 | - | 3271553 | 3271654 | 101, | |
| peak9404 | 1.29 | 0.004570882 | 0.024547089 | 29.9 | 1.09648E-09 | 2.0893E-08 | chr6 | 11185474 | 11185874 | NM_001271033.1 | 1.1E-09 | - | 11185474 | 11185874 | 400, | |
| peak9444 | 1.05 | 8.12831E-06 | 0.000251189 | 13.7 | 2.23872E-09 | 3.80189E-08 | chr6 | 35837324 | 35840394 | NR_034069.1 | 2.2E-09 | - | 35837324 | 35840394 | 354,214,26,55, | |
| peak9469 | 3.12 | 0.004786301 | 0.025118864 | 7.75 | 6.76083E-06 | 3.80189E-05 | chr6 | 44268066 | 44268167 | NM_020745.4 | 0.0000068 | - | 44268066 | 44268167 | 101, | |
| peak9474 | 1.51 | 0.001412538 | 0.010471285 | 14.5 | 3.46737E-07 | 2.88403E-06 | chr6 | 52129107 | 52129256 | NM_001270472.3 | 0.00000035 | - | 52129107 | 52129256 | 149, | |
| peak9477 | 1.51 | 0.001412538 | 0.010471285 | 14.5 | 2.51189E-07 | 2.13796E-06 | chr6 | 52129107 | 52129256 | NM_001366373.2 | 0.00000025 | - | 52129107 | 52129256 | 149, | |
| peak9535 | 1.72 | 0.002187762 | 0.014454398 | 39.3 | 7.76247E-08 | 7.76247E-07 | chr6 | 75893021 | 75893172 | NM_004370.6 | 0.000000078 | - | 75893021 | 75893172 | 151, | |
| peak9582 | 3.77 | 0.002691535 | 0.016595869 | 7.64 | 6.60693E-05 | 0.000295121 | chr6 | 90353615 | 90353766 | NM_014611.3 | 0.000066 | - | 90353615 | 90353766 | 151, | |
| peak9597 | 2.99 | 1E-16 | 1.58489E-14 | 42.5 | 5.7544E-06 | 3.31131E-05 | chr6 | 99728891 | 99729242 | NR_144463.2 | 0.0000058 | - | 99728891 | 99729242 | 351, | |
| peak9640 | 2.76 | 0.001047129 | 0.00851138 | 2.8 | 0.000158489 | 0.000660693 | chr6 | 101296436 | 101311992 | NM_006828.4 | 0.00016 | - | 101296436 | 101311992 | 147,53, | |
| peak9645 | 1.81 | 0.002290868 | 0.014791084 | 5.55 | 0.004570882 | 0.015135612 | chr6 | 108243052 | 108246110 | NM_007214.5 | 0.0046 | - | 108243052 | 108246110 | 61,89, | |
| peak9655 | 5.46 | 2.5704E-07 | 1.54882E-05 | 8.5 | 4.67735E-05 | 0.000218776 | chr6 | 110935529 | 110935730 | NM_015076.5 | 0.000047 | - | 110935529 | 110935730 | 201, | |
| peak9682 | 2.13 | 0.000275423 | 0.003162278 | 5.94 | 1.62181E-06 | 1.09648E-05 | chr6 | 114262898 | 114264584 | NM_001527.4 | 0.0000016 | - | 114262898 | 114264584 | 31,70, | |
| peak9683 | 2.35 | 5.01187E-05 | 0.000954993 | 5.85 | 9.77237E-07 | 7.07946E-06 | chr6 | 114262880 | 114264616 | NR_033441.2 | 0.00000098 | - | 114262880 | 114264616 | 49,102, | |
| peak9684 | 2.62 | 0.000138038 | 0.001949845 | 6.87 | 8.51138E-06 | 4.67735E-05 | chr6 | 114262240 | 114264644 | NR_073443.1 | 0.0000085 | - | 114262240 | 114264644 | 12,58,130, | |
| peak9756 | 2.92 | 0.001318257 | 0.01 | 10.7 | 0.004466836 | 0.015135612 | chr6 | 136742873 | 136847005 | NM_001198611.1 | 0.0045 | - | 136742873 | 136847005 | 64,36, | |
| peak9759 | 3.1 | 0.000524807 | 0.005128614 | 10.2 | 0.003890451 | 0.013182567 | chr6 | 136742875 | 136847007 | NM_001198614.1 | 0.0039 | - | 136742875 | 136847007 | 62,38, | |
| peak9762 | 2.65 | 0.00018197 | 0.002398833 | 6.38 | 0.002511886 | 0.008709636 | chr6 | 136742883 | 136847545 | NM_001198618.1 | 0.0025 | - | 136742883 | 136847545 | 54,46, | |
| peak9771 | 2.49 | 0.001122018 | 0.009120108 | 13.7 | 0.000281838 | 0.001122018 | chr6 | 136742867 | 136871510 | NM_001198617.1 | 0.00028 | - | 136742867 | 136871510 | 70,31, | |
| peak9783 | 2.19 | 3.0903E-06 | 0.00011749 | 10.4 | 4.67735E-05 | 0.000218776 | chr6 | 138752949 | 138753696 | NM_001144060.2 | 0.000047 | - | 138752949 | 138753696 | 747, | |
| peak9787 | 3.41 | 1.51356E-05 | 0.000398107 | 8.2 | 0.000398107 | 0.001513561 | chr6 | 138753308 | 138753559 | NM_020464.2 | 0.0004 | - | 138753308 | 138753559 | 251, | |
| peak9792 | 1.74 | 0.001412538 | 0.010471285 | 25.6 | 5.7544E-06 | 3.31131E-05 | chr6 | 143095692 | 143095943 | NM_006734.4 | 0.0000058 | - | 143095692 | 143095943 | 251, | |
| peak9804 | 2.49 | 0.000758578 | 0.00676083 | 12.3 | 6.91831E-06 | 3.89045E-05 | chr6 | 146264496 | 146264844 | NM_001370328.1 | 0.0000069 | - | 146264496 | 146264844 | 348, | |
| peak9805 | 1.45 | 0.002818383 | 0.017378008 | 19.2 | 8.51138E-05 | 0.000371535 | chr6 | 149983024 | 149983324 | NR_073033.1 | 0.000085 | - | 149983024 | 149983324 | 300, | |
| peak9811 | 1.99 | 8.51138E-05 | 0.001380384 | 19.2 | 1.94984E-06 | 1.28825E-05 | chr6 | 149983065 | 149983315 | NM_001350339.2 | 0.0000019 | - | 149983065 | 149983315 | 250, | LATS1 variant 4 |
| peak9817 | 1.99 | 8.51138E-05 | 0.001380384 | 19.2 | 2.39833E-06 | 1.54882E-05 | chr6 | 149983066 | 149983315 | NM_001350340.2 | 0.0000024 | - | 149983066 | 149983315 | 249, | LATS1 variant 5 |
| peak9823 | 2.04 | 0.001096478 | 0.008912509 | 19.2 | 1.1749E-05 | 6.30957E-05 | chr6 | 149983019 | 149983318 | NM_001350392.2 | 0.000012 | - | 149983019 | 149983318 | 299, | LATS1 variant 6 |
| peak9829 | 2.03 | 0.001513561 | 0.011220185 | 19.2 | 8.91251E-06 | 4.7863E-05 | chr6 | 149983018 | 149983318 | NM_004690.4 | 0.0000089 | - | 149983018 | 149983318 | 300, | LATS1 variant 1 |
| peak9881 | 2.18 | 4.0738E-05 | 0.000831764 | 1.68 | 3.23594E-05 | 0.000154882 | chr7 | 878503 | 881610 | NM_001367687.1 | 0.000032 | + | 878503 | 881610 | 120,28, | |
| peak9883 | 2.1 | 0.000154882 | 0.002137962 | 1.35 | 0.001479108 | 0.005248075 | chr7 | 878503 | 878603 | NM_001171944.2 | 0.0015 | + | 878503 | 878603 | 100, | |
| peak9885 | 2.19 | 0.00042658 | 0.004466836 | 1.52 | 0.001737801 | 0.00616595 | chr7 | 878504 | 878604 | NM_001367633.1 | 0.0017 | + | 878504 | 878604 | 100, | |
| peak9889 | 2.26 | 0.000416869 | 0.004365158 | 1.52 | 0.005370318 | 0.017782794 | chr7 | 878504 | 878605 | NM_001367635.1 | 0.0054 | + | 878504 | 878605 | 101, | |
| peak9891 | 2.47 | 2.18776E-05 | 0.000524807 | 1.78 | 0.004786301 | 0.015848932 | chr7 | 878502 | 881611 | NM_001367636.1 | 0.0048 | + | 878502 | 881611 | 121,29, | |
| peak9895 | 2.14 | 6.76083E-05 | 0.001174898 | 1.35 | 0.004570882 | 0.015488166 | chr7 | 878504 | 881612 | NM_001367643.1 | 0.0046 | + | 878504 | 881612 | 119,30, | |
| peak9896 | 2.47 | 2.18776E-05 | 0.000524807 | 1.78 | 0.000190546 | 0.000776247 | chr7 | 878502 | 881611 | NM_001367647.1 | 0.00019 | + | 878502 | 881611 | 121,29, | |
| peak9898 | 2.61 | 9.12011E-05 | 0.00144544 | 1.86 | 6.16595E-06 | 3.54813E-05 | chr7 | 878513 | 878614 | NM_001367651.1 | 0.0000062 | + | 878513 | 878614 | 101, | |
| peak9900 | 2.26 | 0.000416869 | 0.004365158 | 1.52 | 8.70964E-05 | 0.000380189 | chr7 | 878504 | 878605 | NM_001367653.1 | 0.000087 | + | 878504 | 878605 | 101, | |
| peak9902 | 2.47 | 2.18776E-05 | 0.000524807 | 1.78 | 0.000676083 | 0.002570396 | chr7 | 878502 | 881611 | NM_001367658.1 | 0.00068 | + | 878502 | 881611 | 121,29, | |
| peak9904 | 2.47 | 2.18776E-05 | 0.000524807 | 1.78 | 4.2658E-07 | 3.38844E-06 | chr7 | 878502 | 881611 | NM_001367662.1 | 0.00000043 | + | 878502 | 881611 | 121,29, | |
| peak9907 | 2.19 | 0.00042658 | 0.004466836 | 1.52 | 8.70964E-05 | 0.000380189 | chr7 | 878504 | 878604 | NM_001367665.1 | 0.000087 | + | 878504 | 878604 | 100, | |
| peak9910 | 2.51 | 4.0738E-06 | 0.000144544 | 1.78 | 1.47911E-07 | 1.38038E-06 | chr7 | 878502 | 881610 | NM_001367668.1 | 0.00000015 | + | 878502 | 881610 | 121,28, | |
| peak9913 | 2.47 | 2.18776E-05 | 0.000524807 | 1.78 | 4.16869E-06 | 2.45471E-05 | chr7 | 878502 | 881611 | NM_001367670.1 | 0.0000042 | + | 878502 | 881611 | 121,29, | |
| peak9916 | 2.49 | 1.90546E-05 | 0.00047863 | 1.78 | 5.01187E-08 | 5.37032E-07 | chr7 | 878503 | 878604 | NM_001367673.1 | 0.00000005 | + | 878503 | 878604 | 101, | |
| peak9918 | 2.19 | 0.00042658 | 0.004466836 | 1.52 | 0.001122018 | 0.004073803 | chr7 | 878504 | 878604 | NM_001367674.1 | 0.0011 | + | 878504 | 878604 | 100, | |

| | | | | | | | | | | | | | | | |
|---|---|---|---|---|---|---|---|---|---|---|---|---|---|---|---|
| peak9920 | 2.19 | 0.00042658 | 0.004466836 | 1.52 | 0.001 | 0.003630781 | chr7 | 878504 | 878604 | NM_001367675.1 | 0.001 | + | 878504 | 878604 | 100, |
| peak9921 | 2.26 | 0.000416869 | 0.004365158 | 1.52 | 0.000870964 | 0.003235937 | chr7 | 878504 | 878605 | NM_001367677.1 | 0.00087 | + | 878504 | 878605 | 101, |
| peak9923 | 2.1 | 0.000154882 | 0.002137962 | 1.35 | 2.0893E-05 | 0.000104713 | chr7 | 878504 | 878604 | NM_001367678.1 | 0.000021 | + | 878504 | 878604 | 100, |
| peak9924 | 2.49 | 1.90546E-05 | 0.00047863 | 1.78 | 1.34896E-07 | 1.25893E-06 | chr7 | 878503 | 878604 | NM_001367680.1 | 0.00000013 | + | 878503 | 878604 | 101, |
| peak9926 | 2.47 | 2.18776E-05 | 0.000524807 | 1.78 | 6.76083E-05 | 0.000301995 | chr7 | 878502 | 881611 | NM_001367681.1 | 0.000068 | + | 878502 | 881611 | 121,29, |
| peak9929 | 2.49 | 1.90546E-05 | 0.00047863 | 1.78 | 3.63078E-05 | 0.00017378 | chr7 | 878503 | 878604 | NM_001367686.1 | 0.000036 | + | 878503 | 878604 | 101, |
| peak9932 | 2.14 | 6.76083E-05 | 0.001174898 | 1.35 | 0.000144544 | 0.000616595 | chr7 | 878504 | 881612 | NM_001367690.1 | 0.00014 | + | 878504 | 881612 | 119,30, |
| peak9933 | 2.51 | 4.0738E-06 | 0.000144544 | 1.78 | 0.000229087 | 0.000933254 | chr7 | 878502 | 881610 | NM_001367691.1 | 0.00023 | + | 878502 | 881610 | 121,28, |
| peak9935 | 2.19 | 0.00042658 | 0.004466836 | 1.52 | 6.60693E-07 | 5.01187E-06 | chr7 | 878504 | 878604 | NM_001367693.1 | 0.00000066 | + | 878504 | 878604 | 100, |
| peak9937 | 2.1 | 0.000154882 | 0.002137962 | 1.35 | 6.91831E-06 | 3.89045E-05 | chr7 | 878504 | 878604 | NM_001367694.1 | 0.0000069 | + | 878504 | 878604 | 100, |
| peak9939 | 2.14 | 6.76083E-05 | 0.001174898 | 1.35 | 0.000001 | 7.24436E-06 | chr7 | 878504 | 881612 | NM_001367697.1 | 0.000001 | + | 878504 | 881612 | 119,30, |
| peak9942 | 2.19 | 0.00042658 | 0.004466836 | 1.52 | 0.008317638 | 0.027542287 | chr7 | 878504 | 878604 | NM_001367699.1 | 0.0083 | + | 878504 | 878604 | 100, |
| peak9945 | 2.48 | 6.45654E-05 | 0.001148154 | 1.78 | 0.001202264 | 0.004365158 | chr7 | 878502 | 878602 | NM_001367701.1 | 0.0012 | + | 878502 | 878602 | 100, |
| peak9950 | 2.51 | 4.0738E-06 | 0.000144544 | 1.78 | 0.012589254 | 0.040738028 | chr7 | 878502 | 881610 | NM_001367706.1 | 0.013 | + | 878502 | 881610 | 121,28, |
| peak9952 | 2.47 | 2.18776E-05 | 0.000524807 | 1.78 | 3.98107E-05 | 0.000186209 | chr7 | 878502 | 881611 | NM_025154.6 | 0.00004 | + | 878502 | 881611 | 121,29, |
| peak9954 | 2.1 | 0.000154882 | 0.002137962 | 1.35 | 0.000457088 | 0.001737801 | chr7 | 878504 | 878604 | NR_160281.1 | 0.00046 | + | 878504 | 878604 | 100, |
| peak9955 | 2.19 | 0.00042658 | 0.004466836 | 1.52 | 5.88844E-05 | 0.000263027 | chr7 | 878504 | 878604 | NR_160283.1 | 0.000059 | + | 878504 | 878604 | 100, |
| peak9974 | 1.3 | 0.001513561 | 0.011220185 | 23.4 | 4.36516E-05 | 0.000204174 | chr7 | 4806375 | 4807125 | NM_001037165.2 | 0.000044 | + | 4806375 | 4807125 | 750, |
| peak10013 | 6.68 | 0.000489779 | 0.004897788 | 4.8 | 0.001148154 | 0.018197009 | chr7 | 21550707 | 21550807 | NM_001326542.2 | 0.0011 | + | 21550707 | 21550807 | 100, |
| peak10016 | 5.83 | 0.000776247 | 0.00691831 | 3.4 | 0.002089296 | 0.031622777 | chr7 | 21550694 | 21550795 | NM_001326543.2 | 0.0021 | + | 21550694 | 21550795 | 101, |
| peak10183 | 1.38 | 0.002951209 | 0.018197009 | 3.8 | 0.001122018 | 0.004073803 | chr7 | 77200480 | 77212922 | NM_001131008.2 | 0.0011 | + | 77200480 | 77212922 | 23,77,51, |
| peak10206 | 4.04 | 5.12861E-05 | 0.000977237 | 6.4 | 0.001548817 | 0.005495409 | chr7 | 91632047 | 91632247 | NM_005751.4 | 0.0015 | + | 91632047 | 91632247 | 200, |
| peak10256 | 6.32 | 2.51189E-05 | 0.000588844 | 2 | 4.67735E-08 | 5.01187E-07 | chr7 | 99170551 | 99170651 | NM_138494.3 | 0.000000047 | + | 99170551 | 99170651 | 100, |
| peak10257 | 6.27 | 2.95121E-05 | 0.000660693 | 1.88 | 0.000346737 | 0.001348963 | chr7 | 99170553 | 99170653 | NM_001009960.1 | 0.00035 | + | 99170553 | 99170653 | 100, |
| peak10258 | 6.32 | 2.51189E-05 | 0.000588844 | 2 | 0.000354813 | 0.001380384 | chr7 | 99170548 | 99170649 | NM_001085368.1 | 0.00035 | + | 99170548 | 99170649 | 101, |
| peak10260 | 6.32 | 2.51189E-05 | 0.000588844 | 2 | 0.000288403 | 0.001122018 | chr7 | 99170552 | 99170652 | NM_001363332.1 | 0.00029 | + | 99170552 | 99170652 | 100, |
| peak10275 | 2.85 | 3.98107E-05 | 0.000812831 | 7.4 | 0.000346737 | 0.001348963 | chr7 | 99633974 | 99634125 | NM_001287055.2 | 0.00035 | + | 99633974 | 99634125 | 151, |
| peak10283 | 2.87 | 3.23594E-05 | 0.000707946 | 7.4 | 0.000977237 | 0.003630781 | chr7 | 99633972 | 99634122 | NM_001287054.3 | 0.00098 | + | 99633972 | 99634122 | 150, |
| peak10290 | 3.08 | 1.86209E-05 | 0.000467735 | 7.65 | 0.001949845 | 0.00691831 | chr7 | 99633933 | 99634133 | NM_001346580.2 | 0.0019 | + | 99633933 | 99634133 | 200, |
| peak10297 | 2.37 | 7.4131E-06 | 0.000229087 | 7.91 | 0.002290868 | 0.007943282 | chr7 | 99633977 | 99634128 | NM_001346581.2 | 0.0023 | + | 99633977 | 99634128 | 151, |
| peak10304 | 2.69 | 5.62341E-05 | 0.001047129 | 9.88 | 0.003801894 | 0.012882496 | chr7 | 99634038 | 99634138 | NM_003439.4 | 0.0038 | + | 99634038 | 99634138 | 100, |
| peak10311 | 2.38 | 0.000630957 | 0.006025596 | 9.88 | 0.00144544 | 0.005128614 | chr7 | 99634037 | 99634138 | NR_144477.2 | 0.0014 | + | 99634037 | 99634138 | 101, |
| peak10350 | 2.44 | 0.003548134 | 0.020417379 | 15.5 | 8.91251E-08 | 8.70964E-07 | chr7 | 102087433 | 102088033 | NM_032831.4 | 0.000000089 | + | 102087433 | 102088033 | 600, |
| peak10365 | 2.25 | 0.00017378 | 0.002290868 | 13.7 | 1E-13 | 6.30957E-12 | chr7 | 107398628 | 107399575 | NM_001284291.1 | 1E-13 | + | 107398628 | 107399575 | 947, |
| peak10366 | 2.2 | 0.00162181 | 0.011748976 | 13.5 | 6.30957E-15 | 6.30957E-13 | chr7 | 107398646 | 107399588 | NR_024199.2 | 6.3E-15 | + | 107398646 | 107399588 | 942, |
| peak10367 | 2.15 | 0.00040738 | 0.004365158 | 13.2 | 3.98107E-13 | 2.51189E-11 | chr7 | 107398623 | 107399621 | NM_024814.4 | 4E-13 | + | 107398623 | 107399621 | 998, |
| peak10397 | 1.61 | 0.002691535 | 0.016982437 | 3.18 | 6.60693E-05 | 0.000295121 | chr7 | 132959825 | 132973774 | NM_021807.4 | 0.000066 | + | 132959825 | 132973774 | 101,99, |
| peak10432 | 4.18 | 3.01995E-05 | 0.000676083 | 7 | 1.02329E-05 | 0.000281838 | chr7 | 150028026 | 150028324 | NM_138434.2 | 0.00001 | + | 150028026 | 150028324 | 298, |
| peak10464 | 7.34 | 3.71535E-07 | 2.13796E-05 | 8 | 1.62181E-07 | 8.31764E-06 | chr7 | 155567744 | 155567994 | NM_053043.3 | 0.00000016 | + | 155567744 | 155567994 | 250, |
| peak10493 | 1.68 | 0.003890451 | 0.021877616 | 5.36 | 0.001905461 | 0.006606934 | chr7 | 5541072 | 5541272 | NM_001367781.1 | 0.0019 | - | 5541072 | 5541272 | 200, |
| peak10539 | 5.56 | 0.001174898 | 0.009120108 | 6.75 | 7.94328E-06 | 4.46684E-05 | chr7 | 22985406 | 22985507 | NM_001363466.2 | 0.0000079 | - | 22985406 | 22985507 | 101, |
| peak10541 | 4.19 | 0.001122018 | 0.009120108 | 5.2 | 6.60693E-05 | 0.00144544 | chr7 | 22985359 | 22985560 | NM_032581.4 | 0.000066 | - | 22985359 | 22985560 | 201, |
| peak10549 | 1.18 | 0.003467369 | 0.019952623 | 1.9 | 0.012302688 | 0.039810717 | chr7 | 37951851 | 37953846 | NM_003014.4 | 0.012 | - | 37951851 | 37953846 | 68,32, |
| peak10555 | 2.9 | 0.000102329 | 0.001584893 | 16.2 | 3.98107E-08 | 4.46684E-07 | chr7 | 42005370 | 42005868 | NM_000168.6 | 0.00000004 | - | 42005370 | 42005868 | 498, |
| peak10577 | 2.2 | 3.98107E-05 | 0.000812831 | 8 | 0.000223872 | 0.000912011 | chr7 | 50514011 | 50514312 | NM_001287492.3 | 0.00022 | - | 50514011 | 50514312 | 301, |
| peak10579 | 2.1 | 3.80189E-06 | 0.000134896 | 4.75 | 0.000223872 | 0.000912011 | chr7 | 50514611 | 50515894 | NM_001287492.3 | 0.00022 | - | 50514611 | 50515894 | 384,16, |
| peak10589 | 1.58 | 0.002398833 | 0.015488166 | 24.9 | 1.58489E-05 | 8.12831E-05 | chr7 | 55538842 | 55539138 | NM_001321251.1 | 0.000016 | - | 55538842 | 55539138 | 296, |
| peak10590 | 1.84 | 5.37032E-05 | 0.001 | 5.47 | 0.001047129 | 0.003801894 | chr7 | 55539334 | 55539483 | NM_001321251.1 | 0.001 | - | 55539334 | 55539483 | 149, |
| peak10593 | 1.85 | 8.91251E-05 | 0.00144544 | 13 | 0.00060256 | 0.002290868 | chr7 | 55538849 | 55539100 | NM_001321242.1 | 0.0006 | - | 55538849 | 55539100 | 251, |
| peak10597 | 1.96 | 0.000239883 | 0.002884032 | 15.2 | 0.002238721 | 0.007762471 | chr7 | 55538895 | 55539095 | NM_001284284.1 | 0.0022 | - | 55538895 | 55539095 | 200, |
| peak10602 | 1.75 | 4.2658E-05 | 0.000851138 | 5.42 | 0.002089296 | 0.007413102 | chr7 | 55539335 | 55539484 | NM_001321248.1 | 0.0021 | - | 55539335 | 55539484 | 149, |
| peak10605 | 1.86 | 0.000162181 | 0.002187762 | 18.1 | 0.002290868 | 0.008128305 | chr7 | 55538894 | 55539093 | NM_001321250.1 | 0.0023 | - | 55538894 | 55539093 | 199, |
| peak10609 | 1.55 | 5.37032E-05 | 0.001023293 | 13 | 0.004365158 | 0.014454398 | chr7 | 55538850 | 55539101 | NM_001321249.1 | 0.0044 | - | 55538850 | 55539101 | 251, |
| peak10610 | 2.13 | 0.002137962 | 0.014125375 | 5.94 | 0.005623413 | 0.018620871 | chr7 | 55539350 | 55539451 | NM_001321249.1 | 0.0056 | - | 55539350 | 55539451 | 101, |
| peak10613 | 1.65 | 0.000446684 | 0.004570882 | 15.2 | 0.001122018 | 0.004073803 | chr7 | 55538847 | 55539095 | NM_001284283.2 | 0.0011 | - | 55538847 | 55539095 | 248, |
| peak10617 | 2.04 | 0.000162181 | 0.002187762 | 13 | 0.001071519 | 0.003890451 | chr7 | 55538899 | 55539098 | NM_001284282.2 | 0.0011 | - | 55538899 | 55539098 | 199, |
| peak10620 | 1.53 | 5.7544E-05 | 0.001071519 | 13 | 0.00074131 | 0.002754229 | chr7 | 55538851 | 55539100 | NM_001321243.2 | 0.00074 | - | 55538851 | 55539100 | 249, |

| | | | | | | | | | | | | | | | |
|---|---|---|---|---|---|---|---|---|---|---|---|---|---|---|---|
| peak10624 | 1.65 | 0.000446684 | 0.004570882 | 15.2 | 0.001412538 | 0.005011872 | chr7 | 55538847 | 55539095 | NM_001321246.2 | 0.0014 | - | 55538847 | 55539095 | 248, |
| peak10628 | 2.11 | 0.000295121 | 0.003388442 | 14.6 | 0.005370318 | 0.017782794 | chr7 | 55538904 | 55539104 | NM_001321247.2 | 0.0054 | - | 55538904 | 55539104 | 200, |
| peak10629 | 2.17 | 0.001230269 | 0.009549926 | 6.73 | 0.011220185 | 0.036307805 | chr7 | 55539353 | 55539453 | NM_001321247.2 | 0.011 | - | 55539353 | 55539453 | 100, |
| peak10632 | 1.73 | 2.69153E-06 | 0.000104713 | 16.5 | 1.51356E-07 | 1.38038E-06 | chr7 | 55538853 | 55539451 | NM_030796.5 | 0.00000015 | - | 55538853 | 55539451 | 598, |
| peak10663 | 2.68 | 0.002398833 | 0.015488166 | 3 | 1.69824E-10 | 2.63027E-08 | chr7 | 91503775 | 91503975 | NM_001301134.2 | 1.7E-10 | - | 91503775 | 91503975 | 200, |
| peak10664 | 4.94 | 0.004570882 | 0.024547089 | 3.5 | 9.33254E-08 | 5.24807E-06 | chr7 | 91503734 | 91503835 | NM_001301135.2 | 0.000000093 | - | 91503734 | 91503835 | 101, |
| peak10676 | 1.96 | 0.004073803 | 0.022387211 | 13 | 0.007413102 | 0.024547089 | chr7 | 92762615 | 92762766 | NM_001303496.3 | 0.0074 | - | 92762615 | 92762766 | 151, |
| peak10691 | 1.85 | 0.001148154 | 0.009120108 | 17.3 | 0.012022644 | 0.038904514 | chr7 | 92761962 | 92762113 | NM_001350083.2 | 0.012 | - | 92761962 | 92762113 | 151, |
| peak10696 | 1.85 | 0.000446684 | 0.004570882 | 17.3 | 0.011220185 | 0.036307805 | chr7 | 92761964 | 92762115 | NM_001350085.2 | 0.011 | - | 92761964 | 92762115 | 151, |
| peak10742 | 2.63 | 0.002041738 | 0.013803843 | 10.5 | 8.31764E-05 | 0.000363078 | chr7 | 99758289 | 99758437 | NM_024637.5 | 0.000083 | - | 99758289 | 99758437 | 148, |
| peak10825 | 3.03 | 9.77237E-06 | 0.000288403 | 11.8 | 9.54993E-06 | 5.24807E-05 | chr7 | 135078865 | 135079163 | NM_001190847.2 | 0.0000095 | - | 135078865 | 135079163 | 298, |
| peak10828 | 3.14 | 6.76083E-07 | 3.54813E-05 | 12.4 | 1.07152E-05 | 5.7544E-05 | chr7 | 135078860 | 135079158 | NM_013316.4 | 0.000011 | - | 135078860 | 135079158 | 298, |
| peak10871 | 1.65 | 0.002570396 | 0.016218101 | 23.3 | 2.69153E-05 | 0.000131826 | chr7 | 148768410 | 148769201 | NM_152411.4 | 0.000027 | - | 148768410 | 148769201 | 791, |
| peak10892 | 4.96 | 4.89779E-06 | 0.000165959 | 6.33 | 7.94328E-05 | 0.000354813 | chr7 | 151874323 | 151874474 | NM_170606.3 | 0.000079 | - | 151874323 | 151874474 | 151, |
| peak10894 | 2.64 | 0.000301995 | 0.003467369 | 4.13 | 0.000144544 | 0.00060256 | chr7 | 151877094 | 151877829 | NM_170606.3 | 0.00014 | - | 151877094 | 151877829 | 117,34, |
| peak10895 | 1.9 | 0.000794328 | 0.007079458 | 4.71 | 0.000144544 | 0.00060256 | chr7 | 151878327 | 151878427 | NM_170606.3 | 0.00014 | - | 151878327 | 151878427 | 100, |
| peak10900 | 1.14 | 0.004570882 | 0.024547089 | 7.29 | 8.12831E-05 | 0.000363078 | chr7 | 151900036 | 151917745 | NM_170606.3 | 0.000081 | - | 151900036 | 151917745 | 113,120,129,138, |
| peak10906 | 4.55 | 2.95121E-08 | 2.23872E-06 | 9.5 | 1.12202E-07 | 1.07152E-06 | chr7 | 156474582 | 156474833 | NM_001350954.2 | 0.00000011 | - | 156474582 | 156474833 | 251, |
| peak10910 | 1.08 | 0.003890451 | 0.021379621 | 4.56 | 0.002041738 | 0.007079458 | chr7 | 156476473 | 156476723 | NM_001350957.2 | 0.002 | - | 156476473 | 156476723 | 250, |
| peak10911 | 1.21 | 0.003311311 | 0.019498446 | 4.2 | 0.00144544 | 0.005248075 | chr7 | 156476607 | 156476707 | NM_001350958.2 | 0.0014 | - | 156476607 | 156476707 | 100, |
| peak10912 | 1.12 | 0.003548134 | 0.020417379 | 5.22 | 0.00128825 | 0.004570882 | chr7 | 156476474 | 156476725 | NM_001363410.2 | 0.0013 | - | 156476474 | 156476725 | 251, |
| peak10913 | 4.55 | 2.95121E-08 | 2.23872E-06 | 9.5 | 5.88844E-08 | 6.30957E-07 | chr7 | 156474582 | 156474833 | NM_001363411.2 | 0.000000059 | - | 156474582 | 156474833 | 251, |
| peak10916 | 4.33 | 2.29087E-08 | 1.7378E-06 | 9.5 | 7.94328E-08 | 7.94328E-07 | chr7 | 156474581 | 156474832 | NM_001363413.2 | 0.000000079 | - | 156474581 | 156474832 | 251, |
| peak10917 | 1.34 | 0.002754229 | 0.017378008 | 6.37 | 0.00018197 | 0.000758578 | chr7 | 156476430 | 156476731 | NM_001363413.2 | 0.00018 | - | 156476430 | 156476731 | 301, |
| peak10919 | 4.33 | 1.69824E-08 | 1.34896E-06 | 8.4 | 4.7863E-08 | 5.12861E-07 | chr7 | 156474583 | 156474834 | NM_022458.4 | 0.000000048 | - | 156474583 | 156474834 | 251, |
| peak10922 | 4.33 | 2.29087E-08 | 1.7378E-06 | 9.5 | 1.31826E-07 | 1.23027E-06 | chr7 | 156474581 | 156474832 | NR_146958.2 | 0.00000013 | - | 156474581 | 156474832 | 251, |
| peak11146 | 2.48 | 0.000114815 | 0.001698244 | 5.85 | 7.94328E-05 | 0.000346737 | chr8 | 38704244 | 38705728 | NM_001146216.3 | 0.000079 | + | 38704244 | 38705728 | 93,256, |
| peak11148 | 2.57 | 3.54813E-05 | 0.000758578 | 6.53 | 3.98107E-08 | 4.46684E-07 | chr8 | 38704236 | 38705720 | NM_001330521.2 | 0.00000004 | + | 38704236 | 38705720 | 101,248, |
| peak11150 | 2.48 | 0.001318257 | 0.01 | 6.97 | 2.23872E-06 | 1.44544E-05 | chr8 | 38704217 | 38705753 | NM_001352778.2 | 0.0000022 | + | 38704217 | 38705753 | 120,281, |
| peak11153 | 2.56 | 2.39883E-05 | 0.00057544 | 5.86 | 1.20226E-08 | 1.58489E-07 | chr8 | 38704243 | 38705727 | NM_001352780.2 | 0.000000012 | + | 38704243 | 38705727 | 94,255, |
| peak11155 | 2.46 | 6.60693E-05 | 0.001148154 | 6.48 | 2.29087E-08 | 2.75423E-07 | chr8 | 38704235 | 38705719 | NM_001352785.2 | 0.000000023 | + | 38704235 | 38705719 | 102,247, |
| peak11157 | 2.61 | 1.09648E-05 | 0.00030903 | 7.12 | 1.04713E-05 | 5.62341E-05 | chr8 | 38704233 | 38705718 | NM_001352786.2 | 0.00001 | + | 38704233 | 38705718 | 104,246, |
| peak11159 | 2.53 | 3.38844E-05 | 0.00074131 | 5.86 | 2.0893E-08 | 2.5704E-07 | chr8 | 38704243 | 38705727 | NM_001352787.2 | 0.000000021 | + | 38704243 | 38705727 | 94,255, |
| peak11161 | 2.48 | 0.000269153 | 0.003162278 | 6.82 | 3.63078E-06 | 2.23872E-05 | chr8 | 38704219 | 38705754 | NM_001352788.2 | 0.0000036 | + | 38704219 | 38705754 | 118,282, |
| peak11164 | 2.45 | 0.000812831 | 0.00724436 | 8.95 | 3.46737E-07 | 2.81838E-06 | chr8 | 38704221 | 38705756 | NM_001352789.2 | 0.00000035 | + | 38704221 | 38705756 | 116,284, |
| peak11166 | 2.33 | 0.003311311 | 0.019498446 | 6.22 | 2.23872E-06 | 1.44544E-05 | chr8 | 38700908 | 38705747 | NM_001352792.2 | 0.0000022 | + | 38700908 | 38705747 | 5,121,275, |
| peak11169 | 2.46 | 0.001023293 | 0.008317638 | 9.41 | 5.24807E-07 | 4.0738E-06 | chr8 | 38704223 | 38705758 | NM_001352793.2 | 0.00000052 | + | 38704223 | 38705758 | 114,286, |
| peak11171 | 2.33 | 0.003311311 | 0.019498446 | 6.22 | 1.94984E-06 | 1.28825E-05 | chr8 | 38700908 | 38705747 | NM_001352794.2 | 0.0000019 | + | 38700908 | 38705747 | 5,121,275, |
| peak11173 | 2.33 | 0.003311311 | 0.019498446 | 6.22 | 2.45471E-06 | 1.58489E-05 | chr8 | 38700908 | 38705747 | NM_001352795.2 | 0.0000025 | + | 38700908 | 38705747 | 5,121,275, |
| peak11175 | 2.45 | 0.000199526 | 0.002570396 | 5.94 | 2.5704E-08 | 3.01995E-07 | chr8 | 38704238 | 38705722 | NM_001352799.2 | 0.000000026 | + | 38704238 | 38705722 | 99,250, |
| peak11176 | 2.5 | 0.000281838 | 0.003235937 | 9.41 | 1.31826E-06 | 9.12011E-06 | chr8 | 38704224 | 38705759 | NR_148049.2 | 0.0000013 | + | 38704224 | 38705759 | 113,287, |
| peak11177 | 2.46 | 6.60693E-05 | 0.001148154 | 6.48 | 5.88844E-08 | 6.16595E-07 | chr8 | 38704235 | 38705719 | NR_148051.2 | 0.000000059 | + | 38704235 | 38705719 | 102,247, |
| peak11180 | 2.34 | 0.001096478 | 0.008912509 | 6.44 | 1.25893E-06 | 8.70964E-06 | chr8 | 38700910 | 38705749 | NM_001352782.2 | 0.0000013 | + | 38700910 | 38705749 | 3,121,277, |
| peak11182 | 2.32 | 4.57088E-05 | 0.000912011 | 5.41 | 3.98107E-08 | 4.46684E-07 | chr8 | 38704239 | 38705723 | NM_001352784.2 | 0.00000004 | + | 38704239 | 38705723 | 98,251, |
| peak11185 | 2.52 | 0.000213796 | 0.002691535 | 9.41 | 2.13796E-07 | 1.86209E-06 | chr8 | 38704224 | 38705759 | NM_001352790.2 | 0.00000021 | + | 38704224 | 38705759 | 113,287, |
| peak11188 | 2.31 | 0.002754229 | 0.016982437 | 8.75 | 4.16869E-07 | 3.31131E-06 | chr8 | 38700912 | 38705751 | NM_001352796.2 | 0.00000042 | + | 38700912 | 38705751 | 1,121,279, |
| peak11192 | 2.43 | 0.000891251 | 0.007762471 | 9.02 | 9.12011E-08 | 8.91251E-07 | chr8 | 38704228 | 38705713 | NM_001352804.2 | 0.000000091 | + | 38704228 | 38705713 | 109,241, |
| peak11195 | 2.45 | 0.000288403 | 0.003311311 | 6.8 | 1.04713E-06 | 7.4131E-06 | chr8 | 38704219 | 38705754 | NM_001122824.2 | 0.000001 | + | 38704219 | 38705754 | 118,282, |
| peak11198 | 2.33 | 8.51138E-05 | 0.001380384 | 6.13 | 1.44544E-08 | 1.86209E-07 | chr8 | 38704240 | 38705724 | NM_001352779.2 | 0.000000014 | + | 38704240 | 38705724 | 97,252, |
| peak11201 | 2.28 | 0.00011749 | 0.001737801 | 7.62 | 4.16869E-08 | 4.67735E-07 | chr8 | 38704231 | 38705716 | NM_001352781.2 | 0.000000042 | + | 38704231 | 38705716 | 106,244, |
| peak11204 | 2.47 | 0.000794328 | 0.00691831 | 9.41 | 2.75423E-07 | 2.34423E-06 | chr8 | 38704222 | 38705757 | NM_001352783.2 | 0.00000028 | + | 38704222 | 38705757 | 115,285, |
| peak11205 | 2.55 | 0.000158489 | 0.002137962 | 6.82 | 7.07946E-07 | 5.37032E-06 | chr8 | 38704220 | 38705755 | NM_001352801.2 | 0.00000071 | + | 38704220 | 38705755 | 117,283, |
| peak11206 | 2.45 | 7.58578E-05 | 0.00128825 | 5.48 | 2.04174E-08 | 2.51189E-07 | chr8 | 38704248 | 38705732 | NM_001352803.2 | 0.00000002 | + | 38704248 | 38705732 | 89,260, |
| peak11209 | 2.39 | 0.000107152 | 0.00162181 | 7.58 | 5.01187E-08 | 5.37032E-07 | chr8 | 38704231 | 38705715 | NM_006283.3 | 0.00000005 | + | 38704231 | 38705715 | 106,243, |
| peak11211 | 2.51 | 4.57088E-05 | 0.000891251 | 6.48 | 1.58489E-08 | 2.04174E-07 | chr8 | 38704235 | 38705719 | NM_001352798.1 | 0.000000016 | + | 38704235 | 38705719 | 102,247, |
| peak11214 | 2.48 | 0.001318257 | 0.01 | 6.97 | 1.58489E-06 | 1.07152E-05 | chr8 | 38704217 | 38705753 | NM_001352791.1 | 0.0000016 | + | 38704217 | 38705753 | 120,281, |
| peak11221 | 2.29 | 0.004897788 | 0.025703958 | 26.4 | 1.09648E-10 | 2.95121E-09 | chr8 | 41455831 | 41456432 | NM_178819.4 | 1.1E-10 | + | 41455831 | 41456432 | 601, |

| | | | | | | | | | | | | | | | |
|---|---|---|---|---|---|---|---|---|---|---|---|---|---|---|---|
| peak11224 | 2.31 | 6.91831E-06 | 0.000218776 | 34 | 3.89045E-09 | 6.16595E-08 | chr8 | 41455895 | 41456494 | NM_001363197.2 | 3.9E-09 | + | 41455895 | 41456494 | 599, |
| peak11251 | 1.04 | 1.51356E-05 | 0.000398107 | 10 | 7.07946E-09 | 1.02329E-07 | chr8 | 42852756 | 42873631 | NM_032410.4 | 7.1E-09 | + | 42852756 | 42873631 | 24,35,83,101,105,72,131, |
| peak11264 | 1.17 | 1.99526E-13 | 1.99526E-11 | 9.47 | 2.95121E-08 | 3.46737E-07 | chr8 | 61654072 | 61707622 | NM_017780.4 | 0.00000003 | + | 61654072 | 61707622 | 1584,431,78, |
| peak11268 | 2.37 | 0.000512861 | 0.005011872 | 20.2 | 4.89779E-05 | 0.000223872 | chr8 | 61777896 | 61778545 | NM_017780.4 | 0.000049 | + | 61777896 | 61778545 | 649, |
| peak11269 | 2.29 | 4.67735E-07 | 2.5704E-05 | 10.2 | 0.000251189 | 0.001 | chr8 | 61654115 | 61654762 | NM_001316690.1 | 0.00025 | + | 61654115 | 61654762 | 647, |
| peak11308 | 2.47 | 0.000977237 | 0.008128305 | 4.43 | 2.23872E-10 | 5.49541E-09 | chr8 | 81412019 | 81412169 | NM_001105539.3 | 2.2E-10 | + | 81412019 | 81412169 | 150, |
| peak11320 | 3.31 | 8.31764E-05 | 0.001348963 | 1.88 | 0.012882496 | 0.041686938 | chr8 | 94929273 | 94929373 | NM_001161779.1 | 0.013 | + | 94929273 | 94929373 | 100, |
| peak11322 | 2.14 | 3.16228E-06 | 0.00011749 | 26.3 | 3.98107E-15 | 3.98107E-13 | chr8 | 97345695 | 97346242 | NM_001290225.2 | 4E-15 | + | 97345695 | 97346242 | 547, |
| peak11323 | 2.55 | 4.46684E-06 | 0.000158489 | 28.5 | 5.49541E-08 | 5.7544E-07 | chr8 | 97345730 | 97346279 | NM_014754.3 | 0.000000055 | + | 97345730 | 97346279 | 549, |
| peak11361 | 2.39 | 2.88403E-06 | 0.000109648 | 17.5 | 5.01187E-16 | 6.30957E-14 | chr8 | 110576698 | 110577093 | NM_198120.2 | 5E-16 | + | 110576698 | 110577093 | 395, |
| peak11362 | 2.46 | 0.000691831 | 0.006309573 | 21.6 | 1.99526E-10 | 5.01187E-09 | chr8 | 110567086 | 110577084 | NM_004215.5 | 2E-10 | + | 110567086 | 110577084 | 30,108,92,417, |
| peak11363 | 2.21 | 0.002630268 | 0.016595869 | 17.4 | 1.25893E-11 | 4.46684E-10 | chr8 | 110567079 | 110577079 | NM_001278938.1 | 1.3E-11 | + | 110567079 | 110577079 | 37,108,92,412, |
| peak11381 | 2.09 | 4.89779E-06 | 0.000169824 | 35.9 | 0.000776247 | 0.002884032 | chr8 | 126449196 | 126449547 | NM_025195.4 | 0.00078 | + | 126449196 | 126449547 | 351, |
| peak11384 | 1.05 | 1.1749E-05 | 0.000331131 | 42.2 | 7.94328E-15 | 7.94328E-13 | chr8 | 128752666 | 128753411 | NM_001354870.1 | 7.9E-15 | + | 128752666 | 128753411 | 745, |
| peak11386 | 1.05 | 1.14815E-05 | 0.000323594 | 42.2 | 3.16228E-16 | 3.98107E-14 | chr8 | 128752666 | 128753411 | NM_002467.6 | 3.2E-16 | + | 128752666 | 128753411 | 745, |
| peak11408 | 3.76 | 0.000147911 | 0.002041738 | 12.6 | 0.000141254 | 0.000588844 | chr8 | 144732237 | 144732386 | NM_001261843.2 | 0.00014 | + | 144732237 | 144732386 | 149, |
| peak11412 | 2.62 | 6.0256E-05 | 0.001096478 | 12.8 | 0.000891251 | 0.003235937 | chr8 | 144732153 | 144732753 | NM_014789.3 | 0.00089 | + | 144732153 | 144732753 | 600, |
| peak11490 | 2.42 | 0.001737801 | 0.012302688 | 6.08 | 0.001548817 | 0.005495409 | chr8 | 16021613 | 16021713 | NM_001363744.1 | 0.0015 | - | 16021613 | 16021713 | 100, |
| peak11491 | 2.68 | 0.001071519 | 0.008709636 | 5.12 | 0.000630957 | 0.002398833 | chr8 | 16021605 | 16021705 | NM_138715.3 | 0.00063 | - | 16021605 | 16021705 | 100, |
| peak11493 | 1.4 | 0.000162181 | 0.002187762 | 18.3 | 3.98107E-18 | 7.94328E-16 | chr8 | 16026109 | 16026308 | NM_002445.4 | 4E-18 | - | 16026109 | 16026308 | 199, |
| peak11519 | 1.06 | 0.002818383 | 0.017378008 | 7.07 | 2.75423E-09 | 4.57088E-08 | chr8 | 17503478 | 17504568 | NM_001001931.2 | 2.8E-09 | - | 17503478 | 17504568 | 170,78, |
| peak11528 | 1.06 | 0.001096478 | 0.008912509 | 6.24 | 2.75423E-05 | 0.000131826 | chr8 | 17503487 | 17504578 | NM_001001925.2 | 0.000028 | - | 17503487 | 17504578 | 161,88, |
| peak11568 | 5.51 | 1.09648E-05 | 0.00030903 | 20.6 | 0.008709636 | 0.028183829 | chr8 | 18729745 | 18729945 | NM_015310.4 | 0.0087 | - | 18729745 | 18729945 | 200, |
| peak11629 | 5.32 | 1.99526E-05 | 0.000489779 | 10 | 0.000001 | 7.24436E-06 | chr8 | 33369668 | 33369816 | NM_025115.4 | 0.000001 | - | 33369668 | 33369816 | 148, |
| peak11631 | 2.15 | 0.003715352 | 0.020892961 | 39.9 | 3.63078E-08 | 4.0738E-07 | chr8 | 37732461 | 37732562 | NM_001002814.3 | 0.000000036 | - | 37732461 | 37732562 | 101, |
| peak11633 | 2.15 | 0.003715352 | 0.020892961 | 39.9 | 1.02329E-06 | 7.24436E-06 | chr8 | 37732463 | 37732563 | NM_025151.5 | 0.000001 | - | 37732463 | 37732563 | 100, |
| peak11638 | 3.41 | 0.004786301 | 0.025118864 | 12 | 0.00020893 | 0.000851138 | chr8 | 38133352 | 38133966 | NM_023034.2 | 0.00021 | - | 38133352 | 38133966 | 48,153, |
| peak11728 | 3.55 | 0.000724436 | 0.006606934 | 12.5 | 4.36516E-06 | 2.63027E-05 | chr8 | 62577863 | 62578063 | NM_020164.5 | 0.0000044 | - | 62577863 | 62578063 | 200, |
| peak11729 | 3.55 | 0.000724436 | 0.006606934 | 12.5 | 3.80189E-06 | 2.34423E-05 | chr8 | 62577867 | 62578067 | NM_032467.4 | 0.0000038 | - | 62577867 | 62578067 | 200, |
| peak11731 | 1.25 | 0.002570396 | 0.016218101 | 50.6 | 7.94328E-15 | 6.30957E-13 | chr8 | 66630863 | 66631713 | NM_002603.4 | 7.9E-15 | - | 66630863 | 66631713 | 850, |
| peak11732 | 1.43 | 0.002884032 | 0.017378008 | 47.9 | 3.98107E-14 | 3.16228E-12 | chr8 | 66630893 | 66631690 | NM_001242318.3 | 4E-14 | - | 66630893 | 66631690 | 797, |
| peak11734 | 1.98 | 6.30957E-05 | 0.001122018 | 35.4 | 3.46737E-07 | 2.81838E-06 | chr8 | 67576926 | 67577226 | NM_025054.5 | 0.00000035 | - | 67576926 | 67577226 | 300, |
| peak11735 | 3.26 | 5.88844E-05 | 0.001071519 | 13.7 | 4.2658E-06 | 2.5704E-05 | chr8 | 67577623 | 67577823 | NM_025054.5 | 0.0000043 | - | 67577623 | 67577823 | 200, |
| peak11746 | 4.59 | 1E-16 | 1.58489E-14 | 14.6 | 1.58489E-18 | 3.16228E-16 | chr8 | 75156898 | 75227362 | NM_001363050.1 | 1.6E-18 | - | 75156898 | 75227362 | 512,119,267, |
| peak11748 | 6.55 | 3.98107E-07 | 2.23872E-05 | 7.75 | 0.000630957 | 0.002398833 | chr8 | 75171693 | 75227150 | NM_001317830.2 | 0.00063 | - | 75171693 | 75227150 | 45,55, |
| peak11769 | 3.42 | 0.004677351 | 0.025118864 | 4.57 | 0.002238721 | 0.033113112 | chr8 | 81550575 | 81550676 | NM_001033723.3 | 0.0022 | - | 81550575 | 81550676 | 101, |
| peak11775 | 1.2 | 0.003235937 | 0.019054607 | 9.67 | 1.23027E-05 | 6.45654E-05 | chr8 | 81888888 | 81889139 | NM_018440.4 | 0.000012 | - | 81888888 | 81889139 | 251, |
| peak11882 | 1.28 | 0.000758578 | 0.00676083 | 8.68 | 0.000120226 | 0.000512861 | chr8 | 118811799 | 118811899 | NM_000127.2 | 0.00012 | - | 118811799 | 118811899 | 100, |
| peak11895 | 2.07 | 0.000128825 | 0.001862087 | 10.9 | 8.12831E-08 | 8.12831E-07 | chr8 | 124262472 | 124266316 | NM_001017926.2 | 0.000000081 | - | 124262472 | 124266316 | 88,755, |
| peak11896 | 1.27 | 0.000794328 | 0.007079458 | 11.5 | 1.94984E-07 | 1.69824E-06 | chr8 | 124266513 | 124267207 | NM_001017926.2 | 0.00000019 | - | 124266513 | 124267207 | 694, |
| peak11897 | 2.33 | 0.001122018 | 0.009120108 | 11.5 | 1.94984E-07 | 1.69824E-06 | chr8 | 124267305 | 124267752 | NM_001017926.2 | 0.00000019 | - | 124267305 | 124267752 | 447, |
| peak11898 | 4.06 | 1.8197E-05 | 0.000457088 | 28.6 | 4.89779E-07 | 3.80189E-06 | chr8 | 124268147 | 124268396 | NM_001017926.2 | 0.00000049 | - | 124268147 | 124268396 | 249, |
| peak11900 | 4.49 | 1.1749E-07 | 7.76247E-06 | 5.79 | 5.7544E-07 | 4.46684E-06 | chr8 | 124266133 | 124266334 | NR_037873.1 | 0.00000058 | - | 124266133 | 124266334 | 201, |
| peak11901 | 1.29 | 0.003981072 | 0.022387211 | 8.75 | 2.04174E-07 | 1.77828E-06 | chr8 | 124266532 | 124267181 | NR_037873.1 | 0.0000002 | - | 124266532 | 124267181 | 649, |
| peak11902 | 2.17 | 0.001862087 | 0.012882496 | 12.7 | 2.04174E-07 | 1.77828E-06 | chr8 | 124267280 | 124267730 | NR_037873.1 | 0.0000002 | - | 124267280 | 124267730 | 450, |
| peak11903 | 4.02 | 9.12011E-05 | 0.001479108 | 19 | 3.98107E-07 | 3.16228E-06 | chr8 | 124268178 | 124279529 | NR_037873.1 | 0.0000004 | - | 124268178 | 124279529 | 233,17, |
| peak11905 | 4.54 | 1.62181E-07 | 1.04713E-05 | 3.35 | 2.45471E-07 | 2.0893E-06 | chr8 | 124266145 | 124266346 | NM_007222.5 | 0.00000025 | - | 124266145 | 124266346 | 201, |
| peak11906 | 1.33 | 0.000281838 | 0.003235937 | 11.9 | 3.01995E-07 | 2.51189E-06 | chr8 | 124266545 | 124267196 | NM_007222.5 | 0.0000003 | - | 124266545 | 124267196 | 651, |
| peak11907 | 2.29 | 0.001479108 | 0.010715193 | 12.1 | 3.01995E-07 | 2.51189E-06 | chr8 | 124267295 | 124267745 | NM_007222.5 | 0.0000003 | - | 124267295 | 124267745 | 450, |
| peak11908 | 4.06 | 5.49541E-05 | 0.001023293 | 29.1 | 0.000812831 | 0.003019952 | chr8 | 124268144 | 124268395 | NM_007222.5 | 0.00081 | - | 124268144 | 124268395 | 251, |
| peak11999 | 1.03 | 0.003019952 | 0.018197009 | 2.22 | 0.00128825 | 0.004677351 | chr8 | 131165007 | 131172165 | NM_001362924.1 | 0.0013 | - | 131165007 | 131172165 | 44,56, |
| peak12002 | 1.05 | 0.000524807 | 0.005128614 | 1.98 | 0.000645654 | 0.002398833 | chr8 | 131165012 | 131172171 | NM_001362925.2 | 0.00065 | - | 131165012 | 131172171 | 39,62, |
| peak12008 | 1.01 | 0.000524807 | 0.005128614 | 2.24 | 0.001659587 | 0.005888437 | chr8 | 131165011 | 131172169 | NM_018482.4 | 0.0017 | - | 131165011 | 131172169 | 40,60, |
| peak12022 | 3.71 | 0.002570396 | 0.016218101 | 7.14 | 9.33254E-06 | 5.01187E-05 | chr8 | 141460937 | 141461288 | NM_001160372.4 | 0.0000093 | - | 141460937 | 141461288 | 351, |
| peak12026 | 3.68 | 0.000467735 | 0.004677351 | 7.36 | 4.46684E-06 | 2.69153E-05 | chr8 | 141449248 | 141461339 | NM_001321646.2 | 0.0000045 | - | 141449248 | 141461339 | 48,451, |
| peak12029 | 3.69 | 0.002630268 | 0.016595869 | 7 | 4.2658E-06 | 2.5704E-05 | chr8 | 141460931 | 141461282 | NM_031466.7 | 0.0000043 | - | 141460931 | 141461282 | 351, |
| peak12154 | 1.79 | 6.0256E-05 | 0.001096478 | 53.5 | 1.90546E-10 | 4.7863E-09 | chr8 | 144671295 | 144679536 | NM_001130053.4 | 1.9E-10 | - | 144671295 | 144679536 | 956,14,19, |

| | | | | | | | | | | | | | | | |
|---|---|---|---|---|---|---|---|---|---|---|---|---|---|---|---|
| peak12156 | 1.87 | 6.45654E-05 | 0.001148154 | 34 | 5.01187E-23 | 1.58489E-20 | chr8 | 144671304 | 144672251 | NM_032378.6 | 5E-23 | - | 144671304 | 144672251 | 947, |
| peak12239 | 4.7 | 0.000831764 | 0.00724436 | 8.42 | 0.000346737 | 0.001348963 | chr9 | 4860196 | 4860295 | NM_001286700.1 | 0.00035 | + | 4860196 | 4860295 | 99, |
| peak12269 | 2.3 | 0.003548134 | 0.020417379 | 20.4 | 3.16228E-06 | 1.94984E-05 | chr9 | 35547556 | 35547905 | NM_014806.5 | 0.0000032 | + | 35547556 | 35547905 | 349, |
| peak12273 | 2.96 | 0.002041738 | 0.013803843 | 20 | 5.88844E-06 | 3.38844E-05 | chr9 | 35547562 | 35547813 | NR_052015.4 | 0.0000059 | + | 35547562 | 35547813 | 251, |
| peak12277 | 2.41 | 0.001819701 | 0.012882496 | 17.5 | 3.31131E-06 | 2.04174E-05 | chr9 | 35547545 | 35547845 | NM_001135999.1 | 0.0000033 | + | 35547545 | 35547845 | 300, |
| peak12293 | 2.95 | 0.003467369 | 0.019952623 | 12.5 | 0.003715352 | 0.012589254 | chr9 | 37086918 | 37087018 | NR_036592.1 | 0.0037 | + | 37086918 | 37087018 | 100, |
| peak12316 | 1.68 | 0.004365158 | 0.023442288 | 6 | 2.13796E-07 | 1.86209E-06 | chr9 | 71866016 | 71866216 | NM_001369870.1 | 0.00000021 | + | 71866016 | 71866216 | 200, |
| peak12320 | 2.92 | 0.002089296 | 0.013803843 | 8.37 | 1.90546E-07 | 1.69824E-06 | chr9 | 71840947 | 71842786 | NM_001369871.1 | 0.00000019 | + | 71840947 | 71842786 | 144,106, |
| peak12323 | 1.44 | 0.003630781 | 0.020417379 | 6.71 | 1.04713E-05 | 5.62341E-05 | chr9 | 71865967 | 71866217 | NM_001369871.1 | 0.00001 | + | 71865967 | 71866217 | 250, |
| peak12371 | 1.61 | 0.001698244 | 0.012022644 | 6.71 | 1.65959E-06 | 1.12202E-05 | chr9 | 71865970 | 71866219 | NM_001369875.1 | 0.0000017 | + | 71865970 | 71866219 | 249, |
| peak12414 | 2.11 | 0.002398833 | 0.015488166 | 3.38 | 3.16228E-06 | 1.94984E-05 | chr9 | 91616536 | 91616686 | NM_005226.4 | 0.0000032 | + | 91616536 | 91616686 | 150, |
| peak12416 | 2.77 | 0.003630781 | 0.020892961 | 2.67 | 3.16228E-06 | 0.000104713 | chr9 | 91617780 | 91617881 | NM_005226.4 | 0.0000032 | + | 91617780 | 91617881 | 101, |
| peak12447 | 2.48 | 0.000295121 | 0.003388442 | 6 | 1.62181E-05 | 0.000416869 | chr9 | 96859933 | 96860233 | NM_001253830.2 | 0.000016 | + | 96859933 | 96860233 | 300, |
| peak12448 | 2.49 | 0.00030903 | 0.003467369 | 9.5 | 1.20226E-05 | 0.000323594 | chr9 | 96859937 | 96860238 | NM_177995.3 | 0.000012 | + | 96859937 | 96860238 | 301, |
| peak12449 | 2.52 | 1.47911E-05 | 0.000389045 | 8.75 | 2.23872E-05 | 0.000562341 | chr9 | 96859948 | 96860248 | NM_152422.4 | 0.000022 | + | 96859948 | 96860248 | 300, |
| peak12460 | 3.72 | 0.000114815 | 0.001698244 | 2.82 | 3.63078E-05 | 0.000169824 | chr9 | 101911703 | 101911804 | NM_001130916.3 | 0.000036 | + | 101911703 | 101911804 | 101, |
| peak12464 | 3.6 | 5.7544E-05 | 0.001047129 | 2.04 | 1.99526E-05 | 0.0001 | chr9 | 101911730 | 101911830 | NM_004612.4 | 0.00002 | + | 101911730 | 101911830 | 100, |
| peak12501 | 3.2 | 2.88403E-05 | 0.000660693 | 3.29 | 0.000218776 | 0.004073803 | chr9 | 106864363 | 106864793 | NM_001042551.2 | 0.00022 | + | 106864363 | 106864793 | 111,89, |
| peak12505 | 3.42 | 2.0893E-05 | 0.000512861 | 2.88 | 0.000223872 | 0.004168694 | chr9 | 106864370 | 106864800 | NM_006444.3 | 0.00022 | + | 106864370 | 106864800 | 104,96, |
| peak12509 | 3.7 | 1.20226E-06 | 5.62341E-05 | 2.7 | 0.000446684 | 0.007585776 | chr9 | 106864426 | 106873862 | NM_001042550.2 | 0.00045 | + | 106864426 | 106873862 | 48,150,1, |
| peak12515 | 2.98 | 0.003548134 | 0.020417379 | 1.78 | 3.63078E-05 | 0.000851138 | chr9 | 109690221 | 109690321 | NM_021224.6 | 0.000036 | + | 109690221 | 109690321 | 100, |
| peak12576 | 6.18 | 0.004265795 | 0.022908677 | 5.88 | 0.000616595 | 0.002290868 | chr9 | 123898222 | 123900884 | NM_007018.5 | 0.00062 | + | 123898222 | 123900884 | 38,62, |
| peak12597 | 3.56 | 2.95121E-09 | 2.5704E-07 | 16 | 1.20226E-07 | 1.14815E-06 | chr9 | 128064437 | 128064884 | NM_001354293.2 | 0.00000012 | + | 128064437 | 128064884 | 447, |
| peak12599 | 3.57 | 1.23027E-08 | 0.000001 | 18 | 2.88403E-06 | 1.8197E-05 | chr9 | 128064442 | 128064892 | NM_001282679.2 | 0.0000029 | + | 128064442 | 128064892 | 450, |
| peak12601 | 3.57 | 1.23027E-08 | 0.000001 | 18 | 4.0738E-06 | 2.45471E-05 | chr9 | 128064442 | 128064892 | NM_001282680.3 | 0.0000041 | + | 128064442 | 128064892 | 450, |
| peak12602 | 3.47 | 3.63078E-09 | 3.16228E-07 | 16.2 | 9.33254E-09 | 1.28825E-07 | chr9 | 128064360 | 128064911 | NM_001282681.3 | 9.3E-09 | + | 128064360 | 128064911 | 551, |
| peak12604 | 3.55 | 4.16869E-10 | 3.98107E-08 | 14 | 3.54813E-08 | 4.0738E-07 | chr9 | 128064359 | 128064908 | NM_001330777.3 | 0.000000035 | + | 128064359 | 128064908 | 549, |
| peak12605 | 3.52 | 6.91831E-10 | 6.45654E-08 | 14 | 5.37032E-08 | 5.7544E-07 | chr9 | 128064359 | 128064909 | NM_001330778.3 | 0.000000054 | + | 128064359 | 128064909 | 550, |
| peak12607 | 3.32 | 2.69153E-07 | 1.62181E-05 | 16 | 1.51356E-07 | 1.38038E-06 | chr9 | 128064358 | 128064906 | NM_001354294.2 | 0.00000015 | + | 128064358 | 128064906 | 548, |
| peak12609 | 3.58 | 1.28825E-09 | 1.14815E-07 | 16 | 3.80189E-08 | 4.2658E-07 | chr9 | 128064359 | 128064907 | NM_001354295.2 | 0.000000038 | + | 128064359 | 128064907 | 548, |
| peak12610 | 3.32 | 2.69153E-07 | 1.62181E-05 | 16 | 1.14815E-07 | 1.09648E-06 | chr9 | 128064358 | 128064907 | NM_001354296.2 | 0.00000011 | + | 128064358 | 128064907 | 549, |
| peak12612 | 3.71 | 4.46684E-09 | 3.89045E-07 | 17.6 | 5.7544E-07 | 4.36516E-06 | chr9 | 128064436 | 128064886 | NM_001354297.2 | 0.00000058 | + | 128064436 | 128064886 | 450, |
| peak12614 | 3.32 | 2.69153E-07 | 1.62181E-05 | 16 | 1.28825E-07 | 1.20226E-06 | chr9 | 128064358 | 128064906 | NM_001354298.2 | 0.00000013 | + | 128064358 | 128064906 | 548, |
| peak12616 | 3.62 | 1.86209E-08 | 1.47911E-06 | 18.6 | 4.16869E-06 | 2.45471E-05 | chr9 | 128064449 | 128064899 | NM_001354299.2 | 0.0000042 | + | 128064449 | 128064899 | 450, |
| peak12618 | 3.47 | 5.37032E-09 | 4.57088E-07 | 16.5 | 8.51138E-09 | 1.1749E-07 | chr9 | 128064361 | 128064911 | NM_001354300.2 | 8.5E-09 | + | 128064361 | 128064911 | 550, |
| peak12620 | 3.46 | 6.60693E-09 | 5.49541E-07 | 17.6 | 1.12202E-06 | 7.94328E-06 | chr9 | 128064437 | 128064887 | NM_001354301.2 | 0.0000011 | + | 128064437 | 128064887 | 450, |
| peak12622 | 3.62 | 1.28825E-09 | 1.14815E-07 | 15.6 | 2.39883E-08 | 2.81838E-07 | chr9 | 128064360 | 128064910 | NM_015635.4 | 0.000000024 | + | 128064360 | 128064910 | 550, |
| peak12624 | 3.62 | 1.28825E-09 | 1.14815E-07 | 15.6 | 5.01187E-08 | 5.37032E-07 | chr9 | 128064360 | 128064910 | NR_148732.2 | 0.00000005 | + | 128064360 | 128064910 | 550, |
| peak12627 | 3.54 | 4.57088E-10 | 4.2658E-08 | 14 | 4.46684E-08 | 4.89779E-07 | chr9 | 128064359 | 128064908 | NR_148733.2 | 0.000000045 | + | 128064359 | 128064908 | 549, |
| peak12628 | 3.1 | 0.000851138 | 0.007413102 | 4.44 | 0.003162278 | 0.010715193 | chr9 | 129642615 | 129642866 | NM_001099270.2 | 0.0032 | + | 129642615 | 129642866 | 251, |
| peak12630 | 2.3 | 0.000588844 | 0.005754399 | 6.5 | 1.20226E-09 | 1.38038E-07 | chr9 | 130206903 | 130207651 | NM_001286696.1 | 1.2E-09 | + | 130206903 | 130207651 | 748, |
| peak12632 | 2.25 | 0.000912011 | 0.007762471 | 6.5 | 1.99526E-14 | 1E-11 | chr9 | 130207305 | 130207651 | NM_001286698.1 | 2E-14 | + | 130207305 | 130207651 | 346, |
| peak12636 | 2.51 | 0.003090295 | 0.018197009 | 35.9 | 0.00017378 | 0.000707946 | chr9 | 130869946 | 130870047 | NM_001265614.2 | 0.00017 | + | 130869946 | 130870047 | 101, |
| peak12686 | 1.31 | 0.004786301 | 0.025118864 | 8.33 | 5.37032E-05 | 0.000245471 | chr9 | 132251184 | 132255807 | NR_038955.1 | 0.000054 | + | 132251184 | 132255807 | 394,51, |
| peak12689 | 4.62 | 2.88403E-06 | 0.000109648 | 12.4 | 4.2658E-07 | 3.38844E-06 | chr9 | 133579135 | 133579684 | NR_104230.1 | 0.00000043 | + | 133579135 | 133579684 | 549, |
| peak12700 | 1.09 | 0.003981072 | 0.021877616 | 9.81 | 0.000812831 | 0.003019952 | chr9 | 135553440 | 135554089 | NR_133925.1 | 0.00081 | + | 135553440 | 135554089 | 649, |
| peak12715 | 3.52 | 0.002089296 | 0.014125375 | 1.71 | 0.000269153 | 0.001071519 | chr9 | 137330335 | 137330486 | NM_002957.6 | 0.00027 | + | 137330335 | 137330486 | 151, |
| peak12719 | 3.21 | 0.000186209 | 0.002454709 | 2.16 | 0.000288403 | 0.001148154 | chr9 | 137330347 | 137330497 | NM_001291920.1 | 0.00029 | + | 137330347 | 137330497 | 150, |
| peak12723 | 3.09 | 0.000512861 | 0.005011872 | 1.67 | 0.000147911 | 0.000616595 | chr9 | 137330393 | 137330493 | NM_001291921.2 | 0.00015 | + | 137330393 | 137330493 | 100, |
| peak12740 | 3.75 | 2.5704E-07 | 1.54882E-05 | 13.4 | 4.16869E-05 | 0.000194984 | chr9 | 5921212 | 5921462 | NM_001017969.3 | 0.000042 | - | 5921212 | 5921462 | 250, |
| peak12748 | 3.49 | 5.37032E-06 | 0.00018197 | 5.6 | 2.69153E-05 | 0.000645654 | chr9 | 6014573 | 6014824 | NM_012416.4 | 0.000027 | - | 6014573 | 6014824 | 251, |
| peak12865 | 3.86 | 0.00030903 | 0.003467369 | 10.2 | 0.000295121 | 0.001174898 | chr9 | 26905776 | 26905926 | NM_001321546.1 | 0.0003 | - | 26905776 | 26905926 | 150, |
| peak12890 | 2.84 | 8.31764E-08 | 5.7544E-06 | 9.89 | 2.39883E-05 | 0.000120226 | chr9 | 34370611 | 34371011 | NM_020702.5 | 0.000024 | - | 34370611 | 34371011 | 400, |
| peak12891 | 4.89 | 4.16869E-07 | 2.34423E-05 | 2.56 | 2.39883E-05 | 0.000120226 | chr9 | 34371360 | 34371561 | NM_020702.5 | 0.000024 | - | 34371360 | 34371561 | 201, |
| peak12893 | 1.1 | 0.00042658 | 0.004466836 | 3.2 | 2.39883E-05 | 0.000120226 | chr9 | 34372060 | 34372710 | NM_020702.5 | 0.000024 | - | 34372060 | 34372710 | 650, |
| peak12903 | 2.01 | 0.000123027 | 0.001778279 | 5.52 | 2.34423E-07 | 2.04174E-06 | chr9 | 35660780 | 35661029 | NM_032818.3 | 0.00000023 | - | 35660780 | 35661029 | 249, |
| peak12917 | 3.8 | 0.000812831 | 0.00724436 | 5.5 | 3.71535E-05 | 0.00017378 | chr9 | 37440592 | 37440793 | NM_014872.3 | 0.000037 | - | 37440592 | 37440793 | 201, |

| | | | | | | | | | | | | | | | |
|---|---|---|---|---|---|---|---|---|---|---|---|---|---|---|---|
| peak13069 | 2.05 | 0.001122018 | 0.008912509 | 8.37 | 0.000234423 | 0.000954993 | chr9 | 100886901 | 100887102 | NM_003389.3 | 0.00023 | - | 100886901 | 100887102 | 201, |
| peak13070 | 2.41 | 0.003548134 | 0.020417379 | 6.88 | 0.002089296 | 0.007413102 | chr9 | 100886926 | 100887175 | NM_052820.4 | 0.0021 | - | 100886926 | 100887175 | 249, |
| peak13146 | 2.06 | 0.000707946 | 0.006456542 | 5.25 | 1.69824E-05 | 8.70964E-05 | chr9 | 115927917 | 115928117 | NM_015258.2 | 0.000017 | - | 115927917 | 115928117 | 200, |
| peak13149 | 2.39 | 0.001148154 | 0.009120108 | 15.3 | 1.69824E-05 | 8.70964E-05 | chr9 | 115938881 | 115946082 | NM_015258.2 | 0.000017 | - | 115938881 | 115946082 | 72,172,103,52, |
| peak13171 | 2.05 | 3.16228E-06 | 0.00011749 | 5.5 | 5.01187E-12 | 1.8197E-10 | chr9 | 125582303 | 125582898 | NM_005388.5 | 5E-12 | - | 125582303 | 125582898 | 595, |
| peak13172 | 1.58 | 0.003235937 | 0.019054607 | 1.75 | 3.98107E-07 | 1.77828E-05 | chr9 | 125680959 | 125681108 | NM_001304363.2 | 0.0000004 | - | 125680959 | 125681108 | 149, |
| peak13174 | 1.54 | 0.003311311 | 0.019498446 | 8.4 | 3.98107E-07 | 3.16228E-06 | chr9 | 125681750 | 125681899 | NM_001304363.2 | 0.0000004 | - | 125681750 | 125681899 | 149, |
| peak13175 | 1.42 | 0.003235937 | 0.019054607 | 1.75 | 8.51138E-07 | 3.38844E-05 | chr9 | 125680962 | 125681111 | NM_020924.4 | 0.00000085 | - | 125680962 | 125681111 | 149, |
| peak13177 | 2.91 | 0.001148154 | 0.009120108 | 8.4 | 8.51138E-07 | 6.16595E-06 | chr9 | 125681754 | 125681903 | NM_020924.4 | 0.00000085 | - | 125681754 | 125681903 | 149, |
| peak13197 | 2.95 | 4.89779E-05 | 0.000954993 | 10.5 | 0.000134896 | 0.00057544 | chr9 | 130494048 | 130494393 | NM_001134431.3 | 0.00013 | - | 130494048 | 130494393 | 345, |
| peak13226 | 1.59 | 2.34423E-07 | 1.44544E-05 | 14.4 | 6.0256E-06 | 3.46737E-05 | chr9 | 135202309 | 135203359 | NM_001351527.1 | 0.000006 | - | 135202309 | 135203359 | 1050, |
| peak13232 | 2.02 | 0.001023293 | 0.00851138 | 10.3 | 0.000512861 | 0.001949845 | chr9 | 135202622 | 135203372 | NM_001351528.2 | 0.00051 | - | 135202622 | 135203372 | 750, |
| peak13236 | 2.09 | 1.94984E-05 | 0.000489779 | 11.5 | 0.001380384 | 0.005011872 | chr9 | 135202637 | 135203385 | NM_015046.7 | 0.0014 | - | 135202637 | 135203385 | 748, |
| peak13270 | 3.61 | 0.000512861 | 0.005011872 | 5.25 | 1.99526E-13 | 7.94328E-11 | chr9 | 140082011 | 140082110 | NM_013366.4 | 2E-13 | - | 140082011 | 140082110 | 99, |
| peak13274 | 2.98 | 2.45471E-05 | 0.00057544 | 16.2 | 2.13796E-10 | 5.12861E-09 | chr9 | 140286698 | 140289756 | NR_104599.2 | 2.1E-10 | - | 140286698 | 140289756 | 989,2, |
| peak13334 | 2.07 | 0.00042658 | 0.004466836 | 3.38 | 0.00616595 | 0.020417379 | chr10 | 5772614 | 5772715 | NM_001321783.1 | 0.0062 | + | 5772614 | 5772715 | 101, |
| peak13340 | 2.71 | 3.23594E-06 | 0.00011749 | 30.9 | 0.000123027 | 0.000524807 | chr10 | 5788185 | 5789086 | NM_001321783.1 | 0.00012 | + | 5788185 | 5789086 | 901, |
| peak13344 | 2.24 | 0.000239883 | 0.002884032 | 3.38 | 0.012302688 | 0.039810717 | chr10 | 5772617 | 5772718 | NM_001321784.1 | 0.012 | + | 5772617 | 5772718 | 101, |
| peak13349 | 2.58 | 3.46737E-06 | 0.000125893 | 31.1 | 0.000204174 | 0.000831764 | chr10 | 5788188 | 5789088 | NM_001321784.1 | 0.0002 | + | 5788188 | 5789088 | 900, |
| peak13352 | 1.97 | 0.000812831 | 0.00724436 | 3.18 | 0.005011872 | 0.016982437 | chr10 | 5772607 | 5772707 | NM_001321785.1 | 0.005 | + | 5772607 | 5772707 | 100, |
| peak13355 | 2.17 | 0.002398833 | 0.015488166 | 3.17 | 0.000147911 | 0.000616595 | chr10 | 5784162 | 5784411 | NM_001321785.1 | 0.00015 | + | 5784162 | 5784411 | 249, |
| peak13356 | 1.88 | 0.001862087 | 0.012882496 | 27.8 | 0.000147911 | 0.000616595 | chr10 | 5788212 | 5788909 | NM_001321785.1 | 0.00015 | + | 5788212 | 5788909 | 697, |
| peak13357 | 5.54 | 1.99526E-09 | 1.77828E-07 | 3.21 | 0.000147911 | 0.000616595 | chr10 | 5789007 | 5789207 | NM_001321785.1 | 0.00015 | + | 5789007 | 5789207 | 200, |
| peak13365 | 2.62 | 1.07152E-07 | 7.24436E-06 | 26.5 | 9.12011E-05 | 0.000398107 | chr10 | 5788199 | 5789197 | NM_017782.4 | 0.000091 | + | 5788199 | 5789197 | 998, |
| peak13437 | 1.31 | 0.000831764 | 0.00724436 | 15.6 | 4.16869E-05 | 0.000194984 | chr10 | 13167477 | 13169859 | NM_021980.4 | 0.000042 | + | 13167477 | 13169859 | 90,94,115, |
| peak13462 | 4.09 | 0.001995262 | 0.013489629 | 7.8 | 0.00074131 | 0.012022644 | chr10 | 25313649 | 25313899 | NM_024838.5 | 0.00074 | + | 25313649 | 25313899 | 250, |
| peak13711 | 4.51 | 0.003890451 | 0.021379621 | 7 | 1.04713E-07 | 0.000001 | chr10 | 31809217 | 31809417 | NM_001323676.2 | 0.0000001 | + | 31809217 | 31809417 | 200, |
| peak13735 | 2.64 | 0.003235937 | 0.019054607 | 3.35 | 2.18776E-08 | 2.63027E-07 | chr10 | 35772379 | 35818939 | NM_001282854.2 | 0.000000022 | + | 35772379 | 35818939 | 27,35,39, |
| peak13744 | 2.55 | 8.70964E-05 | 0.001412538 | 4.5 | 0.000165959 | 0.000691831 | chr10 | 38344695 | 38344946 | NM_001278173.1 | 0.00017 | + | 38344695 | 38344946 | 251, |
| peak13747 | 2.56 | 1.7378E-05 | 0.000446684 | 4.5 | 0.000512861 | 0.001949845 | chr10 | 38344698 | 38344948 | NM_001278170.2 | 0.00051 | + | 38344698 | 38344948 | 250, |
| peak13750 | 3.24 | 0.000354813 | 0.003890451 | 3.43 | 1.86209E-05 | 9.54993E-05 | chr10 | 38344693 | 38344943 | NM_001324176.2 | 0.000019 | + | 38344693 | 38344943 | 250, |
| peak13753 | 2.54 | 3.80189E-05 | 0.000794328 | 4.5 | 0.000109648 | 0.00047863 | chr10 | 38344696 | 38344946 | NM_006974.3 | 0.00011 | + | 38344696 | 38344946 | 250, |
| peak13757 | 2.88 | 7.76247E-06 | 0.000239883 | 3.25 | 0.002089296 | 0.00724436 | chr10 | 38344672 | 38344973 | NM_001278171.2 | 0.0021 | + | 38344672 | 38344973 | 301, |
| peak13760 | 2.85 | 1.09648E-05 | 0.00030903 | 3.25 | 0.000295121 | 0.001148154 | chr10 | 38344671 | 38344972 | NM_001278174.2 | 0.0003 | + | 38344671 | 38344972 | 301, |
| peak13762 | 2.58 | 0.000154882 | 0.002137962 | 4.22 | 3.63078E-05 | 0.000169824 | chr10 | 38344679 | 38344929 | NM_001278175.2 | 0.000036 | + | 38344679 | 38344929 | 250, |
| peak13764 | 2.68 | 1.31826E-05 | 0.000354813 | 2.87 | 0.000371535 | 0.001412538 | chr10 | 38344668 | 38344968 | NM_001278176.2 | 0.00037 | + | 38344668 | 38344968 | 300, |
| peak13767 | 2.51 | 8.51138E-05 | 0.001380384 | 3.43 | 4.57088E-05 | 0.00020893 | chr10 | 38344692 | 38344941 | NM_001278177.2 | 0.000046 | + | 38344692 | 38344941 | 249, |
| peak13769 | 2.68 | 1.31826E-05 | 0.000354813 | 2.87 | 0.000371535 | 0.001412538 | chr10 | 38344668 | 38344968 | NM_001324177.2 | 0.00037 | + | 38344668 | 38344968 | 300, |
| peak13772 | 2.51 | 8.51138E-05 | 0.001380384 | 3.43 | 4.7863E-05 | 0.000218776 | chr10 | 38344691 | 38344941 | NM_001324178.2 | 0.000048 | + | 38344691 | 38344941 | 250, |
| peak13775 | 2.48 | 0.000169824 | 0.002238721 | 3.86 | 0.0001 | 0.00042658 | chr10 | 38344689 | 38344938 | NM_006954.2 | 0.0001 | + | 38344689 | 38344938 | 249, |
| peak13822 | 3.7 | 0.000114815 | 0.001698244 | 8.17 | 3.16228E-05 | 0.000151356 | chr10 | 43650569 | 43650819 | NR_135077.1 | 0.000032 | + | 43650569 | 43650819 | 250, |
| peak13828 | 2.38 | 0.000489779 | 0.004897788 | 9.53 | 0.002951209 | 0.01023293 | chr10 | 45498867 | 45499014 | NM_006963.5 | 0.003 | + | 45498867 | 45499014 | 147, |
| peak13887 | 2.33 | 0.001513561 | 0.011220185 | 8.54 | 7.24436E-05 | 0.000323594 | chr10 | 70922228 | 70931150 | NM_001035260.2 | 0.000072 | + | 70922228 | 70931150 | 40,69,239, |
| peak13997 | 1.51 | 0.000131826 | 0.001862087 | 7.14 | 2.23872E-05 | 0.000109648 | chr10 | 86131596 | 86133394 | NM_001351290.2 | 0.000022 | + | 86131596 | 86133394 | 629,20, |
| peak14002 | 1.43 | 0.000190546 | 0.002454709 | 7.14 | 0.00001 | 5.37032E-05 | chr10 | 86131597 | 86133396 | NM_018999.4 | 0.00001 | + | 86131597 | 86133396 | 628,22, |
| peak14074 | 1.23 | 0.003801894 | 0.021379621 | 2.4 | 0.001479108 | 0.022908677 | chr10 | 101464699 | 101464900 | NM_020354.5 | 0.0015 | + | 101464699 | 101464900 | 201, |
| peak14095 | 3.29 | 0.000323594 | 0.003630781 | 9.57 | 8.12831E-07 | 5.88844E-06 | chr10 | 104391364 | 104391765 | NM_016169.3 | 0.00000081 | + | 104391364 | 104391765 | 401, |
| peak14150 | 1.35 | 0.002951209 | 0.017782794 | 58.4 | 1.25893E-16 | 1.58489E-14 | chr10 | 112724113 | 112724807 | NM_001269039.2 | 1.3E-16 | + | 112724113 | 112724807 | 694, |
| peak14175 | 1.86 | 9.33254E-05 | 0.001479108 | 4.6 | 8.12831E-08 | 8.12831E-07 | chr10 | 121657972 | 121658323 | NM_007190.4 | 0.000000081 | + | 121657972 | 121658323 | 351, |
| peak14176 | 2.74 | 0.000331131 | 0.003715352 | 9.67 | 0.006025596 | 0.019952623 | chr10 | 121700220 | 121700321 | NM_007190.4 | 0.006 | + | 121700220 | 121700321 | 101, |
| peak14177 | 2.77 | 0.000263027 | 0.003090295 | 5 | 5.24807E-06 | 3.0903E-05 | chr10 | 121700190 | 121700390 | NR_037771.2 | 0.0000052 | + | 121700190 | 121700390 | 200, |
| peak14307 | 2.03 | 0.001096478 | 0.008912509 | 5.8 | 0.003388442 | 0.011748976 | chr10 | 24909510 | 24909960 | NR_160024.1 | 0.0034 | - | 24909510 | 24909960 | 450, |
| peak14312 | 1.97 | 0.003715352 | 0.020892961 | 5.8 | 0.004897788 | 0.016595869 | chr10 | 24909513 | 24910011 | NR_160025.1 | 0.0049 | - | 24909513 | 24910011 | 498, |
| peak14322 | 1.99 | 0.000102329 | 0.001548817 | 4.7 | 0.015135612 | 0.048977882 | chr10 | 24909471 | 24909820 | NM_001367454.1 | 0.015 | - | 24909471 | 24909820 | 349, |
| peak14327 | 1.94 | 0.002238721 | 0.014791084 | 4.48 | 0.006456542 | 0.021379621 | chr10 | 24909517 | 24909968 | NR_160023.1 | 0.0065 | - | 24909517 | 24909968 | 451, |
| peak14340 | 2.08 | 0.002041738 | 0.013803843 | 5.39 | 0.002454709 | 0.00851138 | chr10 | 24909441 | 24909791 | NM_001367450.1 | 0.0025 | - | 24909441 | 24909791 | 350, |
| peak14345 | 2.16 | 0.002344229 | 0.015135612 | 5.3 | 0.002754229 | 0.009549926 | chr10 | 24909490 | 24909940 | NM_001367451.1 | 0.0028 | - | 24909490 | 24909940 | 450, |

| | | | | | | | | | | | | | | | |
|---|---|---|---|---|---|---|---|---|---|---|---|---|---|---|---|
| peak14351 | 2.2 | 0.004168694 | 0.022387211 | 5.36 | 0.004265795 | 0.014454398 | chr10 | 24909489 | 24909789 | NM_001367452.1 | 0.0043 | - | 24909489 | 24909789 | 300, |
| peak14356 | 2.16 | 4.36516E-05 | 0.000870964 | 4.73 | 0.014125375 | 0.045708819 | chr10 | 24909519 | 24909769 | NR_160021.1 | 0.014 | - | 24909519 | 24909769 | 250, |
| peak14366 | 2.03 | 0.000933254 | 0.007943282 | 5.8 | 0.007762471 | 0.025703958 | chr10 | 24909512 | 24909959 | NM_001367447.1 | 0.0078 | - | 24909512 | 24909959 | 447, |
| peak14370 | 2.26 | 0.000588844 | 0.005623413 | 5.11 | 0.00128825 | 0.004677351 | chr10 | 24909533 | 24909784 | NM_020824.4 | 0.0013 | - | 24909533 | 24909784 | 251, |
| peak14411 | 3.88 | 0.000131826 | 0.001862087 | 5.18 | 2.18776E-05 | 0.000109648 | chr10 | 29840158 | 29843993 | NM_021738.3 | 0.000022 | - | 29840158 | 29843993 | 34,152,14, |
| peak14420 | 3.89 | 0.000165959 | 0.002238721 | 5.4 | 1.8197E-09 | 3.23594E-08 | chr10 | 29840172 | 29844008 | NM_001323600.1 | 1.8E-09 | - | 29840172 | 29844008 | 20,152,29, |
| peak14423 | 2.46 | 2.95121E-06 | 0.000112202 | 8.44 | 2.5704E-05 | 0.000125893 | chr10 | 30315942 | 30316192 | NM_001350001.2 | 0.000026 | - | 30315942 | 30316192 | 250, |
| peak14428 | 2.31 | 4.67735E-06 | 0.000162181 | 9.33 | 9.54993E-06 | 5.12861E-05 | chr10 | 30315949 | 30316199 | NM_020848.4 | 0.0000095 | - | 30315949 | 30316199 | 250, |
| peak14436 | 2.4 | 2.5704E-05 | 0.000588844 | 7.8 | 1.31826E-05 | 6.91831E-05 | chr10 | 30315957 | 30316208 | NM_001350021.2 | 0.000013 | - | 30315957 | 30316208 | 251, |
| peak14442 | 2.51 | 1.94984E-06 | 8.12831E-05 | 8.78 | 1.94984E-05 | 9.77237E-05 | chr10 | 30315946 | 30316196 | NM_001350022.2 | 0.000019 | - | 30315946 | 30316196 | 250, |
| peak14539 | 1.14 | 0.003715352 | 0.020892961 | 18.1 | 8.12831E-05 | 0.000363078 | chr10 | 53457819 | 53459355 | NM_015235.3 | 0.000081 | - | 53457819 | 53459355 | 1536, |
| peak14544 | 3 | 6.60693E-05 | 0.001148154 | 6 | 2.75423E-07 | 1.28825E-05 | chr10 | 61819491 | 61819740 | NM_001149.3 | 0.00000028 | - | 61819491 | 61819740 | 249, |
| peak14546 | 3.12 | 0.000102329 | 0.001584893 | 5.8 | 1.99526E-05 | 0.000501187 | chr10 | 61819513 | 61819763 | NM_001320874.2 | 0.00002 | - | 61819513 | 61819763 | 250, |
| peak14551 | 2.93 | 0.001071519 | 0.008709636 | 3.67 | 0.000123027 | 0.002454709 | chr10 | 61819511 | 61819661 | NM_001204404.2 | 0.00012 | - | 61819511 | 61819661 | 150, |
| peak14553 | 2.67 | 9.12011E-05 | 0.001479108 | 3.8 | 0.000316228 | 0.005623413 | chr10 | 61819514 | 61822869 | NM_001204403.2 | 0.00032 | - | 61819514 | 61822869 | 250,1, |
| peak14631 | 8.24 | 4.46684E-06 | 0.000158489 | 3 | 4.89779E-09 | 4.36516E-07 | chr10 | 70243223 | 70243324 | NM_001324314.2 | 4.9E-09 | - | 70243223 | 70243324 | 101, |
| peak14633 | 7.65 | 2.75423E-05 | 0.000630957 | 3.75 | 2.95121E-07 | 1.38038E-05 | chr10 | 70243251 | 70246907 | NM_001324317.2 | 0.0000003 | - | 70243251 | 70246907 | 94,7, |
| peak14635 | 7.68 | 3.71535E-05 | 0.000776247 | 4.5 | 4.46684E-09 | 4.0738E-07 | chr10 | 70243237 | 70243338 | NM_152707.4 | 4.5E-09 | - | 70243237 | 70243338 | 101, |
| peak14636 | 7.74 | 4.89779E-05 | 0.000954993 | 6.5 | 0.000162181 | 0.003162278 | chr10 | 70243221 | 70243321 | NM_001324315.1 | 0.00016 | - | 70243221 | 70243321 | 100, |
| peak14638 | 8.44 | 2.29087E-06 | 9.12011E-05 | 3.63 | 1.51356E-08 | 1.14815E-06 | chr10 | 70243231 | 70243330 | NM_001324312.2 | 0.000000015 | - | 70243231 | 70243330 | 99, |
| peak14640 | 1.51 | 0.000537032 | 0.005248075 | 8.88 | 5.62341E-06 | 3.23594E-05 | chr10 | 71899468 | 71899815 | NR_073595.1 | 0.0000056 | - | 71899468 | 71899815 | 347, |
| peak14642 | 2.31 | 0.000537032 | 0.005248075 | 4.5 | 3.89045E-05 | 0.00018197 | chr10 | 71899466 | 71899665 | NR_173555.4 | 0.000039 | - | 71899466 | 71899665 | 199, |
| peak14644 | 1.84 | 0.003090295 | 0.018620871 | 5.6 | 6.30957E-20 | 1.58489E-17 | chr10 | 71899502 | 71899847 | NR_073580.2 | 6.3E-20 | - | 71899502 | 71899847 | 345, |
| peak14653 | 1.62 | 0.000213796 | 0.002691535 | 7.25 | 4.89779E-06 | 2.88403E-05 | chr10 | 71899485 | 71899835 | NR_073594.2 | 0.0000049 | - | 71899485 | 71899835 | 350, |
| peak14675 | 5.01 | 0.002187762 | 0.014454398 | 4.06 | 0.014454398 | 0.046773514 | chr10 | 74311057 | 74322709 | NM_001195518.2 | 0.014 | - | 74311057 | 74322709 | 42,57, |
| peak14682 | 1.55 | 2.51189E-05 | 0.000588844 | 9.12 | 2.69153E-05 | 0.000131826 | chr10 | 75567184 | 75568025 | NM_003635.4 | 0.000027 | - | 75567184 | 75568025 | 841, |
| peak14688 | 2.8 | 0.004786301 | 0.025118864 | 35 | 7.94328E-07 | 5.88844E-06 | chr10 | 75573599 | 75573699 | NM_001367527.1 | 0.00000079 | - | 75573599 | 75573699 | 100, |
| peak14689 | 2.82 | 0.003801894 | 0.021379621 | 34.9 | 1.25893E-06 | 8.91251E-06 | chr10 | 75573601 | 75573701 | NM_001367530.1 | 0.0000013 | - | 75573601 | 75573701 | 100, |
| peak14691 | 2.86 | 0.002884032 | 0.017782794 | 34.4 | 1.23027E-06 | 8.51138E-06 | chr10 | 75573605 | 75573706 | NM_001367532.1 | 0.0000012 | - | 75573605 | 75573706 | 101, |
| peak14692 | 2.76 | 0.004786301 | 0.025118864 | 34.4 | 1.58489E-06 | 1.07152E-05 | chr10 | 75573604 | 75573705 | NM_001367533.1 | 0.0000016 | - | 75573604 | 75573705 | 101, |
| peak14693 | 2.8 | 0.004786301 | 0.025118864 | 35 | 7.58578E-07 | 5.62341E-06 | chr10 | 75573600 | 75573700 | NM_001367534.1 | 0.00000076 | - | 75573600 | 75573700 | 100, |
| peak14694 | 2.79 | 0.004570882 | 0.024547089 | 33.6 | 1.02329E-06 | 7.4131E-06 | chr10 | 75573596 | 75573697 | NM_001367536.1 | 0.000001 | - | 75573596 | 75573697 | 101, |
| peak14695 | 2.76 | 0.004786301 | 0.025118864 | 34.4 | 1.62181E-06 | 1.09648E-05 | chr10 | 75573605 | 75573705 | NM_001367537.1 | 0.0000016 | - | 75573605 | 75573705 | 100, |
| peak14696 | 2.8 | 0.004677351 | 0.024547089 | 35 | 1.1749E-06 | 8.31764E-06 | chr10 | 75573599 | 75573700 | NM_001367539.1 | 0.0000012 | - | 75573599 | 75573700 | 101, |
| peak14697 | 2.85 | 0.003467369 | 0.019952623 | 34.4 | 1.14815E-06 | 8.12831E-06 | chr10 | 75573601 | 75573701 | NM_001367542.1 | 0.0000011 | - | 75573601 | 75573701 | 100, |
| peak14698 | 2.84 | 0.002818383 | 0.017378008 | 37.9 | 1.07152E-06 | 7.58578E-06 | chr10 | 75573595 | 75573695 | NM_001367543.1 | 0.0000011 | - | 75573595 | 75573695 | 100, |
| peak14700 | 2.76 | 0.004786301 | 0.025118864 | 34.4 | 1.12202E-06 | 7.94328E-06 | chr10 | 75573603 | 75573703 | NM_001367548.1 | 0.0000011 | - | 75573603 | 75573703 | 100, |
| peak14704 | 2.8 | 0.004786301 | 0.025118864 | 36 | 3.80189E-07 | 3.0903E-06 | chr10 | 75573598 | 75573699 | NR_160040.1 | 0.00000038 | - | 75573598 | 75573699 | 101, |
| peak14705 | 2.76 | 0.004786301 | 0.025118864 | 34.4 | 1.51356E-06 | 1.02329E-05 | chr10 | 75573602 | 75573703 | NR_160042.1 | 0.0000015 | - | 75573602 | 75573703 | 101, |
| peak14706 | 2.8 | 0.004677351 | 0.024547089 | 35 | 7.4131E-07 | 5.49541E-06 | chr10 | 75573599 | 75573700 | NR_160044.1 | 0.00000074 | - | 75573599 | 75573700 | 101, |
| peak14708 | 2.79 | 0.003801894 | 0.021379621 | 37.9 | 1.09648E-06 | 7.76247E-06 | chr10 | 75573595 | 75573695 | NR_160046.1 | 0.0000011 | - | 75573595 | 75573695 | 100, |
| peak14709 | 2.86 | 0.003801894 | 0.021379621 | 33.4 | 1.09648E-06 | 7.76247E-06 | chr10 | 75573597 | 75573697 | NR_160047.1 | 0.0000011 | - | 75573597 | 75573697 | 100, |
| peak14712 | 2.79 | 0.004570882 | 0.024547089 | 33.6 | 1.62181E-06 | 1.09648E-05 | chr10 | 75573596 | 75573697 | NM_172171.2 | 0.0000016 | - | 75573596 | 75573697 | 101, |
| peak14727 | 3.03 | 6.16595E-05 | 0.001096478 | 16 | 4.57088E-05 | 0.000213796 | chr10 | 88259894 | 88260294 | NM_001318328.1 | 0.000046 | - | 88259894 | 88260294 | 400, |
| peak14731 | 2.57 | 0.000158489 | 0.002137962 | 14.8 | 0.000245471 | 0.001 | chr10 | 88259858 | 88260306 | NM_015045.5 | 0.00025 | - | 88259858 | 88260306 | 448, |
| peak14745 | 1.6 | 0.001148154 | 0.009120108 | 5.62 | 0.002344229 | 0.008317638 | chr10 | 97423651 | 97424051 | NM_001143973.1 | 0.0023 | - | 97423651 | 97424051 | 400, |
| peak14747 | 1.63 | 0.000831764 | 0.00724436 | 5.62 | 0.000707946 | 0.002630268 | chr10 | 97423651 | 97424046 | NM_015631.6 | 0.00071 | - | 97423651 | 97424046 | 395, |
| peak14753 | 2.52 | 0.000138038 | 0.001949845 | 2.67 | 0.000229087 | 0.004265795 | chr10 | 101911539 | 101911788 | NM_001347856.2 | 0.00023 | - | 101911539 | 101911788 | 249, |
| peak14754 | 2.11 | 6.30957E-06 | 0.000204174 | 6.83 | 0.000229087 | 0.000912011 | chr10 | 101911887 | 101914693 | NM_001347856.2 | 0.00023 | - | 101911887 | 101914693 | 222,77, |
| peak14755 | 2.2 | 5.49541E-07 | 2.95121E-05 | 8.75 | 0.000275423 | 0.001096478 | chr10 | 101911869 | 101914672 | NM_001347858.2 | 0.00028 | - | 101911869 | 101914672 | 240,56, |
| peak14756 | 2.52 | 0.000138038 | 0.001949845 | 2.67 | 0.000109648 | 0.002238721 | chr10 | 101911537 | 101911786 | NM_001347859.2 | 0.00011 | - | 101911537 | 101911786 | 249, |
| peak14757 | 2.06 | 8.70964E-08 | 6.0256E-06 | 6.86 | 0.000109648 | 0.00047863 | chr10 | 101911885 | 101914690 | NM_001347859.2 | 0.00011 | - | 101911885 | 101914690 | 224,74, |
| peak14758 | 2.53 | 7.76247E-10 | 7.24436E-08 | 7.31 | 4.16869E-05 | 0.000194984 | chr10 | 101911543 | 101914649 | NM_006459.4 | 0.000042 | - | 101911543 | 101914649 | 566,33, |
| peak14759 | 2.24 | 3.89045E-07 | 2.18776E-05 | 5.83 | 0.000301995 | 0.001174898 | chr10 | 101911869 | 101914673 | NR_144755.2 | 0.0003 | - | 101911869 | 101914673 | 240,57, |
| peak14760 | 2.24 | 3.89045E-07 | 2.18776E-05 | 5.83 | 0.000269153 | 0.001071519 | chr10 | 101911869 | 101914673 | NR_144756.2 | 0.00027 | - | 101911869 | 101914673 | 240,57, |
| peak14761 | 2.52 | 0.000138038 | 0.001949845 | 2.67 | 0.000147911 | 0.002884032 | chr10 | 101911537 | 101911786 | NR_144757.2 | 0.00015 | - | 101911537 | 101911786 | 249, |
| peak14762 | 2.1 | 7.58578E-08 | 5.24807E-06 | 8 | 0.000147911 | 0.000616595 | chr10 | 101911884 | 101914689 | NR_144757.2 | 0.00015 | - | 101911884 | 101914689 | 225,73, |

| | | | | | | | | | | | | | | | |
|---|---|---|---|---|---|---|---|---|---|---|---|---|---|---|---|
| peak14763 | 2.26 | 4.2658E-07 | 2.39883E-05 | 7.31 | 8.12831E-05 | 0.000354813 | chr10 | 101911542 | 101914696 | NR_144758.2 | 0.000081 | - | 101911542 | 101914696 | 567,80, |
| peak14766 | 2.3 | 0.000128825 | 0.001862087 | 7.07 | 4.2658E-05 | 0.000199526 | chr10 | 101911845 | 101915907 | NR_144759.2 | 0.000043 | - | 101911845 | 101915907 | 264,80,6, |
| peak14819 | 3.07 | 0.001023293 | 0.008317638 | 4.06 | 1.41254E-05 | 7.4131E-05 | chr10 | 106074150 | 106074301 | NM_001272012.1 | 0.000014 | - | 106074150 | 106074301 | 151, |
| peak14824 | 3.63 | 0.000194984 | 0.002511886 | 3.18 | 1.90546E-05 | 9.54993E-05 | chr10 | 106074146 | 106074296 | NM_001272013.2 | 0.000019 | - | 106074146 | 106074296 | 150, |
| peak14908 | 4.18 | 0.000169824 | 0.002238721 | 2.91 | 0.000331131 | 0.00128825 | chr10 | 118661396 | 118666166 | NM_001258299.1 | 0.00033 | - | 118661396 | 118666166 | 72,29, |
| peak14911 | 2.23 | 7.07946E-05 | 0.001230269 | 19.3 | 4.16869E-09 | 6.45654E-08 | chr10 | 119042666 | 119043166 | NM_173791.5 | 4.2E-09 | - | 119042666 | 119043166 | 500, |
| peak14923 | 2.83 | 0.003019952 | 0.018197009 | 16.1 | 4.2658E-06 | 2.51189E-05 | chr10 | 121334573 | 121334773 | NM_001033925.1 | 0.0000043 | - | 121334573 | 121334773 | 200, |
| peak14958 | 1.57 | 0.001148154 | 0.009120108 | 38.6 | 3.98107E-13 | 2.51189E-11 | chr10 | 124753459 | 124754008 | NM_001372123.1 | 4E-13 | - | 124753459 | 124754008 | 549, |
| peak14961 | 1.57 | 0.000446684 | 0.004570882 | 25.1 | 1.02329E-08 | 1.41254E-07 | chr10 | 124753458 | 124754006 | NM_001372126.1 | 0.00000001 | - | 124753458 | 124754006 | 548, |
| peak14970 | 4.21 | 4.57088E-06 | 0.000158489 | 12 | 0.002041738 | 0.00724436 | chr10 | 125805281 | 125805430 | NM_015892.5 | 0.002 | - | 125805281 | 125805430 | 149, |
| peak14975 | 4.2 | 7.24436E-06 | 0.000229087 | 12 | 0.001995262 | 0.00691831 | chr10 | 125805279 | 125805428 | NM_001270764.2 | 0.002 | - | 125805279 | 125805428 | 149, |
| peak15045 | 3.55 | 8.12831E-06 | 0.000251189 | 10.6 | 0.002238721 | 0.007943282 | chr11 | 10526196 | 10527393 | NM_000480.3 | 0.0022 | + | 10526196 | 10527393 | 10,139, |
| peak15048 | 3.31 | 7.24436E-07 | 3.71535E-05 | 9.5 | 0.002570396 | 0.008709636 | chr11 | 10526178 | 10527427 | NM_001172430.1 | 0.0026 | + | 10526178 | 10527427 | 28,173, |
| peak15049 | 1.92 | 3.16228E-06 | 0.00011749 | 19.6 | 2.34423E-08 | 2.81838E-07 | chr11 | 10795657 | 10800600 | NM_001346279.2 | 0.000000023 | + | 10795657 | 10800600 | 59,97,113,375, |
| peak15050 | 2.1 | 3.71535E-05 | 0.000776247 | 20.4 | 2.95121E-08 | 3.46737E-07 | chr11 | 10795650 | 10800646 | NM_014633.5 | 0.00000003 | + | 10795650 | 10800646 | 66,97,113,421, |
| peak15132 | 4.18 | 0.001122018 | 0.008912509 | 3.75 | 3.80189E-05 | 0.00018197 | chr11 | 32955873 | 32956024 | NM_001076786.3 | 0.000038 | + | 32955873 | 32956024 | 151, |
| peak15188 | 2.84 | 0.000295121 | 0.003388442 | 9 | 5.37032E-05 | 0.000245471 | chr11 | 47594712 | 47594963 | NM_001143984.1 | 0.000054 | + | 47594712 | 47594963 | 251, |
| peak15190 | 2.89 | 0.000295121 | 0.003388442 | 9 | 0.000691831 | 0.002570396 | chr11 | 47594717 | 47594964 | NM_175732.3 | 0.00069 | + | 47594717 | 47594964 | 247, |
| peak15321 | 1.06 | 6.60693E-06 | 0.000213796 | 95.8 | 1.54882E-05 | 7.94328E-05 | chr11 | 58916380 | 58919824 | NM_001142520.2 | 0.000015 | + | 58916380 | 58919824 | 46,602, |
| peak15343 | 1.21 | 3.0903E-05 | 0.000691831 | 78.6 | 4.36516E-10 | 9.54993E-09 | chr11 | 58916392 | 58919784 | NM_198847.3 | 4.4E-10 | + | 58916392 | 58919784 | 34,562, |
| peak15408 | 7.57 | 0.001096478 | 0.008912509 | 9.44 | 1.12202E-05 | 6.0256E-05 | chr11 | 65562742 | 65562893 | NM_004561.4 | 0.000011 | + | 65562742 | 65562893 | 151, |
| peak15417 | 4.67 | 0.000398107 | 0.004265795 | 3.84 | 2.0893E-07 | 1.8197E-06 | chr11 | 66392001 | 66392102 | NM_006328.4 | 0.00000021 | + | 66392001 | 66392102 | 101, |
| peak15465 | 1.36 | 0.001513561 | 0.011220185 | 2.02 | 0.011748976 | 0.03801894 | chr11 | 70118400 | 70118501 | NM_003626.4 | 0.012 | + | 70118400 | 70118501 | 101, |
| peak15487 | 2.94 | 2.23872E-05 | 0.000537032 | 17.8 | 8.91251E-10 | 1.7378E-08 | chr11 | 74347291 | 74351879 | NM_006591.3 | 8.9E-10 | + | 74347291 | 74351879 | 29,271, |
| peak15489 | 1.45 | 1.99526E-10 | 1.94984E-08 | 28.7 | 1.07152E-09 | 2.04174E-08 | chr11 | 74546665 | 74547615 | NM_001098638.2 | 1.1E-09 | + | 74546665 | 74547615 | 950, |
| peak15521 | 2.41 | 6.45654E-05 | 0.001148154 | 4 | 5.7544E-06 | 3.31131E-05 | chr11 | 82892199 | 82895901 | NM_001346413.2 | 0.0000058 | + | 82892199 | 82895901 | 34,156,93,36,181, |
| peak15527 | 2.36 | 2.95121E-05 | 0.000660693 | 4 | 5.88844E-07 | 4.57088E-06 | chr11 | 82892201 | 82895902 | NM_001346414.2 | 0.00000059 | + | 82892201 | 82895902 | 32,156,93,36,182, |
| peak15534 | 2.31 | 0.000218776 | 0.002754229 | 4 | 2.29087E-06 | 1.47911E-05 | chr11 | 82892198 | 82895900 | NM_001346415.2 | 0.0000023 | + | 82892198 | 82895900 | 35,156,93,36,180, |
| peak15539 | 2.32 | 5.62341E-05 | 0.001023293 | 4.4 | 1.58489E-06 | 1.07152E-05 | chr11 | 82892197 | 82895899 | NM_015885.4 | 0.0000016 | + | 82892197 | 82895899 | 36,156,93,36,179, |
| peak15557 | 2.29 | 5.88844E-05 | 0.001071519 | 10.8 | 2.29087E-05 | 0.000112202 | chr11 | 95546661 | 95555045 | NM_001243776.1 | 0.000023 | + | 95546661 | 95555045 | 92,117,78,11, |
| peak15559 | 2.3 | 5.62341E-05 | 0.001047129 | 10.8 | 6.45654E-06 | 3.63078E-05 | chr11 | 95546661 | 95555048 | NM_001243777.1 | 0.0000065 | + | 95546661 | 95555048 | 92,117,78,14, |
| peak15561 | 2.37 | 0.000190546 | 0.002454709 | 9.86 | 2.04174E-05 | 0.000102329 | chr11 | 95546645 | 95552065 | NM_001363604.1 | 0.00002 | + | 95546645 | 95552065 | 108,117,75, |
| peak15563 | 2.39 | 0.00011749 | 0.001737801 | 9.86 | 2.51189E-05 | 0.000123027 | chr11 | 95546646 | 95552066 | NM_014679.5 | 0.000025 | + | 95546646 | 95552066 | 107,117,76, |
| peak15606 | 1.43 | 0.000537032 | 0.005248075 | 34.5 | 0.000154882 | 0.000645654 | chr11 | 111953540 | 111953839 | NM_001082969.2 | 0.00015 | + | 111953540 | 111953839 | 299, |
| peak15607 | 1.37 | 0.00060256 | 0.005754399 | 30.5 | 0.000125893 | 0.000537032 | chr11 | 111953542 | 111953841 | NM_001301017.2 | 0.00013 | + | 111953542 | 111953841 | 299, |
| peak15608 | 2.09 | 0.000223872 | 0.002754229 | 28.3 | 0.000331131 | 0.001318257 | chr11 | 111953546 | 111953795 | NM_001301019.2 | 0.00033 | + | 111953546 | 111953795 | 249, |
| peak15609 | 2.08 | 0.000218776 | 0.002754229 | 28.3 | 0.000398107 | 0.001513561 | chr11 | 111953549 | 111953797 | NM_001301021.2 | 0.0004 | + | 111953549 | 111953797 | 248, |
| peak15639 | 4.78 | 0.001584893 | 0.011481536 | 1.89 | 0.001096478 | 0.017378008 | chr11 | 120957591 | 120957692 | NM_152715.4 | 0.0011 | + | 120957591 | 120957692 | 101, |
| peak15646 | 3.71 | 4.89779E-05 | 0.000933254 | 8 | 2.95121E-05 | 0.000144544 | chr11 | 126162467 | 126162668 | NM_001039661.1 | 0.00003 | + | 126162467 | 126162668 | 201, |
| peak15681 | 2.92 | 0.001258925 | 0.009772372 | 6 | 0.000154882 | 0.000645654 | chr11 | 3380401 | 3380750 | NM_001130520.3 | 0.00015 | - | 3380401 | 3380750 | 349, |
| peak15682 | 3.59 | 5.24807E-05 | 0.001 | 6.11 | 0.002238721 | 0.007943282 | chr11 | 3380405 | 3380706 | NM_001242841.2 | 0.0022 | - | 3380405 | 3380706 | 301, |
| peak15684 | 3.29 | 1.28825E-05 | 0.000354813 | 6 | 0.002041738 | 0.007079458 | chr11 | 3380400 | 3380700 | NR_040083.2 | 0.002 | - | 3380400 | 3380700 | 300, |
| peak15686 | 3.22 | 5.88844E-05 | 0.001071519 | 5.5 | 0.001122018 | 0.004073803 | chr11 | 3380404 | 3380705 | NM_001256825.1 | 0.0011 | - | 3380404 | 3380705 | 301, |
| peak15689 | 3.98 | 1.99526E-05 | 0.000489779 | 7.83 | 0.001659587 | 0.005888437 | chr11 | 4406374 | 4406821 | NM_003141.4 | 0.0017 | - | 4406374 | 4406821 | 447, |
| peak15695 | 2.82 | 7.4131E-05 | 0.001258925 | 2.78 | 1.25893E-15 | 1.25893E-13 | chr11 | 6498207 | 6498457 | NM_001370411.1 | 1.3E-15 | - | 6498207 | 6498457 | 250, |
| peak15700 | 2.8 | 0.000107152 | 0.00162181 | 2.89 | 0.00025704 | 0.001047129 | chr11 | 6498209 | 6498460 | NM_001242854.1 | 0.00026 | - | 6498209 | 6498460 | 251, |
| peak15705 | 2.87 | 0.00042658 | 0.004466836 | 1.25 | 0.000234423 | 0.000933254 | chr11 | 6631792 | 6631893 | NM_006284.4 | 0.00023 | - | 6631792 | 6631893 | 101, |
| peak15708 | 1.81 | 3.23594E-07 | 1.90546E-05 | 15.7 | 0.00017378 | 0.000707946 | chr11 | 6643806 | 6644106 | NM_003737.4 | 0.00017 | - | 6643806 | 6644106 | 300, |
| peak15806 | 4.39 | 0.000416869 | 0.004365158 | 2.76 | 2.63027E-05 | 0.000128825 | chr11 | 46529880 | 46534336 | NM_017749.3 | 0.000026 | - | 46529880 | 46534336 | 40,60, |
| peak15829 | 4.42 | 0.001862087 | 0.012882496 | 2.94 | 0.000107152 | 0.000457088 | chr11 | 46529884 | 46534340 | NM_001367471.1 | 0.00011 | - | 46529884 | 46534340 | 36,64, |
| peak15836 | 1.53 | 0.002089296 | 0.013803843 | 1.65 | 0.001949845 | 0.00691831 | chr11 | 46837905 | 46839960 | NM_001008938.4 | 0.0019 | - | 46837905 | 46839960 | 50,100, |
| peak15840 | 3.3 | 9.77237E-05 | 0.001513561 | 3 | 1.20226E-06 | 4.57088E-05 | chr11 | 47595097 | 47595298 | NM_001318716.1 | 0.0000012 | - | 47595097 | 47595298 | 201, |
| peak15841 | 6.02 | 0.001318257 | 0.01 | 2.1 | 1.07152E-05 | 5.7544E-05 | chr11 | 47594681 | 47594780 | NM_001318725.1 | 0.000011 | - | 47594681 | 47594780 | 99, |
| peak15843 | 6.02 | 0.001318257 | 0.01 | 2.1 | 1.25893E-05 | 6.60693E-05 | chr11 | 47594681 | 47594780 | NM_018095.6 | 0.000013 | - | 47594681 | 47594780 | 99, |
| peak15845 | 5.99 | 0.002137962 | 0.014454398 | 2.68 | 3.98107E-06 | 2.39883E-05 | chr11 | 47594687 | 47594786 | NM_001318717.1 | 0.000004 | - | 47594687 | 47594786 | 99, |
| peak15847 | 6.05 | 0.001174898 | 0.009120108 | 2.1 | 1.1749E-05 | 6.16595E-05 | chr11 | 47594679 | 47594778 | NM_001318718.1 | 0.000012 | - | 47594679 | 47594778 | 99, |
| peak15849 | 5.99 | 0.000954993 | 0.008128305 | 2.68 | 4.16869E-06 | 2.51189E-05 | chr11 | 47594689 | 47594790 | NM_001318719.1 | 0.0000042 | - | 47594689 | 47594790 | 101, |

| | | | | | | | | | | | | | | | |
|---|---|---|---|---|---|---|---|---|---|---|---|---|---|---|---|
| peak15851 | 5.79 | 0.00144544 | 0.010715193 | 2.68 | 4.89779E-06 | 2.88403E-05 | chr11 | 47594687 | 47594787 | NM_001318720.1 | 0.0000049 | - | 47594687 | 47594787 | 100, |
| peak15854 | 3.3 | 9.77237E-05 | 0.001513561 | 3 | 1.99526E-06 | 7.07946E-05 | chr11 | 47595096 | 47595297 | NM_001318721.1 | 0.000002 | - | 47595096 | 47595297 | 201, |
| peak15855 | 5.99 | 0.002137962 | 0.014454398 | 2.68 | 6.45654E-06 | 3.63078E-05 | chr11 | 47594686 | 47594785 | NM_001318722.1 | 0.0000065 | - | 47594686 | 47594785 | 99, |
| peak15857 | 6.05 | 0.001174898 | 0.009120108 | 2.1 | 1.02329E-05 | 5.49541E-05 | chr11 | 47594680 | 47594779 | NM_001318723.1 | 0.00001 | - | 47594680 | 47594779 | 99, |
| peak15859 | 6.05 | 0.001174898 | 0.009120108 | 2.1 | 1.02329E-05 | 5.49541E-05 | chr11 | 47594680 | 47594779 | NM_001318724.1 | 0.00001 | - | 47594680 | 47594779 | 99, |
| peak15861 | 5.99 | 0.002454709 | 0.015848932 | 2.22 | 1.23027E-05 | 6.60693E-05 | chr11 | 47594682 | 47594781 | NM_016506.6 | 0.000012 | - | 47594682 | 47594781 | 99, |
| peak15893 | 1.19 | 0.000676083 | 0.006309573 | 2.45 | 2.81838E-05 | 0.000138038 | chr11 | 62295149 | 62295549 | NM_001346446.2 | 0.000028 | - | 62295149 | 62295549 | 400, |
| peak15897 | 1.36 | 0.000281838 | 0.003235937 | 1.33 | 8.31764E-06 | 4.57088E-05 | chr11 | 62298193 | 62298294 | NM_001346446.2 | 0.0000083 | - | 62298193 | 62298294 | 101, |
| peak15899 | 1.24 | 0.000933254 | 0.007943282 | 2.72 | 8.31764E-06 | 4.57088E-05 | chr11 | 62299341 | 62299841 | NM_001346446.2 | 0.0000083 | - | 62299341 | 62299841 | 500, |
| peak15912 | 1.15 | 7.58578E-05 | 0.00128825 | 2.32 | 0.000125893 | 0.000537032 | chr11 | 62295170 | 62295571 | NM_001346445.2 | 0.00013 | - | 62295170 | 62295571 | 401, |
| peak15917 | 1.24 | 0.000263027 | 0.003090295 | 3.05 | 3.46737E-06 | 2.13796E-05 | chr11 | 62299320 | 62299820 | NM_001346445.2 | 0.0000035 | - | 62299320 | 62299820 | 500, |
| peak15930 | 1.21 | 0.000107152 | 0.00162181 | 2.39 | 2.5704E-05 | 0.000125893 | chr11 | 62295148 | 62295548 | NM_001620.3 | 0.000026 | - | 62295148 | 62295548 | 400, |
| peak15934 | 1.21 | 0.000363078 | 0.003981072 | 1.37 | 6.16595E-06 | 3.46737E-05 | chr11 | 62298191 | 62298292 | NM_001620.3 | 0.0000062 | - | 62298191 | 62298292 | 101, |
| peak15936 | 1.25 | 0.000977237 | 0.008128305 | 2.75 | 6.16595E-06 | 3.46737E-05 | chr11 | 62299339 | 62299839 | NM_001620.3 | 0.0000062 | - | 62299339 | 62299839 | 500, |
| peak15943 | 2.01 | 0.003311311 | 0.019498446 | 5 | 0.000275423 | 0.001096478 | chr11 | 62342572 | 62342819 | NM_022830.3 | 0.00028 | - | 62342572 | 62342819 | 247, |
| peak15979 | 1.96 | 8.91251E-06 | 0.000269153 | 15.9 | 0.000120226 | 0.000512861 | chr11 | 67119114 | 67119311 | NR_046411.1 | 0.00012 | - | 67119114 | 67119311 | 197, |
| peak15980 | 1.84 | 5.7544E-05 | 0.001071519 | 15.9 | 0.004168694 | 0.014125375 | chr11 | 67119114 | 67119262 | NR_046413.1 | 0.0042 | - | 67119114 | 67119262 | 148, |
| peak15993 | 1.82 | 0.000269153 | 0.003162278 | 21.7 | 1.04713E-08 | 1.41254E-07 | chr11 | 68029741 | 68030186 | NM_022338.4 | 0.00000001 | - | 68029741 | 68030186 | 445, |
| peak16012 | 2.67 | 4.46684E-05 | 0.000870964 | 7.69 | 8.51138E-08 | 8.51138E-07 | chr11 | 72003611 | 72003761 | NM_001258392.3 | 0.000000085 | - | 72003611 | 72003761 | 150, |
| peak16016 | 2.54 | 0.001174898 | 0.009332543 | 8.22 | 7.07946E-08 | 7.24436E-07 | chr11 | 72003620 | 72003771 | NM_001258393.3 | 0.000000071 | - | 72003620 | 72003771 | 151, |
| peak16020 | 2.49 | 6.60693E-05 | 0.001148154 | 9.09 | 9.77237E-08 | 9.54993E-07 | chr11 | 72003604 | 72003804 | NM_030813.6 | 0.000000098 | - | 72003604 | 72003804 | 200, |
| peak16023 | 1.42 | 0.000912011 | 0.007762471 | 18.1 | 9.77237E-08 | 9.54993E-07 | chr11 | 72006575 | 72019638 | NM_030813.6 | 0.000000098 | - | 72006575 | 72019638 | 117,162,45,56,68, |
| peak16026 | 1.95 | 0.000512861 | 0.005011872 | 14.6 | 0.005623413 | 0.018620871 | chr11 | 72012890 | 72019601 | NM_001258394.2 | 0.0056 | - | 72012890 | 72019601 | 118,45,56,31, |
| peak16029 | 6.11 | 0.002754229 | 0.017378008 | 6.94 | 0.00616595 | 0.020417379 | chr11 | 72549276 | 72549376 | NM_014824.3 | 0.0062 | - | 72549276 | 72549376 | 100, |
| peak16062 | 2.57 | 4.46684E-06 | 0.000158489 | 4.4 | 2.69153E-07 | 2.29087E-06 | chr11 | 77396183 | 77404650 | NM_016578.3 | 0.00000027 | - | 77396183 | 77404650 | 21,80,99, |
| peak16063 | 2.05 | 0.001174898 | 0.009332543 | 44.2 | 2.69153E-07 | 2.29087E-06 | chr11 | 77409724 | 77412748 | NM_016578.3 | 0.00000027 | - | 77409724 | 77412748 | 14,983, |
| peak16215 | 7.39 | 1.47911E-08 | 1.1749E-06 | 14.2 | 1.7378E-10 | 4.36516E-09 | chr11 | 128844259 | 128844559 | NM_001142685.1 | 1.7E-10 | - | 128844259 | 128844559 | 300, |
| peak16230 | 1.41 | 4.0738E-05 | 0.000831764 | 24.1 | 3.16228E-11 | 9.54993E-10 | chr11 | 130130919 | 130131168 | NM_001301099.1 | 3.2E-11 | - | 130130919 | 130131168 | 249, |
| peak16233 | 1.79 | 0.000831764 | 0.00724436 | 17.4 | 9.77237E-09 | 1.34896E-07 | chr11 | 130130862 | 130131163 | NM_014155.4 | 9.8E-09 | - | 130130862 | 130131163 | 301, |
| peak16236 | 1.68 | 5.12861E-05 | 0.000977237 | 17.6 | 2.5704E-09 | 4.36516E-08 | chr11 | 130130916 | 130131167 | NM_001301098.2 | 2.6E-09 | - | 130130916 | 130131167 | 251, |
| peak16239 | 1.64 | 4.57088E-06 | 0.000158489 | 23.8 | 1.14815E-07 | 1.09648E-06 | chr11 | 130130872 | 130131172 | NM_001370219.1 | 0.00000011 | - | 130130872 | 130131172 | 300, |
| peak16242 | 1.64 | 4.67735E-06 | 0.000162181 | 23.9 | 3.63078E-07 | 2.95121E-06 | chr11 | 130130872 | 130131172 | NM_001370220.1 | 0.00000036 | - | 130130872 | 130131172 | 300, |
| peak16245 | 1.47 | 0.001737801 | 0.012302688 | 19.2 | 1.51356E-08 | 1.94984E-07 | chr11 | 130130867 | 130131168 | NM_001370221.1 | 0.000000015 | - | 130130867 | 130131168 | 301, |
| peak16248 | 1.65 | 5.7544E-06 | 0.000190546 | 24.8 | 1.07152E-07 | 1.02329E-06 | chr11 | 130130874 | 130131173 | NM_001370223.1 | 0.00000011 | - | 130130874 | 130131173 | 299, |
| peak16250 | 1.8 | 7.24436E-07 | 3.71535E-05 | 19.5 | 7.4131E-07 | 5.49541E-06 | chr11 | 130130834 | 130131181 | NR_163264.1 | 0.00000074 | - | 130130834 | 130131181 | 347, |
| peak16252 | 1.81 | 0.001148154 | 0.009120108 | 17.6 | 3.89045E-08 | 4.36516E-07 | chr11 | 130130866 | 130131167 | NR_163265.1 | 0.000000039 | - | 130130866 | 130131167 | 301, |
| peak16255 | 3.21 | 3.98107E-08 | 2.95121E-06 | 6.81 | 6.60693E-06 | 3.80189E-05 | chr11 | 130784892 | 130785538 | NM_001347918.1 | 0.0000066 | - | 130784892 | 130785538 | 646, |
| peak16256 | 1.43 | 0.003715352 | 0.020892961 | 11.8 | 0.003981072 | 0.013489629 | chr11 | 130785885 | 130786035 | NM_001347918.1 | 0.004 | - | 130785885 | 130786035 | 150, |
| peak16258 | 1.65 | 0.001949845 | 0.013182567 | 11.6 | 4.7863E-05 | 0.000218776 | chr11 | 130785882 | 130785983 | NM_001347919.1 | 0.000048 | - | 130785882 | 130785983 | 101, |
| peak16260 | 1.53 | 0.003801894 | 0.021379621 | 11.6 | 4.2658E-05 | 0.000199526 | chr11 | 130785883 | 130785984 | NM_014758.2 | 0.000043 | - | 130785883 | 130785984 | 101, |
| peak16262 | 1.54 | 0.003548134 | 0.020417379 | 11.8 | 3.31131E-05 | 0.000158489 | chr11 | 130785884 | 130785984 | NR_144939.1 | 0.000033 | - | 130785884 | 130785984 | 100, |
| peak16266 | 1.54 | 0.003548134 | 0.020417379 | 11.8 | 0.00047863 | 0.001819701 | chr11 | 130785884 | 130785984 | NM_001347921.1 | 0.00048 | - | 130785884 | 130785984 | 100, |
| peak16292 | 1.35 | 5.7544E-05 | 0.001071519 | 18.3 | 8.91251E-05 | 0.000389045 | chr12 | 977879 | 978079 | NM_213655.4 | 0.000089 | + | 977879 | 978079 | 200, |
| peak16295 | 1.36 | 0.001819701 | 0.012589254 | 18.1 | 0.001023293 | 0.003715352 | chr12 | 1137710 | 1192651 | NM_178039.4 | 0.001 | + | 1137710 | 1192651 | 28,322, |
| peak16299 | 1.38 | 0.002570396 | 0.016595869 | 20.4 | 0.000870964 | 0.003235937 | chr12 | 1137709 | 1192649 | NM_178040.4 | 0.00087 | + | 1137709 | 1192649 | 29,320, |
| peak16303 | 1.12 | 0.003890451 | 0.021877616 | 16.8 | 1.90546E-05 | 9.54993E-05 | chr12 | 1137646 | 1192737 | NR_027946.3 | 0.000019 | + | 1137646 | 1192737 | 92,408, |
| peak16307 | 2.1 | 3.46737E-05 | 0.00074131 | 12 | 0.000346737 | 0.001348963 | chr12 | 1599706 | 1600006 | NR_027946.3 | 0.00035 | + | 1599706 | 1600006 | 300, |
| peak16311 | 2.94 | 0.001258925 | 0.009772372 | 7.22 | 0.000363078 | 0.001412538 | chr12 | 1602800 | 1603101 | NR_027946.3 | 0.00036 | + | 1602800 | 1603101 | 301, |
| peak16312 | 2.75 | 0.000660693 | 0.00616595 | 4.33 | 0.000363078 | 0.001412538 | chr12 | 1603299 | 1603650 | NR_027946.3 | 0.00036 | + | 1603299 | 1603650 | 351, |
| peak16317 | 3.02 | 0.00162181 | 0.011748976 | 16.6 | 0.006456542 | 0.021379621 | chr12 | 1137212 | 1137363 | NR_027949.3 | 0.0065 | + | 1137212 | 1137363 | 151, |
| peak16320 | 1.28 | 0.004786301 | 0.025118864 | 18.9 | 0.000831764 | 0.003019952 | chr12 | 1137710 | 1192651 | NM_001301248.1 | 0.00083 | + | 1137710 | 1192651 | 28,322, |
| peak16340 | 2.02 | 0.002818383 | 0.017378008 | 16.7 | 1.47911E-06 | 1.02329E-05 | chr12 | 9847931 | 9848726 | NM_001004419.5 | 0.0000015 | + | 9847931 | 9848726 | 795, |
| peak16341 | 2 | 0.001584893 | 0.011481536 | 16.8 | 2.75423E-06 | 1.7378E-05 | chr12 | 9847927 | 9848722 | NM_001197317.3 | 0.0000028 | + | 9847927 | 9848722 | 795, |
| peak16343 | 1.81 | 0.002511886 | 0.015848932 | 16.8 | 9.33254E-06 | 5.12861E-05 | chr12 | 9848368 | 9848718 | NM_001197318.3 | 0.0000093 | + | 9848368 | 9848718 | 350, |
| peak16344 | 2.05 | 0.00162181 | 0.011748976 | 16.7 | 2.34423E-06 | 1.51356E-05 | chr12 | 9847930 | 9848725 | NM_001197319.3 | 0.0000023 | + | 9847930 | 9848725 | 795, |
| peak16345 | 2.11 | 0.001548817 | 0.011481536 | 15.7 | 1.09648E-05 | 5.88844E-05 | chr12 | 9847917 | 9848715 | NM_013269.6 | 0.000011 | + | 9847917 | 9848715 | 798, |
| peak16346 | 1.95 | 0.002290868 | 0.014791084 | 26.2 | 7.24436E-07 | 5.37032E-06 | chr12 | 9847950 | 9848701 | NR_036693.3 | 0.00000072 | + | 9847950 | 9848701 | 751, |

| | | | | | | | | | | | | | | | |
|---|---|---|---|---|---|---|---|---|---|---|---|---|---|---|---|
| peak16376 | 2.44 | 2.0893E-07 | 1.28825E-05 | 67.6 | 1.58489E-14 | 1.25893E-12 | chr12 | 14576872 | 14577273 | NM_181352.2 | 1.6E-14 | + | 14576872 | 14577273 | 401, |
| peak16385 | 4.09 | 0.003630781 | 0.020417379 | 4.36 | 7.76247E-08 | 7.76247E-07 | chr12 | 19436442 | 19436592 | NM_001143821.3 | 0.000000078 | + | 19436442 | 19436592 | 150, |
| peak16387 | 4.17 | 0.001047129 | 0.00851138 | 3.4 | 8.51138E-06 | 4.67735E-05 | chr12 | 19436432 | 19436583 | NM_001256470.2 | 0.0000085 | + | 19436432 | 19436583 | 151, |
| peak16388 | 4.06 | 4.67735E-05 | 0.000912011 | 9.71 | 0.001995262 | 0.007079458 | chr12 | 19436350 | 19436601 | NM_019012.6 | 0.002 | + | 19436350 | 19436601 | 251, |
| peak16390 | 4.37 | 0.000616595 | 0.005888437 | 5.33 | 7.24436E-07 | 5.37032E-06 | chr12 | 19436424 | 19436575 | NM_001256787.1 | 0.00000072 | + | 19436424 | 19436575 | 151, |
| peak16398 | 5.04 | 0.001548817 | 0.011481536 | 12 | 4.0738E-06 | 2.45471E-05 | chr12 | 26221761 | 26221962 | NM_001164748.2 | 0.0000041 | + | 26221761 | 26221962 | 201, |
| peak16414 | 1.39 | 0.001584893 | 0.011481536 | 3.67 | 0.000141254 | 0.00060256 | chr12 | 27950731 | 27950932 | NM_020782.2 | 0.00014 | + | 27950731 | 27950932 | 201, |
| peak16424 | 1.5 | 0.004466836 | 0.023988329 | 5.33 | 0.000758578 | 0.012302688 | chr12 | 32490539 | 32491768 | NM_001363603.1 | 0.00076 | + | 32490539 | 32491768 | 101,49, |
| peak16450 | 4.42 | 0.000194984 | 0.002511886 | 2.12 | 1.99526E-14 | 1.58489E-12 | chr12 | 32793436 | 32793537 | NM_001370297.1 | 2E-14 | + | 32793436 | 32793537 | 101, |
| peak16523 | 1.76 | 0.001148154 | 0.009120108 | 3.36 | 3.16228E-11 | 1.07152E-09 | chr12 | 53434113 | 53434312 | NM_001300821.3 | 3.2E-11 | + | 53434113 | 53434312 | 199, |
| peak16525 | 1.82 | 0.001659587 | 0.012022644 | 3.37 | 3.71535E-10 | 8.31764E-09 | chr12 | 53434104 | 53434255 | NM_001330654.2 | 3.7E-10 | + | 53434104 | 53434255 | 151, |
| peak16527 | 2.25 | 0.000676083 | 0.006309573 | 2.97 | 6.30957E-12 | 2.23872E-10 | chr12 | 53434120 | 53434269 | NM_001417.7 | 6.3E-12 | + | 53434120 | 53434269 | 149, |
| peak16536 | 2.24 | 0.001659587 | 0.012022644 | 2.52 | 2.04174E-07 | 1.8197E-06 | chr12 | 53776164 | 53776264 | NM_138473.3 | 0.0000002 | + | 53776164 | 53776264 | 100, |
| peak16539 | 1.5 | 0.002818383 | 0.017378008 | 5.62 | 2.04174E-07 | 1.8197E-06 | chr12 | 53803329 | 53804994 | NM_138473.3 | 0.0000002 | + | 53803329 | 53804994 | 16,284, |
| peak16555 | 1.57 | 0.004677351 | 0.024547089 | 6.11 | 1.44544E-06 | 0.00001 | chr12 | 56397976 | 56398175 | NM_001032386.2 | 0.0000014 | + | 56397976 | 56398175 | 199, |
| peak16558 | 1.56 | 0.000831764 | 0.00724436 | 5.69 | 1.02329E-06 | 7.4131E-06 | chr12 | 56397983 | 56398180 | NM_001032387.2 | 0.000001 | + | 56397983 | 56398180 | 197, |
| peak16620 | 3.81 | 0.002691535 | 0.016595869 | 2.43 | 0.000363078 | 0.006309573 | chr12 | 62986520 | 62986670 | NM_001278471.2 | 0.00036 | + | 62986520 | 62986670 | 150, |
| peak16628 | 2.54 | 0.003548134 | 0.019952623 | 1.86 | 2.0893E-06 | 7.4131E-05 | chr12 | 64472704 | 64472805 | NM_020762.4 | 0.0000021 | + | 64472704 | 64472805 | 101, |
| peak16633 | 3.15 | 0.000141254 | 0.001995262 | 14.1 | 1.7378E-09 | 3.0903E-08 | chr12 | 65269268 | 65269419 | NM_001330186.2 | 1.7E-09 | + | 65269268 | 65269419 | 151, |
| peak16634 | 3.77 | 0.000199526 | 0.002570396 | 14.1 | 7.07946E-08 | 7.07946E-07 | chr12 | 65269266 | 65269367 | NM_001364838.2 | 0.000000071 | + | 65269266 | 65269367 | 101, |
| peak16646 | 3.39 | 3.71535E-05 | 0.000776247 | 4.18 | 8.12831E-06 | 4.46684E-05 | chr12 | 67699600 | 67699750 | NM_001329674.2 | 0.0000081 | + | 67699600 | 67699750 | 150, |
| peak16647 | 4.72 | 0.000660693 | 0.00616595 | 8.12 | 8.12831E-06 | 4.46684E-05 | chr12 | 67699998 | 67700148 | NM_001329674.2 | 0.0000081 | + | 67699998 | 67700148 | 150, |
| peak16650 | 4.06 | 0.000128825 | 0.001862087 | 6.69 | 1.54882E-07 | 1.41254E-06 | chr12 | 67699616 | 67699717 | NM_001329675.2 | 0.00000015 | + | 67699616 | 67699717 | 101, |
| peak16651 | 4.81 | 3.98107E-05 | 0.000812831 | 7.66 | 1.54882E-07 | 1.41254E-06 | chr12 | 67699966 | 67700167 | NM_001329675.2 | 0.00000015 | + | 67699966 | 67700167 | 201, |
| peak16654 | 3.56 | 0.000114815 | 0.001698244 | 9.45 | 2.63027E-06 | 1.65959E-05 | chr12 | 67699575 | 67699725 | NM_001329676.2 | 0.0000026 | + | 67699575 | 67699725 | 150, |
| peak16655 | 4.42 | 0.000107152 | 0.00162181 | 7.59 | 4.2658E-08 | 4.67735E-07 | chr12 | 67699974 | 67700175 | NM_001329676.2 | 0.000000043 | + | 67699974 | 67700175 | 201, |
| peak16658 | 3.51 | 0.000114815 | 0.001698244 | 5.75 | 0.000758578 | 0.002818383 | chr12 | 67699582 | 67699733 | NM_018448.5 | 0.00076 | + | 67699582 | 67699733 | 151, |
| peak16659 | 4.65 | 0.000251189 | 0.003019952 | 6.78 | 5.7544E-07 | 4.46684E-06 | chr12 | 67699982 | 67700132 | NM_018448.5 | 0.00000058 | + | 67699982 | 67700132 | 150, |
| peak16661 | 1.85 | 0.001862087 | 0.012882496 | 17.6 | 8.12831E-10 | 1.62181E-08 | chr12 | 68050896 | 68051746 | NM_003583.4 | 8.1E-10 | + | 68050896 | 68051746 | 850, |
| peak16672 | 2.49 | 0.000398107 | 0.004265795 | 3 | 7.76247E-05 | 0.00162181 | chr12 | 69126449 | 69127311 | NM_001330192.2 | 0.000078 | + | 69126449 | 69127311 | 70,80, |
| peak16673 | 2.56 | 0.003467369 | 0.019952623 | 3.8 | 0.000165959 | 0.003162278 | chr12 | 69126467 | 69127279 | NM_020401.4 | 0.00017 | + | 69126467 | 69127279 | 52,48, |
| peak16699 | 1.61 | 0.000162181 | 0.002187762 | 2.86 | 0.0000001 | 5.62341E-06 | chr12 | 70188274 | 70189101 | NM_001278402.1 | 0.0000001 | + | 70188274 | 70189101 | 20,181, |
| peak16707 | 4.12 | 1.41254E-07 | 9.12011E-06 | 15.1 | 2.95121E-10 | 6.76083E-09 | chr12 | 74932513 | 74932914 | NM_001136262.2 | 3E-10 | + | 74932513 | 74932914 | 401, |
| peak16764 | 3.74 | 0.000120226 | 0.001737801 | 140 | 7.24436E-09 | 1.04713E-07 | chr12 | 95694033 | 95694383 | NM_001352111.2 | 7.2E-09 | + | 95694033 | 95694383 | 350, |
| peak16765 | 3.57 | 0.001122018 | 0.008912509 | 139 | 7.76247E-10 | 1.58489E-08 | chr12 | 95694035 | 95694384 | NM_001352112.2 | 7.8E-10 | + | 95694035 | 95694384 | 349, |
| peak16766 | 3.82 | 6.30957E-05 | 0.001122018 | 119 | 7.94328E-11 | 2.13796E-09 | chr12 | 95694045 | 95694344 | NM_001352113.2 | 7.9E-11 | + | 95694045 | 95694344 | 299, |
| peak16767 | 3.57 | 0.002290868 | 0.014791084 | 138 | 7.58578E-10 | 1.54882E-08 | chr12 | 95694035 | 95694385 | NM_001352115.2 | 7.6E-10 | + | 95694035 | 95694385 | 350, |
| peak16769 | 3.84 | 6.30957E-05 | 0.001122018 | 120 | 3.16228E-11 | 9.33254E-10 | chr12 | 95694044 | 95694343 | NM_001352117.2 | 3.2E-11 | + | 95694044 | 95694343 | 299, |
| peak16770 | 3.87 | 4.36516E-05 | 0.000870964 | 118 | 3.71535E-09 | 5.88844E-08 | chr12 | 95694041 | 95694340 | NM_001352119.2 | 3.7E-09 | + | 95694041 | 95694340 | 299, |
| peak16811 | 1.45 | 0.000363078 | 0.003981072 | 4 | 5.24807E-06 | 3.01995E-05 | chr12 | 105151217 | 105151516 | NM_001173982.2 | 0.0000052 | + | 105151217 | 105151516 | 299, |
| peak16814 | 1.06 | 0.00162181 | 0.011748976 | 4.67 | 0.000151356 | 0.000630957 | chr12 | 105151205 | 105151505 | NM_018413.6 | 0.00015 | + | 105151205 | 105151505 | 300, |
| peak16817 | 1.1 | 0.001122018 | 0.008912509 | 21.4 | 7.94328E-14 | 6.30957E-12 | chr12 | 107208725 | 107209076 | NM_001330146.2 | 7.9E-14 | + | 107208725 | 107209076 | 351, |
| peak16818 | 1.12 | 0.002344229 | 0.015135612 | 21.4 | 3.98107E-13 | 1.99526E-11 | chr12 | 107208726 | 107209076 | NM_001330147.2 | 4E-13 | + | 107208726 | 107209076 | 350, |
| peak16819 | 1.1 | 0.001122018 | 0.008912509 | 21.4 | 3.98107E-14 | 3.16228E-12 | chr12 | 107208725 | 107209076 | NM_001351361.2 | 4E-14 | + | 107208725 | 107209076 | 351, |
| peak16820 | 1.06 | 4.16869E-05 | 0.000851138 | 21.4 | 6.91831E-07 | 5.24807E-06 | chr12 | 107208773 | 107209022 | NM_001351362.2 | 0.00000069 | + | 107208773 | 107209022 | 249, |
| peak16821 | 1.06 | 4.2658E-05 | 0.000851138 | 21.4 | 6.30957E-07 | 4.7863E-06 | chr12 | 107208773 | 107209023 | NM_001351363.2 | 0.00000063 | + | 107208773 | 107209023 | 250, |
| peak16832 | 1.09 | 0.002187762 | 0.014454398 | 20.9 | 7.94328E-13 | 3.98107E-11 | chr12 | 107208726 | 107209076 | NR_147137.2 | 7.9E-13 | + | 107208726 | 107209076 | 350, |
| peak16834 | 1.1 | 0.000223872 | 0.002754229 | 21.4 | 3.80189E-10 | 8.31764E-09 | chr12 | 107208725 | 107209025 | NR_147139.2 | 3.8E-10 | + | 107208725 | 107209025 | 300, |
| peak16922 | 1.29 | 0.000891251 | 0.007585776 | 16.3 | 3.80189E-08 | 4.2658E-07 | chr12 | 122626347 | 122627347 | NR_014938.6 | 0.000000038 | + | 122626347 | 122627347 | 1000, |
| peak16943 | 1.68 | 0.003715352 | 0.020892961 | 2.25 | 0.000147911 | 0.002884032 | chr12 | 132239027 | 132241104 | NM_001261411.2 | 0.00015 | + | 132239027 | 132241104 | 17,94,87, |
| peak16950 | 2.24 | 0.004570882 | 0.024547089 | 3.05 | 3.31131E-05 | 0.000158489 | chr12 | 132445334 | 132445435 | NM_015409.5 | 0.000033 | + | 132445334 | 132445435 | 101, |
| peak16952 | 3.5 | 0.004466836 | 0.023988329 | 1.46 | 3.31131E-05 | 0.000158489 | chr12 | 132471297 | 132472295 | NM_015409.5 | 0.000033 | + | 132471297 | 132472295 | 55,46, |
| peak17010 | 3.28 | 0.001258925 | 0.009772372 | 2.64 | 1.54882E-06 | 1.07152E-05 | chr12 | 416945 | 417045 | NM_001042603.3 | 0.0000015 | - | 416945 | 417045 | 100, |
| peak17014 | 2.13 | 0.001412538 | 0.010715193 | 3.62 | 9.54993E-08 | 9.12011E-07 | chr12 | 2967635 | 2967883 | NM_202003.3 | 0.000000095 | - | 2967635 | 2967883 | 248, |
| peak17015 | 3.39 | 0.0000001 | 6.91831E-06 | 5.39 | 9.54993E-08 | 9.12011E-07 | chr12 | 2968129 | 2968426 | NM_202003.3 | 0.000000095 | - | 2968129 | 2968426 | 297, |
| peak17053 | 2.46 | 0.00042658 | 0.004466836 | 6 | 0.000831764 | 0.013489629 | chr12 | 9905869 | 9906018 | NM_001781.2 | 0.00083 | - | 9905869 | 9906018 | 149, |
| peak17059 | 2.49 | 0.000467735 | 0.004786301 | 23.5 | 2.34423E-06 | 1.54882E-05 | chr12 | 12274133 | 12274284 | NM_002336.3 | 0.0000023 | - | 12274133 | 12274284 | 151, |

| | | | | | | | | | | | | | | | |
|---|---|---|---|---|---|---|---|---|---|---|---|---|---|---|---|
| peak17084 | 2.26 | 0.001778279 | 0.012302688 | 4.57 | 2.88403E-05 | 0.000691831 | chr12 | 30881771 | 30881922 | NM_001002259.2 | 0.000029 | - | 30881771 | 30881922 | 151, |
| peak17085 | 2.36 | 7.58578E-05 | 0.00128825 | 4 | 3.63078E-05 | 0.000851138 | chr12 | 30881735 | 30881934 | NM_001206856.2 | 0.000036 | - | 30881735 | 30881934 | 199, |
| peak17086 | 2.71 | 1.1749E-05 | 0.000323594 | 4 | 3.63078E-05 | 0.000851138 | chr12 | 30881681 | 30881932 | NM_001319842.1 | 0.000036 | - | 30881681 | 30881932 | 251, |
| peak17087 | 2.04 | 0.001949845 | 0.013182567 | 4.57 | 4.16869E-06 | 0.000131826 | chr12 | 30881773 | 30881923 | NM_001319843.1 | 0.0000042 | - | 30881773 | 30881923 | 150, |
| peak17088 | 2.26 | 0.001778279 | 0.012302688 | 4.57 | 2.0893E-05 | 0.000524807 | chr12 | 30881772 | 30881922 | NM_023925.4 | 0.000021 | - | 30881772 | 30881922 | 150, |
| peak17089 | 1.88 | 0.002187762 | 0.014454398 | 4 | 2.39883E-05 | 0.000588844 | chr12 | 30881776 | 30881926 | NM_032156.4 | 0.000024 | - | 30881776 | 30881926 | 150, |
| peak17090 | 2.35 | 8.12831E-05 | 0.001348963 | 4 | 0.000239883 | 0.004466836 | chr12 | 30881732 | 30881932 | NM_001319844.2 | 0.00024 | - | 30881732 | 30881932 | 200, |
| peak17092 | 1.87 | 0.004168694 | 0.022908677 | 4.57 | 0.000331131 | 0.005888437 | chr12 | 30881768 | 30881918 | NM_001319846.2 | 0.00033 | - | 30881768 | 30881918 | 150, |
| peak17149 | 2.21 | 1.99526E-06 | 8.31764E-05 | 28.7 | 0.004677351 | 0.015848932 | chr12 | 49431603 | 49432203 | NM_003482.3 | 0.0047 | - | 49431603 | 49432203 | 600, |
| peak17186 | 2.86 | 0.002951209 | 0.017782794 | 9 | 4.2658E-05 | 0.000954993 | chr12 | 51457518 | 51457668 | NM_030809.3 | 0.000043 | - | 51457518 | 51457668 | 150, |
| peak17190 | 5.61 | 4.67735E-05 | 0.000912011 | 2 | 4.7863E-05 | 0.001071519 | chr12 | 51457176 | 51457276 | NR_045072.2 | 0.000048 | - | 51457176 | 51457276 | 100, |
| peak17213 | 1.42 | 0.004466836 | 0.023988329 | 8.44 | 6.60693E-07 | 4.89779E-06 | chr12 | 54575281 | 54575480 | NM_001351247.1 | 0.00000066 | - | 54575281 | 54575480 | 199, |
| peak17221 | 1.15 | 0.002187762 | 0.014454398 | 8 | 0.000213796 | 0.000870964 | chr12 | 54575285 | 54575485 | NM_001351252.1 | 0.00021 | - | 54575285 | 54575485 | 200, |
| peak17242 | 1.15 | 0.002187762 | 0.014454398 | 8 | 0.000263027 | 0.001047129 | chr12 | 54575285 | 54575485 | NM_001351260.1 | 0.00026 | - | 54575285 | 54575485 | 200, |
| peak17282 | 3.08 | 0.000537032 | 0.005248075 | 13 | 2.81838E-07 | 2.34423E-06 | chr12 | 57396548 | 57396897 | NM_014830.3 | 0.00000028 | - | 57396548 | 57396897 | 349, |
| peak17283 | 2.19 | 1.94984E-06 | 7.94328E-05 | 12.7 | 2.81838E-07 | 2.34423E-06 | chr12 | 57397344 | 57398041 | NM_014830.3 | 0.00000028 | - | 57397344 | 57398041 | 697, |
| peak17330 | 1.94 | 0.002041738 | 0.013803843 | 12.5 | 5.24807E-06 | 3.0903E-05 | chr12 | 72025805 | 72026676 | NM_144982.5 | 0.0000052 | - | 72025805 | 72026676 | 489,11, |
| peak17406 | 4.76 | 0.000457088 | 0.004677351 | 4.29 | 5.01187E-08 | 3.0903E-06 | chr12 | 93171415 | 93171793 | NM_003566.4 | 0.00000005 | - | 93171415 | 93171793 | 24,77, |
| peak17408 | 2.84 | 0.000676083 | 0.006309573 | 15.6 | 1.09648E-08 | 1.47911E-07 | chr12 | 93192816 | 93202913 | NM_003566.4 | 0.000000011 | - | 93192816 | 93202913 | 46,102,331,121, |
| peak17415 | 2.03 | 0.001148154 | 0.009120108 | 5 | 0.000177828 | 0.003388442 | chr12 | 95603637 | 95603788 | NM_018351.4 | 0.00018 | - | 95603637 | 95603788 | 151, |
| peak17432 | 1.27 | 0.000891251 | 0.007585776 | 10.7 | 0.00047863 | 0.001819701 | chr12 | 102147304 | 102153859 | NM_024312.5 | 0.00048 | - | 102147304 | 102153859 | 13,99,86,52, |
| peak17433 | 1.08 | 0.00057544 | 0.005495409 | 9.95 | 8.31764E-05 | 0.000363078 | chr12 | 102158093 | 102158491 | NM_024312.5 | 0.000083 | - | 102158093 | 102158491 | 398, |
| peak17466 | 5.63 | 5.24807E-06 | 0.000177828 | 10.5 | 2.69153E-06 | 1.69824E-05 | chr12 | 109181404 | 109181604 | NM_018984.4 | 0.0000027 | - | 109181404 | 109181604 | 200, |
| peak17518 | 4.48 | 0.000398107 | 0.004265795 | 5.44 | 8.12831E-05 | 0.000363078 | chr12 | 121676088 | 121676238 | NM_001270486.1 | 0.000081 | - | 121676088 | 121676238 | 150, |
| peak17522 | 4.48 | 0.000398107 | 0.004265795 | 5.44 | 7.94328E-05 | 0.000354813 | chr12 | 121676088 | 121676238 | NM_001270485.2 | 0.000079 | - | 121676088 | 121676238 | 150, |
| peak17525 | 4.48 | 0.000398107 | 0.004265795 | 5.44 | 7.76247E-05 | 0.000346737 | chr12 | 121676089 | 121676238 | NM_172226.2 | 0.000078 | - | 121676089 | 121676238 | 149, |
| peak17527 | 4.36 | 0.00074131 | 0.006606934 | 5.44 | 3.71535E-06 | 2.23872E-05 | chr12 | 121676089 | 121676239 | NM_006549.3 | 0.0000037 | - | 121676089 | 121676239 | 150, |
| peak17529 | 1.34 | 0.002137962 | 0.014454398 | 5.25 | 1.69824E-06 | 1.14815E-05 | chr12 | 121693595 | 121706456 | NM_006549.3 | 0.0000017 | - | 121693595 | 121706456 | 12,37,134,16, |
| peak17541 | 2.32 | 0.000151356 | 0.002089296 | 6.24 | 9.12011E-06 | 4.89779E-05 | chr12 | 122957992 | 122958392 | NM_001350937.2 | 0.0000091 | - | 122957992 | 122958392 | 400, |
| peak17542 | 1.87 | 0.003090295 | 0.018620871 | 7.14 | 9.12011E-06 | 4.89779E-05 | chr12 | 122958491 | 122958692 | NM_001350937.2 | 0.0000091 | - | 122958491 | 122958692 | 201, |
| peak17591 | 4.19 | 0.003548134 | 0.020417379 | 2 | 8.31764E-05 | 0.000239883 | chr12 | 133502534 | 133502684 | NM_183238.4 | 0.000083 | - | 133502534 | 133502684 | 150, |
| peak17623 | 4.31 | 0.000151356 | 0.002089296 | 6.37 | 2.69153E-06 | 1.69824E-05 | chr13 | 20567698 | 20567899 | NM_001190965.3 | 0.0000027 | + | 20567698 | 20567899 | 201, |
| peak17632 | 3.18 | 0.004168694 | 0.022908677 | 6.93 | 6.16595E-05 | 0.000275423 | chr13 | 20567656 | 20576997 | NM_001353161.2 | 0.000062 | + | 20567656 | 20576997 | 48,94,8, |
| peak17666 | 3.47 | 7.58578E-07 | 3.80189E-05 | 8.15 | 3.98107E-98 | 3.16228E-94 | chr13 | 28197091 | 28197438 | NM_015972.4 | 4E-98 | + | 28197091 | 28197438 | 347, |
| peak17685 | 3.29 | 0.000194984 | 0.002511886 | 5.19 | 6.76083E-06 | 3.80189E-05 | chr13 | 42876094 | 42876244 | NM_016248.4 | 0.0000068 | + | 42876094 | 42876244 | 150, |
| peak17716 | 3.36 | 7.58578E-05 | 0.001258925 | 9.25 | 0.000125893 | 0.000537032 | chr13 | 50586331 | 50586929 | NM_001007278.3 | 0.00013 | + | 50586331 | 50586929 | 598, |
| peak17717 | 3.38 | 7.58578E-05 | 0.001258925 | 9.25 | 4.57088E-05 | 0.000213796 | chr13 | 50586331 | 50586929 | NM_005798.5 | 0.000046 | + | 50586331 | 50586929 | 598, |
| peak17718 | 3.17 | 0.002041738 | 0.013803843 | 1.7 | 1.28825E-06 | 4.89779E-05 | chr13 | 50571276 | 50571827 | NM_052811.4 | 0.0000013 | + | 50571276 | 50571827 | 76,25, |
| peak17719 | 3.45 | 8.12831E-05 | 0.001348963 | 12 | 1.28825E-06 | 8.91251E-06 | chr13 | 50586344 | 50586892 | NM_052811.4 | 0.0000013 | + | 50586344 | 50586892 | 548, |
| peak17721 | 3.42 | 3.63078E-05 | 0.000776247 | 8.88 | 0.000107152 | 0.000457088 | chr13 | 50586342 | 50586939 | NM_213590.3 | 0.00011 | + | 50586342 | 50586939 | 597, |
| peak17726 | 2.42 | 2.18776E-05 | 0.000524807 | 12.1 | 0.000630957 | 0.002344229 | chr13 | 52604937 | 52605188 | NR_036571.3 | 0.00063 | + | 52604937 | 52605188 | 251, |
| peak17759 | 1.38 | 7.58578E-06 | 0.000234423 | 13.6 | 9.33254E-06 | 5.01187E-05 | chr13 | 103514511 | 103515111 | NM_001204425.1 | 0.0000093 | + | 103514511 | 103515111 | 600, |
| peak17763 | 1.35 | 3.71535E-05 | 0.000794328 | 9.05 | 0.001513561 | 0.005495409 | chr13 | 103514468 | 103515116 | NM_000123.3 | 0.0015 | + | 103514468 | 103515116 | 648, |
| peak17880 | 4.07 | 0.000630957 | 0.005888437 | 6.08 | 0.000489779 | 0.001862087 | chr13 | 26793672 | 26796168 | NM_183045.1 | 0.00049 | - | 26793672 | 26796168 | 132,83,29, |
| peak17884 | 5.77 | 3.46737E-06 | 0.000128825 | 7.86 | 7.24436E-10 | 1.47911E-08 | chr13 | 26793735 | 26795464 | NM_183044.2 | 7.2E-10 | - | 26793735 | 26795464 | 69,80, |
| peak17888 | 5.79 | 4.46684E-06 | 0.000154882 | 4.92 | 1.58489E-16 | 1.99526E-14 | chr13 | 26793752 | 26796154 | NM_005977.4 | 1.6E-16 | - | 26793752 | 26796154 | 52,83,15, |
| peak17911 | 2.21 | 0.001202264 | 0.009549926 | 2.92 | 7.07946E-07 | 5.24807E-06 | chr13 | 31711606 | 31712589 | NM_001349704.2 | 0.00000071 | - | 31711606 | 31712589 | 55,46, |
| peak17913 | 2.56 | 0.00001 | 0.000288403 | 3.67 | 1.14815E-06 | 8.12831E-06 | chr13 | 31711506 | 31712589 | NM_006644.4 | 0.0000011 | - | 31711506 | 31712589 | 155,46, |
| peak17915 | 2.58 | 6.16595E-06 | 0.000199526 | 3.75 | 2.95121E-06 | 1.86209E-05 | chr13 | 31711499 | 31712582 | NM_001286503.1 | 0.000003 | - | 31711499 | 31712582 | 162,39, |
| peak17917 | 1.83 | 5.12861E-05 | 0.000977237 | 4.3 | 0.002454709 | 0.00851138 | chr13 | 31711496 | 31712578 | NM_001286504.1 | 0.0025 | - | 31711496 | 31712578 | 165,35, |
| peak17919 | 2.58 | 7.24436E-06 | 0.000229087 | 3.58 | 8.31764E-07 | 6.0256E-06 | chr13 | 31711503 | 31712586 | NM_001286505.1 | 0.00000083 | - | 31711503 | 31712586 | 158,43, |
| peak17952 | 1.07 | 3.71535E-05 | 0.000776247 | 9.19 | 1.38038E-07 | 1.28825E-06 | chr13 | 41507542 | 41507890 | NM_001145353.1 | 0.00000014 | - | 41507542 | 41507890 | 348, |
| peak17960 | 1.15 | 1.69824E-06 | 7.24436E-05 | 8.58 | 9.12011E-08 | 8.91251E-07 | chr13 | 41507593 | 41507891 | NM_001370329.1 | 0.000000091 | - | 41507593 | 41507891 | 298, |
| peak17963 | 1.25 | 3.98107E-08 | 2.95121E-06 | 9.95 | 0.00060256 | 0.002238721 | chr13 | 41507604 | 41507904 | NM_001370330.1 | 0.0006 | - | 41507604 | 41507904 | 300, |
| peak17978 | 4.41 | 5.88844E-05 | 0.001071519 | 30.2 | 2.23872E-06 | 1.47911E-05 | chr13 | 45147535 | 45147635 | NM_183422.4 | 0.0000022 | - | 45147535 | 45147635 | 100, |
| peak18137 | 3.69 | 0.004466836 | 0.023988329 | 7.33 | 3.16228E-13 | 1.99526E-11 | chr14 | 39649734 | 39649884 | NM_002687.4 | 3.2E-13 | + | 39649734 | 39649884 | 150, |
| peak18151 | 3.37 | 0.001659587 | 0.011748976 | 3.96 | 3.01995E-05 | 0.000147911 | chr14 | 52195197 | 52195447 | NM_001042481.3 | 0.00003 | + | 52195197 | 52195447 | 250, |

| | | | | | | | | | | | | | | | |
|---|---|---|---|---|---|---|---|---|---|---|---|---|---|---|---|
| peak18154 | 3.81 | 0.000104713 | 0.00162181 | 5.4 | 0.000120226 | 0.000512861 | chr14 | 52195255 | 52195356 | NM_152330.4 | 0.00012 | + | 52195255 | 52195356 | 101, |
| peak18157 | 3.66 | 0.000295121 | 0.003388442 | 8.76 | 0.001318257 | 0.004677351 | chr14 | 52195269 | 52195419 | NM_001267047.1 | 0.0013 | + | 52195269 | 52195419 | 150, |
| peak18164 | 1.48 | 1.1749E-05 | 0.000323594 | 37.9 | 3.46737E-09 | 5.49541E-08 | chr14 | 55509682 | 55510928 | NM_080867.3 | 3.5E-09 | + | 55509682 | 55510928 | 1246, |
| peak18165 | 2.93 | 3.63078E-06 | 0.000131826 | 44 | 0.000489779 | 0.001862087 | chr14 | 55509900 | 55510298 | NM_199421.2 | 0.00049 | + | 55509900 | 55510298 | 398, |
| peak18198 | 2.44 | 0.002398833 | 0.015488166 | 26.2 | 2.95121E-07 | 2.45471E-06 | chr14 | 57741042 | 57741143 | NM_018229.4 | 0.0000003 | + | 57741042 | 57741143 | 101, |
| peak18216 | 2.08 | 0.000131826 | 0.001862087 | 15.8 | 0.000281838 | 0.001122018 | chr14 | 60591184 | 60591385 | NM_001330177.2 | 0.00028 | + | 60591184 | 60591385 | 201, |
| peak18229 | 3.04 | 1.28825E-05 | 0.000354813 | 6.23 | 0.006309573 | 0.020892961 | chr14 | 64586160 | 64586310 | NM_182914.2 | 0.0063 | + | 64586160 | 64586310 | 150, |
| peak18232 | 2 | 0.004897788 | 0.025703958 | 3.67 | 0.003162278 | 0.010715193 | chr14 | 64319771 | 64375826 | NM_015180.5 | 0.0032 | + | 64319771 | 64375826 | 90,11, |
| peak18239 | 3.37 | 1.25893E-05 | 0.000346737 | 5.67 | 0.003981072 | 0.013489629 | chr14 | 64586146 | 64586296 | NM_015180.5 | 0.004 | + | 64586146 | 64586296 | 150, |
| peak18251 | 2.5 | 0.001548817 | 0.011481536 | 2.78 | 0.000776247 | 0.012589254 | chr14 | 65528225 | 65528375 | NM_001202559.1 | 0.00078 | + | 65528225 | 65528375 | 150, |
| peak18296 | 2.47 | 0.003890451 | 0.021877616 | 4.4 | 6.45654E-05 | 0.000288403 | chr14 | 67849245 | 67850118 | NM_004094.5 | 0.000065 | + | 67849245 | 67850118 | 62,87, |
| peak18313 | 2.56 | 3.01995E-07 | 1.77828E-05 | 8.25 | 0.001513561 | 0.005370318 | chr14 | 72054774 | 72055173 | NM_001284245.3 | 0.0015 | + | 72054774 | 72055173 | 399, |
| peak18315 | 2.44 | 7.76247E-06 | 0.000239883 | 6.75 | 0.000812831 | 0.003019952 | chr14 | 72054784 | 72055183 | NM_001354285.2 | 0.00081 | + | 72054784 | 72055183 | 399, |
| peak18316 | 2.72 | 0.000707946 | 0.006456542 | 4.86 | 0.001862087 | 0.028183829 | chr14 | 72055530 | 72055730 | NM_001354285.2 | 0.0019 | + | 72055530 | 72055730 | 200, |
| peak18319 | 2.14 | 2.5704E-05 | 0.00060256 | 9.44 | 0.003467369 | 0.011748976 | chr14 | 72054866 | 72055166 | NM_001354286.2 | 0.0035 | + | 72054866 | 72055166 | 300, |
| peak18323 | 2.37 | 1.28825E-06 | 5.88844E-05 | 5.33 | 0.001348963 | 0.004897788 | chr14 | 72054787 | 72055188 | NM_001354287.2 | 0.0013 | + | 72054787 | 72055188 | 401, |
| peak18324 | 2.68 | 0.000707946 | 0.006456542 | 4.86 | 0.000831764 | 0.013489629 | chr14 | 72055537 | 72055738 | NM_001354287.2 | 0.00083 | + | 72055537 | 72055738 | 201, |
| peak18326 | 2.33 | 1.8197E-06 | 7.58578E-05 | 5.33 | 0.000501187 | 0.001905461 | chr14 | 72054788 | 72055189 | NM_001354288.2 | 0.0005 | + | 72054788 | 72055189 | 401, |
| peak18327 | 2.68 | 0.000707946 | 0.006456542 | 4.86 | 0.000851138 | 0.013803843 | chr14 | 72055537 | 72055738 | NM_001354288.2 | 0.00085 | + | 72055537 | 72055738 | 201, |
| peak18330 | 2.39 | 4.46684E-06 | 0.000154882 | 6.07 | 0.000323594 | 0.00128825 | chr14 | 72054803 | 72055204 | NM_015556.4 | 0.00032 | + | 72054803 | 72055204 | 401, |
| peak18331 | 2.84 | 0.000954993 | 0.008128305 | 3.88 | 0.001071519 | 0.016982437 | chr14 | 72055553 | 72055754 | NM_015556.4 | 0.0011 | + | 72055553 | 72055754 | 201, |
| peak18334 | 2.63 | 7.58578E-07 | 3.80189E-05 | 6.07 | 0.000229087 | 0.000912011 | chr14 | 72054805 | 72055206 | NM_001284247.3 | 0.00023 | + | 72054805 | 72055206 | 401, |
| peak18335 | 3.08 | 0.000162181 | 0.002187762 | 3.88 | 0.000891251 | 0.014125375 | chr14 | 72055554 | 72055755 | NM_001284247.3 | 0.00089 | + | 72055554 | 72055755 | 201, |
| peak18338 | 2.31 | 7.94328E-06 | 0.000245471 | 6.07 | 8.31764E-05 | 0.000363078 | chr14 | 72054803 | 72055203 | NM_001284246.2 | 0.000083 | + | 72054803 | 72055203 | 400, |
| peak18339 | 2.9 | 0.00144544 | 0.010715193 | 4.11 | 0.001174898 | 0.004265795 | chr14 | 72055501 | 72055752 | NM_001284246.2 | 0.0012 | + | 72055501 | 72055752 | 251, |
| peak18350 | 3.78 | 6.76083E-06 | 0.000213796 | 8 | 1.99526E-21 | 6.30957E-19 | chr14 | 74035911 | 74040201 | NM_001364177.1 | 2E-21 | + | 74035911 | 74040201 | 189,110, |
| peak18357 | 2.79 | 0.000316228 | 0.003548134 | 7.18 | 1.65959E-05 | 8.31764E-05 | chr14 | 75276283 | 75276484 | NM_019589.3 | 0.000017 | + | 75276283 | 75276484 | 201, |
| peak18370 | 4.61 | 3.01995E-07 | 1.77828E-05 | 3.83 | 0.00025704 | 0.004786301 | chr14 | 77229360 | 77237580 | NM_014909.5 | 0.00026 | + | 77229360 | 77237580 | 113,89,48, |
| peak18403 | 3.25 | 0.000954993 | 0.007943282 | 3.6 | 1.47911E-07 | 1.38038E-06 | chr14 | 89044457 | 89068316 | NM_001160104.2 | 0.00000015 | + | 89044457 | 89068316 | 27,75,49, |
| peak18406 | 2.88 | 0.003630781 | 0.020892961 | 3.6 | 7.07946E-08 | 7.24436E-07 | chr14 | 89044476 | 89068334 | NM_001326297.2 | 0.000000071 | + | 89044476 | 89068334 | 8,75,67, |
| peak18408 | 2.83 | 0.003090295 | 0.018197009 | 3.6 | 4.67735E-08 | 5.01187E-07 | chr14 | 89044472 | 89068331 | NM_001326300.2 | 0.000000047 | + | 89044472 | 89068331 | 12,75,64, |
| peak18409 | 2.83 | 0.003090295 | 0.018197009 | 3.6 | 4.16869E-08 | 4.67735E-07 | chr14 | 89044472 | 89068331 | NM_001326302.2 | 0.000000042 | + | 89044472 | 89068331 | 12,75,64, |
| peak18414 | 3.25 | 0.000954993 | 0.007943282 | 3.6 | 1.69824E-07 | 1.54882E-06 | chr14 | 89044457 | 89068316 | NM_001326310.2 | 0.00000017 | + | 89044457 | 89068316 | 27,75,49, |
| peak18416 | 2.83 | 0.004466836 | 0.023988329 | 3.6 | 7.76247E-08 | 7.76247E-07 | chr14 | 89044475 | 89068334 | NM_001326315.2 | 0.000000078 | + | 89044475 | 89068334 | 9,75,67, |
| peak18419 | 3.39 | 0.000363078 | 0.003890451 | 3.4 | 1.23027E-07 | 1.14815E-06 | chr14 | 89044454 | 89068313 | NM_024824.5 | 0.00000012 | + | 89044454 | 89068313 | 30,75,46, |
| peak18423 | 2.63 | 0.001348963 | 0.01023293 | 4 | 1.77828E-07 | 1.58489E-06 | chr14 | 89044457 | 89069220 | NR_136936.2 | 0.00000018 | + | 89044457 | 89069220 | 27,75,49, |
| peak18428 | 3.46 | 1.07152E-06 | 5.12861E-05 | 15.8 | 0.000162181 | 0.000676083 | chr14 | 90429607 | 90429958 | NM_001008744.2 | 0.00016 | + | 90429607 | 90429958 | 351, |
| peak18431 | 1.32 | 0.000275423 | 0.003235937 | 2.67 | 1.41254E-09 | 2.5704E-08 | chr14 | 90871596 | 90871895 | NM_001363669.1 | 1.4E-09 | + | 90871596 | 90871895 | 299, |
| peak18433 | 1.33 | 0.000977237 | 0.008128305 | 2.5 | 1.58489E-11 | 5.49541E-10 | chr14 | 90871595 | 90871893 | NM_006888.6 | 1.6E-11 | + | 90871595 | 90871893 | 298, |
| peak18435 | 1.52 | 0.002570396 | 0.016218101 | 2.84 | 1.62181E-09 | 2.88403E-08 | chr14 | 90871623 | 90871923 | NM_001363670.1 | 1.6E-09 | + | 90871623 | 90871923 | 300, |
| peak18529 | 3.01 | 0.000562341 | 0.005495409 | 25.6 | 1.86209E-06 | 1.23027E-05 | chr14 | 103188682 | 103193081 | NM_015156.4 | 0.0000019 | + | 103188682 | 103193081 | 71,277, |
| peak18536 | 1.18 | 0.001995262 | 0.013803843 | 3.53 | 1.02329E-07 | 0.000001 | chr14 | 103803528 | 103804777 | NM_001969.5 | 0.0000001 | + | 103803528 | 103804777 | 36,114, |
| peak18548 | 2.94 | 1.23027E-08 | 0.000001 | 18.5 | 1.02329E-06 | 7.24436E-06 | chr14 | 105267783 | 105268774 | NM_001137601.3 | 0.000001 | + | 105267783 | 105268774 | 991, |
| peak18556 | 2.94 | 0.002818383 | 0.017378008 | 3.47 | 0.001 | 0.003630781 | chr14 | 21897223 | 21897324 | NM_020920.4 | 0.001 | - | 21897223 | 21897324 | 101, |
| peak18560 | 2.62 | 0.000123027 | 0.001778279 | 45.3 | 1.04713E-06 | 7.4131E-06 | chr14 | 21899361 | 21899910 | NM_001170629.2 | 0.000001 | - | 21899361 | 21899910 | 549, |
| peak18628 | 1.6 | 0.000288403 | 0.003311311 | 21 | 7.24436E-06 | 4.0738E-05 | chr14 | 35231152 | 35234034 | NM_013448.3 | 0.0000072 | - | 35231152 | 35234034 | 277,122, |
| peak18629 | 2.93 | 0.00042658 | 0.004466836 | 3.25 | 0.00616595 | 0.020417379 | chr14 | 35245597 | 35245698 | NM_013448.3 | 0.0062 | - | 35245597 | 35245698 | 101, |
| peak18630 | 1.59 | 0.00030903 | 0.003467369 | 21 | 1.41254E-05 | 7.4131E-05 | chr14 | 35231153 | 35234035 | NM_182648.2 | 0.000014 | - | 35231153 | 35234035 | 276,123, |
| peak18631 | 2.93 | 0.00042658 | 0.004466836 | 3.25 | 0.01 | 0.032359366 | chr14 | 35245599 | 35245700 | NM_182648.2 | 0.01 | - | 35245599 | 35245700 | 101, |
| peak18640 | 3.44 | 0.001380384 | 0.010471285 | 4.6 | 4.7863E-05 | 0.000218776 | chr14 | 36159098 | 36190982 | NM_001330075.3 | 0.000048 | - | 36159098 | 36190982 | 111,89, |
| peak18647 | 3.02 | 0.000338844 | 0.003801894 | 4.6 | 8.12831E-05 | 0.000354813 | chr14 | 36159047 | 36191031 | NM_001346248.2 | 0.000081 | - | 36159047 | 36191031 | 162,138, |
| peak18652 | 2.99 | 0.004365158 | 0.023442288 | 5.38 | 7.07946E-05 | 0.000316228 | chr14 | 36159135 | 36191020 | NM_194301.4 | 0.000071 | - | 36159135 | 36191020 | 74,127, |
| peak18670 | 4.04 | 0.004265795 | 0.023442288 | 1.82 | 0.000446684 | 0.001737801 | chr14 | 50262574 | 50266205 | NM_001301732.2 | 0.00045 | - | 50262574 | 50266205 | 88,13, |
| peak18675 | 1.46 | 0.004466836 | 0.023988329 | 1.45 | 2.75423E-08 | 3.23594E-07 | chr14 | 51132320 | 51134675 | NM_021818.4 | 0.000000028 | - | 51132320 | 51134675 | 17,84, |
| peak18677 | 2.32 | 4.7863E-05 | 0.000933254 | 2 | 0.000354813 | 0.001380384 | chr14 | 51206097 | 51208392 | NM_020921.3 | 0.00035 | - | 51206097 | 51208392 | 108,93, |
| peak18695 | 4.39 | 0.000588844 | 0.005623413 | 6 | 0.002137962 | 0.031622777 | chr14 | 53504271 | 53504471 | NM_001160147.2 | 0.0021 | - | 53504271 | 53504471 | 200, |
| peak18699 | 4.42 | 0.000562341 | 0.005495409 | 6 | 0.001778279 | 0.026915348 | chr14 | 53504271 | 53504472 | NM_001160148.2 | 0.0018 | - | 53504271 | 53504472 | 201, |

| | | | | | | | | | | | | | | | |
|---|---|---|---|---|---|---|---|---|---|---|---|---|---|---|---|
| peak18703 | 4.42 | 0.000457088 | 0.004677351 | 6 | 0.002570396 | 0.03801894 | chr14 | 53504269 | 53504470 | NM_030637.3 | 0.0026 | - | 53504269 | 53504470 | 201, |
| peak18744 | 5.34 | 8.12831E-06 | 0.000245471 | 4.21 | 3.46737E-09 | 5.62341E-08 | chr14 | 61747559 | 61747659 | NM_001017970.3 | 3.5E-09 | - | 61747559 | 61747659 | 100, |
| peak18803 | 3.03 | 0.000371535 | 0.004073803 | 5.57 | 5.7544E-05 | 0.00025704 | chr14 | 78140044 | 78140195 | NM_006020.3 | 0.000058 | - | 78140044 | 78140195 | 151, |
| peak18806 | 4 | 5.62341E-06 | 0.000186209 | 4.17 | 9.33254E-06 | 5.12861E-05 | chr14 | 78217725 | 78221457 | NM_001318844.2 | 0.0000093 | - | 78217725 | 78221457 | 98,148, |
| peak18809 | 5 | 5.88844E-05 | 0.001071519 | 3.64 | 0.000812831 | 0.003019952 | chr14 | 78217772 | 78221358 | NM_012245.3 | 0.00081 | - | 78217772 | 78221358 | 51,49, |
| peak18827 | 1.28 | 0.000114815 | 0.001698244 | 13.8 | 1.12202E-07 | 1.07152E-06 | chr14 | 89628846 | 89647076 | NM_005197.4 | 0.00000011 | - | 89628846 | 89647076 | 467,32, |
| peak18829 | 1.28 | 0.000676083 | 0.006309573 | 10.6 | 1.54882E-06 | 1.04713E-05 | chr14 | 89628938 | 89647068 | NM_001085471.2 | 0.0000015 | - | 89628938 | 89647068 | 375,24, |
| peak18860 | 1.69 | 0.001122018 | 0.008912509 | 8.51 | 5.01187E-11 | 1.54882E-09 | chr14 | 93407882 | 93408181 | NM_001142593.3 | 5E-11 | - | 93407882 | 93408181 | 299, |
| peak18861 | 1.61 | 0.000776247 | 0.00691831 | 7.3 | 3.71535E-10 | 8.31764E-09 | chr14 | 93407848 | 93408149 | NM_014216.6 | 3.7E-10 | - | 93407848 | 93408149 | 301, |
| peak18862 | 1.67 | 0.000194984 | 0.002511886 | 8.88 | 6.30957E-20 | 1.25893E-17 | chr14 | 93407861 | 93408161 | NM_001363707.1 | 6.3E-20 | - | 93407861 | 93408161 | 300, |
| peak18871 | 2.27 | 8.70964E-06 | 0.00025704 | 2.57 | 1E-23 | 3.98107E-21 | chr14 | 94849078 | 94849178 | NM_000295.5 | 1E-23 | - | 94849078 | 94849178 | 100, |
| peak18873 | 2.26 | 2.5704E-05 | 0.00060256 | 2.26 | 1.58489E-12 | 7.94328E-11 | chr14 | 94849083 | 94849183 | NM_001002236.3 | 1.6E-12 | - | 94849083 | 94849183 | 100, |
| peak18875 | 2.26 | 8.31764E-06 | 0.000251189 | 2.55 | 6.30957E-11 | 1.86209E-09 | chr14 | 94849081 | 94849180 | NM_001127705.2 | 6.3E-11 | - | 94849081 | 94849180 | 99, |
| peak18879 | 1.8 | 0.000776247 | 0.00691831 | 17.7 | 0.002089296 | 0.007413102 | chr14 | 95569820 | 95570419 | NM_001271282.3 | 0.0021 | - | 95569820 | 95570419 | 599, |
| peak18883 | 1.78 | 0.001318257 | 0.01023293 | 17.7 | 0.001479108 | 0.005248075 | chr14 | 95569818 | 95570417 | NM_001291628.1 | 0.0015 | - | 95569818 | 95570417 | 599, |
| peak18895 | 1.62 | 0.003162278 | 0.018620871 | 5.52 | 5.49541E-06 | 3.16228E-05 | chr14 | 99981143 | 99982545 | NM_001144995.2 | 0.0000055 | - | 99981143 | 99982545 | 529,21, |
| peak18918 | 3.53 | 7.76247E-05 | 0.00128825 | 1.88 | 1.8197E-05 | 9.12011E-05 | chr14 | 103406003 | 103406256 | NM_006035.4 | 0.000018 | - | 103406003 | 103406256 | 61,90, |
| peak18946 | 2.04 | 3.16228E-06 | 0.00011749 | 33.6 | 2.13796E-10 | 5.24807E-09 | chr14 | 105477507 | 105478207 | NM_017955.4 | 2.1E-10 | - | 105477507 | 105478207 | 700, |
| peak18964 | 1.19 | 0.000457088 | 0.004677351 | 6.25 | 1.94984E-06 | 1.28825E-05 | chr14 | 105685345 | 105688144 | NM_001242788.2 | 0.0000019 | - | 105685345 | 105688144 | 45,82,62,160, |
| peak19002 | 2.11 | 0.000218776 | 0.002754229 | 106 | 1.58489E-14 | 1.58489E-12 | chr15 | 38643325 | 38643724 | NM_152594.3 | 1.6E-14 | + | 38643325 | 38643724 | 399, |
| peak19017 | 3.98 | 3.23594E-07 | 1.86209E-05 | 11.2 | 9.77237E-05 | 0.00042658 | chr15 | 41625226 | 41648250 | NM_001243143.2 | 0.000098 | + | 41625226 | 41648250 | 22,69,144,97,14, |
| peak19025 | 2.92 | 9.33254E-06 | 0.000275423 | 11 | 0.009120108 | 0.030199517 | chr15 | 42042432 | 42042632 | NM_001080541.2 | 0.0091 | + | 42042432 | 42042632 | 200, |
| peak19026 | 2.22 | 0.004365158 | 0.023442288 | 11.8 | 0.002691535 | 0.009332543 | chr15 | 42058601 | 42058802 | NM_001080541.2 | 0.0027 | + | 42058601 | 42058802 | 201, |
| peak19032 | 2.78 | 0.000295121 | 0.003388442 | 9.1 | 0.008912509 | 0.029512092 | chr15 | 42042519 | 42042620 | NM_001164273.1 | 0.0089 | + | 42042519 | 42042620 | 101, |
| peak19125 | 1.6 | 0.000295121 | 0.003388442 | 10 | 3.31131E-09 | 5.37032E-08 | chr15 | 59323294 | 59323843 | NM_001270528.2 | 3.3E-09 | + | 59323294 | 59323843 | 549, |
| peak19127 | 3.04 | 0.002818383 | 0.017378008 | 7 | 3.31131E-09 | 5.37032E-08 | chr15 | 59373403 | 59377874 | NM_001270528.2 | 3.3E-09 | + | 59373403 | 59377874 | 80,153,17, |
| peak19130 | 1.52 | 0.000141254 | 0.001995262 | 8.25 | 2.13796E-09 | 3.71535E-08 | chr15 | 59323241 | 59323837 | NM_001270529.2 | 2.1E-09 | + | 59323241 | 59323837 | 596, |
| peak19132 | 2.47 | 0.001071519 | 0.008709636 | 7.14 | 2.13796E-09 | 3.71535E-08 | chr15 | 59373293 | 59377889 | NM_001270529.2 | 2.1E-09 | + | 59373293 | 59377889 | 190,126,32, |
| peak19135 | 1.7 | 8.31764E-05 | 0.001380384 | 8.25 | 1.8197E-09 | 3.23594E-08 | chr15 | 59323241 | 59323838 | NM_001330331.2 | 1.8E-09 | + | 59323241 | 59323838 | 597, |
| peak19137 | 2.74 | 0.000151356 | 0.002137962 | 7.17 | 1.8197E-09 | 3.23594E-08 | chr15 | 59373292 | 59377911 | NM_001330331.2 | 1.8E-09 | + | 59373292 | 59377911 | 191,153,54, |
| peak19140 | 1.71 | 0.000102329 | 0.001548817 | 8.25 | 1.77828E-09 | 3.16228E-08 | chr15 | 59323241 | 59323837 | NM_017610.8 | 1.8E-09 | + | 59323241 | 59323837 | 596, |
| peak19142 | 2.17 | 0.002187762 | 0.014454398 | 6.25 | 1.77828E-09 | 3.16228E-08 | chr15 | 59373291 | 59377888 | NM_017610.8 | 1.8E-09 | + | 59373291 | 59377888 | 192,126,31, |
| peak19144 | 1.54 | 0.000138038 | 0.001949845 | 8.25 | 2.69153E-09 | 4.46684E-08 | chr15 | 59323290 | 59323840 | NM_001270530.1 | 2.7E-09 | + | 59323290 | 59323840 | 550, |
| peak19146 | 2.82 | 0.000467735 | 0.004677351 | 7 | 2.69153E-09 | 4.46684E-08 | chr15 | 59373399 | 59377897 | NM_001270530.1 | 2.7E-09 | + | 59373399 | 59377897 | 84,126,40, |
| peak19208 | 1.18 | 0.004570882 | 0.023988329 | 15 | 0.001047129 | 0.003801894 | chr15 | 67482982 | 67483480 | NM_005902.4 | 0.001 | + | 67482982 | 67483480 | 498, |
| peak19210 | 1.4 | 0.000223872 | 0.002754229 | 6.73 | 1.77828E-05 | 9.12011E-05 | chr15 | 68378670 | 68379070 | NM_016166.2 | 0.000018 | + | 68378670 | 68379070 | 400, |
| peak19269 | 2.51 | 0.001047129 | 0.008709636 | 2.29 | 0.00017378 | 0.003311311 | chr15 | 81592595 | 81592745 | NM_001172128.2 | 0.00017 | + | 81592595 | 81592745 | 150, |
| peak19274 | 2.51 | 0.001047129 | 0.008709636 | 2.29 | 0.000281838 | 0.005011872 | chr15 | 81592593 | 81592744 | NM_001352684.2 | 0.00028 | + | 81592593 | 81592744 | 151, |
| peak19297 | 4.21 | 1.23027E-05 | 0.000338844 | 2.7 | 0.000346737 | 0.006025596 | chr15 | 85345133 | 85345284 | NM_014630.3 | 0.00035 | + | 85345133 | 85345284 | 151, |
| peak19299 | 1.77 | 4.89779E-05 | 0.000954993 | 18.4 | 2.69153E-06 | 1.69824E-05 | chr15 | 86122441 | 86122891 | NM_006738.6 | 0.0000027 | + | 86122441 | 86122891 | 450, |
| peak19300 | 2.12 | 1.58489E-07 | 1.02329E-05 | 22.4 | 6.45654E-05 | 0.000288403 | chr15 | 86123290 | 86124240 | NM_006738.6 | 0.000065 | + | 86123290 | 86124240 | 950, |
| peak19306 | 1.86 | 0.000234423 | 0.002818383 | 20.3 | 2.23872E-06 | 1.44544E-05 | chr15 | 86122440 | 86122890 | NM_007200.5 | 0.0000022 | + | 86122440 | 86122890 | 450, |
| peak19307 | 1.87 | 4.7863E-07 | 2.63027E-05 | 21.9 | 0.000436516 | 0.001659587 | chr15 | 86123288 | 86123938 | NM_007200.5 | 0.00044 | + | 86123288 | 86123938 | 650, |
| peak19485 | 3.66 | 0.000380189 | 0.004168694 | 3 | 1.31826E-08 | 1.69824E-07 | chr15 | 34445139 | 34446863 | NM_024713.3 | 0.000000013 | - | 34445139 | 34446863 | 131,19, |
| peak19489 | 4.46 | 0.003388442 | 0.019498446 | 2.25 | 5.12861E-07 | 2.23872E-05 | chr15 | 37183945 | 37184046 | NM_172315.3 | 0.00000051 | - | 37183945 | 37184046 | 101, |
| peak19496 | 4.46 | 0.003388442 | 0.019498446 | 2.25 | 9.54993E-06 | 0.000269153 | chr15 | 37183945 | 37184046 | NM_170677.5 | 0.0000095 | - | 37183945 | 37184046 | 101, |
| peak19506 | 6.63 | 8.51138E-06 | 0.00025704 | 11.5 | 0.001258925 | 0.004570882 | chr15 | 41379840 | 41384334 | NR_104038.1 | 0.0013 | - | 41379840 | 41384334 | 40,110, |
| peak19509 | 5.63 | 2.04174E-06 | 8.31764E-05 | 11.3 | 0.001479108 | 0.005248075 | chr15 | 41379773 | 41384366 | NM_017553.3 | 0.0015 | - | 41379773 | 41384366 | 107,142, |
| peak19549 | 2.2 | 0.002570396 | 0.016218101 | 5.8 | 8.70964E-06 | 4.67735E-05 | chr15 | 43661194 | 43662010 | NM_152455.3 | 0.0000087 | - | 43661194 | 43662010 | 131,217, |
| peak19552 | 1.96 | 0.001412538 | 0.010715193 | 5.6 | 3.01995E-06 | 0.0001 | chr15 | 43661198 | 43662064 | NM_001372080.1 | 0.000003 | - | 43661198 | 43662064 | 127,271, |
| peak19559 | 4.51 | 0.000891251 | 0.007585776 | 16.3 | 0.014454398 | 0.046773514 | chr15 | 44876155 | 44876455 | NM_001160227.2 | 0.014 | - | 44876155 | 44876455 | 300, |
| peak19630 | 2.28 | 0.001412538 | 0.010471285 | 6.29 | 2.69153E-05 | 0.000131826 | chr15 | 59179193 | 59179449 | NM_001013843.3 | 0.000027 | - | 59179193 | 59179449 | 125,25, |
| peak19634 | 2.28 | 0.001412538 | 0.010471285 | 6.29 | 0.000588844 | 0.002238721 | chr15 | 59179194 | 59179450 | NM_024755.4 | 0.00059 | - | 59179194 | 59179450 | 124,26, |
| peak19668 | 2.56 | 0.000251189 | 0.003019952 | 3.58 | 1.94984E-06 | 1.28825E-05 | chr15 | 62207978 | 62208178 | NM_020821.3 | 0.0000019 | - | 62207978 | 62208178 | 200, |
| peak19672 | 2.84 | 6.91831E-05 | 0.001202264 | 2.81 | 6.76083E-09 | 9.77237E-08 | chr15 | 62207969 | 62208170 | NM_001018088.2 | 6.8E-09 | - | 62207969 | 62208170 | 201, |
| peak19677 | 2.71 | 0.000114815 | 0.001698244 | 2.81 | 3.80189E-07 | 3.0903E-06 | chr15 | 62208006 | 62208157 | NM_018080.3 | 0.00000038 | - | 62208006 | 62208157 | 151, |
| peak19683 | 2.64 | 0.000346737 | 0.003801894 | 2.4 | 0.000199526 | 0.000812831 | chr15 | 64067519 | 64067620 | NM_003922.4 | 0.0002 | - | 64067519 | 64067620 | 101, |

| | | | | | | | | | | | | | | | |
|---|---|---|---|---|---|---|---|---|---|---|---|---|---|---|---|
| peak19761 | 1.24 | 0.002818383 | 0.017378008 | 36.2 | 3.71535E-07 | 3.01995E-06 | chr15 | 75663711 | 75668113 | NM_001145358.2 | 0.00000037 | - | 75663711 | 75668113 | 839,108, |
| peak19765 | 1.25 | 0.002290868 | 0.014791084 | 38.6 | 1.41254E-06 | 9.54993E-06 | chr15 | 75663715 | 75668069 | NM_015477.3 | 0.0000014 | - | 75663715 | 75668069 | 835,64, |
| peak19767 | 1.94 | 0.000660693 | 0.00616595 | 4.56 | 0.001148154 | 0.004168694 | chr15 | 75684703 | 75687117 | NM_015477.3 | 0.0011 | - | 75684703 | 75687117 | 453,97, |
| peak19769 | 1.26 | 0.001995262 | 0.013489629 | 38.6 | 4.7863E-06 | 2.81838E-05 | chr15 | 75663714 | 75668068 | NM_001145357.2 | 0.0000048 | - | 75663714 | 75668068 | 836,63, |
| peak19771 | 1.93 | 0.000645654 | 0.00616595 | 4.56 | 0.00128825 | 0.004570882 | chr15 | 75684703 | 75687116 | NM_001145357.2 | 0.0013 | - | 75684703 | 75687116 | 453,96, |
| peak19803 | 1.53 | 0.00144544 | 0.010715193 | 4.25 | 1.8197E-07 | 1.62181E-06 | chr15 | 77407371 | 77407522 | NM_024776.4 | 0.00000018 | - | 77407371 | 77407522 | 151, |
| peak19816 | 2.97 | 0.004786301 | 0.025118864 | 7.56 | 0.001659587 | 0.005888437 | chr15 | 82443875 | 82444323 | NM_001040610.2 | 0.0017 | - | 82443875 | 82444323 | 448, |
| peak19830 | 2.39 | 0.002089296 | 0.014125375 | 3.41 | 0.001819701 | 0.006456542 | chr15 | 90378523 | 90378674 | NM_005829.5 | 0.0018 | - | 90378523 | 90378674 | 151, |
| peak19831 | 2.38 | 0.002089296 | 0.014125375 | 3.41 | 0.001071519 | 0.003890451 | chr15 | 90378520 | 90378671 | NR_037582.2 | 0.0011 | - | 90378520 | 90378671 | 151, |
| peak19836 | 1.93 | 1.58489E-05 | 0.000416869 | 3.41 | 3.89045E-05 | 0.00018197 | chr15 | 91525100 | 91527335 | NM_003981.4 | 0.000039 | - | 91525100 | 91527335 | 111,88, |
| peak19869 | 1.71 | 0.003981072 | 0.021877616 | 8.13 | 0.002238721 | 0.007943282 | chr15 | 101718262 | 101719006 | NM_014918.5 | 0.0022 | - | 101718262 | 101719006 | 744, |
| peak19871 | 2.26 | 0.000223872 | 0.002754229 | 8.87 | 1.07152E-05 | 5.62341E-05 | chr16 | 597777 | 597928 | NM_005632.3 | 0.000011 | + | 597777 | 597928 | 151, |
| peak19885 | 3.4 | 1.44544E-05 | 0.000380189 | 6.37 | 1.86209E-06 | 1.23027E-05 | chr16 | 1270372 | 1270722 | NM_021098.3 | 0.0000019 | + | 1270372 | 1270722 | 350, |
| peak19896 | 1.86 | 0.000537032 | 0.005248075 | 4.35 | 1.04713E-09 | 1.99526E-08 | chr16 | 2087938 | 2088088 | NM_004785.6 | 0.000000001 | + | 2087938 | 2088088 | 150, |
| peak19897 | 1.87 | 3.46737E-07 | 1.99526E-05 | 4.35 | 0.002691535 | 0.009332543 | chr16 | 2086976 | 2088088 | NM_001252073.2 | 0.0027 | + | 2086976 | 2088088 | 23,63,262, |
| peak19901 | 1.62 | 1.62181E-05 | 0.000416869 | 13.9 | 8.51138E-10 | 1.65959E-08 | chr16 | 2546040 | 2546538 | NM_001199107.2 | 8.5E-10 | + | 2546040 | 2546538 | 498, |
| peak19904 | 1.61 | 5.49541E-05 | 0.001023293 | 13.9 | 1.25893E-09 | 2.34423E-08 | chr16 | 2546040 | 2546590 | NM_020705.3 | 1.3E-09 | + | 2546040 | 2546590 | 550, |
| peak19918 | 3.45 | 1.51356E-07 | 9.77237E-06 | 1.19 | 3.23594E-05 | 0.000154882 | chr16 | 2815135 | 2815236 | NM_016333.4 | 0.000032 | + | 2815135 | 2815236 | 101, |
| peak19953 | 2.29 | 0.002041738 | 0.013803843 | 4.06 | 1.69824E-05 | 8.70964E-05 | chr16 | 3529569 | 3533431 | NM_001083601.3 | 0.000017 | + | 3529569 | 3533431 | 31,97,69, |
| peak19991 | 6.64 | 6.91831E-05 | 0.001202264 | 6.67 | 0.005370318 | 0.017782794 | chr16 | 4921239 | 4922922 | NM_001288656.1 | 0.0054 | + | 4921239 | 4922922 | 63,38, |
| peak19993 | 3.36 | 0.004786301 | 0.025118864 | 11.8 | 4.16869E-09 | 6.45654E-08 | chr16 | 5134830 | 5135081 | NM_019109.5 | 4.2E-09 | + | 5134830 | 5135081 | 251, |
| peak19994 | 4.65 | 2.81838E-08 | 2.13796E-06 | 7.75 | 4.16869E-09 | 3.80189E-07 | chr16 | 5135280 | 5135630 | NM_019109.5 | 4.2E-09 | + | 5135280 | 5135630 | 350, |
| peak19996 | 4.83 | 5.88844E-08 | 4.2658E-06 | 7.75 | 5.7544E-06 | 0.00017378 | chr16 | 5135334 | 5135635 | NM_001330504.1 | 0.0000058 | + | 5135334 | 5135635 | 301, |
| peak20004 | 1.38 | 0.000162181 | 0.002187762 | 5.33 | 1.54882E-06 | 1.07152E-05 | chr16 | 10997631 | 11002949 | NM_001286403.2 | 0.0000015 | + | 10997631 | 11002949 | 121,69,110, |
| peak20009 | 1.4 | 0.000186209 | 0.002398833 | 5.33 | 6.30957E-06 | 3.63078E-05 | chr16 | 10997633 | 11004106 | NR_104444.2 | 0.0000063 | + | 10997633 | 11004106 | 119,69,62, |
| peak20028 | 6.54 | 3.0903E-05 | 0.000691831 | 1.71 | 4.89779E-09 | 7.58578E-08 | chr16 | 14354844 | 14354945 | NM_001365412.2 | 4.9E-09 | + | 14354844 | 14354945 | 101, |
| peak20034 | 6.2 | 0.00020893 | 0.002691535 | 2.16 | 6.16595E-06 | 3.54813E-05 | chr16 | 14354846 | 14354947 | NM_001365411.2 | 0.0000062 | + | 14354846 | 14354947 | 101, |
| peak20038 | 6.4 | 8.51138E-05 | 0.001380384 | 1.45 | 8.12831E-05 | 0.000363078 | chr16 | 14354839 | 14354940 | NM_001365413.2 | 0.000081 | + | 14354839 | 14354940 | 101, |
| peak20042 | 6.4 | 8.51138E-05 | 0.001380384 | 1.45 | 1.8197E-07 | 1.62181E-06 | chr16 | 14354839 | 14354940 | NM_001365414.2 | 0.00000018 | + | 14354839 | 14354940 | 101, |
| peak20068 | 4.73 | 0.002818383 | 0.017378008 | 22.2 | 1E-11 | 3.89045E-10 | chr16 | 19278563 | 19278713 | NM_001330509.1 | 1E-11 | + | 19278563 | 19278713 | 150, |
| peak20070 | 4.73 | 0.002818383 | 0.017378008 | 22.2 | 6.30957E-12 | 2.45471E-10 | chr16 | 19278558 | 19278708 | NM_016524.4 | 6.3E-12 | + | 19278558 | 19278708 | 150, |
| peak20072 | 4.52 | 0.002630268 | 0.016595869 | 18.5 | 2.51189E-12 | 1.04713E-10 | chr16 | 19278555 | 19278706 | NM_001308157.2 | 2.5E-12 | + | 19278555 | 19278706 | 151, |
| peak20138 | 3 | 0.004897788 | 0.025703958 | 2.6 | 4.46684E-05 | 0.001023293 | chr16 | 23687199 | 23687399 | NM_032486.4 | 0.000045 | + | 23687199 | 23687399 | 200, |
| peak20144 | 3 | 0.004897788 | 0.025703958 | 2.6 | 8.31764E-05 | 0.001737801 | chr16 | 23687199 | 23687399 | NR_037573.2 | 0.000083 | + | 23687199 | 23687399 | 200, |
| peak20156 | 1.93 | 0.003981072 | 0.021877616 | 3.71 | 2.88403E-05 | 0.000141254 | chr16 | 24802694 | 24802894 | NM_001351850.2 | 0.000029 | + | 24802694 | 24802894 | 200, |
| peak20163 | 1.87 | 0.001071519 | 0.008709636 | 2.83 | 0.000223872 | 0.000891251 | chr16 | 24802670 | 24802870 | NM_001330520.3 | 0.00022 | + | 24802670 | 24802870 | 200, |
| peak20170 | 2.09 | 0.000831764 | 0.00724436 | 3.67 | 0.000154882 | 0.000645654 | chr16 | 24802669 | 24802869 | NM_014494.4 | 0.00015 | + | 24802669 | 24802869 | 200, |
| peak20183 | 2.74 | 0.003548134 | 0.020417379 | 9.17 | 0.001380384 | 0.005011872 | chr16 | 27790145 | 27790395 | NM_015202.4 | 0.0014 | + | 27790145 | 27790395 | 250, |
| peak20203 | 1.23 | 0.001174898 | 0.009332543 | 10.4 | 5.12861E-07 | 3.98107E-06 | chr16 | 30409539 | 30409838 | NM_152652.3 | 0.00000051 | + | 30409539 | 30409838 | 299, |
| peak20208 | 3.71 | 5.88844E-06 | 0.000190546 | 1.59 | 0.001230269 | 0.004466836 | chr16 | 30721307 | 30722083 | NM_006662.3 | 0.0012 | + | 30721307 | 30722083 | 142,9, |
| peak20249 | 3.63 | 3.38844E-05 | 0.00074131 | 2.13 | 0.000288403 | 0.001148154 | chr16 | 31092849 | 31094319 | NM_014699.4 | 0.00029 | + | 31092849 | 31094319 | 173,28, |
| peak20258 | 1.37 | 0.000389045 | 0.004168694 | 25.2 | 6.0256E-08 | 6.30957E-07 | chr16 | 48295351 | 48303934 | NM_001300948.3 | 0.00000006 | + | 48295351 | 48303934 | 147,95,8, |
| peak20260 | 1.35 | 3.63078E-06 | 0.000131826 | 36.1 | 2.0893E-07 | 1.8197E-06 | chr16 | 48292638 | 48304005 | NM_031490.5 | 0.00000021 | + | 48292638 | 48304005 | 13,164,95,79, |
| peak20269 | 2.64 | 0.002344229 | 0.015135612 | 4.12 | 4.46684E-06 | 2.63027E-05 | chr16 | 50187965 | 50188116 | NM_001365324.3 | 0.0000045 | + | 50187965 | 50188116 | 151, |
| peak20272 | 2.08 | 0.00162181 | 0.011748976 | 3.07 | 1.47911E-05 | 7.58578E-05 | chr16 | 50828175 | 50828276 | NM_015247.2 | 0.000015 | + | 50828175 | 50828276 | 101, |
| peak20275 | 2.04 | 0.003235937 | 0.019054607 | 3.78 | 4.0738E-06 | 2.45471E-05 | chr16 | 50828164 | 50828264 | NM_001042355.2 | 0.0000041 | + | 50828164 | 50828264 | 100, |
| peak20287 | 2.04 | 0.000436516 | 0.004570882 | 1.55 | 0.000204174 | 0.000831764 | chr16 | 53191236 | 53191336 | NM_001352127.2 | 0.0002 | + | 53191236 | 53191336 | 100, |
| peak20335 | 3 | 0.000831764 | 0.00724436 | 2.33 | 4.2658E-08 | 2.75423E-06 | chr16 | 56852645 | 56857624 | NM_001242795.2 | 0.000000043 | + | 56852645 | 56857624 | 5,90,6, |
| peak20367 | 3.5 | 0.000562341 | 0.005495409 | 14.4 | 4.36516E-07 | 3.38844E-06 | chr16 | 58552659 | 58552808 | NM_024860.3 | 0.00000044 | + | 58552659 | 58552808 | 149, |
| peak20370 | 3.08 | 0.002041738 | 0.013803843 | 3.71 | 7.58578E-07 | 5.62341E-06 | chr16 | 66918342 | 66918542 | NM_001329928.2 | 0.00000076 | + | 66918342 | 66918542 | 200, |
| peak20371 | 3.32 | 0.00018197 | 0.002398833 | 8 | 7.58578E-07 | 5.62341E-06 | chr16 | 66918790 | 66918990 | NM_001329928.2 | 0.00000076 | + | 66918790 | 66918990 | 200, |
| peak20378 | 3.08 | 0.002041738 | 0.013803843 | 3.71 | 4.46684E-07 | 3.46737E-06 | chr16 | 66918339 | 66918539 | NM_001329930.2 | 0.00000045 | + | 66918339 | 66918539 | 200, |
| peak20391 | 3.31 | 0.000158489 | 0.002137962 | 8 | 6.76083E-07 | 5.01187E-06 | chr16 | 66918796 | 66918996 | NM_001329933.2 | 0.00000068 | + | 66918796 | 66918996 | 200, |
| peak20395 | 1.68 | 0.000537032 | 0.005370318 | 7 | 0.000001 | 7.07946E-06 | chr16 | 66918754 | 66919005 | NM_001329934.2 | 0.000001 | + | 66918754 | 66919005 | 251, |
| peak20413 | 2.85 | 1.99526E-07 | 1.23027E-05 | 1.38 | 3.16228E-41 | 3.16228E-38 | chr16 | 67902484 | 67904833 | NM_001322040.2 | 3.2E-41 | + | 67902484 | 67904833 | 18,131, |
| peak20415 | 2.01 | 3.80189E-06 | 0.000138038 | 4.6 | 1.62181E-06 | 1.09648E-05 | chr16 | 67927256 | 67942865 | NM_006742.3 | 0.0000016 | + | 67927256 | 67942865 | 18,283, |
| peak20422 | 3.38 | 0.000457088 | 0.004677351 | 3.88 | 2.45471E-05 | 0.000120226 | chr16 | 68597161 | 68597311 | NM_001305203.2 | 0.000025 | + | 68597161 | 68597311 | 150, |

| | | | | | | | | | | | | | | | |
|---|---|---|---|---|---|---|---|---|---|---|---|---|---|---|---|
| peak20423 | 3.38 | 0.00047863 | 0.004897788 | 3.88 | 5.88844E-05 | 0.000263027 | chr16 | 68597159 | 68597309 | NM_001305207.2 | 0.000059 | + | 68597159 | 68597309 | 150, |
| peak20424 | 3.56 | 0.000549541 | 0.005370318 | 4.43 | 3.71535E-05 | 0.00017378 | chr16 | 68597165 | 68597315 | NM_001305208.2 | 0.000037 | + | 68597165 | 68597315 | 150, |
| peak20425 | 3.41 | 0.003467369 | 0.019952623 | 6.4 | 0.000616595 | 0.002290868 | chr16 | 68597169 | 68597270 | NM_133458.4 | 0.00062 | + | 68597169 | 68597270 | 101, |
| peak20426 | 3.38 | 0.000457088 | 0.004677351 | 3.88 | 1.65959E-05 | 8.51138E-05 | chr16 | 68597162 | 68597312 | NR_130976.2 | 0.000017 | + | 68597162 | 68597312 | 150, |
| peak20427 | 3.38 | 0.000457088 | 0.004677351 | 3.88 | 1.86209E-05 | 9.33254E-05 | chr16 | 68597162 | 68597312 | NR_130977.2 | 0.000019 | + | 68597162 | 68597312 | 150, |
| peak20430 | 5.23 | 0.00144544 | 0.010715193 | 5.33 | 0.001148154 | 0.004168694 | chr16 | 69725972 | 69726122 | NM_173215.3 | 0.0011 | + | 69725972 | 69726122 | 150, |
| peak20431 | 3.1 | 0.000204174 | 0.002570396 | 7.23 | 0.001737801 | 0.006025596 | chr16 | 69727166 | 69727565 | NM_173215.3 | 0.0017 | + | 69727166 | 69727565 | 399, |
| peak20433 | 4.88 | 9.77237E-05 | 0.001513561 | 5.45 | 1.99526E-06 | 1.31826E-05 | chr16 | 69725942 | 69726193 | NM_001113178.3 | 0.000002 | + | 69725942 | 69726193 | 251, |
| peak20434 | 3.61 | 0.00040738 | 0.004365158 | 8.73 | 1.99526E-06 | 1.31826E-05 | chr16 | 69727241 | 69727541 | NM_001113178.3 | 0.000002 | + | 69727241 | 69727541 | 300, |
| peak20437 | 5.13 | 9.12011E-05 | 0.001479108 | 6.83 | 8.12831E-06 | 4.46684E-05 | chr16 | 69725962 | 69726212 | NM_001367709.1 | 0.0000081 | + | 69725962 | 69726212 | 250, |
| peak20438 | 3.28 | 0.000162181 | 0.002187762 | 7.62 | 8.12831E-06 | 4.46684E-05 | chr16 | 69727158 | 69727558 | NM_001367709.1 | 0.0000081 | + | 69727158 | 69727558 | 400, |
| peak20441 | 4.81 | 0.00011749 | 0.001737801 | 5.45 | 1.99526E-06 | 1.31826E-05 | chr16 | 69725943 | 69726194 | NM_006599.4 | 0.000002 | + | 69725943 | 69726194 | 251, |
| peak20442 | 3.58 | 0.000676083 | 0.006309573 | 8.73 | 1.99526E-06 | 1.31826E-05 | chr16 | 69727241 | 69727542 | NM_006599.4 | 0.000002 | + | 69727241 | 69727542 | 301, |
| peak20445 | 5.09 | 0.000147911 | 0.002089296 | 5.45 | 2.0893E-06 | 1.38038E-05 | chr16 | 69725940 | 69726141 | NM_138713.4 | 0.0000021 | + | 69725940 | 69726141 | 201, |
| peak20446 | 3.64 | 0.000346737 | 0.003890451 | 8.04 | 2.0893E-06 | 1.38038E-05 | chr16 | 69727239 | 69727539 | NM_138713.4 | 0.0000021 | + | 69727239 | 69727539 | 300, |
| peak20449 | 5.14 | 0.000331131 | 0.003715352 | 6.08 | 2.18776E-06 | 1.44544E-05 | chr16 | 69725970 | 69726220 | NM_138714.4 | 0.0000022 | + | 69725970 | 69726220 | 250, |
| peak20450 | 3.23 | 0.00018197 | 0.002344229 | 7.23 | 2.18776E-06 | 1.44544E-05 | chr16 | 69727166 | 69727565 | NM_138714.4 | 0.0000022 | + | 69727166 | 69727565 | 399, |
| peak20453 | 4.9 | 0.000363078 | 0.003981072 | 8.67 | 3.31131E-06 | 2.04174E-05 | chr16 | 69725933 | 69726134 | NM_173214.3 | 0.0000033 | + | 69725933 | 69726134 | 201, |
| peak20454 | 3.38 | 0.000446684 | 0.004570882 | 6.68 | 3.31131E-06 | 2.04174E-05 | chr16 | 69727183 | 69727534 | NM_173214.3 | 0.0000033 | + | 69727183 | 69727534 | 351, |
| peak20483 | 1.89 | 0.00018197 | 0.002398833 | 15.3 | 2.39883E-05 | 0.000120226 | chr16 | 71961611 | 71961861 | NM_001270978.2 | 0.000024 | + | 71961611 | 71961861 | 250, |
| peak20487 | 4.17 | 0.000281838 | 0.003235937 | 2.29 | 1.54882E-05 | 0.00040738 | chr16 | 72057067 | 72057168 | NM_001361.5 | 0.000015 | + | 72057067 | 72057168 | 101, |
| peak20491 | 1.95 | 0.003019952 | 0.018197009 | 7.17 | 3.0903E-05 | 0.000147911 | chr16 | 74338204 | 74339248 | NM_002811.5 | 0.000031 | + | 74338204 | 74339248 | 88,62, |
| peak20501 | 2.42 | 2.69153E-07 | 1.62181E-05 | 4.54 | 0.00017378 | 0.000724436 | chr16 | 77228353 | 77228603 | NM_001286639.2 | 0.00017 | + | 77228353 | 77228603 | 250, |
| peak20504 | 2.35 | 1.02329E-06 | 4.89779E-05 | 4.98 | 0.000190546 | 0.000776247 | chr16 | 77228361 | 77228611 | NM_001286640.2 | 0.00019 | + | 77228361 | 77228611 | 250, |
| peak20507 | 1.4 | 0.000104713 | 0.001584893 | 5.31 | 0.00074131 | 0.002754229 | chr16 | 77228372 | 77228572 | NM_014940.4 | 0.00074 | + | 77228372 | 77228572 | 200, |
| peak20520 | 3.36 | 0.001819701 | 0.012882496 | 3.43 | 1.12202E-10 | 1.86209E-08 | chr16 | 83830262 | 83830362 | NM_001220490.2 | 1.1E-10 | + | 83830262 | 83830362 | 100, |
| peak20525 | 3.86 | 2.04174E-06 | 8.31764E-05 | 3.89 | 2.69153E-08 | 3.23594E-07 | chr16 | 84684517 | 84690608 | NM_001303451.2 | 0.000000027 | + | 84684517 | 84690608 | 19,132, |
| peak20527 | 3.86 | 1.99526E-06 | 8.12831E-05 | 3.89 | 4.89779E-09 | 7.58578E-08 | chr16 | 84684517 | 84690607 | NM_024731.4 | 4.9E-09 | + | 84684517 | 84690607 | 19,131, |
| peak20535 | 1.92 | 0.003311311 | 0.019054607 | 5.88 | 0.002691535 | 0.009332543 | chr16 | 85121907 | 85122157 | NM_001286565.1 | 0.0027 | + | 85121907 | 85122157 | 250, |
| peak20544 | 2.67 | 0.004168694 | 0.022908677 | 23.3 | 3.71535E-06 | 2.23872E-05 | chr16 | 85706213 | 85706463 | NM_014615.5 | 0.0000037 | + | 85706213 | 85706463 | 250, |
| peak20548 | 1.82 | 0.001202264 | 0.009549926 | 17.3 | 0.002137962 | 0.007413102 | chr16 | 88500974 | 88501125 | NM_001367624.2 | 0.0021 | + | 88500974 | 88501125 | 151, |
| peak20561 | 4.84 | 4.36516E-05 | 0.000870964 | 2.02 | 0.000154882 | 0.000645654 | chr16 | 89616909 | 89617009 | NM_003119.4 | 0.00015 | + | 89616909 | 89617009 | 100, |
| peak20615 | 4.83 | 5.12861E-08 | 3.71535E-06 | 7.75 | 0.000001 | 3.89045E-05 | chr16 | 5135337 | 5135634 | NM_001289029.1 | 0.000001 | - | 5135337 | 5135634 | 297, |
| peak20617 | 4.67 | 3.31131E-08 | 2.51189E-06 | 7.75 | 1.90546E-05 | 0.00047863 | chr16 | 5135280 | 5135624 | NM_201598.3 | 0.000019 | - | 5135280 | 5135624 | 344, |
| peak20640 | 1.91 | 0.001071519 | 0.008709636 | 10.7 | 0.00030903 | 0.001230269 | chr16 | 17201507 | 17201906 | NM_022166.4 | 0.00031 | - | 17201507 | 17201906 | 399, |
| peak20647 | 1.67 | 2.75423E-05 | 0.000630957 | 26.1 | 3.38844E-10 | 7.76247E-09 | chr16 | 18820265 | 18820864 | NM_015092.4 | 3.4E-10 | - | 18820265 | 18820864 | 599, |
| peak20654 | 6.43 | 2.39883E-08 | 1.86209E-06 | 12.1 | 0.000831764 | 0.003090295 | chr16 | 19716599 | 19716849 | NM_001348529.2 | 0.00083 | - | 19716599 | 19716849 | 250, |
| peak20658 | 6.43 | 1.38038E-08 | 1.09648E-06 | 12.7 | 0.000977237 | 0.003548134 | chr16 | 19716598 | 19716848 | NM_001348531.2 | 0.00098 | - | 19716598 | 19716848 | 250, |
| peak20664 | 6.09 | 6.60693E-07 | 3.38844E-05 | 10.6 | 0.001862087 | 0.006456542 | chr16 | 19716556 | 19716806 | NM_001348535.2 | 0.0019 | - | 19716556 | 19716806 | 250, |
| peak20667 | 2.08 | 0.000954993 | 0.008128305 | 2.3 | 1.38038E-05 | 7.07946E-05 | chr16 | 19725972 | 19726272 | NM_001348535.2 | 0.000014 | - | 19725972 | 19726272 | 300, |
| peak20668 | 6.39 | 4.89779E-07 | 2.69153E-05 | 10.8 | 0.003467369 | 0.012022644 | chr16 | 19716639 | 19716789 | NM_001348536.2 | 0.0035 | - | 19716639 | 19716789 | 150, |
| peak20687 | 2.11 | 0.001698244 | 0.012022644 | 24.8 | 0.000295121 | 0.001174898 | chr16 | 23078875 | 23079026 | NM_020718.3 | 0.0003 | - | 23078875 | 23079026 | 151, |
| peak20688 | 2.45 | 0.000234423 | 0.002884032 | 9.64 | 1.44544E-08 | 1.86209E-07 | chr16 | 23079375 | 23079676 | NM_020718.3 | 0.000000014 | - | 23079375 | 23079676 | 301, |
| peak20716 | 7.07 | 7.4131E-06 | 0.000229087 | 31.7 | 5.88844E-07 | 4.46684E-06 | chr16 | 30594591 | 30596467 | NM_152458.7 | 0.00000059 | - | 30594591 | 30596467 | 173,25, |
| peak20795 | 2.58 | 0.000194984 | 0.002511886 | 3.44 | 2.18776E-07 | 1.07152E-05 | chr16 | 66544888 | 66545138 | NM_004614.5 | 0.00000022 | - | 66544888 | 66545138 | 250, |
| peak20797 | 2.7 | 0.004786301 | 0.025118864 | 3.67 | 0.000120226 | 0.002398833 | chr16 | 66545028 | 66545129 | NR_073520.2 | 0.00012 | - | 66545028 | 66545129 | 101, |
| peak20799 | 2.88 | 0.000812831 | 0.00724436 | 3.44 | 3.54813E-05 | 0.000831764 | chr16 | 66544890 | 66545140 | NM_001172643.1 | 0.000035 | - | 66544890 | 66545140 | 250, |
| peak20801 | 2.96 | 0.004786301 | 0.025118864 | 3.78 | 1.7378E-05 | 0.000446684 | chr16 | 66545026 | 66545126 | NM_001271935.1 | 0.000017 | - | 66545026 | 66545126 | 100, |
| peak20803 | 2.7 | 0.004897788 | 0.025703958 | 3.56 | 2.45471E-07 | 1.1749E-05 | chr16 | 66545030 | 66545130 | NM_001172645.1 | 0.00000025 | - | 66545030 | 66545130 | 100, |
| peak20829 | 1.16 | 0.000251189 | 0.003019952 | 18.9 | 3.0903E-07 | 2.5704E-06 | chr16 | 69152104 | 69152403 | NM_001366605.1 | 0.00000031 | - | 69152104 | 69152403 | 299, |
| peak20831 | 1.4 | 0.000123027 | 0.001819701 | 16.2 | 2.69153E-07 | 2.23872E-06 | chr16 | 69152109 | 69152406 | NM_001366604.1 | 0.00000027 | - | 69152109 | 69152406 | 297, |
| peak20833 | 1.28 | 8.91251E-05 | 0.00144544 | 18.2 | 6.30957E-22 | 1.58489E-19 | chr16 | 69152108 | 69152404 | NM_001002847.3 | 6.3E-22 | - | 69152108 | 69152404 | 296, |
| peak20835 | 1.38 | 0.000562341 | 0.005370318 | 16.9 | 1.25893E-15 | 1.58489E-13 | chr16 | 69152108 | 69152406 | NM_001040144.2 | 1.3E-15 | - | 69152108 | 69152406 | 298, |
| peak20837 | 1.35 | 0.000123027 | 0.001778279 | 16.2 | 4.67735E-05 | 0.000213796 | chr16 | 69152110 | 69152408 | NM_001366602.1 | 0.000047 | - | 69152110 | 69152408 | 298, |
| peak20839 | 1.3 | 0.000223872 | 0.002754229 | 16.7 | 6.30957E-20 | 1.58489E-17 | chr16 | 69152111 | 69152411 | NM_001366603.1 | 6.3E-20 | - | 69152111 | 69152411 | 300, |
| peak20841 | 1.28 | 0.000162181 | 0.002187762 | 18.4 | 1.99526E-14 | 1.58489E-12 | chr16 | 69152107 | 69152401 | NM_001366606.1 | 2E-14 | - | 69152107 | 69152401 | 294, |
| peak20843 | 1.3 | 0.000223872 | 0.002754229 | 16.7 | 1.28825E-10 | 3.38844E-09 | chr16 | 69152111 | 69152411 | NM_001040146.5 | 1.3E-10 | - | 69152111 | 69152411 | 300, |

| | | | | | | | | | | | | | | | |
|---|---|---|---|---|---|---|---|---|---|---|---|---|---|---|---|
| peak20845 | 1.29 | 9.33254E-05 | 0.001479108 | 16.2 | 1.99526E-18 | 3.98107E-16 | chr16 | 69152110 | 69152408 | NM_001039690.5 | 2E-18 | - | 69152110 | 69152408 | 298, |
| peak20862 | 5.31 | 0.00025704 | 0.003019952 | 6.75 | 4.36516E-05 | 0.000204174 | chr16 | 71319174 | 71319373 | NM_001099642.2 | 0.000044 | - | 71319174 | 71319373 | 199, |
| peak20864 | 4.92 | 0.001348963 | 0.01023293 | 5 | 9.54993E-05 | 0.00040738 | chr16 | 71319159 | 71319358 | NM_001324374.2 | 0.000095 | - | 71319159 | 71319358 | 199, |
| peak20867 | 4.93 | 0.000436516 | 0.004570882 | 6.47 | 2.45471E-06 | 1.58489E-05 | chr16 | 71319165 | 71319365 | NM_001324378.2 | 0.0000025 | - | 71319165 | 71319365 | 200, |
| peak20869 | 5.19 | 0.000288403 | 0.003311311 | 5.17 | 7.07946E-05 | 0.000316228 | chr16 | 71319165 | 71319365 | NM_001324379.2 | 0.000071 | - | 71319165 | 71319365 | 200, |
| peak20870 | 5.3 | 0.00040738 | 0.004365158 | 6.5 | 3.54813E-05 | 0.000169824 | chr16 | 71319173 | 71319373 | NM_018348.6 | 0.000035 | - | 71319173 | 71319373 | 200, |
| peak20872 | 5.03 | 0.000234423 | 0.002818383 | 4.14 | 2.69153E-05 | 0.000131826 | chr16 | 71319188 | 71319389 | NM_001324377.2 | 0.000027 | - | 71319188 | 71319389 | 201, |
| peak20890 | 4.45 | 4.46684E-05 | 0.000891251 | 1.42 | 0.013182567 | 0.042657952 | chr16 | 72830120 | 72830221 | NM_006885.4 | 0.013 | - | 72830120 | 72830221 | 101, |
| peak20894 | 4.59 | 0.002951209 | 0.017782794 | 2.94 | 0.00074131 | 0.002754229 | chr16 | 72991575 | 72991676 | NM_006885.4 | 0.00074 | - | 72991575 | 72991676 | 101, |
| peak20900 | 3.13 | 0.000234423 | 0.002884032 | 3 | 0.002511886 | 0.008709636 | chr16 | 72821370 | 72821571 | NM_001164766.2 | 0.0025 | - | 72821370 | 72821571 | 201, |
| peak20905 | 1.71 | 0.000457088 | 0.004677351 | 4.38 | 0.000275423 | 0.005011872 | chr16 | 72828219 | 72828470 | NM_001164766.2 | 0.00028 | - | 72828219 | 72828470 | 251, |
| peak20908 | 4.61 | 0.000131826 | 0.001905461 | 2.14 | 0.000275423 | 0.001096478 | chr16 | 72830114 | 72830215 | NM_001164766.2 | 0.00028 | - | 72830114 | 72830215 | 101, |
| peak20940 | 1.35 | 0.003715352 | 0.020892961 | 8.22 | 5.7544E-08 | 6.0256E-07 | chr16 | 74502948 | 74511327 | NR_027265.2 | 0.000000058 | - | 74502948 | 74511327 | 13,89,114,63,87,34, |
| peak20942 | 6.21 | 2.29087E-07 | 1.38038E-05 | 6.88 | 0.000588844 | 0.002238721 | chr16 | 74694935 | 74695086 | NM_001370534.1 | 0.00059 | - | 74694935 | 74695086 | 151, |
| peak20943 | 5.9 | 3.38844E-08 | 2.51189E-06 | 5.07 | 9.12011E-07 | 6.60693E-06 | chr16 | 74694943 | 74695143 | NM_001370536.1 | 0.00000091 | - | 74694943 | 74695143 | 200, |
| peak20944 | 6.13 | 5.12861E-08 | 3.71535E-06 | 6.43 | 6.30957E-07 | 4.7863E-06 | chr16 | 74694941 | 74695141 | NM_001370539.1 | 0.00000063 | - | 74694941 | 74695141 | 200, |
| peak20974 | 1.96 | 2.13796E-06 | 8.70964E-05 | 10.4 | 0.012589254 | 0.040738028 | chr16 | 75646301 | 75646600 | NM_001324451.2 | 0.013 | - | 75646301 | 75646600 | 299, |
| peak20978 | 1.54 | 0.001698244 | 0.012022644 | 22 | 1.04713E-05 | 5.62341E-05 | chr16 | 80718676 | 80718877 | NM_152342.4 | 0.00001 | - | 80718676 | 80718877 | 201, |
| peak20989 | 6.61 | 5.12861E-07 | 2.81838E-05 | 9.29 | 3.89045E-05 | 0.00018197 | chr16 | 82033106 | 82033407 | NM_145168.3 | 0.000039 | - | 82033106 | 82033407 | 301, |
| peak21007 | 4.4 | 2.51189E-11 | 2.75423E-09 | 11.6 | 4.67735E-05 | 0.000213796 | chr16 | 86565528 | 86565975 | NM_001159377.2 | 0.000047 | - | 86565528 | 86565975 | 447, |
| peak21010 | 4.4 | 2.51189E-11 | 2.75423E-09 | 11.6 | 5.12861E-05 | 0.000234423 | chr16 | 86565528 | 86565975 | NM_001159378.2 | 0.000051 | - | 86565528 | 86565975 | 447, |
| peak21012 | 2.57 | 7.07946E-05 | 0.001230269 | 11.6 | 0.002754229 | 0.009549926 | chr16 | 86565526 | 86565775 | NM_001159379.2 | 0.0028 | - | 86565526 | 86565775 | 249, |
| peak21014 | 2.57 | 7.07946E-05 | 0.001230269 | 11.6 | 0.001737801 | 0.00616595 | chr16 | 86565526 | 86565774 | NM_001159380.2 | 0.0017 | - | 86565526 | 86565774 | 248, |
| peak21018 | 2.1 | 9.12011E-05 | 0.001479108 | 10.9 | 2.5704E-05 | 0.000125893 | chr16 | 86565539 | 86565840 | NR_027489.2 | 0.000026 | - | 86565539 | 86565840 | 301, |
| peak21145 | 2 | 2.75423E-06 | 0.000104713 | 1.24 | 1.14815E-05 | 6.0256E-05 | chr17 | 16946169 | 16946270 | NM_001364716.2 | 0.000011 | + | 16946169 | 16946270 | 101, |
| peak21152 | 2.15 | 3.16228E-06 | 0.00011749 | 1.27 | 3.31131E-06 | 2.04174E-05 | chr17 | 16946168 | 16946269 | NM_015134.4 | 0.0000033 | + | 16946168 | 16946269 | 101, |
| peak21191 | 2.27 | 0.001071519 | 0.008709636 | 7.45 | 0.001995262 | 0.007079458 | chr17 | 27861271 | 27869632 | NM_025142.1 | 0.002 | + | 27861271 | 27869632 | 47,54, |
| peak21199 | 1.72 | 0.001230269 | 0.009549926 | 7.5 | 5.01187E-09 | 7.58578E-08 | chr17 | 28817200 | 28849545 | NM_001007024.2 | 0.000000005 | + | 28817200 | 28849545 | 35,75,30,83,274, |
| peak21202 | 1.74 | 0.001905461 | 0.013182567 | 5.29 | 5.01187E-09 | 4.46684E-07 | chr17 | 28851133 | 28851234 | NM_001007024.2 | 0.000000005 | + | 28851133 | 28851234 | 101, |
| peak21203 | 1.77 | 0.004677351 | 0.024547089 | 8.8 | 3.54813E-07 | 2.88403E-06 | chr17 | 28817218 | 28849567 | NM_004871.3 | 0.00000035 | + | 28817218 | 28849567 | 17,75,30,83,296, |
| peak21213 | 1.99 | 3.0903E-07 | 1.8197E-05 | 4.38 | 4.2658E-05 | 0.000199526 | chr17 | 29652915 | 29654761 | NM_000267.3 | 0.000043 | + | 29652915 | 29654761 | 355,245, |
| peak21217 | 2.05 | 7.4131E-07 | 3.80189E-05 | 4.4 | 0.000138038 | 0.000588844 | chr17 | 29653057 | 29654753 | NM_001042492.3 | 0.00014 | + | 29653057 | 29654753 | 213,237, |
| peak21225 | 3.48 | 0.003090295 | 0.018620871 | 12.4 | 4.89779E-05 | 0.000223872 | chr17 | 33585747 | 33586147 | NM_001330183.2 | 0.000049 | + | 33585747 | 33586147 | 400, |
| peak21226 | 3.49 | 0.001737801 | 0.012302688 | 9.67 | 4.89779E-05 | 0.000223872 | chr17 | 33586246 | 33586646 | NM_001330183.2 | 0.000049 | + | 33586246 | 33586646 | 400, |
| peak21231 | 3.5 | 0.001995262 | 0.013803843 | 12.4 | 0.000131826 | 0.000549541 | chr17 | 33585746 | 33586146 | NM_144975.4 | 0.00013 | + | 33585746 | 33586146 | 400, |
| peak21232 | 3.66 | 0.001479108 | 0.010964782 | 10.7 | 0.000131826 | 0.000549541 | chr17 | 33586244 | 33586643 | NM_144975.4 | 0.00013 | + | 33586244 | 33586643 | 399, |
| peak21283 | 1.49 | 9.77237E-05 | 0.001513561 | 4.83 | 3.89045E-05 | 0.00018197 | chr17 | 41352432 | 41355701 | NM_031862.4 | 0.000039 | + | 41352432 | 41355701 | 193,95,58,4, |
| peak21291 | 1.46 | 1.99526E-06 | 8.12831E-05 | 6.08 | 1.07152E-05 | 5.7544E-05 | chr17 | 41352414 | 41362013 | NM_001291572.2 | 0.000011 | + | 41352414 | 41362013 | 211,95,94, |
| peak21312 | 1.11 | 0.002951209 | 0.017782794 | 17.5 | 2.04174E-08 | 2.51189E-07 | chr17 | 41569592 | 41570180 | NM_001302623.2 | 0.00000002 | + | 41569592 | 41570180 | 68,132, |
| peak21314 | 1.25 | 0.004168694 | 0.022908677 | 18 | 1.07152E-08 | 1.44544E-07 | chr17 | 41569589 | 41570176 | NM_001322220.2 | 0.000000011 | + | 41569589 | 41570176 | 71,128, |
| peak21317 | 1.11 | 0.002951209 | 0.017782794 | 17.5 | 1.8197E-08 | 2.23872E-07 | chr17 | 41569592 | 41570180 | NM_001322219.2 | 0.000000018 | + | 41569592 | 41570180 | 68,132, |
| peak21343 | 3.3 | 1.58489E-05 | 0.000416869 | 6.22 | 2.75423E-06 | 1.7378E-05 | chr17 | 42635100 | 42635299 | NM_001466.4 | 0.0000028 | + | 42635100 | 42635299 | 199, |
| peak21357 | 2.52 | 0.000275423 | 0.003235937 | 1.37 | 1.44544E-06 | 0.00001 | chr17 | 45000575 | 45009494 | NM_054022.4 | 0.0000014 | + | 45000575 | 45009494 | 12,65,109,62, |
| peak21359 | 2.41 | 0.000363078 | 0.003890451 | 1.32 | 2.18776E-08 | 2.69153E-07 | chr17 | 45000575 | 45009495 | NR_148349.2 | 0.000000022 | + | 45000575 | 45009495 | 12,65,109,63, |
| peak21360 | 2.85 | 0.001 | 0.008317638 | 1.32 | 1.23027E-08 | 1.62181E-07 | chr17 | 45008487 | 45009496 | NR_148350.2 | 0.000000012 | + | 45008487 | 45009496 | 86,64, |
| peak21361 | 2.4 | 0.000398107 | 0.004265795 | 1.32 | 4.67735E-08 | 5.12861E-07 | chr17 | 45000575 | 45009495 | NR_148351.2 | 0.000000047 | + | 45000575 | 45009495 | 12,65,109,63, |
| peak21362 | 1.13 | 5.88844E-05 | 0.001071519 | 16.2 | 8.31764E-07 | 6.16595E-06 | chr17 | 45759842 | 45760091 | NM_002265.6 | 0.00000083 | + | 45759842 | 45760091 | 249, |
| peak21382 | 3.18 | 0.0001 | 0.001548817 | 2.14 | 4.0738E-06 | 2.45471E-05 | chr17 | 47904108 | 47904860 | NM_001199155.2 | 0.0000041 | + | 47904108 | 47904860 | 53,98, |
| peak21388 | 3.18 | 0.0001 | 0.001548817 | 2.14 | 4.7863E-06 | 2.81838E-05 | chr17 | 47904108 | 47904860 | NM_001346706.2 | 0.0000048 | + | 47904108 | 47904860 | 53,98, |
| peak21477 | 1.13 | 0.00128825 | 0.01 | 14.8 | 5.7544E-06 | 3.31131E-05 | chr17 | 71232934 | 71233083 | NM_001288770.3 | 0.0000058 | + | 71232934 | 71233083 | 149, |
| peak21499 | 4.72 | 0.000338844 | 0.003801894 | 4 | 7.4131E-07 | 3.0903E-05 | chr17 | 76045390 | 76045491 | NM_001142640.1 | 0.00000074 | + | 76045390 | 76045491 | 101, |
| peak21505 | 4.72 | 0.000338844 | 0.003801894 | 4 | 8.31764E-07 | 3.38844E-05 | chr17 | 76045390 | 76045490 | NM_018996.3 | 0.00000083 | + | 76045390 | 76045490 | 100, |
| peak21542 | 3.54 | 7.24436E-07 | 3.71535E-05 | 3.2 | 0.003235937 | 0.047863009 | chr17 | 648159 | 648360 | NM_015721.3 | 0.0032 | - | 648159 | 648360 | 201, |
| peak21544 | 2.2 | 0.004897788 | 0.025703958 | 5.4 | 1.12202E-05 | 0.00030903 | chr17 | 649856 | 650057 | NM_015721.3 | 0.000011 | - | 649856 | 650057 | 201, |
| peak21557 | 3.59 | 0.000630957 | 0.005888437 | 7.24 | 8.91251E-05 | 0.000389045 | chr17 | 962023 | 970382 | NM_001282149.2 | 0.000089 | - | 962023 | 970382 | 84,66, |
| peak21562 | 3.51 | 0.000758578 | 0.00676083 | 7.28 | 0.00144544 | 0.005248075 | chr17 | 962020 | 970379 | NM_001322840.2 | 0.0014 | - | 962020 | 970379 | 87,63, |
| peak21567 | 3.57 | 4.67735E-06 | 0.000162181 | 10.8 | 5.24807E-05 | 0.000239883 | chr17 | 961994 | 970403 | NM_021962.5 | 0.000052 | - | 961994 | 970403 | 113,87, |

| | | | | | | | | | | | | | | | |
|---|---|---|---|---|---|---|---|---|---|---|---|---|---|---|---|
| peak21572 | 3.45 | 0.000645654 | 0.006025596 | 7.24 | 0.000295121 | 0.001174898 | chr17 | 962015 | 970373 | NM_001159746.3 | 0.0003 | - | 962015 | 970373 | 92,57, |
| peak21577 | 3.51 | 0.000758578 | 0.00676083 | 7.28 | 0.00040738 | 0.001548817 | chr17 | 962022 | 970381 | NM_001322841.2 | 0.00041 | - | 962022 | 970381 | 85,65, |
| peak21602 | 1.84 | 0.000660693 | 0.00616595 | 1.53 | 8.31764E-05 | 0.000363078 | chr17 | 2601294 | 2601445 | NM_001366662.1 | 0.000083 | - | 2601294 | 2601445 | 151, |
| peak21610 | 4.55 | 3.80189E-06 | 0.000134896 | 5.16 | 3.31131E-05 | 0.000158489 | chr17 | 3910081 | 3910231 | NM_015113.4 | 0.000033 | - | 3910081 | 3910231 | 150, |
| peak21611 | 1.9 | 2.34423E-05 | 0.000562341 | 9.13 | 2.5704E-06 | 1.65959E-05 | chr17 | 3920923 | 3921228 | NM_015113.4 | 0.0000026 | - | 3920923 | 3921228 | 101,99, |
| peak21620 | 1.21 | 0.000707946 | 0.006456542 | 8.44 | 4.36516E-06 | 2.63027E-05 | chr17 | 4175580 | 4186109 | NM_003342.4 | 0.0000044 | - | 4175580 | 4186109 | 231,17, |
| peak21623 | 3.28 | 1.41254E-05 | 0.000380189 | 8.57 | 0.00001 | 5.37032E-05 | chr17 | 4575900 | 4576200 | NM_001278241.2 | 0.00001 | - | 4575900 | 4576200 | 300, |
| peak21627 | 3.58 | 0.000724436 | 0.006606934 | 10.8 | 7.76247E-07 | 5.7544E-06 | chr17 | 4575918 | 4576168 | NM_014389.3 | 0.00000078 | - | 4575918 | 4576168 | 250, |
| peak21639 | 2.02 | 3.98107E-05 | 0.000812831 | 10.4 | 0.003630781 | 0.012302688 | chr17 | 7138888 | 7139135 | NM_001284518.1 | 0.0036 | - | 7138888 | 7139135 | 247, |
| peak21663 | 3.16 | 0.001380384 | 0.010471285 | 3.14 | 1.44544E-06 | 5.37032E-05 | chr17 | 8445488 | 8448805 | NM_001256012.2 | 0.0000014 | - | 8445488 | 8448805 | 110,40, |
| peak21671 | 2.43 | 0.000114815 | 0.001698244 | 18.8 | 5.88844E-08 | 6.30957E-07 | chr17 | 10584119 | 10590092 | NM_004589.4 | 0.000000059 | - | 10584119 | 10590092 | 451,49, |
| peak21672 | 2.62 | 0.000489779 | 0.004897788 | 5.75 | 0.001 | 0.003630781 | chr17 | 12895767 | 12895916 | NM_173717.1 | 0.001 | - | 12895767 | 12895916 | 149, |
| peak21683 | 3.4 | 7.07946E-06 | 0.000223872 | 5 | 1.38038E-05 | 7.24436E-05 | chr17 | 16001705 | 16004808 | NM_006311.4 | 0.000014 | - | 16001705 | 16004808 | 105,245, |
| peak21689 | 3.2 | 7.4131E-05 | 0.001258925 | 3.16 | 2.75423E-05 | 0.000134896 | chr17 | 15995364 | 16004825 | NM_001190440.1 | 0.000028 | - | 15995364 | 16004825 | 8,130,262, |
| peak21693 | 3.29 | 9.77237E-05 | 0.001513561 | 5.5 | 0.000645654 | 0.002454709 | chr17 | 16001691 | 16004845 | NM_001190438.1 | 0.00065 | - | 16001691 | 16004845 | 119,282, |
| peak21708 | 4.09 | 1.77828E-06 | 7.58578E-05 | 21.5 | 1.69824E-08 | 2.13796E-07 | chr17 | 17749873 | 17750074 | NM_001288787.2 | 0.000000017 | - | 17749873 | 17750074 | 201, |
| peak21711 | 4.14 | 1.12202E-05 | 0.000316228 | 11.8 | 4.2658E-05 | 0.000199526 | chr17 | 17749863 | 17750063 | NM_001288788.2 | 0.000043 | - | 17749863 | 17750063 | 200, |
| peak21719 | 4.12 | 1.58489E-06 | 6.91831E-05 | 10.9 | 0.001513561 | 0.005370318 | chr17 | 17749859 | 17750009 | NM_001350332.2 | 0.0015 | - | 17749859 | 17750009 | 150, |
| peak21732 | 1.4 | 0.000457088 | 0.004677351 | 22.7 | 0.000275423 | 0.001096478 | chr17 | 19850803 | 19861658 | NM_007202.4 | 0.00028 | - | 19850803 | 19861658 | 15,332, |
| peak21745 | 1.26 | 0.001174898 | 0.009332543 | 24.7 | 5.88844E-06 | 3.38844E-05 | chr17 | 26975712 | 26976150 | NR_045585.1 | 0.0000059 | - | 26975712 | 26976150 | 438, |
| peak21746 | 5.3 | 1.8197E-06 | 7.58578E-05 | 19.2 | 0.000416869 | 0.00162181 | chr17 | 27051464 | 27051614 | NM_138463.4 | 0.00042 | - | 27051464 | 27051614 | 150, |
| peak21794 | 5.92 | 1.65959E-06 | 7.07946E-05 | 4.18 | 7.07946E-08 | 7.24436E-07 | chr17 | 27401329 | 27401580 | NM_001346766.2 | 0.000000071 | - | 27401329 | 27401580 | 251, |
| peak21866 | 1.72 | 0.000524807 | 0.005128614 | 2.35 | 2.81838E-05 | 0.000138038 | chr17 | 37420509 | 37421669 | NM_001184906.2 | 0.000028 | - | 37420509 | 37421669 | 131,20, |
| peak21867 | 1.63 | 0.000323594 | 0.003630781 | 2.5 | 1.99526E-05 | 0.0001 | chr17 | 37420505 | 37421665 | NM_032875.3 | 0.00002 | - | 37420505 | 37421665 | 135,16, |
| peak21868 | 1.37 | 0.004073803 | 0.022387211 | 2.31 | 3.54813E-05 | 0.000165959 | chr17 | 37420521 | 37420622 | NM_001370208.1 | 0.000035 | - | 37420521 | 37420622 | 101, |
| peak21869 | 1.45 | 0.004073803 | 0.022387211 | 2.39 | 2.69153E-05 | 0.000131826 | chr17 | 37420531 | 37420632 | NM_001370209.1 | 0.000027 | - | 37420531 | 37420632 | 101, |
| peak21870 | 2.95 | 0.000389045 | 0.004168694 | 37.7 | 0.000295121 | 0.001174898 | chr17 | 37915764 | 37915915 | NM_001284516.1 | 0.0003 | - | 37915764 | 37915915 | 151, |
| peak21871 | 2.95 | 0.000371535 | 0.003981072 | 37.7 | 0.000169824 | 0.000707946 | chr17 | 37915766 | 37915916 | NM_001284514.1 | 0.00017 | - | 37915766 | 37915916 | 150, |
| peak21872 | 3.06 | 8.51138E-07 | 4.2658E-05 | 37.7 | 2.23872E-06 | 1.44544E-05 | chr17 | 37915767 | 37915968 | NM_001257408.2 | 0.0000022 | - | 37915767 | 37915968 | 201, |
| peak21873 | 2.97 | 0.000371535 | 0.003981072 | 37.7 | 0.000234423 | 0.000933254 | chr17 | 37915764 | 37915914 | NM_001257409.2 | 0.00023 | - | 37915764 | 37915914 | 150, |
| peak21874 | 2.95 | 0.000371535 | 0.003981072 | 37.7 | 0.000338844 | 0.001348963 | chr17 | 37915765 | 37915916 | NM_001257410.2 | 0.00034 | - | 37915765 | 37915916 | 151, |
| peak21875 | 3 | 0.000331131 | 0.003715352 | 36 | 0.000630957 | 0.002344229 | chr17 | 37915762 | 37915912 | NM_001257411.2 | 0.00063 | - | 37915762 | 37915912 | 150, |
| peak21876 | 3.01 | 0.000316228 | 0.003630781 | 37.7 | 6.91831E-05 | 0.00030903 | chr17 | 37915768 | 37915919 | NM_001257412.2 | 0.000069 | - | 37915768 | 37915919 | 151, |
| peak21877 | 2.97 | 0.000346737 | 0.003801894 | 37.7 | 0.000245471 | 0.001 | chr17 | 37915765 | 37915915 | NM_001257413.2 | 0.00025 | - | 37915765 | 37915915 | 150, |
| peak21878 | 3.06 | 8.51138E-07 | 4.2658E-05 | 37.7 | 2.18776E-06 | 1.44544E-05 | chr17 | 37915767 | 37915968 | NM_001257414.2 | 0.0000022 | - | 37915767 | 37915968 | 201, |
| peak21879 | 3.08 | 3.0903E-07 | 1.77828E-05 | 37.7 | 4.7863E-08 | 5.12861E-07 | chr17 | 37915769 | 37915970 | NM_001284515.2 | 0.000000048 | - | 37915769 | 37915970 | 201, |
| peak21880 | 3.06 | 8.51138E-07 | 4.2658E-05 | 37.7 | 1.25893E-06 | 8.70964E-06 | chr17 | 37915768 | 37915968 | NM_012481.5 | 0.0000013 | - | 37915768 | 37915968 | 200, |
| peak21881 | 2.95 | 0.000389045 | 0.004168694 | 37.7 | 0.00030903 | 0.001202264 | chr17 | 37915764 | 37915915 | NM_183228.3 | 0.00031 | - | 37915764 | 37915915 | 151, |
| peak21882 | 2.95 | 0.000389045 | 0.004168694 | 37.7 | 0.000112202 | 0.00047863 | chr17 | 37915764 | 37915915 | NM_183229.3 | 0.00011 | - | 37915764 | 37915915 | 151, |
| peak21883 | 2.95 | 0.000389045 | 0.004168694 | 37.7 | 0.000147911 | 0.000616595 | chr17 | 37915764 | 37915915 | NM_183230.3 | 0.00015 | - | 37915764 | 37915915 | 151, |
| peak21884 | 3 | 0.000331131 | 0.003715352 | 36 | 0.000371535 | 0.001412538 | chr17 | 37915761 | 37915911 | NM_183231.3 | 0.00037 | - | 37915761 | 37915911 | 150, |
| peak21885 | 3 | 0.000331131 | 0.003715352 | 36 | 0.000512861 | 0.001949845 | chr17 | 37915761 | 37915911 | NM_183232.3 | 0.00051 | - | 37915761 | 37915911 | 150, |
| peak21916 | 1.36 | 1.09648E-05 | 0.00030903 | 7.45 | 2.81838E-08 | 3.31131E-07 | chr17 | 42289060 | 42293046 | NM_001076684.3 | 0.000000028 | - | 42289060 | 42293046 | 55,134,121,65,25, |
| peak21921 | 3.49 | 0.001737801 | 0.012302688 | 1.56 | 6.30957E-32 | 3.98107E-29 | chr17 | 42288458 | 42288696 | NM_001076683.2 | 6.3E-32 | - | 42288458 | 42288696 | 62,39, |
| peak21922 | 1.01 | 0.000229087 | 0.002818383 | 7.03 | 6.30957E-32 | 3.98107E-29 | chr17 | 42289069 | 42293056 | NM_001076683.2 | 6.3E-32 | - | 42289069 | 42293056 | 46,134,121,65,35, |
| peak21930 | 2.85 | 0.000691831 | 0.006456542 | 4.17 | 1.7378E-06 | 1.14815E-05 | chr17 | 42478462 | 42478662 | NM_001304943.1 | 0.0000017 | - | 42478462 | 42478662 | 200, |
| peak21941 | 2.16 | 0.003019952 | 0.018197009 | 4.33 | 5.62341E-07 | 4.36516E-06 | chr17 | 42478460 | 42478660 | NM_001304939.2 | 0.00000056 | - | 42478460 | 42478660 | 200, |
| peak21952 | 2.18 | 0.003311311 | 0.019054607 | 4.29 | 5.49541E-07 | 4.16869E-06 | chr17 | 42478447 | 42478648 | NM_001304941.2 | 0.00000055 | - | 42478447 | 42478648 | 201, |
| peak21957 | 2.85 | 0.000691831 | 0.006456542 | 4.17 | 2.5704E-06 | 1.65959E-05 | chr17 | 42478462 | 42478662 | NM_001304942.2 | 0.0000026 | - | 42478462 | 42478662 | 200, |
| peak21973 | 1.86 | 0.001995262 | 0.013489629 | 4.36 | 0.000346737 | 0.001348963 | chr17 | 46148164 | 46148313 | NM_001127228.2 | 0.00035 | - | 46148164 | 46148313 | 149, |
| peak21996 | 2.74 | 0.004168694 | 0.022908677 | 1.8 | 3.31131E-05 | 0.000158489 | chr17 | 48777201 | 48777975 | NM_052855.4 | 0.000033 | - | 48777201 | 48777975 | 53,47, |
| peak22012 | 2.44 | 0.003090295 | 0.018620871 | 2.41 | 0.005011872 | 0.016982437 | chr17 | 54991109 | 54991210 | NM_005082.5 | 0.005 | - | 54991109 | 54991210 | 101, |
| peak22014 | 1.5 | 0.001071519 | 0.008709636 | 8.14 | 1.28825E-09 | 2.39883E-08 | chr17 | 55028036 | 55028186 | NM_004645.3 | 1.3E-09 | - | 55028036 | 55028186 | 150, |
| peak22019 | 1.53 | 0.004466836 | 0.023988329 | 11.8 | 0.000676083 | 0.002511886 | chr17 | 56386177 | 56386477 | NM_001261835.2 | 0.00068 | - | 56386177 | 56386477 | 300, |
| peak22030 | 3.22 | 0.002089296 | 0.014125375 | 4.75 | 0.001202264 | 0.004365158 | chr17 | 56572421 | 56572621 | NM_004687.5 | 0.0012 | - | 56572421 | 56572621 | 200, |
| peak22036 | 2.88 | 0.000177828 | 0.002344229 | 6.46 | 1.07152E-05 | 5.7544E-05 | chr17 | 58121248 | 58121398 | NM_022070.5 | 0.000011 | - | 58121248 | 58121398 | 150, |
| peak22057 | 2.7 | 0.003162278 | 0.019054607 | 2.89 | 0.000104713 | 0.000457088 | chr17 | 65105711 | 65110499 | NM_001330447.2 | 0.0001 | - | 65105711 | 65110499 | 91,60, |

| | | | | | | | | | | | | | | | |
|---|---|---|---|---|---|---|---|---|---|---|---|---|---|---|---|
| peak22061 | 2.57 | 0.002951209 | 0.018197009 | 2.89 | 0.000104713 | 0.000446684 | chr17 | 65105709 | 65110497 | NM_014877.4 | 0.0001 | - | 65105709 | 65110497 | 93,58, |
| peak22066 | 2.02 | 3.89045E-06 | 0.000138038 | 14.4 | 4.46684E-06 | 2.63027E-05 | chr17 | 71203740 | 71204537 | NM_001098832.1 | 0.0000045 | - | 71203740 | 71204537 | 797, |
| peak22068 | 2.27 | 1.14815E-05 | 0.000323594 | 7.8 | 0.000630957 | 0.002754229 | chr17 | 71203788 | 71204037 | NM_001289410.1 | 0.00063 | - | 71203788 | 71204037 | 249, |
| peak22075 | 3.38 | 1.65959E-05 | 0.000436516 | 5.56 | 4.2658E-05 | 0.000199526 | chr17 | 73269309 | 73269658 | NM_021734.4 | 0.000043 | - | 73269309 | 73269658 | 349, |
| peak22107 | 1.45 | 0.004897788 | 0.025118864 | 16.8 | 0.000104713 | 0.000446684 | chr17 | 74077146 | 74077495 | NM_001013839.4 | 0.0001 | - | 74077146 | 74077495 | 349, |
| peak22130 | 3.65 | 5.37032E-09 | 4.57088E-07 | 10.9 | 0.000380189 | 0.001479108 | chr17 | 76803087 | 76803685 | NM_001321291.1 | 0.00038 | - | 76803087 | 76803685 | 598, |
| peak22135 | 2.19 | 0.00025704 | 0.003019952 | 40 | 7.76247E-07 | 5.7544E-06 | chr17 | 76989336 | 76989585 | NM_001159772.2 | 0.00000078 | - | 76989336 | 76989585 | 249, |
| peak22136 | 2.28 | 0.000831764 | 0.00724436 | 39.1 | 2.45471E-07 | 2.0893E-06 | chr17 | 76989340 | 76989541 | NM_001159773.2 | 0.00000025 | - | 76989340 | 76989541 | 201, |
| peak22138 | 2.18 | 0.000263027 | 0.003090295 | 49.6 | 1.09648E-07 | 1.07152E-06 | chr17 | 76989339 | 76989588 | NM_138793.4 | 0.00000011 | - | 76989339 | 76989588 | 249, |
| peak22140 | 4.35 | 0.000251189 | 0.002951209 | 6.78 | 3.98107E-05 | 0.000186209 | chr17 | 77768496 | 77768695 | NM_020649.3 | 0.00004 | - | 77768496 | 77768695 | 199, |
| peak22147 | 4.41 | 0.000524807 | 0.005248075 | 4.07 | 0.015135612 | 0.047863009 | chr17 | 77909981 | 77910381 | NM_001271845.2 | 0.015 | - | 77909981 | 77910381 | 400, |
| peak22163 | 1.83 | 4.67735E-06 | 0.000158489 | 10.4 | 0.004265795 | 0.014454398 | chr17 | 79525910 | 79526360 | NM_001369698.1 | 0.0043 | - | 79525910 | 79526360 | 450, |
| peak22202 | 2.33 | 0.000363078 | 0.003981072 | 4.45 | 0.00018197 | 0.00074131 | chr18 | 8825464 | 8825814 | NM_015210.4 | 0.00018 | + | 8825464 | 8825814 | 350, |
| peak22209 | 1.17 | 0.004570882 | 0.024547089 | 9.43 | 7.58578E-07 | 5.62341E-06 | chr18 | 9258232 | 9258880 | NM_001083625.3 | 0.00000076 | + | 9258232 | 9258880 | 648, |
| peak22216 | 1.31 | 0.002238721 | 0.014791084 | 7 | 6.16595E-05 | 0.000275423 | chr18 | 9258221 | 9258871 | NM_015208.5 | 0.000062 | + | 9258221 | 9258871 | 650, |
| peak22217 | 2.96 | 0.004466836 | 0.023988329 | 5 | 0.000776247 | 0.002818383 | chr18 | 9283186 | 9283287 | NM_015208.5 | 0.00078 | + | 9283186 | 9283287 | 101, |
| peak22224 | 1.42 | 0.000138038 | 0.001949845 | 6.58 | 0.000158489 | 0.000645654 | chr18 | 9258317 | 9258868 | NM_001204056.1 | 0.00016 | + | 9258317 | 9258868 | 551, |
| peak22225 | 2.96 | 0.004466836 | 0.023988329 | 5.22 | 0.001479108 | 0.005248075 | chr18 | 9283184 | 9283285 | NM_001204056.1 | 0.0015 | + | 9283184 | 9283285 | 101, |
| peak22230 | 1.34 | 7.76247E-05 | 0.00128825 | 4.08 | 0.001548817 | 0.005495409 | chr18 | 9536818 | 9537117 | NM_006788.4 | 0.0015 | + | 9536818 | 9537117 | 299, |
| peak22322 | 2.81 | 6.91831E-05 | 0.001202264 | 9.67 | 4.2658E-09 | 6.60693E-08 | chr18 | 56586147 | 56586448 | NM_001318726.2 | 4.3E-09 | + | 56586147 | 56586448 | 301, |
| peak22326 | 2.93 | 7.24436E-05 | 0.001258925 | 9.67 | 3.16228E-14 | 2.51189E-12 | chr18 | 56586143 | 56586442 | NM_001353533.2 | 3.2E-14 | + | 56586143 | 56586442 | 299, |
| peak22330 | 2.53 | 1.86209E-05 | 0.000467735 | 14.2 | 2.04174E-10 | 5.01187E-09 | chr18 | 56586112 | 56586462 | NM_018181.6 | 2E-10 | + | 56586112 | 56586462 | 350, |
| peak22334 | 2.55 | 1.86209E-05 | 0.000467735 | 14.2 | 3.71535E-10 | 8.31764E-09 | chr18 | 56586113 | 56586462 | NR_148459.2 | 3.7E-10 | + | 56586113 | 56586462 | 349, |
| peak22339 | 2.99 | 9.54993E-05 | 0.001479108 | 17.5 | 9.54993E-10 | 1.86209E-08 | chr18 | 56586130 | 56586429 | NM_001318727.2 | 9.5E-10 | + | 56586130 | 56586429 | 299, |
| peak22343 | 2.68 | 3.46737E-05 | 0.00074131 | 14.2 | 1.28825E-10 | 3.38844E-09 | chr18 | 56586118 | 56586467 | NM_001353525.2 | 1.3E-10 | + | 56586118 | 56586467 | 349, |
| peak22348 | 2.95 | 4.2658E-05 | 0.000851138 | 9.67 | 3.31131E-06 | 2.04174E-05 | chr18 | 56586141 | 56586442 | NM_001353526.2 | 0.0000033 | + | 56586141 | 56586442 | 301, |
| peak22352 | 2.93 | 0.000144544 | 0.002041738 | 9.67 | 3.54813E-06 | 2.18776E-05 | chr18 | 56586139 | 56586440 | NM_001353530.2 | 0.0000035 | + | 56586139 | 56586440 | 301, |
| peak22357 | 2.82 | 8.12831E-05 | 0.001318257 | 9.67 | 4.16869E-06 | 2.51189E-05 | chr18 | 56586148 | 56586446 | NR_148460.2 | 0.0000042 | + | 56586148 | 56586446 | 298, |
| peak22362 | 2.6 | 1.69824E-05 | 0.000446684 | 15 | 1.20226E-09 | 2.23872E-08 | chr18 | 56586111 | 56586460 | NM_001318728.2 | 1.2E-09 | + | 56586111 | 56586460 | 349, |
| peak22366 | 2.82 | 0.000114815 | 0.001698244 | 9 | 2.95121E-10 | 6.76083E-09 | chr18 | 56586137 | 56586437 | NM_001353531.2 | 3E-10 | + | 56586137 | 56586437 | 300, |
| peak22370 | 2.71 | 3.80189E-05 | 0.000794328 | 15.8 | 5.01187E-16 | 6.30957E-14 | chr18 | 56586120 | 56586471 | NM_001353535.2 | 5E-16 | + | 56586120 | 56586471 | 351, |
| peak22374 | 2.7 | 7.24436E-05 | 0.001258925 | 9.67 | 4.2658E-09 | 6.60693E-08 | chr18 | 56586148 | 56586449 | NM_001353527.2 | 4.3E-09 | + | 56586148 | 56586449 | 301, |
| peak22378 | 3.23 | 6.91831E-05 | 0.001202264 | 17.5 | 2.13796E-09 | 3.71535E-08 | chr18 | 56586128 | 56586427 | NM_001353528.2 | 2.1E-09 | + | 56586128 | 56586427 | 299, |
| peak22382 | 2.5 | 6.91831E-05 | 0.001202264 | 17.5 | 2.88403E-10 | 6.76083E-09 | chr18 | 56586126 | 56586474 | NM_001353529.2 | 2.9E-10 | + | 56586126 | 56586474 | 348, |
| peak22386 | 3.08 | 4.2658E-05 | 0.000851138 | 15.2 | 6.30957E-11 | 1.86209E-09 | chr18 | 56586131 | 56586432 | NM_001353532.2 | 6.3E-11 | + | 56586131 | 56586432 | 301, |
| peak22390 | 3.2 | 7.58578E-05 | 0.001258925 | 17.5 | 1.25893E-11 | 4.67735E-10 | chr18 | 56586127 | 56586427 | NM_001353534.2 | 1.3E-11 | + | 56586127 | 56586427 | 300, |
| peak22394 | 3 | 9.12011E-05 | 0.001479108 | 17.5 | 7.94328E-17 | 1.25893E-14 | chr18 | 56586130 | 56586430 | NM_001353536.2 | 7.9E-17 | + | 56586130 | 56586430 | 300, |
| peak22398 | 3.25 | 6.16595E-05 | 0.001096478 | 17.5 | 1E-16 | 1.58489E-14 | chr18 | 56586128 | 56586427 | NM_001353537.2 | 1E-16 | + | 56586128 | 56586427 | 299, |
| peak22402 | 1.98 | 0.000229087 | 0.002818383 | 17.5 | 2.51189E-17 | 3.98107E-15 | chr18 | 56586124 | 56586571 | NM_001353538.2 | 2.5E-17 | + | 56586124 | 56586571 | 447, |
| peak22433 | 1.72 | 1.20226E-05 | 0.000331131 | 2.8 | 0.000537032 | 0.009120108 | chr18 | 60646115 | 60646516 | NM_194449.4 | 0.00054 | + | 60646115 | 60646516 | 401, |
| peak22447 | 4.4 | 0.002454709 | 0.015848932 | 15 | 2.34423E-07 | 1.99526E-06 | chr18 | 72344317 | 72344618 | NM_001146190.1 | 0.00000023 | + | 72344317 | 72344618 | 301, |
| peak22449 | 4.46 | 0.002187762 | 0.014454398 | 13.1 | 2.88403E-06 | 1.8197E-05 | chr18 | 72344306 | 72344605 | NM_001146189.1 | 0.0000029 | + | 72344306 | 72344605 | 299, |
| peak22451 | 4.72 | 0.001202264 | 0.009549926 | 13 | 1.86209E-05 | 9.33254E-05 | chr18 | 72344311 | 72344610 | NM_017757.2 | 0.000019 | + | 72344311 | 72344610 | 299, |
| peak22524 | 3.67 | 0.000229087 | 0.002818383 | 11.5 | 0.000295121 | 0.001148154 | chr18 | 19153515 | 19153666 | NM_052911.3 | 0.0003 | - | 19153515 | 19153666 | 151, |
| peak22682 | 1.82 | 6.91831E-07 | 3.63078E-05 | 8.34 | 9.77237E-05 | 0.00042658 | chr19 | 3122400 | 3122750 | NM_002067.5 | 0.000098 | + | 3122400 | 3122750 | 350, |
| peak22700 | 2.9 | 0.001318257 | 0.01 | 21 | 1.62181E-06 | 1.09648E-05 | chr19 | 4409019 | 4409713 | NM_005483.3 | 0.0000016 | + | 4409019 | 4409713 | 694, |
| peak22735 | 2.48 | 2.45471E-05 | 0.00057544 | 31.7 | 4.36516E-09 | 6.76083E-08 | chr19 | 7083014 | 7083313 | NM_024341.3 | 4.4E-09 | + | 7083014 | 7083313 | 299, |
| peak22748 | 1.33 | 0.000707946 | 0.006456542 | 9.83 | 6.16595E-09 | 9.12011E-08 | chr19 | 7584189 | 7584485 | NM_018083.5 | 6.2E-09 | + | 7584189 | 7584485 | 296, |
| peak22764 | 4.35 | 0.001819701 | 0.012589254 | 2 | 0.00060256 | 0.01 | chr19 | 9452825 | 9452925 | NM_001202406.1 | 0.0006 | + | 9452825 | 9452925 | 100, |
| peak22766 | 4.35 | 0.001819701 | 0.012589254 | 2 | 0.00057544 | 0.009549926 | chr19 | 9452824 | 9452925 | NM_001202409.1 | 0.00058 | + | 9452824 | 9452925 | 101, |
| peak22768 | 4.35 | 0.001819701 | 0.012589254 | 2 | 0.000190546 | 0.003630781 | chr19 | 9452825 | 9452926 | NM_001202407.2 | 0.00019 | + | 9452825 | 9452926 | 101, |
| peak22799 | 4.94 | 1.8197E-05 | 0.000467735 | 10 | 0.000870964 | 0.003162278 | chr19 | 11242953 | 11243054 | NM_000527.5 | 0.00087 | + | 11242953 | 11243054 | 101, |
| peak22803 | 4.94 | 3.0903E-06 | 0.000114815 | 9.4 | 3.54813E-06 | 2.13796E-05 | chr19 | 11242955 | 11243055 | NM_001195798.2 | 0.0000035 | + | 11242955 | 11243055 | 100, |
| peak22806 | 4.75 | 2.95121E-05 | 0.000660693 | 11.6 | 0.000162181 | 0.000660693 | chr19 | 11242949 | 11243050 | NM_001195803.2 | 0.00016 | + | 11242949 | 11243050 | 101, |
| peak22824 | 1.12 | 0.001096478 | 0.008912509 | 6.45 | 4.57088E-08 | 5.01187E-07 | chr19 | 12903485 | 12903783 | NM_002229.3 | 0.000000046 | + | 12903485 | 12903783 | 298, |
| peak22892 | 2.93 | 0.002630268 | 0.016595869 | 7.6 | 9.77237E-07 | 7.07946E-06 | chr19 | 18779132 | 18779382 | NM_001345982.2 | 0.00000098 | + | 18779132 | 18779382 | 250, |
| peak22896 | 2.59 | 0.003311311 | 0.019054607 | 9 | 1.86209E-05 | 9.33254E-05 | chr19 | 18779119 | 18779417 | NM_018316.3 | 0.000019 | + | 18779119 | 18779417 | 298, |

| | | | | | | | | | | | | | | | |
|---|---|---|---|---|---|---|---|---|---|---|---|---|---|---|---|
| peak22911 | 3.82 | 2.18776E-06 | 8.91251E-05 | 8.62 | 1.94984E-08 | 2.39883E-07 | chr19 | 19466865 | 19467216 | NM_015329.4 | 0.000000019 | + | 19466865 | 19467216 | 351, |
| peak22929 | 2.22 | 0.003388442 | 0.019498446 | 9.75 | 5.37032E-06 | 3.0903E-05 | chr19 | 20003055 | 20003353 | NM_001331133.1 | 0.0000054 | + | 20003055 | 20003353 | 298, |
| peak22930 | 2.35 | 0.001023293 | 0.00851138 | 9.75 | 1.86209E-05 | 9.33254E-05 | chr19 | 20003053 | 20003352 | NM_001331134.1 | 0.000019 | + | 20003053 | 20003352 | 299, |
| peak22931 | 1.33 | 0.000416869 | 0.004365158 | 9.75 | 2.88403E-06 | 1.8197E-05 | chr19 | 20003038 | 20003438 | NM_021047.3 | 0.0000029 | + | 20003038 | 20003438 | 400, |
| peak22978 | 3.42 | 0.000933254 | 0.007943282 | 3.65 | 0.00030903 | 0.001230269 | chr19 | 32971339 | 32973060 | NM_001172774.2 | 0.00031 | + | 32971339 | 32973060 | 124,76, |
| peak22986 | 1.41 | 0.002089296 | 0.014125375 | 14.2 | 0.004265795 | 0.014454398 | chr19 | 34303847 | 34303947 | NM_001129995.2 | 0.0043 | + | 34303847 | 34303947 | 100, |
| peak22987 | 1.47 | 0.002630268 | 0.016595869 | 15.6 | 0.000467735 | 0.001778279 | chr19 | 34303840 | 34303940 | NM_024076.3 | 0.00047 | + | 34303840 | 34303940 | 100, |
| peak22997 | 1.53 | 0.003311311 | 0.019498446 | 36.6 | 8.12831E-09 | 1.14815E-07 | chr19 | 36726590 | 36726889 | NM_001099638.1 | 8.1E-09 | + | 36726590 | 36726889 | 299, |
| peak23000 | 1.06 | 0.004365158 | 0.023442288 | 39.8 | 6.30957E-13 | 3.16228E-11 | chr19 | 36726582 | 36726883 | NM_001099639.1 | 6.3E-13 | + | 36726582 | 36726883 | 301, |
| peak23075 | 1.89 | 5.01187E-05 | 0.000954993 | 4.43 | 0.000125893 | 0.000537032 | chr19 | 39667256 | 39668415 | NM_001014831.3 | 0.00013 | + | 39667256 | 39668415 | 99,101, |
| peak23078 | 1.89 | 5.01187E-05 | 0.000954993 | 4.43 | 3.71535E-06 | 2.29087E-05 | chr19 | 39667256 | 39668415 | NM_001014832.2 | 0.0000037 | + | 39667256 | 39668415 | 99,101, |
| peak23081 | 1.53 | 7.07946E-05 | 0.001230269 | 5.25 | 2.88403E-07 | 2.39883E-06 | chr19 | 39667265 | 39668374 | NM_001014834.3 | 0.00000029 | + | 39667265 | 39668374 | 90,60, |
| peak23084 | 1.47 | 0.00018197 | 0.002344229 | 6.85 | 3.0903E-07 | 2.5704E-06 | chr19 | 39667246 | 39668406 | NM_001014835.2 | 0.00000031 | + | 39667246 | 39668406 | 109,92, |
| peak23087 | 1.6 | 0.002691535 | 0.016595869 | 5.95 | 2.69153E-06 | 1.69824E-05 | chr19 | 39667289 | 39668398 | NM_005884.4 | 0.0000027 | + | 39667289 | 39668398 | 66,84, |
| peak23096 | 4.12 | 0.000288403 | 0.003311311 | 5.67 | 0.000269153 | 0.004897788 | chr19 | 39915905 | 39916056 | NM_022835.3 | 0.00027 | + | 39915905 | 39916056 | 151, |
| peak23108 | 1.51 | 0.000851138 | 0.007413102 | 1.54 | 0.00030903 | 0.001230269 | chr19 | 41798163 | 41798313 | NM_144732.5 | 0.00031 | + | 41798163 | 41798313 | 150, |
| peak23154 | 1.45 | 0.00128825 | 0.009772372 | 5.33 | 0.000676083 | 0.011220185 | chr19 | 44610579 | 44610827 | NM_001321645.2 | 0.00068 | + | 44610579 | 44610827 | 248, |
| peak23157 | 1.35 | 0.001995262 | 0.013803843 | 6.67 | 0.000354813 | 0.001380384 | chr19 | 44610573 | 44610821 | NM_013398.4 | 0.00035 | + | 44610573 | 44610821 | 248, |
| peak23164 | 2.57 | 7.4131E-05 | 0.001258925 | 5.69 | 3.16228E-05 | 0.000151356 | chr19 | 45166189 | 45166439 | NM_001135769.3 | 0.000032 | + | 45166189 | 45166439 | 250, |
| peak23167 | 2.5 | 2.95121E-05 | 0.000660693 | 5.56 | 1.54882E-05 | 7.94328E-05 | chr19 | 45166183 | 45166434 | NM_006505.5 | 0.000015 | + | 45166183 | 45166434 | 251, |
| peak23209 | 1.87 | 0.000977237 | 0.008128305 | 28.8 | 6.60693E-05 | 0.000295121 | chr19 | 47424927 | 47440534 | NM_004491.5 | 0.000066 | + | 47424927 | 47440534 | 686,14, |
| peak23229 | 1.53 | 0.004677351 | 0.024547089 | 4.5 | 7.07946E-05 | 0.000316228 | chr19 | 52327906 | 52328353 | NM_002030.5 | 0.000071 | + | 52327906 | 52328353 | 447, |
| peak23235 | 1.63 | 1.14815E-05 | 0.000323594 | 7 | 0.00047863 | 0.001819701 | chr19 | 52827887 | 52828335 | NM_001297625.1 | 0.00048 | + | 52827887 | 52828335 | 448, |
| peak23275 | 2.46 | 0.001659587 | 0.012022644 | 14.1 | 0.009772372 | 0.031622777 | chr19 | 56127929 | 56128376 | NM_001195605.1 | 0.0098 | + | 56127929 | 56128376 | 447, |
| peak23281 | 3.02 | 0.000549541 | 0.005370318 | 6.75 | 4.36516E-08 | 2.75423E-06 | chr19 | 56888971 | 56889170 | NR_024055.2 | 0.000000044 | + | 56888971 | 56889170 | 199, |
| peak23284 | 2.27 | 3.63078E-05 | 0.000758578 | 6.5 | 9.12011E-08 | 5.12861E-06 | chr19 | 56888851 | 56889201 | NR_024057.2 | 0.000000091 | + | 56888851 | 56889201 | 350, |
| peak23286 | 2.27 | 3.63078E-05 | 0.000758578 | 6.75 | 1.69824E-07 | 8.70964E-06 | chr19 | 56888857 | 56889207 | NR_033418.1 | 0.00000017 | + | 56888857 | 56889207 | 350, |
| peak23287 | 2.65 | 0.003801894 | 0.021379621 | 13 | 0.000223872 | 0.000891251 | chr19 | 56934359 | 56934559 | NM_001159860.1 | 0.00022 | + | 56934359 | 56934559 | 200, |
| peak23293 | 2.51 | 0.000107152 | 0.00162181 | 9.2 | 5.88844E-07 | 4.46684E-06 | chr19 | 57765173 | 57765722 | NM_001023563.4 | 0.00000059 | + | 57765173 | 57765722 | 549, |
| peak23297 | 2.34 | 5.24807E-05 | 0.001 | 10.7 | 4.57088E-06 | 2.75423E-05 | chr19 | 57765153 | 57765654 | NM_001145078.2 | 0.0000046 | + | 57765153 | 57765654 | 501, |
| peak23307 | 2.61 | 1.09648E-05 | 0.000316228 | 16.9 | 0.001096478 | 0.003981072 | chr19 | 57910050 | 57910348 | NM_152909.3 | 0.0011 | + | 57910050 | 57910348 | 298, |
| peak23308 | 2.51 | 6.60693E-07 | 3.38844E-05 | 29.8 | 0.000724436 | 0.002691535 | chr19 | 57910071 | 57910371 | NM_001172773.2 | 0.00072 | + | 57910071 | 57910371 | 300, |
| peak23369 | 1.61 | 0.004073803 | 0.022387211 | 9.57 | 2.39883E-06 | 1.54882E-05 | chr19 | 58805843 | 58806044 | NM_021089.3 | 0.0000024 | + | 58805843 | 58806044 | 201, |
| peak23405 | 1.19 | 0.003090295 | 0.018620871 | 18.1 | 0.00074131 | 0.002754229 | chr19 | 3631124 | 3631373 | NM_012398.3 | 0.00074 | - | 3631124 | 3631373 | 249, |
| peak23438 | 3.24 | 5.88844E-06 | 0.000190546 | 5.11 | 0.000870964 | 0.003162278 | chr19 | 6441815 | 6442064 | NM_024103.3 | 0.00087 | - | 6441815 | 6442064 | 249, |
| peak23452 | 4.44 | 0.000229087 | 0.002754229 | 4.71 | 0.000354813 | 0.00616595 | chr19 | 9523350 | 9523451 | NM_001271314.1 | 0.00035 | - | 9523350 | 9523451 | 101, |
| peak23453 | 2.99 | 9.54993E-05 | 0.001479108 | 3.33 | 0.000109648 | 0.002238721 | chr19 | 9523749 | 9523949 | NM_001271314.1 | 0.00011 | - | 9523749 | 9523949 | 200, |
| peak23455 | 4.44 | 0.000229087 | 0.002754229 | 4.71 | 0.000269153 | 0.004897788 | chr19 | 9523350 | 9523451 | NM_006631.3 | 0.00027 | - | 9523350 | 9523451 | 101, |
| peak23456 | 3.03 | 9.54993E-05 | 0.001479108 | 3.33 | 5.7544E-05 | 0.001258925 | chr19 | 9523749 | 9523950 | NM_006631.3 | 0.000058 | - | 9523749 | 9523950 | 201, |
| peak23458 | 4.44 | 0.000229087 | 0.002754229 | 4.71 | 0.001348963 | 0.021379621 | chr19 | 9523354 | 9523454 | NM_001370374.1 | 0.0013 | - | 9523354 | 9523454 | 100, |
| peak23459 | 3.03 | 9.54993E-05 | 0.001479108 | 3.33 | 0.001348963 | 0.021379621 | chr19 | 9523753 | 9523953 | NM_001370374.1 | 0.0013 | - | 9523753 | 9523953 | 200, |
| peak23460 | 4.44 | 0.000229087 | 0.002754229 | 4.71 | 0.001862087 | 0.028183829 | chr19 | 9523353 | 9523454 | NM_001370375.1 | 0.0019 | - | 9523353 | 9523454 | 101, |
| peak23461 | 3.03 | 9.54993E-05 | 0.001479108 | 3.33 | 0.001862087 | 0.028183829 | chr19 | 9523752 | 9523953 | NM_001370375.1 | 0.0019 | - | 9523752 | 9523953 | 201, |
| peak23464 | 4.44 | 0.000229087 | 0.002754229 | 4.71 | 0.000165959 | 0.003235937 | chr19 | 9523352 | 9523452 | NM_001370376.1 | 0.00017 | - | 9523352 | 9523452 | 100, |
| peak23465 | 2.99 | 9.54993E-05 | 0.001479108 | 3.33 | 3.71535E-05 | 0.000870964 | chr19 | 9523749 | 9523998 | NM_001370376.1 | 0.000037 | - | 9523749 | 9523998 | 249, |
| peak23468 | 4.44 | 0.000229087 | 0.002754229 | 4.71 | 0.00040738 | 0.007079458 | chr19 | 9523352 | 9523452 | NM_001370377.1 | 0.00041 | - | 9523352 | 9523452 | 100, |
| peak23469 | 2.99 | 9.54993E-05 | 0.001479108 | 3.33 | 9.54993E-05 | 0.001949845 | chr19 | 9523748 | 9523997 | NM_001370377.1 | 0.000095 | - | 9523748 | 9523997 | 249, |
| peak23471 | 4.44 | 0.000229087 | 0.002754229 | 4.71 | 0.000758578 | 0.012302688 | chr19 | 9523353 | 9523454 | NM_001370378.1 | 0.00076 | - | 9523353 | 9523454 | 101, |
| peak23472 | 3.03 | 9.54993E-05 | 0.001479108 | 3.33 | 0.000758578 | 0.012302688 | chr19 | 9523752 | 9523952 | NM_001370378.1 | 0.00076 | - | 9523752 | 9523952 | 200, |
| peak23475 | 4.44 | 0.000229087 | 0.002754229 | 4.71 | 0.00018197 | 0.003467369 | chr19 | 9523351 | 9523451 | NM_001370379.1 | 0.00018 | - | 9523351 | 9523451 | 100, |
| peak23476 | 2.98 | 0.000354813 | 0.003890451 | 3.33 | 8.70964E-05 | 0.001819701 | chr19 | 9523747 | 9523996 | NM_001370379.1 | 0.000087 | - | 9523747 | 9523996 | 249, |
| peak23479 | 4.44 | 0.000229087 | 0.002754229 | 4.71 | 0.000269153 | 0.004897788 | chr19 | 9523352 | 9523452 | NM_001370380.1 | 0.00027 | - | 9523352 | 9523452 | 100, |
| peak23480 | 2.99 | 9.54993E-05 | 0.001479108 | 3.33 | 6.0256E-05 | 0.001318257 | chr19 | 9523748 | 9523996 | NM_001370380.1 | 0.00006 | - | 9523748 | 9523996 | 248, |
| peak23483 | 4.44 | 0.000229087 | 0.002754229 | 4.71 | 0.001318257 | 0.020417379 | chr19 | 9523353 | 9523454 | NM_001370381.1 | 0.0013 | - | 9523353 | 9523454 | 101, |
| peak23484 | 3.03 | 9.54993E-05 | 0.001479108 | 3.33 | 0.001318257 | 0.020417379 | chr19 | 9523752 | 9523953 | NM_001370381.1 | 0.0013 | - | 9523752 | 9523953 | 201, |
| peak23485 | 4.44 | 0.000229087 | 0.002754229 | 4.71 | 0.000389045 | 0.00676083 | chr19 | 9523351 | 9523451 | NM_001370382.1 | 0.00039 | - | 9523351 | 9523451 | 100, |
| peak23486 | 2.99 | 0.000154882 | 0.002137962 | 3.33 | 0.000114815 | 0.002290868 | chr19 | 9523747 | 9523995 | NM_001370382.1 | 0.00011 | - | 9523747 | 9523995 | 248, |

| | | | | | | | | | | | | | | | |
|---|---|---|---|---|---|---|---|---|---|---|---|---|---|---|---|
| peak23488 | 4.44 | 0.000229087 | 0.002754229 | 4.71 | 0.000588844 | 0.009772372 | chr19 | 9523353 | 9523453 | NM_001370383.1 | 0.00059 | - | 9523353 | 9523453 | 100, |
| peak23489 | 3.03 | 9.54993E-05 | 0.001479108 | 3.33 | 0.000190546 | 0.003630781 | chr19 | 9523751 | 9523951 | NM_001370383.1 | 0.00019 | - | 9523751 | 9523951 | 200, |
| peak23491 | 4.44 | 0.000229087 | 0.002754229 | 4.71 | 0.000758578 | 0.012302688 | chr19 | 9523352 | 9523452 | NM_001370384.1 | 0.00076 | - | 9523352 | 9523452 | 100, |
| peak23492 | 2.96 | 9.33254E-05 | 0.001479108 | 3.33 | 0.000245471 | 0.004466836 | chr19 | 9523749 | 9523998 | NM_001370384.1 | 0.00025 | - | 9523749 | 9523998 | 249, |
| peak23495 | 4.44 | 0.000229087 | 0.002754229 | 4.71 | 0.000870964 | 0.014125375 | chr19 | 9523352 | 9523453 | NM_001370386.1 | 0.00087 | - | 9523352 | 9523453 | 101, |
| peak23496 | 3 | 9.33254E-05 | 0.001479108 | 3.33 | 0.000870964 | 0.014125375 | chr19 | 9523750 | 9523949 | NM_001370386.1 | 0.00087 | - | 9523750 | 9523949 | 199, |
| peak23500 | 4.44 | 0.000229087 | 0.002754229 | 4.71 | 0.000229087 | 0.004265795 | chr19 | 9523352 | 9523452 | NM_001370387.1 | 0.00023 | - | 9523352 | 9523452 | 100, |
| peak23501 | 2.99 | 9.54993E-05 | 0.001479108 | 3.33 | 5.37032E-05 | 0.001174898 | chr19 | 9523749 | 9523998 | NM_001370387.1 | 0.000054 | - | 9523749 | 9523998 | 249, |
| peak23504 | 4.44 | 0.000229087 | 0.002754229 | 4.71 | 0.000467735 | 0.007943282 | chr19 | 9523352 | 9523453 | NM_001370388.1 | 0.00047 | - | 9523352 | 9523453 | 101, |
| peak23505 | 3.03 | 9.54993E-05 | 0.001479108 | 3.33 | 0.000128825 | 0.002570396 | chr19 | 9523750 | 9523950 | NM_001370388.1 | 0.00013 | - | 9523750 | 9523950 | 200, |
| peak23507 | 4.44 | 0.000229087 | 0.002754229 | 4.71 | 0.001258925 | 0.019952623 | chr19 | 9523353 | 9523453 | NM_001370389.1 | 0.0013 | - | 9523353 | 9523453 | 100, |
| peak23508 | 3.03 | 9.54993E-05 | 0.001479108 | 3.33 | 0.001258925 | 0.019952623 | chr19 | 9523751 | 9523950 | NM_001370389.1 | 0.0013 | - | 9523751 | 9523950 | 199, |
| peak23509 | 4.44 | 0.000229087 | 0.002754229 | 4.71 | 0.000331131 | 0.005888437 | chr19 | 9523354 | 9523455 | NM_001370390.1 | 0.00033 | - | 9523354 | 9523455 | 101, |
| peak23510 | 3.03 | 0.000141254 | 0.001995262 | 4.4 | 0.000331131 | 0.005888437 | chr19 | 9523754 | 9523955 | NM_001370390.1 | 0.00033 | - | 9523754 | 9523955 | 201, |
| peak23512 | 4.44 | 0.000229087 | 0.002754229 | 4.71 | 0.000162181 | 0.003162278 | chr19 | 9523351 | 9523451 | NM_001370391.1 | 0.00016 | - | 9523351 | 9523451 | 100, |
| peak23513 | 2.99 | 0.000154882 | 0.002137962 | 3.33 | 4.0738E-05 | 0.000933254 | chr19 | 9523747 | 9523995 | NM_001370391.1 | 0.000041 | - | 9523747 | 9523995 | 248, |
| peak23517 | 4.44 | 0.000229087 | 0.002754229 | 4.71 | 0.000190546 | 0.003630781 | chr19 | 9523351 | 9523451 | NM_001370392.1 | 0.00019 | - | 9523351 | 9523451 | 100, |
| peak23518 | 2.99 | 0.000154882 | 0.002137962 | 3.33 | 5.37032E-05 | 0.001174898 | chr19 | 9523746 | 9523994 | NM_001370392.1 | 0.000054 | - | 9523746 | 9523994 | 248, |
| peak23522 | 4.44 | 0.000229087 | 0.002754229 | 4.71 | 0.000489779 | 0.008317638 | chr19 | 9523354 | 9523454 | NM_001370393.1 | 0.00049 | - | 9523354 | 9523454 | 100, |
| peak23523 | 2.77 | 0.00017378 | 0.002344229 | 4.4 | 0.000489779 | 0.008317638 | chr19 | 9523753 | 9523954 | NM_001370393.1 | 0.00049 | - | 9523753 | 9523954 | 201, |
| peak23526 | 4.44 | 0.000229087 | 0.002754229 | 4.71 | 0.000660693 | 0.010964782 | chr19 | 9523352 | 9523452 | NM_001370394.1 | 0.00066 | - | 9523352 | 9523452 | 100, |
| peak23527 | 2.99 | 9.54993E-05 | 0.001479108 | 3.33 | 0.000169824 | 0.003235937 | chr19 | 9523749 | 9523998 | NM_001370394.1 | 0.00017 | - | 9523749 | 9523998 | 249, |
| peak23530 | 4.44 | 0.000229087 | 0.002754229 | 4.71 | 0.000501187 | 0.00851138 | chr19 | 9523354 | 9523455 | NM_001370395.1 | 0.0005 | - | 9523354 | 9523455 | 101, |
| peak23531 | 3.03 | 0.000141254 | 0.001995262 | 4.4 | 0.000501187 | 0.00851138 | chr19 | 9523754 | 9523955 | NM_001370395.1 | 0.0005 | - | 9523754 | 9523955 | 201, |
| peak23533 | 4.44 | 0.000229087 | 0.002754229 | 4.71 | 0.00017378 | 0.003311311 | chr19 | 9523352 | 9523452 | NM_001370396.1 | 0.00017 | - | 9523352 | 9523452 | 100, |
| peak23534 | 2.99 | 9.54993E-05 | 0.001479108 | 3.33 | 4.0738E-05 | 0.000933254 | chr19 | 9523749 | 9523998 | NM_001370396.1 | 0.000041 | - | 9523749 | 9523998 | 249, |
| peak23537 | 4.44 | 0.000229087 | 0.002754229 | 4.71 | 0.000645654 | 0.010715193 | chr19 | 9523353 | 9523454 | NM_001370397.1 | 0.00065 | - | 9523353 | 9523454 | 101, |
| peak23538 | 3.03 | 9.54993E-05 | 0.001479108 | 3.33 | 0.000645654 | 0.010715193 | chr19 | 9523752 | 9523953 | NM_001370397.1 | 0.00065 | - | 9523752 | 9523953 | 201, |
| peak23541 | 4.44 | 0.000229087 | 0.002754229 | 4.71 | 0.000204174 | 0.003890451 | chr19 | 9523354 | 9523454 | NM_001370398.1 | 0.0002 | - | 9523354 | 9523454 | 100, |
| peak23542 | 2.77 | 0.00017378 | 0.002344229 | 4.4 | 0.000204174 | 0.003890451 | chr19 | 9523753 | 9523954 | NM_001370398.1 | 0.0002 | - | 9523753 | 9523954 | 201, |
| peak23545 | 4.44 | 0.000229087 | 0.002754229 | 4.71 | 0.000190546 | 0.003630781 | chr19 | 9523353 | 9523453 | NM_001370399.1 | 0.00019 | - | 9523353 | 9523453 | 100, |
| peak23546 | 3.03 | 9.54993E-05 | 0.001479108 | 3.33 | 4.67735E-05 | 0.001047129 | chr19 | 9523751 | 9523950 | NM_001370399.1 | 0.000047 | - | 9523751 | 9523950 | 199, |
| peak23548 | 4.44 | 0.000229087 | 0.002754229 | 4.71 | 0.000323594 | 0.005754399 | chr19 | 9523353 | 9523454 | NM_001370400.1 | 0.00032 | - | 9523353 | 9523454 | 101, |
| peak23549 | 3.03 | 9.54993E-05 | 0.001479108 | 3.33 | 0.000323594 | 0.005754399 | chr19 | 9523753 | 9523953 | NM_001370400.1 | 0.00032 | - | 9523753 | 9523953 | 200, |
| peak23568 | 2.28 | 7.07946E-05 | 0.001230269 | 17.3 | 6.30957E-11 | 1.7378E-09 | chr19 | 9763771 | 9767237 | NM_001130031.2 | 6.3E-11 | - | 9763771 | 9767237 | 786,15, |
| peak23569 | 2.34 | 1.65959E-05 | 0.000436516 | 17.2 | 6.30957E-11 | 1.69824E-09 | chr19 | 9763769 | 9767234 | NM_001130032.2 | 6.3E-11 | - | 9763769 | 9767234 | 788,12, |
| peak23571 | 2.74 | 8.51138E-05 | 0.001380384 | 17.2 | 2.51189E-09 | 4.16869E-08 | chr19 | 9763956 | 9764506 | NM_017656.4 | 2.5E-09 | - | 9763956 | 9764506 | 550, |
| peak23572 | 2.32 | 2.95121E-05 | 0.000660693 | 17.2 | 9.12011E-10 | 1.77828E-08 | chr19 | 9763770 | 9767237 | NM_001300885.2 | 9.1E-10 | - | 9763770 | 9767237 | 784,15, |
| peak23586 | 3.31 | 5.37032E-07 | 2.88403E-05 | 18.7 | 2.04174E-05 | 0.000102329 | chr19 | 11256751 | 11256995 | NM_001317031.1 | 0.00002 | - | 11256751 | 11256995 | 244, |
| peak23593 | 4.56 | 6.0256E-06 | 0.000194984 | 6.75 | 3.31131E-05 | 0.000794328 | chr19 | 12256725 | 12257017 | NM_145233.4 | 0.000033 | - | 12256725 | 12257017 | 292, |
| peak23595 | 4.89 | 1.44544E-05 | 0.000389045 | 7.5 | 1.7378E-05 | 0.000446684 | chr19 | 12256702 | 12257003 | NR_037801.2 | 0.000017 | - | 12256702 | 12257003 | 301, |
| peak23622 | 1.05 | 2.13796E-05 | 0.000524807 | 2.84 | 0.008912509 | 0.029512092 | chr19 | 15379759 | 15391112 | NM_058243.2 | 0.0089 | - | 15379759 | 15391112 | 94,319,38, |
| peak23663 | 3.91 | 0.000870964 | 0.007585776 | 5.6 | 0.002754229 | 0.009549926 | chr19 | 19823501 | 19823700 | NM_021030.3 | 0.0028 | - | 19823501 | 19823700 | 199, |
| peak23700 | 2.89 | 0.002290868 | 0.015135612 | 3 | 0.00144544 | 0.022387211 | chr19 | 37038531 | 37038632 | NM_001352273.2 | 0.0014 | - | 37038531 | 37038632 | 101, |
| peak23710 | 5.23 | 0.004073803 | 0.022387211 | 2.25 | 1.12202E-05 | 0.00030903 | chr19 | 38187857 | 38187957 | NM_001172677.1 | 0.000011 | - | 38187857 | 38187957 | 100, |
| peak23712 | 5.23 | 0.004073803 | 0.022387211 | 2.25 | 2.81838E-06 | 9.54993E-05 | chr19 | 38187868 | 38187968 | NM_032689.5 | 0.0000028 | - | 38187868 | 38187968 | 100, |
| peak23737 | 4.96 | 4.67735E-06 | 0.000162181 | 12.3 | 0.009549926 | 0.031622777 | chr19 | 42702898 | 42702998 | NR_073048.2 | 0.0095 | - | 42702898 | 42702998 | 100, |
| peak23756 | 2.7 | 0.00020893 | 0.002691535 | 4.56 | 0.000169824 | 0.000707946 | chr19 | 43031473 | 43032583 | NM_001205344.2 | 0.00017 | - | 43031473 | 43032583 | 79,121, |
| peak23757 | 2.42 | 0.001698244 | 0.012022644 | 5.17 | 2.13796E-07 | 1.07152E-05 | chr19 | 44610627 | 44610826 | NR_033341.1 | 0.00000021 | - | 44610627 | 44610826 | 199, |
| peak23829 | 3.32 | 0.004365158 | 0.023442288 | 1.9 | 6.91831E-07 | 2.88403E-05 | chr19 | 48714265 | 48714366 | NM_001351786.2 | 0.00000069 | - | 48714265 | 48714366 | 101, |
| peak23859 | 1.75 | 0.00057544 | 0.005495409 | 14.4 | 0.000588844 | 0.002238721 | chr19 | 51301594 | 51301694 | NM_001290150.1 | 0.00059 | - | 51301594 | 51301694 | 100, |
| peak23860 | 1.48 | 0.002290868 | 0.015135612 | 14.6 | 5.49541E-05 | 0.000251189 | chr19 | 51301595 | 51301696 | NM_001290151.1 | 0.000055 | - | 51301595 | 51301696 | 101, |
| peak23861 | 1.94 | 0.000169824 | 0.002238721 | 17.2 | 1.62181E-06 | 1.09648E-05 | chr19 | 51301591 | 51301691 | NM_001290153.1 | 0.0000016 | - | 51301591 | 51301691 | 100, |
| peak23862 | 1.92 | 0.00025704 | 0.003019952 | 14.4 | 4.36516E-05 | 0.000199526 | chr19 | 51301594 | 51301694 | NM_001290154.1 | 0.000044 | - | 51301594 | 51301694 | 100, |
| peak23863 | 1.87 | 0.000158489 | 0.002137962 | 18.8 | 5.01187E-05 | 0.000229087 | chr19 | 51301589 | 51301689 | NM_001290155.1 | 0.00005 | - | 51301589 | 51301689 | 100, |
| peak23864 | 3.16 | 7.94328E-05 | 0.001318257 | 15.7 | 2.75423E-08 | 3.23594E-07 | chr19 | 52394182 | 52394383 | NM_023074.4 | 0.000000028 | - | 52394182 | 52394383 | 201, |
| peak23914 | 2.55 | 0.001862087 | 0.012882496 | 2 | 6.30957E-14 | 2.51189E-11 | chr19 | 53645125 | 53645276 | NM_001172675.2 | 6.3E-14 | - | 53645125 | 53645276 | 151, |

| | | | | | | | | | | | | | | | |
|---|---|---|---|---|---|---|---|---|---|---|---|---|---|---|---|
| peak23917 | 2.55 | 0.001862087 | 0.012882496 | 2.5 | 1E-14 | 6.30957E-12 | chr19 | 53645122 | 53645273 | NM_032584.3 | 1E-14 | - | 53645122 | 53645273 | 151, |
| peak23919 | 2.55 | 0.001862087 | 0.012882496 | 2.5 | 0.000870964 | 0.014125375 | chr19 | 53645123 | 53645273 | NM_001172674.1 | 0.00087 | - | 53645123 | 53645273 | 150, |
| peak23946 | 2.23 | 9.54993E-09 | 7.76247E-07 | 54.6 | 0.000676083 | 0.002511886 | chr20 | 2097459 | 2097858 | NM_080836.4 | 0.00068 | + | 2097459 | 2097858 | 399, |
| peak23978 | 2.73 | 7.76247E-07 | 3.89045E-05 | 2.58 | 5.37032E-08 | 5.7544E-07 | chr20 | 11903558 | 11903858 | NM_001282552.2 | 0.000000054 | + | 11903558 | 11903858 | 300, |
| peak23980 | 2.13 | 0.000263027 | 0.003090295 | 2.81 | 2.81838E-08 | 3.31131E-07 | chr20 | 11903465 | 11903864 | NM_181443.3 | 0.000000028 | + | 11903465 | 11903864 | 399, |
| peak23983 | 2.34 | 2.29087E-05 | 0.000549541 | 2.8 | 4.7863E-08 | 5.12861E-07 | chr20 | 11903503 | 11903853 | NM_001282551.1 | 0.000000048 | + | 11903503 | 11903853 | 350, |
| peak23987 | 2.66 | 5.7544E-06 | 0.000190546 | 2.58 | 1.47911E-08 | 1.90546E-07 | chr20 | 11903557 | 11903807 | NM_014962.4 | 0.000000015 | + | 11903557 | 11903807 | 250, |
| peak23999 | 5.93 | 1.7378E-05 | 0.000446684 | 16.5 | 5.88844E-08 | 6.16595E-07 | chr20 | 17970732 | 17970978 | NM_001363738.1 | 0.000000059 | + | 17970732 | 17970978 | 246, |
| peak24001 | 5.92 | 1.07152E-05 | 0.00030903 | 16.4 | 7.24436E-06 | 4.0738E-05 | chr20 | 17970727 | 17970975 | NM_001310339.2 | 0.0000072 | + | 17970727 | 17970975 | 248, |
| peak24005 | 6.62 | 5.62341E-05 | 0.001023293 | 15 | 8.12831E-05 | 0.000354813 | chr20 | 17970822 | 17970972 | NM_052865.4 | 0.000081 | + | 17970822 | 17970972 | 150, |
| peak24036 | 1.3 | 0.003715352 | 0.020892961 | 4.17 | 3.71535E-05 | 0.00017378 | chr20 | 18295966 | 18296263 | NM_001352460.2 | 0.000037 | + | 18295966 | 18296263 | 297, |
| peak24046 | 6.5 | 5.37032E-08 | 3.89045E-06 | 4.62 | 0.000467735 | 0.001778279 | chr20 | 19702825 | 19703024 | NM_020689.4 | 0.00047 | + | 19702825 | 19703024 | 199, |
| peak24074 | 1.02 | 0.004897788 | 0.025118864 | 7.11 | 0.001230269 | 0.004466836 | chr20 | 25206326 | 25206525 | NM_001322386.1 | 0.0012 | + | 25206326 | 25206525 | 199, |
| peak24093 | 2.87 | 0.002137962 | 0.014125375 | 15.6 | 9.33254E-08 | 9.12011E-07 | chr20 | 30754561 | 30754762 | NM_001363731.1 | 0.000000093 | + | 30754561 | 30754762 | 201, |
| peak24100 | 1.38 | 8.70964E-05 | 0.001412538 | 26.2 | 0.000158489 | 0.000660693 | chr20 | 30922018 | 30922516 | NM_004798.4 | 0.00016 | + | 30922018 | 30922516 | 498, |
| peak24118 | 1.61 | 0.00040738 | 0.004365158 | 40.1 | 1.44544E-10 | 3.71535E-09 | chr20 | 32232790 | 32233290 | NM_001032999.3 | 1.4E-10 | + | 32232790 | 32233290 | 500, |
| peak24120 | 1.61 | 0.00040738 | 0.004365158 | 47.8 | 5.62341E-10 | 1.20226E-08 | chr20 | 32232787 | 32233288 | NM_001039709.1 | 5.6E-10 | + | 32232787 | 32233288 | 501, |
| peak24123 | 1.67 | 0.000331131 | 0.003715352 | 38.3 | 7.94328E-11 | 2.18776E-09 | chr20 | 32232795 | 32233295 | NM_005093.3 | 7.9E-11 | + | 32232795 | 32233295 | 500, |
| peak24131 | 2.86 | 0.002238721 | 0.014791084 | 5.62 | 1.69824E-06 | 1.14815E-05 | chr20 | 33122567 | 33122714 | NM_001319157.1 | 0.0000017 | + | 33122567 | 33122714 | 147, |
| peak24145 | 3.26 | 0.001348963 | 0.01023293 | 4.89 | 1.02329E-07 | 0.000001 | chr20 | 34092089 | 34092389 | NM_007186.6 | 0.0000001 | + | 34092089 | 34092389 | 300, |
| peak24197 | 1.91 | 0.003388442 | 0.019498446 | 8.8 | 0.000109648 | 0.000467735 | chr20 | 39987428 | 39987529 | NR_126051.2 | 0.00011 | + | 39987428 | 39987529 | 101, |
| peak24205 | 3.26 | 0.000141254 | 0.001995262 | 2 | 0.001096478 | 0.003981072 | chr20 | 43595201 | 43600824 | NR_147975.2 | 0.0011 | + | 43595201 | 43600824 | 43,106, |
| peak24206 | 3.01 | 0.000363078 | 0.003981072 | 2.18 | 4.67735E-06 | 2.75423E-05 | chr20 | 43595202 | 43601968 | NR_147974.2 | 0.0000047 | + | 43595202 | 43601968 | 42,81,27, |
| peak24207 | 2.48 | 0.000645654 | 0.006025596 | 2.15 | 3.80189E-05 | 0.000177828 | chr20 | 43595202 | 43607159 | NM_001352385.2 | 0.000038 | + | 43595202 | 43607159 | 42,81,76, |
| peak24209 | 1.8 | 0.000102329 | 0.001584893 | 6.67 | 3.80189E-05 | 0.000177828 | chr20 | 43703741 | 43703892 | NM_001352385.2 | 0.000038 | + | 43703741 | 43703892 | 151, |
| peak24214 | 4.7 | 1.99526E-05 | 0.000489779 | 19.7 | 0.000354813 | 0.001380384 | chr20 | 44511235 | 44511434 | NM_080603.5 | 0.00035 | + | 44511235 | 44511434 | 199, |
| peak24215 | 2.32 | 0.001513561 | 0.010964782 | 15.8 | 8.91251E-05 | 0.000389045 | chr20 | 44512026 | 44512274 | NM_080603.5 | 0.000089 | + | 44512026 | 44512274 | 248, |
| peak24243 | 1.06 | 0.00074131 | 0.00676083 | 2 | 0.000389045 | 0.001513561 | chr20 | 49196350 | 49197837 | NM_001278618.1 | 0.00039 | + | 49196350 | 49197837 | 113,36, |
| peak24244 | 1.96 | 0.000234423 | 0.002884032 | 2.39 | 1.58489E-05 | 8.12831E-05 | chr20 | 49199315 | 49199415 | NM_001278618.1 | 0.000016 | + | 49199315 | 49199415 | 100, |
| peak24246 | 1.35 | 0.000831764 | 0.00724436 | 11.4 | 1.07152E-07 | 1.04713E-06 | chr20 | 49576098 | 49576596 | NM_014484.5 | 0.00000011 | + | 49576098 | 49576596 | 498, |
| peak24291 | 1.29 | 0.003548134 | 0.020417379 | 6.33 | 0.000251189 | 0.004570882 | chr20 | 60777272 | 60777623 | NM_015666.3 | 0.00025 | + | 60777272 | 60777623 | 351, |
| peak24293 | 4.34 | 2.23872E-07 | 1.38038E-05 | 3.63 | 0.00057544 | 0.002187762 | chr20 | 60835141 | 60839767 | NM_001363878.1 | 0.00058 | + | 60835141 | 60839767 | 40,76,83, |
| peak24297 | 5.99 | 2.0893E-05 | 0.000512861 | 1.38 | 0.000724436 | 0.002691535 | chr20 | 60835179 | 60847203 | NM_014835.4 | 0.00072 | + | 60835179 | 60847203 | 2,76,23, |
| peak24318 | 5.01 | 0.000977237 | 0.008128305 | 3.25 | 0.000223872 | 0.004168694 | chr20 | 62730705 | 62730903 | NM_000913.6 | 0.00022 | + | 62730705 | 62730903 | 198, |
| peak24320 | 5.01 | 0.000977237 | 0.008128305 | 3.25 | 0.000269153 | 0.004897788 | chr20 | 62730703 | 62730902 | NM_001318853.2 | 0.00027 | + | 62730703 | 62730902 | 199, |
| peak24322 | 5.01 | 0.000977237 | 0.008128305 | 3.25 | 0.000269153 | 0.004897788 | chr20 | 62730703 | 62730902 | NM_182647.4 | 0.00027 | + | 62730703 | 62730902 | 199, |
| peak24324 | 5.07 | 0.000524807 | 0.005128614 | 2.67 | 0.000346737 | 0.00616595 | chr20 | 62730706 | 62730906 | NM_001318854.1 | 0.00035 | + | 62730706 | 62730906 | 200, |
| peak24326 | 5.04 | 0.000758578 | 0.00676083 | 2.67 | 0.000131826 | 0.002570396 | chr20 | 62730709 | 62730908 | NM_001318855.1 | 0.00013 | + | 62730709 | 62730908 | 199, |
| peak24491 | 1.02 | 1.44544E-05 | 0.000380189 | 33.8 | 3.16228E-11 | 9.54993E-10 | chr20 | 35412384 | 35413682 | NM_080627.4 | 3.2E-11 | - | 35412384 | 35413682 | 1298, |
| peak24499 | 3.08 | 7.07946E-07 | 3.71535E-05 | 7.19 | 1.23027E-05 | 6.45654E-05 | chr20 | 36146013 | 36146161 | NM_001317075.1 | 0.000012 | - | 36146013 | 36146161 | 148, |
| peak24500 | 2.99 | 1.54882E-06 | 6.76083E-05 | 7.28 | 4.16869E-06 | 2.51189E-05 | chr20 | 36146017 | 36146166 | NM_001317074.1 | 0.0000042 | - | 36146017 | 36146166 | 149, |
| peak24501 | 3.02 | 1.34896E-06 | 6.0256E-05 | 7.06 | 6.0256E-06 | 3.46737E-05 | chr20 | 36146014 | 36146162 | NM_001167823.2 | 0.000006 | - | 36146014 | 36146162 | 148, |
| peak24502 | 2.99 | 1.54882E-06 | 6.76083E-05 | 7.28 | 4.16869E-06 | 2.51189E-05 | chr20 | 36146017 | 36146166 | NM_001167821.1 | 0.0000042 | - | 36146017 | 36146166 | 149, |
| peak24503 | 2.99 | 1.94984E-06 | 7.94328E-05 | 7.28 | 1.02329E-05 | 5.49541E-05 | chr20 | 36146017 | 36146168 | NM_001167822.2 | 0.00001 | - | 36146017 | 36146168 | 151, |
| peak24504 | 3.01 | 1.31826E-06 | 6.0256E-05 | 7.34 | 3.23594E-06 | 1.99526E-05 | chr20 | 36146015 | 36146163 | NM_006698.4 | 0.0000032 | - | 36146015 | 36146163 | 148, |
| peak24505 | 3.02 | 1.34896E-06 | 6.0256E-05 | 7.06 | 1.47911E-06 | 0.00001 | chr20 | 36146014 | 36146162 | NM_001167820.1 | 0.0000015 | - | 36146014 | 36146162 | 148, |
| peak24506 | 1.24 | 0.000338844 | 0.003801894 | 19.3 | 0.000154882 | 0.000645654 | chr20 | 36640112 | 36640713 | NM_001303457.2 | 0.00015 | - | 36640112 | 36640713 | 601, |
| peak24508 | 1.29 | 0.000380189 | 0.004168694 | 25.1 | 0.000162181 | 0.000676083 | chr20 | 36640108 | 36640707 | NM_014657.3 | 0.00016 | - | 36640108 | 36640707 | 599, |
| peak24526 | 1.21 | 0.000295121 | 0.003388442 | 9.86 | 6.16595E-09 | 9.12011E-08 | chr20 | 44699072 | 44718502 | NM_001348151.2 | 6.2E-09 | - | 44699072 | 44718502 | 103,47, |
| peak24625 | 1.15 | 0.000489779 | 0.004897788 | 11.7 | 2.51189E-07 | 2.13796E-06 | chr20 | 49509437 | 49510135 | NM_001282531.3 | 0.00000025 | - | 49509437 | 49510135 | 698, |
| peak24628 | 1.06 | 3.98107E-05 | 0.000812831 | 10.7 | 1.7378E-07 | 1.58489E-06 | chr20 | 49509452 | 49510152 | NM_001347511.2 | 0.00000017 | - | 49509452 | 49510152 | 700, |
| peak24631 | 1.19 | 3.71535E-05 | 0.000776247 | 10 | 1.54882E-07 | 1.41254E-06 | chr20 | 49509446 | 49510146 | NM_015339.5 | 0.00000015 | - | 49509446 | 49510146 | 700, |
| peak24636 | 1.01 | 7.58578E-05 | 0.00128825 | 13.3 | 3.46737E-08 | 3.98107E-07 | chr20 | 49509480 | 49510127 | NM_181442.4 | 0.000000035 | - | 49509480 | 49510127 | 647, |
| peak24641 | 2.47 | 0.004570882 | 0.024547089 | 3.33 | 1.90546E-08 | 2.34423E-07 | chr20 | 50048695 | 50048845 | NM_001258296.2 | 0.000000019 | - | 50048695 | 50048845 | 150, |
| peak24648 | 2.57 | 0.003090295 | 0.018620871 | 2.11 | 1.69824E-08 | 2.13796E-07 | chr20 | 50048706 | 50048807 | NM_012340.5 | 0.000000017 | - | 50048706 | 50048807 | 101, |
| peak24650 | 3.07 | 0.000147911 | 0.002089296 | 4.57 | 1.69824E-08 | 2.13796E-07 | chr20 | 50140301 | 50140501 | NM_012340.5 | 0.000000017 | - | 50140301 | 50140501 | 200, |
| peak24655 | 2.92 | 0.000144544 | 0.002041738 | 3.75 | 0.000562341 | 0.002137962 | chr20 | 50140300 | 50140500 | NM_173091.4 | 0.00056 | - | 50140300 | 50140500 | 200, |

| | | | | | | | | | | | | | | | |
|---|---|---|---|---|---|---|---|---|---|---|---|---|---|---|---|
| peak24664 | 3.84 | 0.000218776 | 0.002754229 | 3.7 | 0.000199526 | 0.000812831 | chr20 | 50140331 | 50140581 | NM_001258292.2 | 0.0002 | - | 50140331 | 50140581 | 250, |
| peak24678 | 2.45 | 0.004570882 | 0.024547089 | 11.7 | 0.004570882 | 0.015488166 | chr20 | 50769390 | 50769490 | NM_199426.2 | 0.0046 | - | 50769390 | 50769490 | 100, |
| peak24687 | 1.42 | 0.00018197 | 0.002398833 | 3.12 | 1.58489E-05 | 0.000416869 | chr20 | 54945230 | 54945378 | NM_003600.4 | 0.000016 | - | 54945230 | 54945378 | 148, |
| peak24688 | 1.39 | 5.62341E-06 | 0.000186209 | 3.5 | 3.71535E-05 | 0.000870964 | chr20 | 54945037 | 54945383 | NM_198433.3 | 0.000037 | - | 54945037 | 54945383 | 346, |
| peak24694 | 1.01 | 8.51138E-05 | 0.001380384 | 5.73 | 1E-11 | 3.71535E-10 | chr20 | 61538582 | 61541371 | NM_033081.2 | 1E-11 | - | 61538582 | 61541371 | 129,321, |
| peak24697 | 3.15 | 0.002187762 | 0.014454398 | 6.17 | 7.94328E-11 | 2.39883E-09 | chr20 | 61512934 | 61513384 | NM_001193369.2 | 7.9E-11 | - | 61512934 | 61513384 | 450, |
| peak24704 | 1.09 | 2.81838E-06 | 0.000107152 | 6.08 | 4.0738E-06 | 2.45471E-05 | chr20 | 61528344 | 61541370 | NM_080797.4 | 0.0000041 | - | 61528344 | 61541370 | 4,214,213,320, |
| peak24706 | 1.02 | 0.001737801 | 0.012302688 | 16.5 | 1.12202E-09 | 2.13796E-08 | chr20 | 61537098 | 61537398 | NM_080796.3 | 1.1E-09 | - | 61537098 | 61537398 | 300, |
| peak24710 | 1.13 | 0.000416869 | 0.004466836 | 6.44 | 0.00616595 | 0.020892961 | chr20 | 61538538 | 61541325 | NM_022105.4 | 0.0062 | - | 61538538 | 61541325 | 173,275, |
| peak24719 | 1.6 | 0.000630957 | 0.005888437 | 2.18 | 1.09648E-05 | 5.88844E-05 | chr20 | 62595678 | 62595976 | NM_020713.3 | 0.000011 | - | 62595678 | 62595976 | 66,134, |
| peak24724 | 1.53 | 0.002041738 | 0.013803843 | 2.29 | 0.001513561 | 0.005370318 | chr21 | 18937784 | 18937935 | NM_001207063.2 | 0.0015 | + | 18937784 | 18937935 | 151, |
| peak24733 | 3.23 | 0.001584893 | 0.011481536 | 17 | 0.000630957 | 0.002398833 | chr21 | 30415842 | 30419458 | NM_001032410.1 | 0.00063 | + | 30415842 | 30419458 | 78,471, |
| peak24734 | 3.92 | 0.000794328 | 0.00691831 | 2.38 | 0.001584893 | 0.023988329 | chr21 | 30415850 | 30419066 | NM_006447.3 | 0.0016 | + | 30415850 | 30419066 | 70,79, |
| peak24830 | 1.44 | 0.002089296 | 0.014125375 | 77.1 | 4.36516E-09 | 6.76083E-08 | chr21 | 38884344 | 38884892 | NM_001396.4 | 4.4E-09 | + | 38884344 | 38884892 | 548, |
| peak24881 | 2.75 | 1.20226E-05 | 0.000338844 | 8 | 3.16228E-13 | 1.99526E-11 | chr21 | 9908312 | 9968545 | NR_038327.2 | 3.2E-13 | - | 9908312 | 9968545 | 120,231,59,30, |
| peak24883 | 1.7 | 4.2658E-07 | 2.39883E-05 | 36.5 | 6.91831E-05 | 0.00030903 | chr21 | 16337082 | 16338027 | NR_003489.4 | 0.000069 | - | 16337082 | 16338027 | 945, |
| peak24911 | 5.92 | 0.000001 | 4.7863E-05 | 7.5 | 0.000281838 | 0.005128614 | chr21 | 32492674 | 32492974 | NM_001353694.1 | 0.00028 | - | 32492674 | 32492974 | 300, |
| peak24916 | 5.41 | 5.24807E-06 | 0.000177828 | 8.5 | 0.002089296 | 0.030902954 | chr21 | 32492678 | 32492928 | NM_001353689.1 | 0.0021 | - | 32492678 | 32492928 | 250, |
| peak24919 | 5.88 | 1.41254E-06 | 6.16595E-05 | 7.5 | 0.000245471 | 0.004466836 | chr21 | 32492673 | 32492972 | NM_001353690.1 | 0.00025 | - | 32492673 | 32492972 | 299, |
| peak24922 | 5.88 | 1.41254E-06 | 6.16595E-05 | 7.5 | 0.000218776 | 0.004073803 | chr21 | 32492673 | 32492972 | NM_001353691.1 | 0.00022 | - | 32492673 | 32492972 | 299, |
| peak24929 | 5.92 | 0.000001 | 4.7863E-05 | 7.5 | 0.000269153 | 0.004897788 | chr21 | 32492674 | 32492973 | NM_003253.3 | 0.00027 | - | 32492674 | 32492973 | 299, |
| peak24981 | 5.02 | 0.000162181 | 0.002187762 | 4.65 | 1.07152E-05 | 5.7544E-05 | chr21 | 43412800 | 43412950 | NM_001098402.2 | 0.000011 | - | 43412800 | 43412950 | 150, |
| peak24986 | 5.21 | 0.000186209 | 0.002454709 | 3.82 | 6.91831E-07 | 5.12861E-06 | chr21 | 43412790 | 43412941 | NM_001098403.2 | 0.00000069 | - | 43412790 | 43412941 | 151, |
| peak24991 | 5.29 | 0.000630957 | 0.006025596 | 3.09 | 4.7863E-06 | 2.81838E-05 | chr21 | 43412829 | 43412930 | NM_001320729.2 | 0.0000048 | - | 43412829 | 43412930 | 101, |
| peak24998 | 4.97 | 0.000229087 | 0.002818383 | 3.69 | 3.31131E-05 | 0.000158489 | chr21 | 43412804 | 43412954 | NM_001320731.2 | 0.000033 | - | 43412804 | 43412954 | 150, |
| peak25004 | 4.99 | 0.004677351 | 0.024547089 | 3.64 | 4.67735E-10 | 1.02329E-08 | chr21 | 43412828 | 43412929 | NM_020727.5 | 4.7E-10 | - | 43412828 | 43412929 | 101, |
| peak25011 | 2.77 | 9.12011E-05 | 0.001479108 | 14.9 | 9.33254E-06 | 5.12861E-05 | chr21 | 45647407 | 45647806 | NM_001283052.2 | 0.0000093 | - | 45647407 | 45647806 | 399, |
| peak25012 | 1.84 | 0.001 | 0.008317638 | 3.68 | 0.000218776 | 0.000891251 | chr21 | 45648004 | 45648105 | NM_001283052.2 | 0.00022 | - | 45648004 | 45648105 | 101, |
| peak25015 | 3 | 0.000151356 | 0.002089296 | 13.8 | 4.16869E-05 | 0.000194984 | chr21 | 45647400 | 45647799 | NM_001365759.2 | 0.000042 | - | 45647400 | 45647799 | 399, |
| peak25016 | 1.77 | 0.003890451 | 0.021877616 | 4 | 0.000691831 | 0.002570396 | chr21 | 45647997 | 45648098 | NM_001365759.2 | 0.00069 | - | 45647997 | 45648098 | 101, |
| peak25023 | 1.79 | 1.31826E-05 | 0.000354813 | 5.5 | 0.000316228 | 0.001230269 | chr21 | 47743367 | 47743714 | NM_001286476.1 | 0.00032 | - | 47743367 | 47743714 | 347, |
| peak25051 | 3.39 | 7.94328E-05 | 0.001318257 | 12.4 | 5.01187E-06 | 2.88403E-05 | chr22 | 20755644 | 20760098 | NR_046282.2 | 0.000005 | + | 20755644 | 20760098 | 18,432, |
| peak25061 | 1.51 | 0.003162278 | 0.018620871 | 5.33 | 0.00162181 | 0.005754399 | chr22 | 21288525 | 21304391 | NR_005207.4 | 0.0016 | + | 21288525 | 21304391 | 7,393, |
| peak25078 | 1.44 | 3.98107E-09 | 3.46737E-07 | 27.4 | 3.71535E-07 | 3.01995E-06 | chr22 | 22051219 | 22052016 | NM_148175.3 | 0.00000037 | + | 22051219 | 22052016 | 797, |
| peak25087 | 2.85 | 0.002454709 | 0.015848932 | 10.1 | 2.69153E-10 | 6.30957E-09 | chr22 | 23656198 | 23657693 | NM_021574.3 | 2.7E-10 | + | 23656198 | 23657693 | 62,163,74, |
| peak25129 | 1.91 | 0.002344229 | 0.015135612 | 3.8 | 0.000776247 | 0.012589254 | chr22 | 29179625 | 29182317 | NM_001284264.1 | 0.00078 | + | 29179625 | 29182317 | 8,241, |
| peak25130 | 1.73 | 0.002818383 | 0.017378008 | 3.8 | 0.000912011 | 0.014454398 | chr22 | 29179520 | 29182311 | NM_173510.4 | 0.00091 | + | 29179520 | 29182311 | 113,235, |
| peak25131 | 1.83 | 0.001023293 | 0.00851138 | 3.8 | 5.7544E-05 | 0.001258925 | chr22 | 29179525 | 29182316 | NM_001284265.1 | 0.000058 | + | 29179525 | 29182316 | 108,240, |
| peak25132 | 3.4 | 3.63078E-05 | 0.000776247 | 13.8 | 1.65959E-09 | 2.95121E-08 | chr22 | 29445222 | 29445969 | NM_032173.3 | 1.7E-09 | + | 29445222 | 29445969 | 747, |
| peak25143 | 1.4 | 0.001479108 | 0.010964782 | 7 | 0.003467369 | 0.011748976 | chr22 | 30061001 | 30069369 | NM_181825.3 | 0.0035 | + | 30061001 | 30069369 | 52,114,123,112, |
| peak25232 | 2.68 | 4.89779E-06 | 0.000165959 | 6.56 | 3.01995E-05 | 0.000144544 | chr22 | 35660647 | 35661198 | NM_001003681.3 | 0.00003 | + | 35660647 | 35661198 | 551, |
| peak25235 | 2.74 | 1.02329E-06 | 4.89779E-05 | 6.22 | 2.45471E-07 | 2.0893E-06 | chr22 | 35660647 | 35661197 | NM_001362972.2 | 0.00000025 | + | 35660647 | 35661197 | 550, |
| peak25238 | 2.46 | 0.000954993 | 0.007943282 | 6.5 | 4.7863E-06 | 2.81838E-05 | chr22 | 35660777 | 35661174 | NR_027780.2 | 0.0000048 | + | 35660777 | 35661174 | 397, |
| peak25243 | 1.94 | 4.7863E-08 | 3.46737E-06 | 26.5 | 0.00030903 | 0.001230269 | chr22 | 36661216 | 36661758 | NM_001136540.1 | 0.00031 | + | 36661216 | 36661758 | 542, |
| peak25244 | 1.8 | 0.00040738 | 0.004365158 | 30.6 | 0.004786301 | 0.016218101 | chr22 | 36657734 | 36661804 | NM_001136541.1 | 0.0048 | + | 36657734 | 36661804 | 34,608, |
| peak25274 | 3.9 | 2.13796E-05 | 0.000524807 | 6.43 | 1.65959E-10 | 4.16869E-09 | chr22 | 41277808 | 41282343 | NM_022098.4 | 1.7E-10 | + | 41277808 | 41282343 | 373,27, |
| peak25293 | 2.03 | 0.000323594 | 0.003630781 | 22.2 | 8.91251E-07 | 6.60693E-06 | chr22 | 45581124 | 45581422 | NM_007172.4 | 0.00000089 | + | 45581124 | 45581422 | 298, |
| peak25295 | 2.15 | 5.01187E-06 | 0.000169824 | 27.2 | 1.54882E-06 | 1.07152E-05 | chr22 | 45581118 | 45581466 | NM_153645.2 | 0.0000015 | + | 45581118 | 45581466 | 348, |
| peak25302 | 2.33 | 0.000616595 | 0.005888437 | 4.91 | 0.012022644 | 0.038904514 | chr22 | 47074981 | 47075082 | NM_015124.5 | 0.012 | + | 47074981 | 47075082 | 101, |
| peak25318 | 2.24 | 0.00017378 | 0.002344229 | 76.6 | 7.07946E-06 | 3.98107E-05 | chr22 | 19025827 | 19026422 | NM_001173534.2 | 0.0000071 | - | 19025827 | 19026422 | 595, |
| peak25320 | 2.05 | 4.16869E-05 | 0.000851138 | 51.9 | 6.76083E-07 | 5.01187E-06 | chr22 | 19025842 | 19026441 | NM_005137.3 | 0.00000068 | - | 19025842 | 19026441 | 599, |
| peak25325 | 2.94 | 0.002691535 | 0.016595869 | 2.57 | 7.24436E-08 | 4.2658E-06 | chr22 | 19127500 | 19130255 | NM_022719.3 | 0.000000072 | - | 19127500 | 19130255 | 37,96,17, |
| peak25328 | 2.92 | 0.002754229 | 0.016982437 | 2.33 | 4.2658E-05 | 0.000977237 | chr22 | 19127501 | 19130254 | NR_134304.2 | 0.000043 | - | 19127501 | 19130254 | 39,96,16, |
| peak25416 | 1.33 | 0.004786301 | 0.025118864 | 23.8 | 1.14815E-05 | 6.0256E-05 | chr22 | 31740833 | 31741134 | NM_014323.3 | 0.000011 | - | 31740833 | 31741134 | 301, |
| peak25436 | 1.81 | 1.94984E-08 | 1.51356E-06 | 14.6 | 5.88844E-05 | 0.000269153 | chr22 | 32112567 | 32134488 | NM_173566.3 | 0.000059 | - | 32112567 | 32134488 | 710,73,116, |
| peak25494 | 3.06 | 1.54882E-07 | 0.00001 | 17.3 | 0.00011749 | 0.000501187 | chr22 | 37578249 | 37578649 | NM_031910.4 | 0.00012 | - | 37578249 | 37578649 | 400, |
| peak25583 | 3.87 | 0.000512861 | 0.005128614 | 9.17 | 1.12202E-05 | 5.88844E-05 | chrX | 9686530 | 9686731 | NM_001139466.1 | 0.000011 | + | 9686530 | 9686731 | 201, |

| | | | | | | | | | | | | | | | |
|---|---|---|---|---|---|---|---|---|---|---|---|---|---|---|---|
| peak25646 | 3.95 | 3.01995E-05 | 0.000676083 | 4.2 | 3.98107E-06 | 2.39883E-05 | chrX | 24557260 | 24557560 | NM_001142386.3 | 0.000004 | + | 24557260 | 24557560 | 300, |
| peak25660 | 3.52 | 0.002089296 | 0.014125375 | 3.05 | 0.006309573 | 0.020892961 | chrX | 41193464 | 41196674 | NM_001193417.3 | 0.0063 | + | 41193464 | 41196674 | 86,14, |
| peak25662 | 2.93 | 5.01187E-05 | 0.000954993 | 4.62 | 4.36516E-07 | 3.38844E-06 | chrX | 44921903 | 44922974 | NM_001291416.1 | 0.00000044 | + | 44921903 | 44922974 | 90,308, |
| peak25663 | 2.27 | 7.4131E-06 | 0.000229087 | 19.2 | 4.36516E-07 | 3.38844E-06 | chrX | 44928834 | 44929529 | NM_001291416.1 | 0.00000044 | + | 44928834 | 44929529 | 695, |
| peak25664 | 2.2 | 3.31131E-06 | 0.000123027 | 18.7 | 2.34423E-07 | 2.04174E-06 | chrX | 44920646 | 44929559 | NM_001291418.1 | 0.00000023 | + | 44920646 | 44929559 | 18,396,736, |
| peak25666 | 2.15 | 1.86209E-06 | 7.76247E-05 | 20.5 | 3.89045E-07 | 3.16228E-06 | chrX | 44922960 | 44929568 | NM_001291421.1 | 0.00000039 | + | 44922960 | 44929568 | 102,745, |
| peak25668 | 2.88 | 1.99526E-07 | 1.25893E-05 | 8.83 | 3.38844E-07 | 2.75423E-06 | chrX | 44922706 | 44929166 | NM_021140.3 | 0.00000034 | + | 44922706 | 44929166 | 356,343, |
| peak25670 | 1.99 | 0.000707946 | 0.006456542 | 3.27 | 2.45471E-07 | 2.0893E-06 | chrX | 44922683 | 44922983 | NR_111960.1 | 0.00000025 | + | 44922683 | 44922983 | 300, |
| peak25671 | 2.07 | 0.000234423 | 0.002818383 | 22.7 | 2.45471E-07 | 2.0893E-06 | chrX | 44928842 | 44929539 | NR_111960.1 | 0.00000025 | + | 44928842 | 44929539 | 697, |
| peak25676 | 2.84 | 3.38844E-05 | 0.000724436 | 18.3 | 7.58578E-06 | 4.16869E-05 | chrX | 57620614 | 57620865 | NM_007157.3 | 0.0000076 | + | 57620614 | 57620865 | 251, |
| peak25683 | 3.11 | 0.001778279 | 0.012589254 | 23.2 | 0.00042658 | 0.00162181 | chrX | 66945376 | 66945527 | NM_000044.6 | 0.00043 | + | 66945376 | 66945527 | 151, |
| peak25685 | 3.95 | 0.000371535 | 0.003981072 | 9.18 | 0.009332543 | 0.030199517 | chrX | 66948716 | 66948866 | NM_000044.6 | 0.0093 | + | 66948716 | 66948866 | 150, |
| peak25688 | 2.97 | 0.000630957 | 0.006025596 | 27.3 | 0.000416869 | 0.001584893 | chrX | 66945384 | 66945535 | NM_001011645.3 | 0.00042 | + | 66945384 | 66945535 | 151, |
| peak25690 | 3.77 | 0.000194984 | 0.002511886 | 9.24 | 0.002187762 | 0.007585776 | chrX | 66948718 | 66948869 | NM_001011645.3 | 0.0022 | + | 66948718 | 66948869 | 151, |
| peak25748 | 4.02 | 1.8197E-05 | 0.000457088 | 3.5 | 0.00057544 | 0.002187762 | chrX | 123019934 | 123020134 | NM_001204401.1 | 0.00058 | + | 123019934 | 123020134 | 200, |
| peak25749 | 3.71 | 2.51189E-05 | 0.000588844 | 4.42 | 0.001230269 | 0.004466836 | chrX | 123019949 | 123020100 | NM_001167.3 | 0.0012 | + | 123019949 | 123020100 | 151, |
| peak25752 | 2.83 | 0.000147911 | 0.002089296 | 16.4 | 3.31131E-05 | 0.000158489 | chrX | 129147004 | 129147405 | NM_001184772.2 | 0.000033 | + | 129147004 | 129147405 | 401, |
| peak25753 | 1.27 | 0.002754229 | 0.017378008 | 8.29 | 0.014454398 | 0.046773514 | chrX | 129150053 | 129154974 | NM_001184772.2 | 0.014 | + | 129150053 | 129154974 | 136,15, |
| peak25755 | 2.83 | 0.000147911 | 0.002089296 | 16.4 | 0.000141254 | 0.000588844 | chrX | 129147003 | 129147403 | NM_021946.4 | 0.00014 | + | 129147003 | 129147403 | 400, |
| peak25764 | 1.16 | 1.1749E-06 | 5.49541E-05 | 12.4 | 1.20226E-08 | 1.58489E-07 | chrX | 3227849 | 3227999 | NM_015419.4 | 0.000000012 | - | 3227849 | 3227999 | 150, |
| peak25815 | 3.13 | 0.002818383 | 0.017378008 | 3.69 | 0.000398107 | 0.001513561 | chrX | 39922137 | 39922238 | NM_001123383.1 | 0.0004 | - | 39922137 | 39922238 | 101, |
| peak25818 | 2.34 | 1.8197E-05 | 0.000457088 | 6.91 | 5.24807E-05 | 0.000239883 | chrX | 39934011 | 39934262 | NM_001123383.1 | 0.000052 | - | 39934011 | 39934262 | 251, |
| peak25821 | 2.68 | 5.01187E-07 | 2.69153E-05 | 5.95 | 0.006456542 | 0.021379621 | chrX | 39934039 | 39934239 | NM_001123385.2 | 0.0065 | - | 39934039 | 39934239 | 200, |
| peak25871 | 3.17 | 0.000275423 | 0.003235937 | 12.5 | 5.24807E-05 | 0.000239883 | chrX | 63410219 | 63410518 | NM_152424.4 | 0.000052 | - | 63410219 | 63410518 | 299, |
| peak25872 | 4.27 | 3.54813E-06 | 0.000128825 | 13.8 | 5.24807E-05 | 0.000239883 | chrX | 63411164 | 63411413 | NM_152424.4 | 0.000052 | - | 63411164 | 63411413 | 249, |
| peak25901 | 4.26 | 1.41254E-06 | 6.30957E-05 | 18.2 | 3.63078E-09 | 5.7544E-08 | chrX | 79932534 | 79932734 | NM_153252.5 | 3.6E-09 | - | 79932534 | 79932734 | 200, |
| peak25971 | 3.56 | 0.00057544 | 0.005495409 | 14.2 | 2.45471E-07 | 2.0893E-06 | chrX | 117043664 | 117043964 | NM_001168302.1 | 0.00000025 | - | 117043664 | 117043964 | 300, |

Legends: Specific sites of 1850 up regulated genes of breast cancer cells, p-values <0.05 were indicated.

Supplementary Table 3. The relationship between METTL3 and patient clinicopathological features.

| Cytosol expression | | | | | | Nucleus expression | | | | | |
|---|---|---|---|---|---|---|---|---|---|---|---|
| | variables | METTL3 expression low | METTL3 expression high | total | $\chi^2$ | p value | variables | METTL3 expression low | METTL3 expression high | total | $\chi^2$ | p value |
| Age (year) | | | | | 7.973 | 0.005 | | | | | 4.476 | 0.034 |
| | ≤54 | 42 | 25 | 67 | | | ≤54 | 40 | 27 | 67 | | |
| | >54 | 23 | 38 | 61 | | | >54 | 25 | 36 | 61 | | |
| Grade | | | | | 2.095 | 0.148 | | | | | 0.097 | 0.756 |
| | II | 48 | 39 | 87 | | | II | 45 | 42 | 87 | | |
| | III | 17 | 24 | 41 | | | III | 20 | 21 | 41 | | |
| T stage | | | | | 2.012 | 0.156 | | | | | 1.106 | 0.293 |
| | T1 | 20 | 27 | 47 | | | T1 | 21 | 26 | 47 | | |
| | T2/T3 | 45 | 36 | 81 | | | T2/T3 | 44 | 37 | 81 | | |
| N stage | | | | | 0.181 | 0.671 | | | | | 1.115 | 0.291 |
| | N0 | 41 | 42 | 83 | | | N0 | 45 | 38 | 83 | | |
| | N1 | 24 | 21 | 45 | | | N1 | 20 | 25 | 45 | | |
| TNM stage | | | | | 0.181 | 0.671 | | | | | 1.115 | 0.291 |
| | I/II | 41 | 42 | 83 | | | I/II | 45 | 38 | 83 | | |
| | III | 24 | 21 | 45 | | | III | 20 | 25 | 45 | | |

Legend: The age of patients was significantly related to the expression of METTL3, p-values <0.05 were indicated.

Supplementary Table 4. KEGG analysis of MCF-7 between METTL3 KO group and control group.

| NAME | SIZE | ES | NES | NOM p-val | FDR q-val |
|---|---|---|---|---|---|
| OOCYTE_MEIOSIS | 117 | 0.5346071 | 1.9137065 | 0.000000000 | 0.005284764 |
| MRNA_SURVEILLANCE_PATHWAY | 84 | 0.5534028 | 1.9046290 | 0.000000000 | 0.002642382 |
| CELL_CYCLE | 124 | 0.5127093 | 1.8674089 | 0.000000000 | 0.003356671 |
| SPLICEOSOME | 132 | 0.4741051 | 1.7391235 | 0.001503759 | 0.018853806 |
| RNA_TRANSPORT | 154 | 0.4595019 | 1.7093091 | 0.000000000 | 0.021680197 |
| HERPES_SIMPLEX_VIRUS_1_INFECTION | 445 | 0.4113639 | 1.6958411 | 0.000000000 | 0.020975525 |
| RNA_DEGRADATION | 75 | 0.4849807 | 1.6435858 | 0.001555210 | 0.036382508 |
| GABAERGIC_SYNAPSE | 72 | 0.4761663 | 1.6059915 | 0.001589825 | 0.051397340 |
| HOMOLOGOUS_RECOMBINATION | 40 | 0.5132999 | 1.5605139 | 0.006722689 | 0.076921080 |
| HIPPO_SIGNALING_PATHWAY | 150 | 0.4178041 | 1.5406752 | 0.000000000 | 0.085010010 |
| UBIQUITIN_MEDIATED_PROTEOLYSIS | 134 | 0.4043445 | 1.4986324 | 0.007352941 | 0.118713150 |
| APELIN_SIGNALING_PATHWAY | 128 | 0.4029374 | 1.4699396 | 0.001466276 | 0.142974240 |
| TGF_BETA_SIGNALING_PATHWAY | 89 | 0.4186491 | 1.4527082 | 0.010903426 | 0.155976560 |
| VASCULAR_SMOOTH_MUSCLE_CONTRACTION | 116 | 0.4029590 | 1.4498663 | 0.004830918 | 0.148824350 |
| DOPAMINERGIC_SYNAPSE | 120 | 0.3980478 | 1.4371443 | 0.004538578 | 0.156639950 |
| HIPPO_SIGNALING_PATHWAY_MULTIPLE_SPECIES | 27 | 0.5142397 | 1.4324483 | 0.037037037 | 0.153369350 |
| RIBOSOME_BIOGENESIS_IN_EUKARYOTES | 94 | 0.4092717 | 1.4309617 | 0.009615385 | 0.146196280 |
| GASTRIC_ACID_SECRETION | 67 | 0.4297816 | 1.4207530 | 0.019969279 | 0.152647550 |
| BASAL_TRANSCRIPTION_FACTORS | 43 | 0.4624984 | 1.3942719 | 0.047540985 | 0.182233600 |
| DNA_REPLICATION | 35 | 0.4767786 | 1.3934253 | 0.056270096 | 0.174091710 |
| MISMATCH_REPAIR | 22 | 0.5160127 | 1.3896858 | 0.065404475 | 0.170953360 |
| FANCONI_ANEMIA_PATHWAY | 53 | 0.4390387 | 1.3818991 | 0.037953794 | 0.175050240 |
| OLFACTORY_TRANSDUCTION | 78 | 0.4044665 | 1.3640484 | 0.027067669 | 0.194316210 |
| SALIVARY_SECRETION | 72 | 0.4021712 | 1.3588563 | 0.044045676 | 0.194244400 |
| CIRCADIAN_ENTRAINMENT | 88 | 0.3877488 | 1.3430887 | 0.029363785 | 0.210896020 |
| PROGESTERONE_MEDIATED_OOCYTE_MATURATION | 92 | 0.3883264 | 1.3403295 | 0.046296295 | 0.207687240 |
| OXYTOCIN_SIGNALING_PATHWAY | 145 | 0.3549204 | 1.3207366 | 0.033923306 | 0.233246090 |
| SEROTONERGIC_SYNAPSE | 92 | 0.3824075 | 1.3182973 | 0.037854890 | 0.229258980 |
| NUCLEOTIDE_EXCISION_REPAIR | 45 | 0.4252379 | 1.3069832 | 0.079288030 | 0.242049900 |
| LONG_TERM_POTENTIATION | 59 | 0.4031246 | 1.2937704 | 0.095477390 | 0.259286050 |
| EPITHELIAL_CELL_SIGNALING_IN_HELICOBACTER_PYLORI_INFECTION | 66 | 0.3826618 | 1.2655542 | 0.098412700 | 0.310552750 |
| MORPHINE_ADDICTION | 74 | 0.3758763 | 1.2646908 | 0.091338580 | 0.302524200 |
| CHOLINERGIC_SYNAPSE | 104 | 0.3554510 | 1.2639638 | 0.075079870 | 0.295116340 |
| GNRH_SIGNALING_PATHWAY | 89 | 0.3582945 | 1.2501591 | 0.097222224 | 0.317240860 |
| GAP_JUNCTION | 79 | 0.3661117 | 1.2439343 | 0.124203820 | 0.321709070 |
| CELLULAR_SENESCENCE | 154 | 0.3311127 | 1.2389222 | 0.068513120 | 0.324681850 |
| PROTEASOME | 42 | 0.4020604 | 1.2266492 | 0.174917490 | 0.343749640 |
| RELAXIN_SIGNALING_PATHWAY | 120 | 0.3390088 | 1.2250588 | 0.102325580 | 0.338223130 |
| SMALL_CELL_LUNG_CANCER | 91 | 0.3512376 | 1.2193893 | 0.105100460 | 0.341905740 |

| | | | | | |
|---|---|---|---|---|---|
| LYSINE_DEGRADATION | 59 | 0.3734347 | 1.2173640 | 0.143084260 | 0.338183400 |
| MICRORNAS_IN_CANCER | 202 | 0.3165989 | 1.2169042 | 0.079528720 | 0.331035140 |
| CUSHING_SYNDROME | 142 | 0.3190008 | 1.1781247 | 0.126488090 | 0.415634190 |
| MUCIN_TYPE_O_GLYCAN_BIOSYNTHESIS | 29 | 0.4150059 | 1.1714602 | 0.200339560 | 0.423202540 |
| SIGNALING_PATHWAYS_REGULATING_PLURIPOTENCY_OF_STEM_CELLS | 124 | 0.3166348 | 1.1584489 | 0.175273870 | 0.449142220 |
| PHOTOTRANSDUCTION | 21 | 0.4407705 | 1.1526666 | 0.246329530 | 0.454842930 |
| MELANOMA | 63 | 0.3556762 | 1.1507057 | 0.210784320 | 0.450001960 |
| PATHWAYS_IN_CANCER | 482 | 0.2749186 | 1.1373020 | 0.093053736 | 0.478168550 |
| LINOLEIC_ACID_METABOLISM | 23 | 0.4189925 | 1.1365999 | 0.265758100 | 0.470292720 |
| CGMP_PKG_SIGNALING_PATHWAY | 153 | 0.3061270 | 1.1358678 | 0.181683900 | 0.462754880 |
| P53_SIGNALING_PATHWAY | 72 | 0.3403194 | 1.1328530 | 0.228384990 | 0.461586300 |
| ADHERENS_JUNCTION | 72 | 0.3422486 | 1.1318606 | 0.246489850 | 0.455262300 |
| HUMAN_T_CELL_LEUKEMIA_VIRUS_1_INFECTION | 210 | 0.2909505 | 1.1240133 | 0.176978420 | 0.466458980 |
| MELANOGENESIS | 94 | 0.3182994 | 1.1230234 | 0.224880380 | 0.460184160 |
| NICOTINE_ADDICTION | 23 | 0.4202755 | 1.1129996 | 0.288135600 | 0.478579820 |
| GLUTAMATERGIC_SYNAPSE | 99 | 0.3096806 | 1.1079668 | 0.223255810 | 0.482766630 |
| AMPHETAMINE_ADDICTION | 59 | 0.3443154 | 1.1024379 | 0.282009720 | 0.489098580 |
| LONG_TERM_DEPRESSION | 55 | 0.3384669 | 1.0998576 | 0.273015900 | 0.487326260 |
| FOCAL_ADHESION | 191 | 0.2865585 | 1.0974743 | 0.224606570 | 0.485625830 |
| PROTEIN_EXPORT | 23 | 0.4048240 | 1.0934819 | 0.318471340 | 0.488134300 |
| HEDGEHOG_SIGNALING_PATHWAY | 44 | 0.3585204 | 1.0902132 | 0.315424620 | 0.488575670 |
| WNT_SIGNALING_PATHWAY | 145 | 0.2948518 | 1.0883931 | 0.277862600 | 0.485167530 |
| PATHOGENIC_ESCHERICHIA_COLI_INFECTION | 54 | 0.3374427 | 1.0734826 | 0.312195120 | 0.515888450 |
| GASTRIC_CANCER | 134 | 0.2964991 | 1.0734042 | 0.300000000 | 0.507839860 |
| TIGHT_JUNCTION | 163 | 0.2859149 | 1.0617218 | 0.316100450 | 0.530555800 |
| AGE_RAGE_SIGNALING_PATHWAY_IN_DIABETIC_COMPLICATIONS | 100 | 0.3006987 | 1.0603343 | 0.336906580 | 0.526037600 |
| RENIN_SECRETION | 64 | 0.3175464 | 1.0479243 | 0.359424920 | 0.551186400 |
| SYNAPTIC_VESICLE_CYCLE | 67 | 0.3180729 | 1.0474786 | 0.356120820 | 0.544145300 |
| PI3K_AKT_SIGNALING_PATHWAY | 308 | 0.2580940 | 1.0343182 | 0.346206900 | 0.572140400 |
| PARATHYROID_HORMONE_SYNTHESIS,_SECRETION_AND_ACTION | 96 | 0.2993756 | 1.0341368 | 0.382581650 | 0.564352800 |
| THYROID_HORMONE_SIGNALING_PATHWAY | 111 | 0.2884254 | 1.0317439 | 0.377329200 | 0.562784600 |
| BREAST_CANCER | 135 | 0.2806279 | 1.0305302 | 0.378582200 | 0.558084300 |
| HEPATITIS_B | 149 | 0.2741688 | 1.0253376 | 0.393343420 | 0.564283300 |
| MTOR_SIGNALING_PATHWAY | 145 | 0.2772505 | 1.0237315 | 0.386567180 | 0.560946350 |
| COLORECTAL_CANCER | 85 | 0.2938270 | 1.0212166 | 0.405492720 | 0.559763700 |
| VASOPRESSIN_REGULATED_WATER_REABSORPTION | 41 | 0.3320863 | 1.0029157 | 0.477777780 | 0.601990640 |
| RETROGRADE_ENDOCANNABINOID_SIGNALING | 129 | 0.2729698 | 0.9953883 | 0.466565340 | 0.613724600 |
| KAPOSI_SARCOMA_ASSOCIATED_HERPESVIRUS_INFECTION | 168 | 0.2633826 | 0.9897852 | 0.474926260 | 0.621332200 |
| NOD_LIKE_RECEPTOR_SIGNALING_PATHWAY | 148 | 0.2645839 | 0.9767695 | 0.509984600 | 0.648490250 |
| PHOSPHATIDYLINOSITOL_SIGNALING_SYSTEM | 94 | 0.2740582 | 0.9552897 | 0.558461550 | 0.699325700 |
| GLUCAGON_SIGNALING_PATHWAY | 96 | 0.2724017 | 0.9534820 | 0.556082100 | 0.695676800 |

| | | | | | |
|---|---|---|---|---|---|
| CHRONIC_MYELOID_LEUKEMIA | 76 | 0.2811971 | 0.9515398 | 0.563779530 | 0.692440030 |
| TASTE_TRANSDUCTION | 49 | 0.2998638 | 0.9458617 | 0.545600000 | 0.699451800 |
| TRANSCRIPTIONAL_MISREGULATION_IN_CANCER | 171 | 0.2513842 | 0.9455702 | 0.601470600 | 0.691828550 |
| RNA_POLYMERASE | 31 | 0.3291008 | 0.9443388 | 0.551155100 | 0.686853300 |
| PANCREATIC_SECRETION | 74 | 0.2741840 | 0.9331481 | 0.612800000 | 0.708255800 |
| LONGEVITY_REGULATING_PATHWAY_MULTIPLE_SPECIES | 57 | 0.2900646 | 0.9296853 | 0.588996770 | 0.709028070 |
| BLADDER_CANCER | 40 | 0.3094515 | 0.9196600 | 0.576411960 | 0.727074440 |
| CORTISOL_SYNTHESIS_AND_SECRETION | 57 | 0.2722303 | 0.8901959 | 0.672727300 | 0.792753100 |
| PROSTATE_CANCER | 94 | 0.2551715 | 0.8877878 | 0.716440440 | 0.789769650 |
| LONGEVITY_REGULATING_PATHWAY | 82 | 0.2603190 | 0.8847933 | 0.694400000 | 0.788567500 |
| ADRENERGIC_SIGNALING_IN_CARDIOMYOCYTES | 136 | 0.2403438 | 0.8827432 | 0.746031760 | 0.784949900 |
| COLLECTING_DUCT_ACID_SECRETION | 24 | 0.3164214 | 0.8565510 | 0.683219200 | 0.837856100 |
| BACTERIAL_INVASION_OF_EPITHELIAL_CELLS | 74 | 0.2500622 | 0.8358663 | 0.800623060 | 0.873645540 |
| FOLATE_BIOSYNTHESIS | 24 | 0.3036263 | 0.8111425 | 0.716666640 | 0.912118000 |
| CIRCADIAN_RHYTHM | 31 | 0.2771940 | 0.8044128 | 0.787269700 | 0.913871050 |
| COCAINE_ADDICTION | 41 | 0.2615469 | 0.7899913 | 0.812709030 | 0.927762570 |
| PROTEIN_DIGESTION_AND_ABSORPTION | 76 | 0.2187850 | 0.7374296 | 0.953125000 | 0.980386140 |
| SHIGELLOSIS | 65 | 0.2260721 | 0.7346541 | 0.954113900 | 0.972784600 |
| GLYCOSYLPHOSPHATIDYLINOSITOL_(GPI)_ANCHOR_BIOSYNTHESIS | 25 | 0.2551617 | 0.7009829 | 0.906303200 | 0.986277940 |
| RHEUMATOID_ARTHRITIS | 80 | 0.2075296 | 0.6987315 | 0.970920860 | 0.977609800 |
| TERPENOID_BACKBONE_BIOSYNTHESIS | 21 | 0.2683342 | 0.6853379 | 0.911262800 | 0.974261800 |
| METABOLISM_OF_XENOBIOTICS_BY_CYTOCHROME_P450 | 46 | -0.7037460 | -2.3227816 | 0.000000000 | 0.000000000 |
| CHEMICAL_CARCINOGENESIS | 52 | -0.6348975 | -2.1284776 | 0.000000000 | 0.000000000 |
| ANTIGEN_PROCESSING_AND_PRESENTATION | 60 | -0.6133799 | -2.0852156 | 0.000000000 | 0.000000000 |
| ARGININE_AND_PROLINE_METABOLISM | 44 | -0.6242090 | -2.0294104 | 0.000000000 | 0.000000000 |
| PORPHYRIN_AND_CHLOROPHYLL_METABOLISM | 25 | -0.6887931 | -1.9743640 | 0.000000000 | 0.001801736 |
| PENTOSE_PHOSPHATE_PATHWAY | 29 | -0.6566440 | -1.9725279 | 0.000000000 | 0.001501447 |
| GRAFT_VERSUS_HOST_DISEASE | 30 | -0.6484959 | -1.9380019 | 0.000000000 | 0.002802064 |
| GALACTOSE_METABOLISM | 26 | -0.6628082 | -1.9243160 | 0.000000000 | 0.002657091 |
| AUTOIMMUNE_THYROID_DISEASE | 32 | -0.6233491 | -1.9170128 | 0.000000000 | 0.002968264 |
| FAT_DIGESTION_AND_ABSORPTION | 27 | -0.6450065 | -1.9000487 | 0.000000000 | 0.003535357 |
| STAPHYLOCOCCUS_AUREUS_INFECTION | 56 | -0.5578943 | -1.8988348 | 0.000000000 | 0.003213961 |
| ALLOGRAFT_REJECTION | 28 | -0.6487026 | -1.8930165 | 0.000000000 | 0.003254676 |
| COMPLEMENT_AND_COAGULATION_CASCADES | 72 | -0.5278851 | -1.8753360 | 0.000000000 | 0.003265762 |
| NITROGEN_METABOLISM | 16 | -0.7200358 | -1.8680484 | 0.000000000 | 0.003284841 |
| FRUCTOSE_AND_MANNOSE_METABOLISM | 33 | -0.6185392 | -1.8579480 | 0.000000000 | 0.003927829 |
| CARBOHYDRATE_DIGESTION_AND_ABSORPTION | 34 | -0.6014708 | -1.8469299 | 0.000000000 | 0.004042661 |
| TYPE_I_DIABETES_MELLITUS | 34 | -0.5936338 | -1.8232661 | 0.000000000 | 0.005651953 |
| GLYCOLYSIS_/_GLUCONEOGENESIS | 62 | -0.5241401 | -1.8133312 | 0.000000000 | 0.005937023 |
| STEROID_HORMONE_BIOSYNTHESIS | 37 | -0.5759194 | -1.8017946 | 0.002564103 | 0.007235488 |
| ARGININE_BIOSYNTHESIS | 17 | -0.6874320 | -1.7936444 | 0.000000000 | 0.007413429 |

| | | | | | |
|---|---|---|---|---|---|
| ARACHIDONIC_ACID_METABOLISM | 52 | -0.5034511 | -1.7003648 | 0.000000000 | 0.022118751 |
| DRUG_METABOLISM_CYTOCHROME_P450 | 44 | -0.5167210 | -1.6865586 | 0.004975124 | 0.023487797 |
| STEROID_BIOSYNTHESIS | 19 | -0.6349593 | -1.6849338 | 0.014851485 | 0.023117764 |
| AMINO_SUGAR_AND_NUCLEOTIDE_SUGAR_METABOLISM | 47 | -0.5147307 | -1.6831356 | 0.002659574 | 0.022588948 |
| PENTOSE_AND_GLUCURONATE_INTERCONVERSIONS | 16 | -0.6651417 | -1.6821744 | 0.009975063 | 0.022153500 |
| DRUG_METABOLISM_OTHER_ENZYMES | 56 | -0.4914888 | -1.6606933 | 0.000000000 | 0.027197815 |
| LYSOSOME | 122 | -0.4247743 | -1.6485690 | 0.003105590 | 0.029977636 |
| NON_ALCOHOLIC_FATTY_LIVER_DISEASE_(NAFLD) | 145 | -0.4164187 | -1.6450669 | 0.000000000 | 0.029691488 |
| PEROXISOME | 78 | -0.4578171 | -1.6438245 | 0.002941176 | 0.028906679 |
| FATTY_ACID_DEGRADATION | 41 | -0.5052317 | -1.6415334 | 0.004750594 | 0.028542904 |
| ALANINE,_ASPARTATE_AND_GLUTAMATE_METABOLISM | 30 | -0.5470795 | -1.6407957 | 0.002551020 | 0.027784022 |
| SYSTEMIC_LUPUS_ERYTHEMATOSUS | 117 | -0.4306128 | -1.6390088 | 0.000000000 | 0.027354350 |
| CYTOKINE_CYTOKINE_RECEPTOR_INTERACTION | 211 | -0.3960312 | -1.6339558 | 0.000000000 | 0.027618919 |
| BETA_ALANINE_METABOLISM | 28 | -0.5469775 | -1.6283941 | 0.020304568 | 0.028295178 |
| RIBOSOME | 149 | -0.4079007 | -1.6059694 | 0.000000000 | 0.034120973 |
| PHENYLALANINE_METABOLISM | 16 | -0.6115105 | -1.5887628 | 0.034567900 | 0.038411766 |
| RETINOL_METABOLISM | 39 | -0.4931922 | -1.5593607 | 0.011904762 | 0.048732550 |
| LEUKOCYTE_TRANSENDOTHELIAL_MIGRATION | 106 | -0.4184410 | -1.5532364 | 0.005681818 | 0.050460152 |
| NATURAL_KILLER_CELL_MEDIATED_CYTOTOXICITY | 99 | -0.4141562 | -1.5517760 | 0.002840909 | 0.050017570 |
| VEGF_SIGNALING_PATHWAY | 58 | -0.4454053 | -1.5329237 | 0.000000000 | 0.057472040 |
| FC_EPSILON_RI_SIGNALING_PATHWAY | 61 | -0.4466009 | -1.5268699 | 0.005333333 | 0.058937468 |
| PARKINSON_DISEASE | 133 | -0.3924953 | -1.5224127 | 0.000000000 | 0.059462335 |
| ADIPOCYTOKINE_SIGNALING_PATHWAY | 64 | -0.4379740 | -1.5159737 | 0.010362694 | 0.061699560 |
| INSULIN_RESISTANCE | 101 | -0.3982464 | -1.4977200 | 0.000000000 | 0.069447440 |
| PRIMARY_BILE_ACID_BIOSYNTHESIS | 15 | -0.5968982 | -1.4969113 | 0.043902438 | 0.068394380 |
| CALCIUM_SIGNALING_PATHWAY | 167 | -0.3678696 | -1.4939686 | 0.006006006 | 0.068570980 |
| OVARIAN_STEROIDOGENESIS | 41 | -0.4595149 | -1.4886183 | 0.028277636 | 0.069807634 |
| ETHER_LIPID_METABOLISM | 41 | -0.4575913 | -1.4878703 | 0.010309278 | 0.068830356 |
| TYPE_II_DIABETES_MELLITUS | 42 | -0.4583874 | -1.4837737 | 0.005405406 | 0.069954050 |
| GLYCOSPHINGOLIPID_BIOSYNTHESIS_GANGLIO_SERIES | 15 | -0.5776730 | -1.4447482 | 0.082725060 | 0.094968470 |
| GLUTATHIONE_METABOLISM | 47 | -0.4419902 | -1.4434694 | 0.029484030 | 0.094232515 |
| INFLAMMATORY_MEDIATOR_REGULATION_OF_TRP_CHANNELS | 88 | -0.3841207 | -1.4336865 | 0.011049724 | 0.100416675 |
| CHOLESTEROL_METABOLISM | 42 | -0.4502297 | -1.4325995 | 0.026109660 | 0.099250130 |
| BIOSYNTHESIS_OF_UNSATURATED_FATTY_ACIDS | 27 | -0.4987567 | -1.4283376 | 0.072463766 | 0.100647370 |
| TYROSINE_METABOLISM | 32 | -0.4745273 | -1.4228991 | 0.039893616 | 0.102812800 |
| PHOSPHOLIPASE_D_SIGNALING_PATHWAY | 138 | -0.3570704 | -1.4200753 | 0.005730659 | 0.103140090 |
| FC_GAMMA_R_MEDIATED_PHAGOCYTOSIS | 89 | -0.3800546 | -1.4113920 | 0.017391304 | 0.108299820 |
| OTHER_GLYCAN_DEGRADATION | 18 | -0.5362620 | -1.4107624 | 0.069135800 | 0.107305840 |
| CITRATE_CYCLE_(TCA_CYCLE) | 30 | -0.4733595 | -1.4097345 | 0.056470588 | 0.106133200 |
| INTESTINAL_IMMUNE_NETWORK_FOR_IGA_PRODUCTION | 38 | -0.4519332 | -1.4082408 | 0.043373495 | 0.105573180 |
| PURINE_METABOLISM | 122 | -0.3567234 | -1.4024241 | 0.002958580 | 0.108457920 |

| | | | | | |
|---|---|---|---|---|---|
| PPAR_SIGNALING_PATHWAY | 64 | -0.4009843 | -1.3998756 | 0.034210525 | 0.108717560 |
| HEMATOPOIETIC_CELL_LINEAGE | 79 | -0.3893919 | -1.3988720 | 0.019607844 | 0.107663630 |
| ANTIFOLATE_RESISTANCE | 31 | -0.4636727 | -1.3970035 | 0.057692308 | 0.107492510 |
| GLYCOSAMINOGLYCAN_BIOSYNTHESIS_CHONDROITIN_SULFATE_/_DERMATAN_SULFATE | 20 | -0.5245104 | -1.3919725 | 0.064133020 | 0.109964960 |
| BUTANOATE_METABOLISM | 24 | -0.4854510 | -1.3731359 | 0.075060530 | 0.124703840 |
| INFLUENZA_A | 149 | -0.3488921 | -1.3664217 | 0.014880952 | 0.128705070 |
| NON_SMALL_CELL_LUNG_CANCER | 66 | -0.3815979 | -1.3581591 | 0.051075270 | 0.135622740 |
| GLYCEROLIPID_METABOLISM | 52 | -0.3973323 | -1.3557088 | 0.041558440 | 0.136508480 |
| GLYCOSPHINGOLIPID_BIOSYNTHESIS_LACTO_AND_NEOLACTO_SERIES | 25 | -0.4789693 | -1.3553953 | 0.086206900 | 0.134847670 |
| OXIDATIVE_PHOSPHORYLATION | 127 | -0.3490287 | -1.3514482 | 0.014662757 | 0.136723970 |
| CAMP_SIGNALING_PATHWAY | 182 | -0.3333354 | -1.3493005 | 0.009345794 | 0.136825840 |
| PYRUVATE_METABOLISM | 38 | -0.4272022 | -1.3430712 | 0.064516130 | 0.141725480 |
| PRIMARY_IMMUNODEFICIENCY | 31 | -0.4458249 | -1.3416079 | 0.073604060 | 0.141215000 |
| JAK_STAT_SIGNALING_PATHWAY | 121 | -0.3484663 | -1.3406802 | 0.032163743 | 0.140296380 |
| PLATINUM_DRUG_RESISTANCE | 68 | -0.3749844 | -1.3256845 | 0.067934780 | 0.154879240 |
| ALPHA_LINOLENIC_ACID_METABOLISM | 20 | -0.4832602 | -1.3209558 | 0.129411770 | 0.158850460 |
| CENTRAL_CARBON_METABOLISM_IN_CANCER | 61 | -0.3781401 | -1.3206730 | 0.058333334 | 0.157137040 |
| HIF_1_SIGNALING_PATHWAY | 94 | -0.3557498 | -1.3160166 | 0.038251366 | 0.160158600 |
| NICOTINATE_AND_NICOTINAMIDE_METABOLISM | 27 | -0.4436034 | -1.3141055 | 0.103686640 | 0.160045280 |
| PROTEIN_PROCESSING_IN_ENDOPLASMIC_RETICULUM | 158 | -0.3268047 | -1.3103749 | 0.024390243 | 0.163022560 |
| STARCH_AND_SUCROSE_METABOLISM | 31 | -0.4473287 | -1.3073328 | 0.119700745 | 0.165180180 |
| VALINE,_LEUCINE_AND_ISOLEUCINE_DEGRADATION | 46 | -0.4044118 | -1.3048762 | 0.070175440 | 0.166229980 |
| LEGIONELLOSIS | 54 | -0.3875036 | -1.3024219 | 0.072639230 | 0.167471330 |
| AMYOTROPHIC_LATERAL_SCLEROSIS_(ALS) | 47 | -0.3864511 | -1.3011973 | 0.066838050 | 0.167034420 |
| FATTY_ACID_ELONGATION | 27 | -0.4440093 | -1.3006350 | 0.086206900 | 0.165906790 |
| RAP1_SIGNALING_PATHWAY | 189 | -0.3199277 | -1.2998272 | 0.012618297 | 0.164993960 |
| HISTIDINE_METABOLISM | 21 | -0.4781089 | -1.2997003 | 0.119718310 | 0.163454120 |
| THIAMINE_METABOLISM | 16 | -0.4895103 | -1.2864429 | 0.144278600 | 0.177415890 |
| CARDIAC_MUSCLE_CONTRACTION | 71 | -0.3633528 | -1.2852060 | 0.070528970 | 0.176947010 |
| THERMOGENESIS | 221 | -0.3058897 | -1.2835689 | 0.017301038 | 0.177060870 |
| PERTUSSIS | 73 | -0.3632758 | -1.2835451 | 0.064245810 | 0.175196300 |
| PROXIMAL_TUBULE_BICARBONATE_RECLAMATION | 19 | -0.4734367 | -1.2704505 | 0.134663340 | 0.189381050 |
| SPHINGOLIPID_SIGNALING_PATHWAY | 117 | -0.3297332 | -1.2701705 | 0.052478135 | 0.187797860 |
| RENIN_ANGIOTENSIN_SYSTEM | 21 | -0.4632871 | -1.2657450 | 0.161764710 | 0.191956650 |
| ABC_TRANSPORTERS | 42 | -0.3974114 | -1.2639980 | 0.128608930 | 0.192173630 |
| VIRAL_CARCINOGENESIS | 191 | -0.3061597 | -1.2585855 | 0.049019610 | 0.197225720 |
| AXON_GUIDANCE | 171 | -0.3074399 | -1.2579639 | 0.036544852 | 0.196184600 |
| AMPK_SIGNALING_PATHWAY | 115 | -0.3296958 | -1.2532026 | 0.059347183 | 0.199736920 |
| HUNTINGTON_DISEASE | 183 | -0.3051923 | -1.2484401 | 0.045592707 | 0.204409440 |
| VIRAL_MYOCARDITIS | 55 | -0.3651846 | -1.2479844 | 0.086720870 | 0.202973190 |
| CHOLINE_METABOLISM_IN_CANCER | 96 | -0.3275338 | -1.2382607 | 0.072222225 | 0.214246690 |

| | | | | | |
|---|---|---|---|---|---|
| GLYCEROPHOSPHOLIPID_METABOLISM | 87 | -0.3387934 | -1.2342460 | 0.109625670 | 0.218803640 |
| BASE_EXCISION_REPAIR | 33 | -0.4082963 | -1.2322102 | 0.142506140 | 0.219976510 |
| GLYCINE,_SERINE_AND_THREONINE_METABOLISM | 36 | -0.4011854 | -1.2318119 | 0.151219520 | 0.218425300 |
| ERBB_SIGNALING_PATHWAY | 81 | -0.3435532 | -1.2296628 | 0.111688310 | 0.219587770 |
| ALZHEIMER_DISEASE | 167 | -0.3053984 | -1.2253475 | 0.067278290 | 0.223810580 |
| HUMAN_IMMUNODEFICIENCY_VIRUS_1_INFECTION | 192 | -0.2988579 | -1.2232958 | 0.067961164 | 0.224583220 |
| C_TYPE_LECTIN_RECEPTOR_SIGNALING_PATHWAY | 97 | -0.3250908 | -1.2228960 | 0.085635360 | 0.223219620 |
| CYSTEINE_AND_METHIONINE_METABOLISM | 46 | -0.3706465 | -1.2223815 | 0.148717950 | 0.221975450 |
| EPSTEIN_BARR_VIRUS_INFECTION | 183 | -0.3013155 | -1.2207065 | 0.045307443 | 0.222694930 |
| CELL_ADHESION_MOLECULES_(CAMS) | 136 | -0.3126775 | -1.2184404 | 0.073746316 | 0.224025370 |
| INFLAMMATORY_BOWEL_DISEASE_(IBD) | 51 | -0.3641039 | -1.2147500 | 0.155495970 | 0.227298560 |
| INSULIN_SIGNALING_PATHWAY | 130 | -0.3101356 | -1.2101622 | 0.097058825 | 0.231128750 |
| NEUROACTIVE_LIGAND_RECEPTOR_INTERACTION | 215 | -0.2909402 | -1.2043150 | 0.054545455 | 0.237504170 |
| REGULATION_OF_LIPOLYSIS_IN_ADIPOCYTES | 49 | -0.3618686 | -1.1948229 | 0.154228850 | 0.249939100 |
| PYRIMIDINE_METABOLISM | 54 | -0.3528065 | -1.1941708 | 0.170792070 | 0.248834120 |
| MINERAL_ABSORPTION | 42 | -0.3720032 | -1.1913689 | 0.178217830 | 0.251258760 |
| FLUID_SHEAR_STRESS_AND_ATHEROSCLEROSIS | 132 | -0.3013558 | -1.1909375 | 0.096096100 | 0.249885070 |
| OSTEOCLAST_DIFFERENTIATION | 116 | -0.3073788 | -1.1906033 | 0.106628240 | 0.248433950 |
| MEASLES | 121 | -0.3094420 | -1.1849867 | 0.107692310 | 0.255198700 |
| HUMAN_PAPILLOMAVIRUS_INFECTION | 306 | -0.2716409 | -1.1763369 | 0.046099290 | 0.267557830 |
| EGFR_TYROSINE_KINASE_INHIBITOR_RESISTANCE | 77 | -0.3264856 | -1.1763248 | 0.159459460 | 0.265382560 |
| MAPK_SIGNALING_PATHWAY | 275 | -0.2725282 | -1.1687030 | 0.073684210 | 0.275641260 |
| B_CELL_RECEPTOR_SIGNALING_PATHWAY | 70 | -0.3264826 | -1.1559585 | 0.187335090 | 0.295969200 |
| ENDOCRINE_AND_OTHER_FACTOR_REGULATED_CALCIUM_REABSORPTION | 41 | -0.3574399 | -1.1547987 | 0.220095700 | 0.295691300 |
| HEPATITIS_C | 137 | -0.2935676 | -1.1505594 | 0.138643070 | 0.300748400 |
| ECM_RECEPTOR_INTERACTION | 78 | -0.3154752 | -1.1413789 | 0.183673470 | 0.315882120 |
| BASAL_CELL_CARCINOMA | 59 | -0.3298688 | -1.1392858 | 0.200527700 | 0.317410200 |
| MANNOSE_TYPE_O_GLYCAN_BIOSYNTHESIS | 21 | -0.4177033 | -1.1310070 | 0.262086500 | 0.330663900 |
| TOXOPLASMOSIS | 106 | -0.3044662 | -1.1276355 | 0.189701900 | 0.334974350 |
| RAS_SIGNALING_PATHWAY | 203 | -0.2743125 | -1.1269833 | 0.124590166 | 0.333702620 |
| PRION_DISEASES | 32 | -0.3745491 | -1.1267147 | 0.260975600 | 0.331684770 |
| CHAGAS_DISEASE_(AMERICAN_TRYPANOSOMIASIS) | 95 | -0.3048780 | -1.1220087 | 0.193905820 | 0.337944200 |
| ALDOSTERONE_REGULATED_SODIUM_REABSORPTION | 32 | -0.3723760 | -1.1209319 | 0.267002520 | 0.337524440 |
| FOXO_SIGNALING_PATHWAY | 126 | -0.2897119 | -1.1186079 | 0.213675220 | 0.339520630 |
| HEPATOCELLULAR_CARCINOMA | 158 | -0.2795597 | -1.1136484 | 0.150769230 | 0.346957240 |
| OTHER_TYPES_OF_O_GLYCAN_BIOSYNTHESIS | 22 | -0.3954251 | -1.1107203 | 0.272093030 | 0.350238080 |
| PHAGOSOME | 140 | -0.2812355 | -1.1086347 | 0.209770110 | 0.351689460 |
| ASTHMA | 21 | -0.4025746 | -1.0985887 | 0.295121940 | 0.371135100 |
| HUMAN_CYTOMEGALOVIRUS_INFECTION | 205 | -0.2635377 | -1.0965077 | 0.167224080 | 0.373267050 |
| ENDOCYTOSIS | 240 | -0.2627726 | -1.0963826 | 0.193430660 | 0.370853220 |
| TH17_CELL_DIFFERENTIATION | 95 | -0.2971745 | -1.0919672 | 0.239554320 | 0.377963130 |

| | | | | | |
|---|---|---|---|---|---|
| GLYOXYLATE_AND_DICARBOXYLATE_METABOLISM | 27 | -0.3715660 | -1.0862017 | 0.313539200 | 0.388313260 |
| GLIOMA | 74 | -0.3033951 | -1.0829052 | 0.268617030 | 0.393233150 |
| ENDOMETRIAL_CANCER | 58 | -0.3150016 | -1.0816427 | 0.288311700 | 0.393254280 |
| TOLL_LIKE_RECEPTOR_SIGNALING_PATHWAY | 88 | -0.2925549 | -1.0786307 | 0.292134820 | 0.396938740 |
| PROTEOGLYCANS_IN_CANCER | 192 | -0.2632765 | -1.0777968 | 0.247524750 | 0.396170530 |
| PLATELET_ACTIVATION | 119 | -0.2788812 | -1.0774012 | 0.235772360 | 0.394423220 |
| ALCOHOLISM | 163 | -0.2683263 | -1.0763118 | 0.274576280 | 0.394150880 |
| ESTROGEN_SIGNALING_PATHWAY | 120 | -0.2762115 | -1.0672239 | 0.264331220 | 0.411835370 |
| NEUROTROPHIN_SIGNALING_PATHWAY | 115 | -0.2819871 | -1.0658495 | 0.290502800 | 0.412194200 |
| N_GLYCAN_BIOSYNTHESIS | 48 | -0.3179286 | -1.0638539 | 0.314356450 | 0.414059800 |
| APOPTOSIS | 132 | -0.2702236 | -1.0573157 | 0.286644970 | 0.426709380 |
| FERROPTOSIS | 39 | -0.3359644 | -1.0568261 | 0.334177230 | 0.425258520 |
| REGULATION_OF_ACTIN_CYTOSKELETON | 198 | -0.2566118 | -1.0519344 | 0.294670850 | 0.434339520 |
| THYROID_CANCER | 37 | -0.3404059 | -1.0517904 | 0.362944160 | 0.431923720 |
| NECROPTOSIS | 144 | -0.2661188 | -1.0477219 | 0.308333340 | 0.438963350 |
| RENAL_CELL_CARCINOMA | 65 | -0.2908222 | -1.0402874 | 0.348571420 | 0.454151840 |
| MALARIA | 41 | -0.3253453 | -1.0361768 | 0.370466320 | 0.461053700 |
| TUBERCULOSIS | 156 | -0.2603973 | -1.0345379 | 0.334426220 | 0.462660900 |
| PANCREATIC_CANCER | 75 | -0.2872846 | -1.0341746 | 0.367688030 | 0.460635240 |
| ALDOSTERONE_SYNTHESIS_AND_SECRETION | 87 | -0.2798176 | -1.0291182 | 0.348703180 | 0.470304220 |
| INSULIN_SECRETION | 72 | -0.2865239 | -1.0259483 | 0.404371600 | 0.475028750 |
| ARRHYTHMOGENIC_RIGHT_VENTRICULAR_CARDIOMYOPATHY_(ARVC) | 70 | -0.2899045 | -1.0234582 | 0.379603400 | 0.478718730 |
| ACUTE_MYELOID_LEUKEMIA | 62 | -0.2866652 | -1.0154976 | 0.395894440 | 0.495520400 |
| TNF_SIGNALING_PATHWAY | 107 | -0.2636427 | -1.0079810 | 0.440318320 | 0.512366700 |
| CHEMOKINE_SIGNALING_PATHWAY | 168 | -0.2475723 | -1.0004078 | 0.460317460 | 0.529191400 |
| NOTCH_SIGNALING_PATHWAY | 47 | -0.3013955 | -0.9961230 | 0.459948330 | 0.537405900 |
| IL_17_SIGNALING_PATHWAY | 79 | -0.2753728 | -0.9839990 | 0.459610020 | 0.567203040 |
| AMOEBIASIS | 85 | -0.2692584 | -0.9798918 | 0.460227280 | 0.575001960 |
| INOSITOL_PHOSPHATE_METABOLISM | 71 | -0.2755332 | -0.9775584 | 0.479564040 | 0.578365500 |
| ENDOCRINE_RESISTANCE | 94 | -0.2597407 | -0.9738233 | 0.519021750 | 0.585338200 |
| THYROID_HORMONE_SYNTHESIS | 62 | -0.2812413 | -0.9705275 | 0.497382200 | 0.590583500 |
| AUTOPHAGY_ANIMAL | 123 | -0.2531570 | -0.9688863 | 0.527950300 | 0.591783300 |
| PROLACTIN_SIGNALING_PATHWAY | 63 | -0.2767168 | -0.9679014 | 0.488311680 | 0.590969100 |
| TH1_AND_TH2_CELL_DIFFERENTIATION | 81 | -0.2666145 | -0.9630224 | 0.538674060 | 0.601246400 |
| SPHINGOLIPID_METABOLISM | 45 | -0.2894295 | -0.9593853 | 0.501272260 | 0.608126000 |
| HYPERTROPHIC_CARDIOMYOPATHY_(HCM) | 82 | -0.2656406 | -0.9536029 | 0.535519100 | 0.621249500 |
| T_CELL_RECEPTOR_SIGNALING_PATHWAY | 88 | -0.2561338 | -0.9423973 | 0.591891900 | 0.649352970 |
| RIG_I_LIKE_RECEPTOR_SIGNALING_PATHWAY | 54 | -0.2791851 | -0.9315450 | 0.595567900 | 0.675578500 |
| AMINOACYL_TRNA_BIOSYNTHESIS | 44 | -0.2864379 | -0.9231307 | 0.569711570 | 0.695954560 |
| VIBRIO_CHOLERAE_INFECTION | 47 | -0.2808882 | -0.9216883 | 0.568000000 | 0.696012440 |
| DILATED_CARDIOMYOPATHY_(DCM) | 85 | -0.2544757 | -0.9205792 | 0.659517400 | 0.695381500 |

| | | | | | |
|---|---|---|---|---|---|
| TRYPTOPHAN_METABOLISM | 43 | -0.2801380 | -0.9198942 | 0.601467000 | 0.693481560 |
| SALMONELLA_INFECTION | 81 | -0.2482373 | -0.9182283 | 0.657223800 | 0.694098100 |
| GLYCOSAMINOGLYCAN_DEGRADATION | 18 | -0.3485469 | -0.9168951 | 0.561445800 | 0.694111650 |
| LEISHMANIASIS | 66 | -0.2629819 | -0.9164488 | 0.649456500 | 0.691468900 |
| BILE_SECRETION | 55 | -0.2668008 | -0.8992962 | 0.666666700 | 0.733464400 |
| VITAMIN_DIGESTION_AND_ABSORPTION | 19 | -0.3380592 | -0.8966253 | 0.618581900 | 0.736856400 |
| MITOPHAGY_ANIMAL | 63 | -0.2527371 | -0.8795934 | 0.718421040 | 0.775924150 |
| APOPTOSIS_MULTIPLE_SPECIES | 31 | -0.2874987 | -0.8781193 | 0.667532440 | 0.775481640 |
| CYTOSOLIC_DNA_SENSING_PATHWAY | 49 | -0.2613117 | -0.8692758 | 0.724310760 | 0.792437100 |
| AUTOPHAGY_OTHER | 31 | -0.2731261 | -0.8456846 | 0.758104740 | 0.840400040 |
| SNARE_INTERACTIONS_IN_VESICULAR_TRANSPORT | 34 | -0.2695399 | -0.8092166 | 0.810256400 | 0.903201040 |
| NF_KAPPA_B_SIGNALING_PATHWAY | 88 | -0.2202461 | -0.8049725 | 0.925729450 | 0.905270640 |
| AFRICAN_TRYPANOSOMIASIS | 33 | -0.2520601 | -0.7839755 | 0.865822800 | 0.929529300 |
| PANTOTHENATE_AND_COA_BIOSYNTHESIS | 16 | -0.2999851 | -0.7759533 | 0.802884640 | 0.934284570 |
| PROPANOATE_METABOLISM | 32 | -0.2464302 | -0.7524998 | 0.905213300 | 0.953022960 |
| GLYCOSAMINOGLYCAN_BIOSYNTHESIS_HEPARAN_SULFATE_/_HEPARIN | 22 | -0.2593738 | -0.7210922 | 0.881355940 | 0.970565800 |
| ONE_CARBON_POOL_BY_FOLATE | 20 | -0.2581922 | -0.6990164 | 0.915662650 | 0.976956960 |
| SELENOCOMPOUND_METABOLISM | 18 | -0.2381074 | -0.6224972 | 0.935162070 | 0.992344700 |

Legend: KEGG pathways of differentially expressed genes that were altered significantly after METTL3 knockout, p-values <0.05 were indicated.

Supplementary Table 5. Metabolites involved in METTL3 knockout in MCF-7

| Level2 | Level1 | Pathway | KEGG | NumberCompound | NumberFeature | Compound | Feature |
|---|---|---|---|---|---|---|---|
| Amino acid metabolism | Metabolism | Glycine, serine and threonine metabolism; Arginine biosynthesis; Alanine, aspartate and glutamate metabolism; Cysteine and methionine metabolism; Lysine biosynthesis; Histidine metabolism; Arginine and proline metabolism | map00260; map00220; map00250; map00270; map00300; map00340; map00330 | 9 | 11 | C00049; C01042; C01682; C01035; C03406; C00740; C05570; C03145; C00631 | pos-0.899_304.13816; neg-0.857_290.12246; pos-1.374_145.08496; neg-3.455_177.04577; neg-0.912_175.0478; neg-0.839_133.03726; neg-0.837_105.04214; pos-1.363_304.13821; pos-1.327_105.04254; neg-0.865_185.99259; |
| Biosynthesis of other secondary metabolites | Metabolism | Monobactam biosynthesis Biosynthesis of various secondary metabolites - part 3; Clavulanic acid biosynthesi | map00261; map00997; map00331 | 2 | 2 | C00049; C06655 | neg-0.839_133.03726; neg-0.86_246.1325 |
| Cancer: overview | Human Diseases | Central carbon metabolism in cancer; Choline metabolism in cancer | map05230; map05231 | 4 | 4 | C00049; C00093; C00631; C00670 | neg-0.839_133.03726; neg-0.857_172.01339; neg-0.865_185.99259; pos-0.849_257.10254 |
| Carbohydrate metabolism | Metabolism | Glycolysis / Gluconeogenesis Pentose phosphate pathway; Glyoxylate and dicarboxylate metabolism; Galactose metabolism; Amino sugar and nucleotide sugar metabolism | map00010; map00030; map00630; map00052; map00520 | 2 | 3 | C00052; C00631 | neg-0.909_566.05511; neg-1.008_566.05514; neg-0.865_185.99259 |
| Cell motility | Cellular Processes | Bacterial chemotaxis | map02030 | 1 | 1 | C00049 | neg-0.839_133.03726 |
| Chemical structure transformation maps | Metabolism | Biosynthesis of plant secondary metabolites; Biosynthesis of alkaloids derived from ornithine, lysine and nicotinic acid; Biosynthesis of plant hormones; Biosynthesis of phenylpropanoids; Biosynthesis of terpenoids and steroids; Biosynthesis of alkaloids derived from shikimate pathwa | map01060; map01064; map01070; map01061; map01062; map01063 | 2 | 2 | C00049; C00631 | neg-0.839_133.03726; neg-0.865_185.99259 |
| Digestive system | Organismal Systems | Protein digestion and absorption | map04974 | 1 | 1 | C00049 | neg-0.839_133.03726 |
| Drug resistance: antimicrobial | Human Diseases | Vancomycin resistance | map01502 | 1 | 2 | C00740 | pos-1.327_105.04254; neg-0.837_105.04214 |
| Endocrine and metabolic disease | Human Diseases | Insulin resistance | map04931 | 1 | 1 | C02571 | neg-3.788_203.11557 |
| Endocrine system | Organismal Systems | Glucagon signaling pathway | map04922 | 1 | 1 | C00631 | neg-0.865_185.99259 |
| Energy metabolism | Metabolism | Carbon fixation in photosynthetic organisms Methane metabolism | map00710; map00680 | 2 | 2 | C00049; C00631 | neg-0.839_133.03726; neg-0.865_185.99259 |
| Global and overview maps | Metabolism | Metabolic pathways; Biosynthesis of secondary metabolites; Microbial metabolism in diverse environments; Carbon metabolism; 2-Oxocarboxylic acid metabolism; Biosynthesis of amino acids; Biosynthesis of cofactors | map01100; map01110; map01120; map01200; map01210; map01230; map01240 | 17 | 24 | C00049; C01042; C00052; C00112; C00299; C17947; C06655; C03406; C00106; C14519; C01368; C01035; C01596; C00093; C00740; C05570; C00631 | neg-0.857_290.12246; neg-0.978_212.00824; neg-0.837_105.04214; pos-1.363_324.03551; pos-1.327_105.04254; neg-0.89_403.01788; neg-0.865_185.99259; pos-0.899_229.08831; neg-0.86_246.1325; neg-0.912_175.0478; neg-0.919_115.02663; neg-1.384_115.02663; neg-0.857_172.01339; pos-1.374_145.08496; neg-0.909_566.05511; neg-1.548_244.06945; neg-0.916_324.0357; pos-3.257_218.0727; pos-1.528_112.02721; neg-3.282_218.0724; neg-0.839_133.03726; neg-1.008_566.05514; |
| Glycan biosynthesis and metabolism | Metabolism | O-Antigen nucleotide sugar biosynthesis | map00541 | 1 | 2 | C00052 | neg-0.909_566.05511; neg-1.008_566.05514 |
| Lipid metabolism | Metabolism | Glycerolipid metabolism; Glycerophospholipid metabolism; Ether lipid metabolism; Biosynthesis of unsaturated fatty acids | map00561; map00564; map00565; map01040 | 4 | 4 | C06428; C00631; C00670; C00093 | pos-7.327_302.22438; neg-0.857_172.01339; neg-0.865_185.99259; pos-0.849_257.10254 |

| | | | | | | | |
|---|---|---|---|---|---|---|---|
| Membrane transport | Environmental Information Processing | ABC transporters | map02010 | 4 | 5 | C00299; C00049; C01682; C00093 | pos-0.899_304.13816; neg-0.857_172.01339; neg-0.839_133.03726; neg-1.548_244.06945; pos-1.363_304.13821 |
| Metabolism of cofactors and vitamins | Metabolism | Nicotinate and nicotinamide metabolism; Pantothenate and CoA biosynthesis | map00760; map00770 | 3 | 5 | C00049; C00106; C01596 | pos-1.528_112.02721; neg-0.919_115.02663; neg-0.839_133.03726; neg-1.384_115.02663; pos-1.371_112.0272 |
| Metabolism of other amino acids | Metabolism | beta-Alanine metabolism; Cyanoamino acid metabolism; Phosphonate and phosphinate metabolism | map00410; map00460; map00440 | 3 | 4 | C00049; C17947; C00106 | neg-0.839_133.03726; neg-0.978_212.00824; pos-1.371_112.0272; pos-1.528_112.02721 |
| Nucleotide metabolism | Metabolism | Pyrimidine metabolism | map00240 | 4 | 7 | C00299; C01368; C00112; C00106 | pos-1.528_112.02721; neg-1.548_244.06945; pos-1.363_324.03551; pos-1.371_112.0272; pos-0.902_324.03535; neg-0.916_324.0357; neg-0.89_403.01788 |
| Sensory system | Organismal Systems | Taste transduction | map04742 | 1 | 2 | C00740 | pos-1.327_105.04254; neg-0.837_105.04214 |
| Signal transductior | Environmental Information Processing | Two-component system | map02020 | 1 | 1 | C00049 | neg-0.839_133.03726 |
| Signaling molecules and interactio | Environmental Information Processing | Neuroactive ligand-receptor interaction | map04080 | 1 | 1 | C00049 | neg-0.839_133.03726 |
| Translation | Genetic Information Processing | Aminoacyl-tRNA biosynthesi | map00970 | 1 | 1 | C00049 | neg-0.839_133.03726 |
| Xenobiotics biodegradation and metabolism | Metabolism | Polycyclic aromatic hydrocarbon degradation | map00624 | 1 | 2 | C14519 | pos-3.257_218.0727; neg-3.282_218.0724 |

Legend: KEGG pathways of different metabolites that were altered significantly after knocking out METTL3 expression, p-values <0.05 were indicate
